# Supplementary material for: Facile access to benzofuran derivatives through radical reactions with heteroatom-centered super-electron-donors
Source: Nat Commun. 2023 Nov 15;14:7381. doi: 10.1038/s41467-023-43198-y (PMC10651860; doi:10.1038/s41467-023-43198-y)
Supplement: Supplementary file 1 — Supplementary Information [file 41467_2023_43198_MOESM1_ESM.pdf]

Supplementary Information

Facile Access to Benzofuran Derivatives through Radical Reactions with Heteroatom-centered Super-electron-donors

Shichun Jiang[1†], Wei Wang[1†], Chengli Mou[2†], Juan Zou[2], Zhichao Jin[1,*], Gefei Hao[1,*] and Yonggui Robin Chi[1,3,*]

Affiliations:

[1]National Key Laboratory of Green Pesticide, Key Laboratory of Green Pesticide and Agricultural Bioengineering, Ministry of Education, Guizhou University, Guiyang 550025, China.

[2]Guizhou University of Traditional Chinese Medicine, Guiyang 550025, China.

[3]School of Chemistry, Chemical Engineering, and Biotechnology, Nanyang Technological University, Singapore 637371, Singapore.

*Corresponding author. Email:

zcjin@gzu.edu.cn;

gefeihao@foxmail.com;

robinchi@ntu.edu.sg.

Table of Contents

Ⅰ. Supplementary Methods 3
General Information 3
Experimental Section 4
Mechanistic Considerations 11
Synthetic Transformations 24
Antibacterial Activity in *Vitro* 27
Ⅱ. Supplementary Notes 34
Characterization of Products 34
Ⅲ. Supplementary Figures 65
Stereochemistry Determination by *X*-ray Crystallographic Analysis 65
^{1}H NMR, ^{13}C NMR, ^{19}F NMR and ^{31}P NMR Spectra 66
Ⅳ. Predicted Cytotoxicity of The Compounds Against Human Cells 164

Ⅰ. Supplementary Methods

General Information

Commercially available materials purchased from Energy Chemical, J&K and Bide were used as received. All the dry solvents were directly bought from Energy Chemical. Unless otherwise specified, all reactions were carried out under an atmosphere of N_2 in 10 mL threaded anaerobic tube. NMR spectra were measured either on a JEOL-ECX 500 (500 MHz) or on a Bruker ASCEND 400 (400 MHz) spectrometer. The chemical shift values were corrected to 7.26 ppm (^{1}H NMR) and 77.16 ppm (^{13}C NMR) for $CDCl_3$. ^{1}H NMR splitting patterns are designated as singlet (s), double (d), triplet (t), quartet (q), doublet of doublets (dd), multiplets (m), and etc. All first-order splitting patterns were assigned on the base of the appearance of the multiplet. Splitting patterns that could not be easily interpreted are designated as multiplet (m) or broad (br). High resolution mass spectrometer analysis (HRMS) was performed on Thermo Fisher Q Exactive mass spectrometer. Melting Point (MP): Melting points were measured on a Beijing Tech Instrument X-4 digital display micro melting point apparatus and are uncorrected. Electron paramagnetic resonance spectroscopy spectra were measured on Chinainstru&Quantumtech (Hefei) EPR200-Plus with continues-wave X band frequency. Voltammetry experiments (CV, DPV) were performed using a CH Instruments 600E Electrochemical Analyzer/Workstation and the data were processed using CHI software v9.24. Analytical thin-layer chromatography (TLC) was carried out on pre-coated silica gel plate (0.2 mm thickness). Visualization was performed using a UV lamp.

Experimental Section

1. Preparation of substrates

Part 1. General procedure for the preparation of aryl allene ether 1[1]

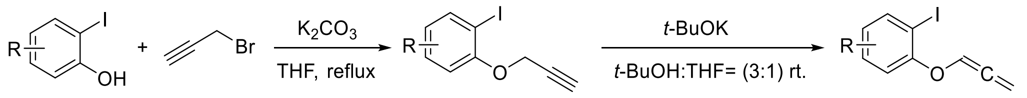

A suspension of 2-iodophenol (1.0 eq.), propargyl bromide (1.2 eq.) and potassium carbonate (1.2 eq.) in THF was refluxed for 6 h. Water and ethyl acetate were then added to the reaction layers and the organic layers was separated, dried (Na_2SO_4), evaporated under reduced pressure and the residue dissolved in *t*-butyl alcohol – THF (3:1 v/v). Potassium *t*-butoxide (1.2 eq.) was then added and the resulting mixture was stirred at room temperature for 16 h. when the solvent was evaporated under reduced pressure and dichloromethane added. The organic layer was separated and washed with water, dried (Na_2SO_4) and evaporated under reduced pressure. The residue was purified by silica gel chromatography (PE / EA as eluent) to give the corresponding aryl allene ether.

Part 2. General procedure for the preparation of aryl allene ether 1[2]

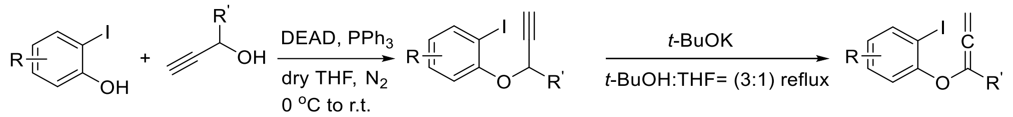

Step 1. An oven dried round-bottom equipped with a magnetic stirring bar was charged with propargylic alcohol (1.0 eq.), THF were added by using syringes, *o*-iodophenol (1.0 eq.), triphenylphosphine (1.1 eq.) were added to the above solution, and the flask was set under N_2 through three cycles of evacuation and recompression with N_2. Then the solution was cooled to 0 ℃ and diethyl azodicarboxylate (DEAD, 1.2 eq.) was added dropwise via syringe. The ice bath was removed and stirred under argon for overnight. The mixture was extracted three times with ethyl acetate. The combined organic fractions were dried over Na_2SO_4 and concentrated under vacuum. The residue was purified by silica gel chromatography (PE / EA as eluent) to give the corresponding propargylic ethers.

Step 2. The corresponding propargylic ethers dissolved in *t*-butyl alcohol – THF (3:1 v / v). Potassium *t*-butoxide (1.2 eq.) was then added and the resulting mixture was stirred under reflux for 6 h. when the solvent was evaporated under reduced pressure and dichloromethane added. The organic layer was separated and washed with water, dried (Na_2SO_4) and evaporated under reduced

pressure. The residue was purified by silica gel chromatography (PE / EA as eluent) to give the corresponding aryl allene ether.

2. Condition optimization for the synthesis of 3a

Supplementary Table 1. Condition optimization for the synthesis of 3a[a]

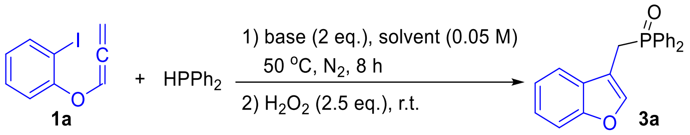

Entry	Base	Solvent	X	Temp. (°C)	Yield[b] (%)
**1**	Cs_2CO_3 / NaOH / DBU / DIEA etc.	DME	I	30	NR
**2**	KO^{t}Bu	DME	I	30	16[c]
**3**	NaHMDS	DME	I	30	17[c]
**4**	LiHMDS	DME	I	30	71[c]
**5**	$KHMDS_2$	DME	I	30	2[c]
**6**	LDA	DME	I	30	76[c]
**7**	LDA (0.5 eq)	DME	I	30	22[c]
**8**	LDA (1.0 eq)	DME	I	30	41[c]
**9**	LDA (1.5 eq)	DME	I	30	68[c]
**10**	LDA (2.5 eq)	DME	I	30	71[c]
**11**	LDA	EA / CH_2Cl_2 / Hexane / $CDCl_3$ / CCl_4	I	30	NR
**12**	LDA	1,4-Dioxane	I	30	37
**13**	LDA	THF	I	30	65
**14**	LDA	CH_3CN	I	30	22
**15**	LDA	MTBE	I	30	20
**16**	LDA	Et_2O	I	30	30
**17**	LDA	Toluene	I	30	15
**18**	LDA	$PhCF_3$	I	30	20
**19**	LDA	DME	I	30	76
**20**	LDA	$(CH_2Cl)_2$	I	30	15
**21**	LDA	Cyclohexane	I	30	8
**22**	LDA	DMSO	I	30	60
**23**	LDA	DMF	I	30	27
**24**	LDA	DME	I	0	59
**25**	LDA	DME	I	50	80
**26**	LDA	DME	I	60	74
**27**	LDA	DME (0.10 M)	I	50	69
**28**	LDA	DME	Br / Cl	50	trace

[a] All reactions were performed using **1a** (0.10 mmol), $HPPh_2$ (0.20 mmol), base (0.20 mmol), solvent (2.0 mL) for 8 h under N_2. H_2O_2 (0.25 mmol) was added into reaction system at 0 ℃, then warm the system to room temperature, and react for 1 h. [b] Yields of isolated products after column chromatography. [c] Assay yields determined by ^{1}HNMR spectroscopy of the crude reaction mixtures using 1,3,5-Trimethoxybenzene as an internal standard. DBU = 1,8-Dizabicyclo[5.4.0]undec-7-ene. DIEA = *N*,*N*-Diisopropylethylamine. HMDS = hexamethydisilylamine. LDA = Lithium diisopropylamide. THF = Tetrahydrofuran. MTBE = *tert*-Butyl methyl ether. DME = 1,2-Dimethoxyethane.

3. Condition optimization for the synthesis of 3c

Supplementary Table 2. Condition optimization for the synthesis of 3c[a]

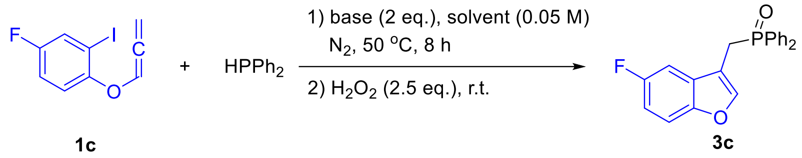

Entry	Base (2 eq.)	Solvent	Yield[b] (%)
**1**	LDA	DME	50
**2**	NaHMDS	DME	48
**3**	LiHMDS	DME	63
**4**	KHMDS	DME	24
**5**	LiHMDS	1,4-Dioxane	17
**6**	LiHMDS	THF	41
**7**	LiHMDS	CH_3CN	7
**8**	LiHMDS	MTBE	34
**9**	LiHMDS	Toluene	15
**10**	LiHMDS	$PhCF_3$	13

[a] All reactions were performed using **1c** (0.10 mmol), $HPPh_2$ (0.20 mmol), base (0.20 mmol), solvent (2 mL) at 50 ℃ for 8 h under N_2. H_2O_2 (0.25 mmol) was added into reaction system at 0 ℃, then warm the system to room temperature, and react for 1 h; [b] Yields of isolated products after column chromatography.

4. Air stability test for the benzofurymethyldiphenylphosphine

Control experiments to systematically compare the stabilities of the benzofurymethyl diphenylphosphine product **2a** and the diphenylmethyl phosphine. Stark contrast could be observed in their deterioration rates in THF-*d8* after a certain period (**Supplementary Figure 1** and **Supplementary Figure 2**).

The less stability of the product **2** than the diphenylmethyl phosphine is believed to be attributed to the strong electron-donating benzofuran group existed in the product structure. The electron density on the P atom of the product **2** could be increased by the benzofuran substituted methylene group, so that the reducing ability of the product **2** is stronger than the diphenylmethyl phosphine and could be more easily oxidized in air conditions.

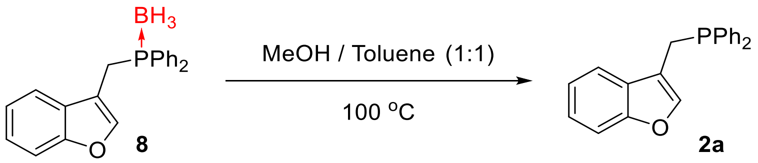

To a 10 mL anaerobic tube with a magnetic stir bar was added **8** (0.15 mmol) and 2 mL solvent (MeOH : Toluene = 1 : 1) at 100 ℃ for 24 h under N_2. After the borane-protected phosphorus compound was reduced, the solvent and trimethyl borate were evaporated under reduced pressure. ^{31}P NMR spectroscopy was used to analyze the stabilities of the benzofurymethyl diphenylphosphine product **2a** and diphenylmethyl phosphine under N_2 condition and the

stabilities after exposure to air for 2 h, 6 h, 12 h, 24 h and 48 h.

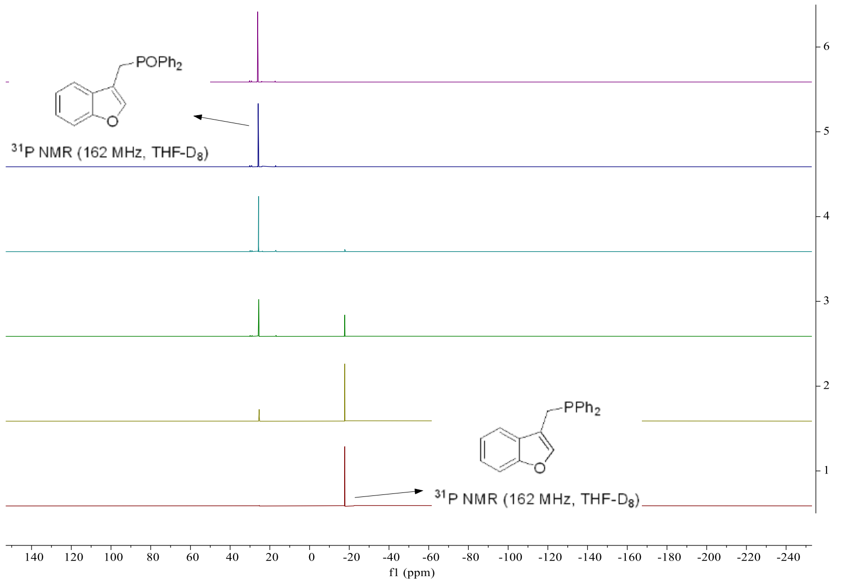

Supplementary Figure 1. (1) ^{31}P NMR spectroscopy of compound benzofurymethyl diphenylphosphine under N_2 condition. (2) ^{31}P NMR spectrum after 2 h in air. (3) ^{31}P NMR spectrum after 6 h in air. (4) ^{31}P NMR spectrum after 12 h in air. (5) ^{31}P NMR spectrum after 24 h in air. (6) ^{31}P NMR spectrum after 48 h in air.

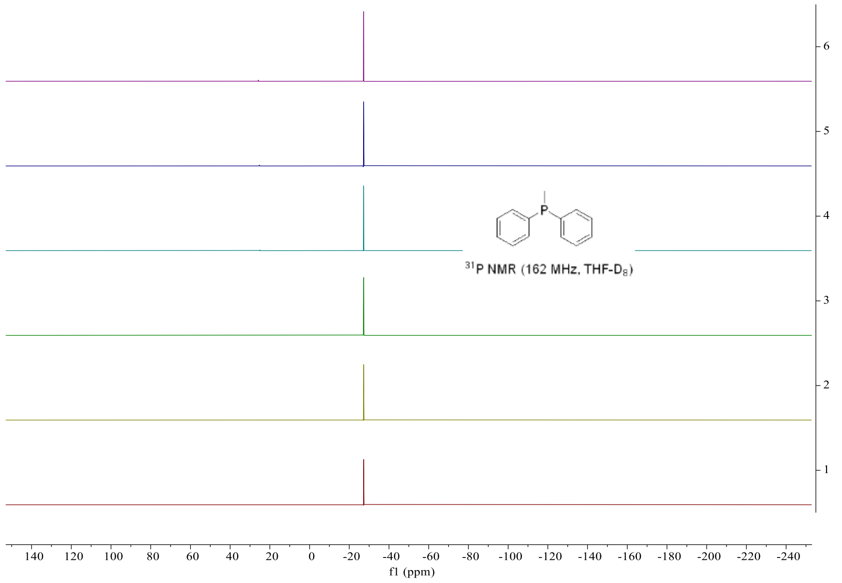

Supplementary Figure 2. (1) ^{31}P NMR spectroscopy of compound diphenylmethylphosphine under N_2 condition. (2) ^{31}P NMR spectrum after 2 h in air. (3) ^{31}P NMR spectrum after 6 h in air. (4) ^{31}P NMR spectrum after 12 h in air. (5) ^{31}P NMR spectrum after 24 h in air. (6) ^{31}P NMR spectrum after 48 h in air.

5. Condition optimization for the synthesis of 5a

Supplementary Table 3. Condition optimization for the synthesis of 5a[a]

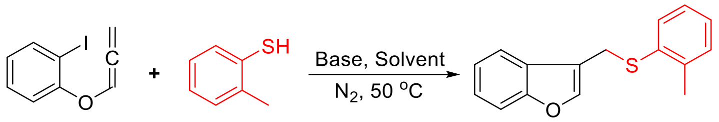

1a 4a 5a

Entry	1a (mmol)	4a (mmol)	Base	Solvent	Yield[b] (%)
**1**	0.10	0.15	LiHMDS (2.0 eq.)	DME	37
**2**	0.10	0.12	LiHMDS (2.0 eq.)	DME	44
**2**	0.10	0.10	LiHMDS (2.0 eq.)	DME	61
**3**	0.12	0.10	LiHMDS (2.4 eq.)	DME	82
**4**	0.15	0.10	LiHMDS (3.0 eq.)	DME	95 (94) [c]
**5**	0.20	0.10	LiHMDS (4.0 eq.)	DME	69
**6**	0.15	0.10	LiHMDS (2.0 eq.)	DME	98 (95) [c]
**7**	0.15	0.10	NaHMDS (3.0 eq.)	DME	9
**8**	0.15	0.10	KHMDS (3.0 eq.)	DME	trace
**9**	0.15	0.10	LDA (3.0 eq.)	DME	20
**10** [d]	0.15	0.10	LiHMDS (2.0 eq.)	DME	NR

[a] Unless otherwise specified, the reactions were carried using **1a**, **4a**, base, DME (2.0 mL) at 50 ℃ for 12 h under N_2. [b] Assay yields determined by ^{1}HNMR spectroscopy of the crude reaction mixtures using 1,3,5-Trimethoxybenzene as an internal standard. [c] Isolated yield of **5a**.[d] I is replaced by Br or Cl.

6. Condition optimization for the synthesis of 7a

Supplementary Table 4. Condition optimization for the synthesis of 7a[a]

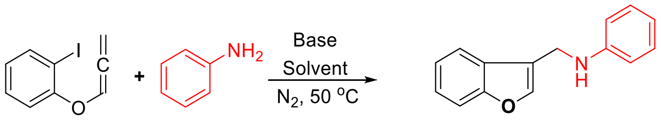

1a 6a 7a

Entry	1a (mmol)	6a (mmol)	Base	Solvent	Time (h)	Yield[b] (%)
**1**	0.10	0.11	LDA	DME	12	31
**2**	0.10	0.11	LiHMDS	DME	12	29
**3**	0.10	0.11	NaHMDS	DME	12	trace
**4**	0.10	0.11	KHMDS	DME	12	trace
**5**	0.10	0.11	LDA	THF	12	32
**6**	0.10	0.10	LDA	DME	12	36
**7**	0.10	0.15	LDA	DME	12	23
**8**	0.10	0.10	LDA (1.0 eq.)	DME	12	20
**9**	0.12	0.10	LDA (2.4 eq.)	DME	12	36
**10**	0.15	0.10	LDA (3.0 eq.)	DME	12	34
**11**	0.10	0.10	LDA	DME	24	48
**12**	0.10	0.10	LDA	DME	38	36
**13** [c]	0.10	0.10	LDA	DME	24	NR

[a] Unless otherwise specified, the reactions were carried using **1a** (0.10 mmol), **6a** (0.10 mmol), base (0.2 mmol), solvent (2.0 mL) at 50 ℃ under N_2. [b] Isolated yield of **7a.** [c] I is replaced by Br or Cl.

7. Reaction screening of hydroxyl groups

Supplementary Table 5. Reaction screening of hydroxyl groups[a]

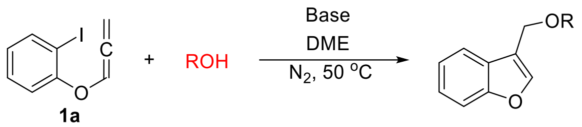

Entry	R	Base	Yield%
**1**	Ph	LDA	0
**2**	Ph	LiHMDS	0
**3**	Ph	NaHMDS	0
**4**	Ph	KHMDS	0
**5**	isobutyl	LDA	0

[a] Unless otherwise specified, the reactions were carried using **1a** (0.10 mmol), ROH (0.2 mmol), base (0.2 mmol), DME (2.0 mL) for 8 h under N_2.

8. General procedure for the preparation of 3

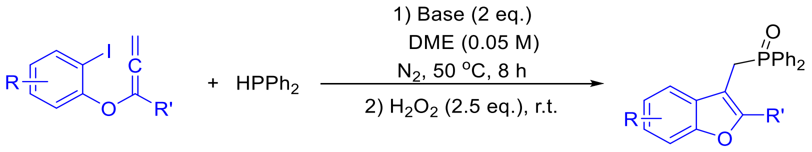

1 **3**

To a 10 mL anaerobic tube with a magnetic stir bar was added **1** (0.10 mmol) and $HPPh_2$ (0.20 mmol) with 0.20 mmol of LDA (or LiHMDS) and 2 mL DME at 50 ℃ for 8 h under N_2. H_2O_2 (0.25 mmol) was added into reaction system at 0 ℃, then warm the system to room temperature, and react for 1 h. After the trivalent phosphine was oxidized (monitored by TLC), water (4 mL) and ethyl acetate (2 mL) were then added and the organic layers separated, brine washing, dried (Na_2SO_4), evaporated under reduced pressure. The reaction mixture was subjected to column chromatography on silica gel (3:1 PE / EA) to afford the desired product **3**.

9. General procedure for the preparation of 5

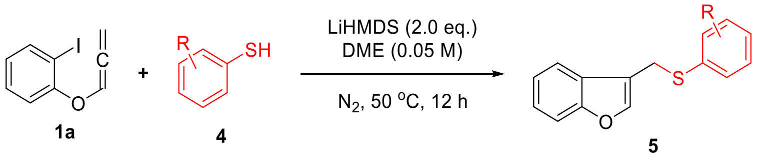

To a 10 mL anaerobic tube with a magnetic stir bar was added **1a** (0.15 mmol) and **4** (0.10 mmol) with 0.20 mmol of LiHMDS and 2 mL DME at 50 ℃ for 12 h under N_2. Water (4 mL) and ethyl acetate (2 mL) were then added and the organic layers separated, brine washing, dried (Na_2SO_4), evaporated under reduced pressure. The reaction mixture was subjected to column chromatography on silica gel (300:1 PE / EA) to afford the desired product **5**.

10. General procedure for the preparation of 7

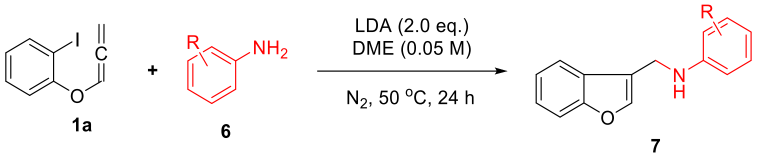

To a 10 mL anaerobic tube with a magnetic stir bar was added **1a** (0.10 mmol) and **6** (0.10 mmol) with 0.20 mmol of LDA and 2 mL DME at 50 ℃ for 24 h under N_2. Water (4 mL) and ethyl acetate (2 mL) were then added and the organic layers separated, brine washing, dried (Na_2SO_4), evaporated under reduced pressure. The reaction mixture was subjected to column chromatography on silica gel (300:1 PE / EA) to afford the desired product **7**.

11. General procedure for the preparation of 8

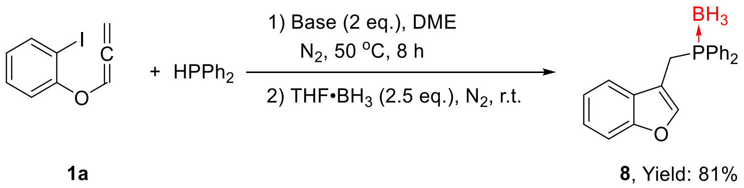

To a 100 mL Schleck vial with a magnetic stir bar was added **1a** (3.88 mmol) and $HPPh_2$ (7.75 mmol) with 7.75 mmol of LDA and 40 mL DME at 50 ℃ for 8 h under N_2. THF•BH_3 (9.70 mmol) was added into reaction system at 0 ℃ under N_2, then warm the system to room temperature, and react for 2 h. The reaction system was quenched by methanol. Water (10 mL) and ethyl acetate (10 mL) were then added and the organic layers separated, brine washing, dried (Na_2SO_4), evaporated under reduced pressure. The reaction mixture was subjected to column chromatography on silica gel (100:1 PE / EA) to afford the desired product **8** (1.04 g, 81%).

Mechanistic Considerations

1. Inhibition by Radical Scavengers

TEMPO was selected as the radical scavenger to block the intermolecular radical coupling processes (**Supplementary Table 6**).

Supplementary Table 6. Inhibition by radical scavengers

Target product	Yield[a] (%)	Yield[b] (%)
**3a**	41	17
**5a**	0	3
**7a**	0	0

a. The specific experimental operation methods refer to the general procedures for preparing **3**, **5**, and **7**, respectively, and add TEMPO (2.0 eq.). b. The reactions were carried using **1a**, PPh_2Li / PhSLi (*t*-BuOK)/ PhNHLi, TEMPO (2.0 eq.), solvent (2.0 mL) for 8 h under N_2.

2. Dark control Experiments

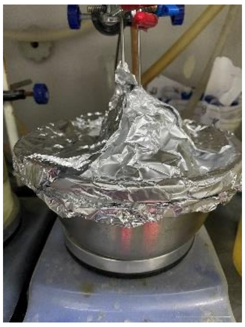

To a 10 mL anaerobic tube wrapped with aluminum foil was added **1a**, $HPPh_2$ / **4a** / **6a**, base, and 2 mL DME. The specific experimental operation methods refer to the general procedures of preparation **3**, **5** and **7**, respectively, and then the whole reaction apparatus was also wrapped with aluminum foil to ensure the reaction was proceed under dark conditions (**Supplementary Figure 3**).

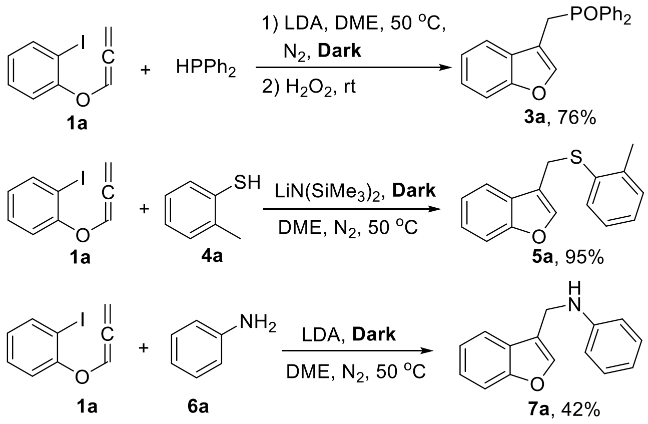

Supplementary Figure 3. Dark control experiments

3. Radical-Radical Coupled Disulfide

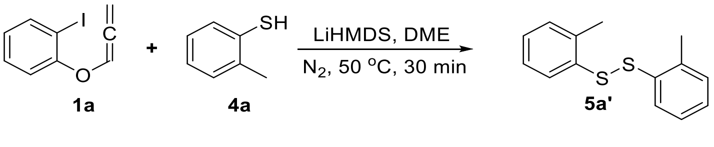

^{1}H NMR (400 MHz, $CDCl_3$) δ 7.61 – 7.45 (m, 2H), 7.20 – 7.01 (m, 6H), 2.43 (s, 6H).

^{13}C NMR (101 MHz, $CDCl_3$) δ 137.4, 135.4, 130.3, 128.7, 127.3, 126.7, 20.0.

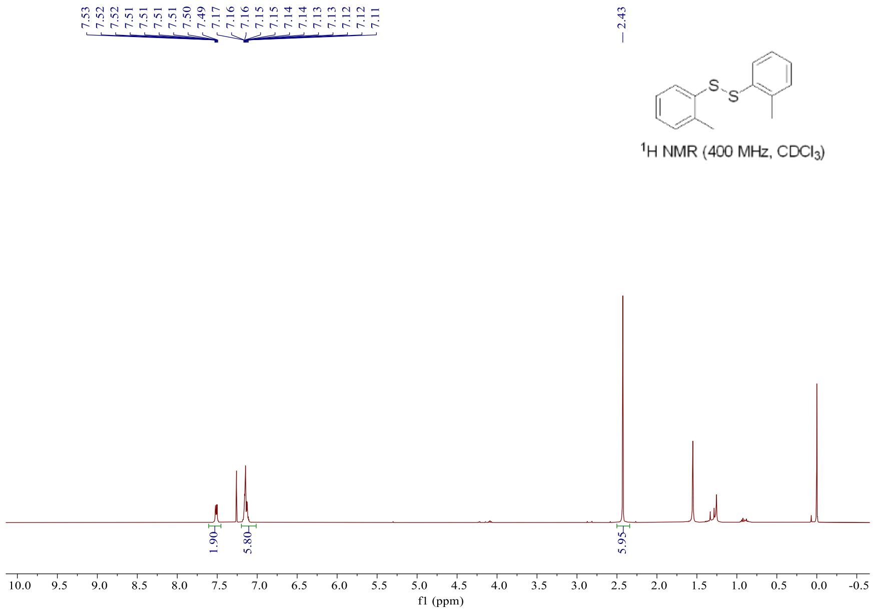

Supplementary Figure 4. ^{1}H NMR of **5a'**

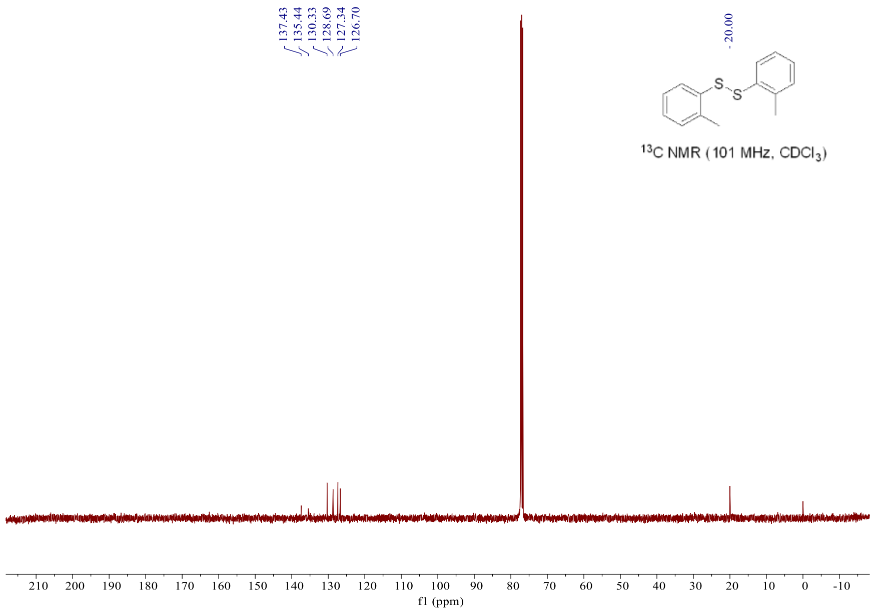

Supplementary Figure 5. ^{13}C NMR of **5a'**

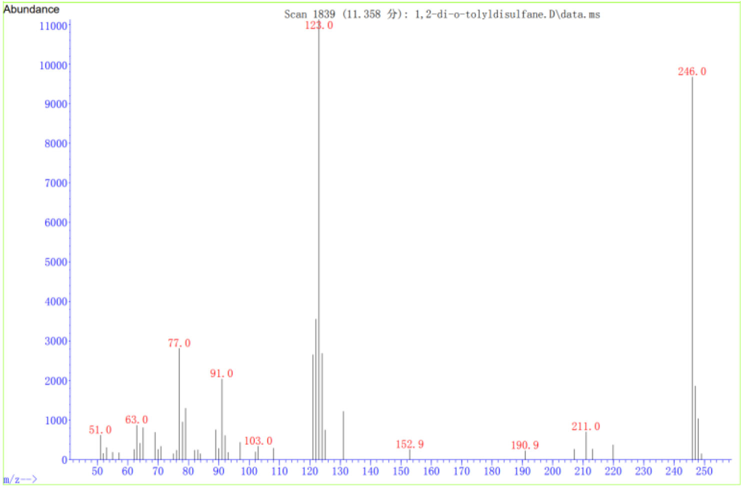

Supplementary Figure 6. GC-MS after 30 min of reaction

4. Density functional theory (DFT) calculation for radical reaction

For exploring the detail of the reaction mechanism more deeply, density functional theory (DFT) calculations performed with Gaussian 16 B.01. Geometry optimizations were performed using the M06-2X functional with the dispersion correction D3. Multiple basis sets 6-31g(d,p) / Lanl2dz employed for the geometry optimization: 6-31g(d,p) for elements C, H, O, Li, and P; Lanl2dz for I. The solvent module IEF-PCM (DME, eps = 7.189, epsinf = 1.972) was employed for simulating solvent condition. Minima and transition structures on the potential energy surface were confirmed using frequency analysis at the same level of theory, showing respectively zero and only one imaginary frequency. Gibbs energies were evaluated at the reaction temperature of 50 ^{o}C (323.15 K). Single point corrections were through the high-level basis sets 6-311g++(2d,2p) / Lanl08.

DFT calculation of the radical reaction between $HPPh_2$ and 1a

The proposed mechanism is including two possible pathways: triplet state pathway and singlet state pathway (**Supplementary Figure 7**). For the triplet state pathway, there are three steps: (1) cations exchange between $HPPh_2$ and LDA; (2) electron transfer from phosphor to benzene ring; and (3) intersystem crossing and quenching. For the singlet state pathway, there are also three steps: (1) cations exchange between $HPPh_2$ and LDA; (2) electrophilic substitution; and (3) nucleophilic substitution and quenching.

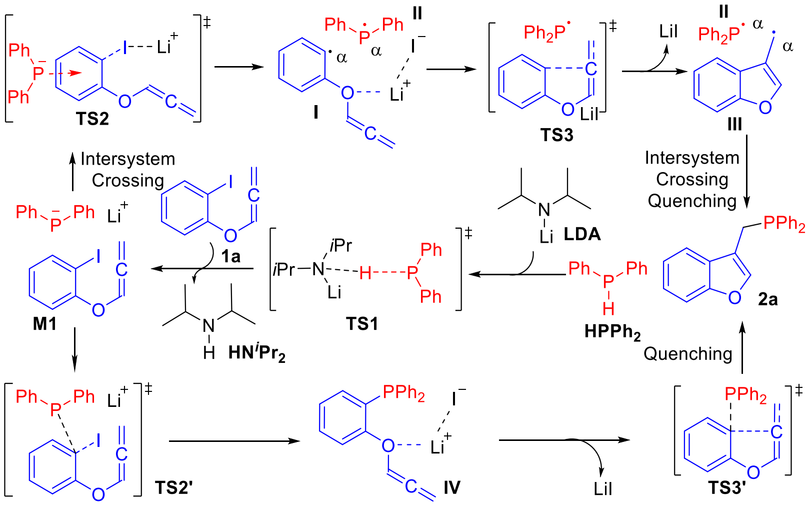

Supplementary Figure 7. Proposed reaction mechanism

The triplet state pathway vs the singlet state pathway

For finding the most favorite pathway of the reaction out, we compared the Gibbs free energy profile of the triplet state pathway and the singlet state pathway. As shown in **Supplementary Figure 8**, at the second step of these two pathways, the intermediate **M1** generates following intermediates via transition states **TS2** or **TS2'**. The Gibbs energy barrier of the singlet state pathway is 20.09 kcal / mol, and of the triplet state pathway is 14.44 kcal / mol. Furthermore, the process from **IV** to **TS3'** in the singlet pathway has a quite high energy barrier (36.52 kcal/mol). The triplet pathway occupies larger advantage under the condition of reaction kinetics. Therefore, the triplet state pathway is the most favorite choice of the reaction.

Coordinates of the optimized structures are provided as source data. A source data file is present.

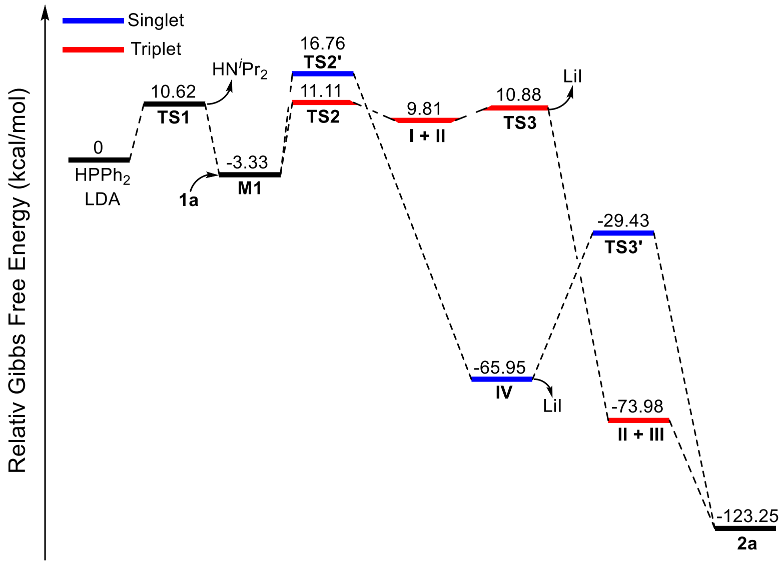

Supplementary Figure 8. Gibbs free energy profile of the two pathways

5. Failed trials in the synthesis of reaction substrates for radical clock experiment

Unfortunately, none of these experiments provided the desired compound (**Supplementary Figure 9**). The isomerization of the alkyne molecule **s1** gave the phenol **s2** or the de-halogenated product **s3** in moderate to good yields without formation of the target allene product **s4**. The base-promoted isomerization of the alkyne **s5** provided the conjugated triene product **s6** in a good yield, with no formation of the target allene product **s7** observed.

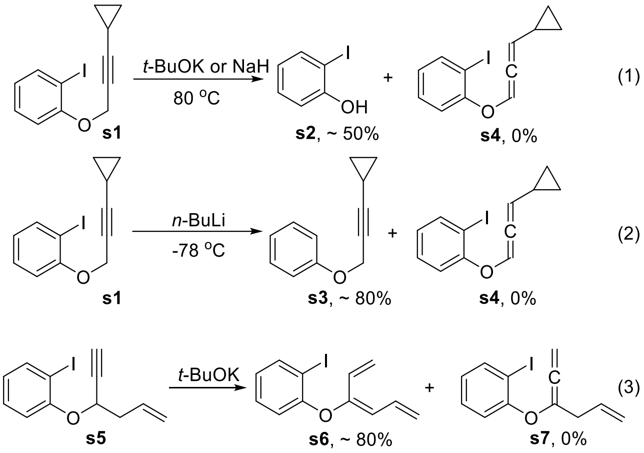

Supplementary Figure 9. Failed Experiments on the Substrate Synthesis for Radical Clock Experiment

6. Electron Paramagnetic Resonance (EPR) Experiment

Electron paramagnetic resonance spectroscopy spectra were measured on Chinainstru&Quantumtech (Hefei) EPR200-Plus with continues-wave X band frequency (**Supplementary Figure 10**). In each case the modulation amplitude = 1 G, sweep time = 10 s, microwave power = 0.0002 mW (corresponding to attenuation = 60 dB, which was sufficiently high to avoid power saturation at T = 298 K), time constant = 0.3 s and repeat number = 3. EPR studies were carried out by using reaction mixtures after stirring for 10~20 min.

(**Green** line) The X-band EPR spectrum of 1:1 stoichiometric reaction of $HPPh_2$ (0.2mmol) and LDA (0.2mmol) was measured at 298 K with DME (2 mL) as solvent at a microwave frequency of 9.418043137 GHz. (Cyan line) The X-band EPR spectrum of 1:2 stoichiometric reaction of **1a** (0.1mmol) and $HPPh_2$ (0.2mmol) was measured at 298 K with DME (2 mL) as solvent at a microwave frequency of 9.418989182 GHz. (**Black** line) The X-band EPR spectrum of 1:2 stoichiometric reaction of **1a** (0.1mmol) and LDA (0.2mmol) was measured at 298 K with DME (2

mL) as solvent at a microwave frequency of 9.417981148 GHz. (**Red** line) The X-band EPR spectrum of 1:2:2 stoichiometric reaction of **1a** (0.1mmol), $HPPh_2$ (0.2 mmol) and LDA (0.2mmol) was measured at 298 K with DME (2 mL) as solvent at a microwave frequency of 9.418333054 GHz (g = 2.0023).

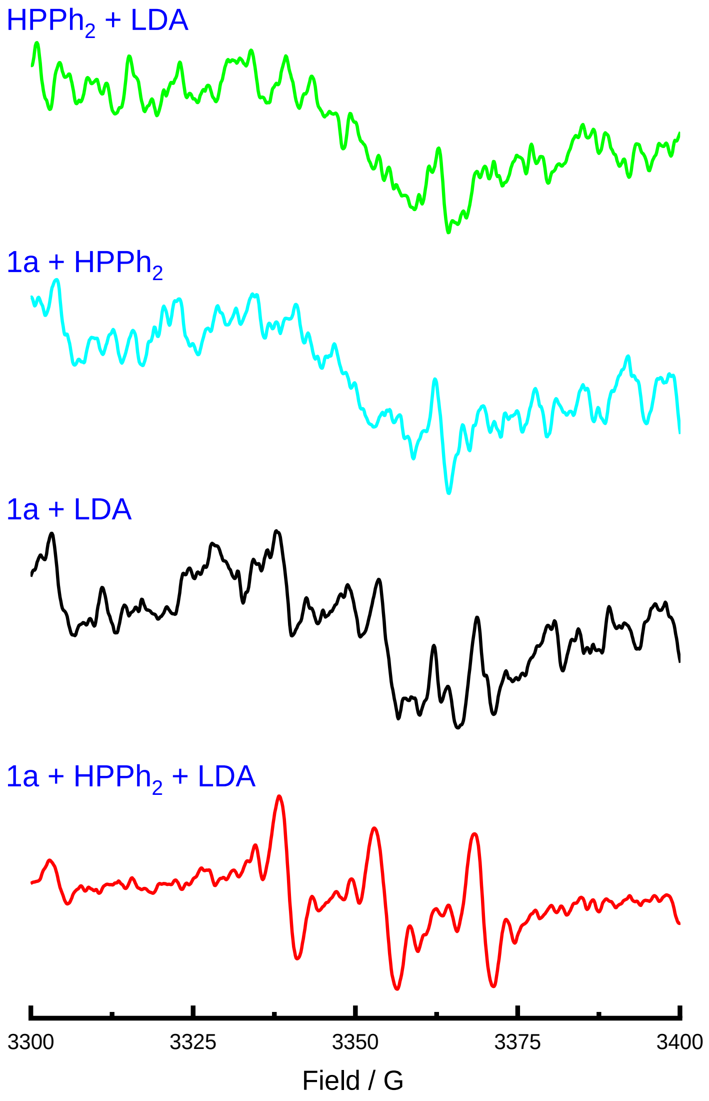

Supplementary Figure 10. EPR spectrums of the reaction mixtures

7. Control Experiments

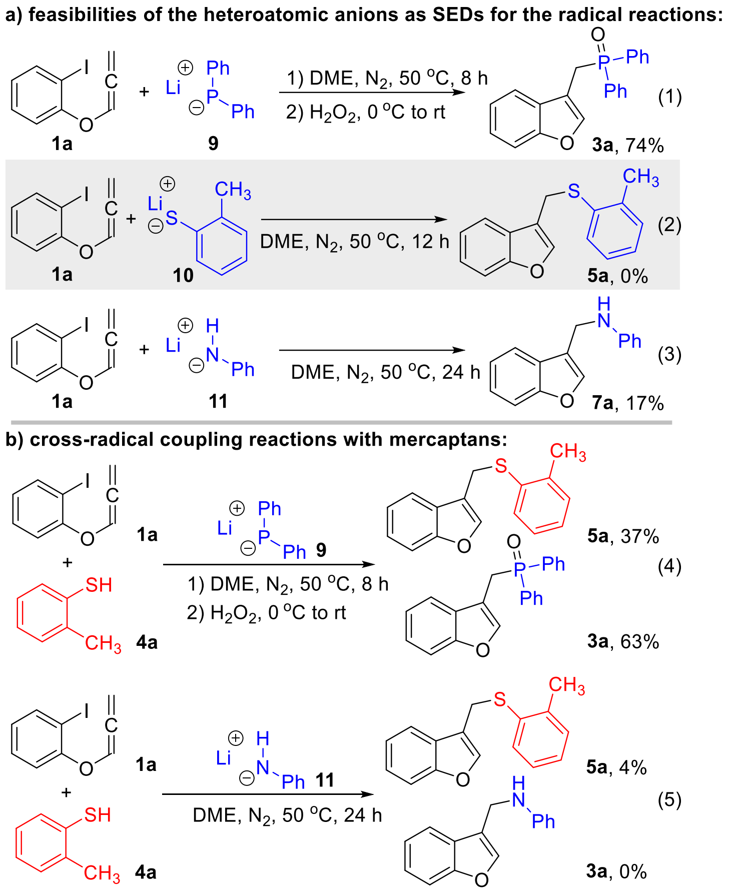

Supplementary Figure 11. Control experiments

General procedure for the control experiments

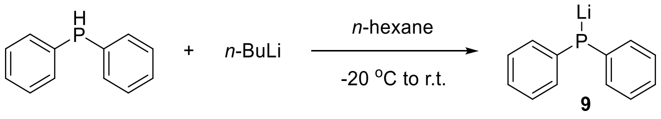

According to the methods reported in the literature,[3] *n*-BuLi (2.5 M in dry THF, 1.07 mL, 2.69 mmol) was added to a solution of $HPPh_2$ (2.95 mmol, 550 mg) in dry *n*-hexane (20 mL) at -20 °C under N_2. The reaction mixture was allowed to slowly warm to room temperature over 3 h, then put it in a glove box filled with nitrogen, the solid was isolated and washed with *n*-hexane (10 mL) to afford **9** (515.83 mg, >99 %) as a yellow solid.

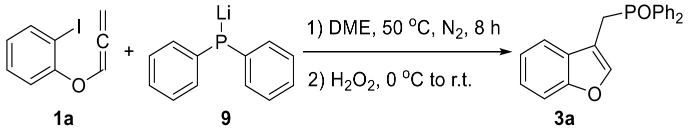

To a 10 mL anaerobic tube with a magnetic stir bar was added **1a** (0.10 mmol) and **9** (1 M in dry THF, 200 μL, 0.2 mmol) and 2 mL DME at 50 ℃ for 8 h under N_2. H_2O_2 (0.25 mmol) was added into reaction system at 0 ℃, then warm the system to room temperature, and react for 1 h. The reaction

mixture was subjected to column chromatography on silica gel (3:1 PE / EA) to afford the desired product **3a** (24.6 mg, 74%).

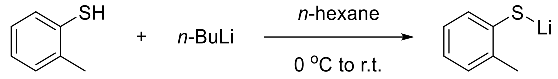

4a **10**

According to the methods reported in the literature,[4] *n*-BuLi (2.5 M in dry THF, 2.9 mL, 7.32 mmol) was added to a solution of **4a** (8.05 mmol, 1 g) in dry *n*-hexane (10 mL) at 0 °C under N_2. The reaction mixture was allowed to slowly warm to room temperature over 5 min, then put it in a glove box filled with nitrogen, the solid was isolated and washed with *n*-hexane (20 mL) to afford **10** (893.5 mg, 94 %) as a white solid.

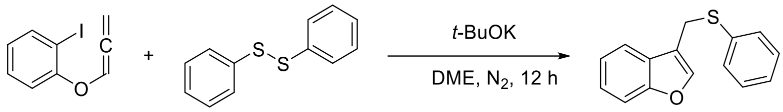

1a **S12**

To a 10 mL anaerobic tube with a magnetic stir bar was added **1a** (0.30 mmol) and diphenyl disulfide (0.1 mmol) with 0.20 mmol of *t*-BuOK and 2 mL DME at 50 ℃ for 12 h under N_2. The reaction mixture was subjected to column chromatography on silica gel (300:1 PE / EA) to afford the desired product **S12** (2.6 mg, 11%).

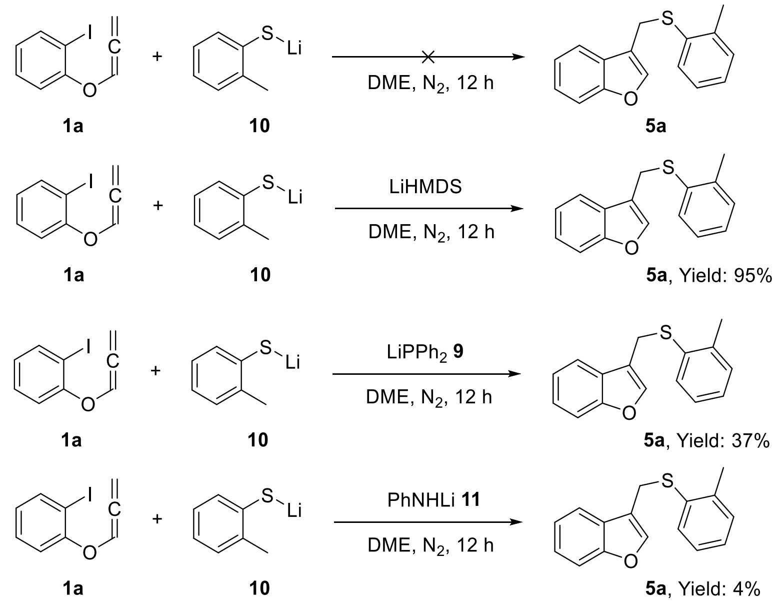

Supplementary Figure 12. Cross-radical coupling reactions with mercaptans

To a 10 mL anaerobic tube with a magnetic stir bar was added **1a** (0.30 mmol) and **10** (0.1 mmol) with LiHMDS / **9** / **11** (0.20 mmol) and 2 mL DME at 50 ℃ for 12 h under N_2. The reaction mixture was subjected to column chromatography on silica gel (300:1 PE / EA) to afford the desired product

5a (**Supplementary Figure 12**).

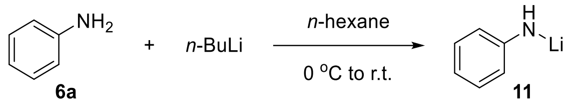

According to the literature's reported methods,[5] *n*-BuLi (2.5 M in dry THF, 730 μL, 1.82 mmol) was added to a solution of **6a** (2.0 mmol, 186.26 mg) in dry *n*-hexane (5 mL) at 0 °C under N_2. The reaction mixture was allowed to slowly warm to room temperature over 30 min, then put it in a glove box filled with nitrogen, the solid was isolated and washed with *n*-hexane (10 mL) to afford (5 mL) to afford **11** (168.40 mg, 93 %) as a gray solid.

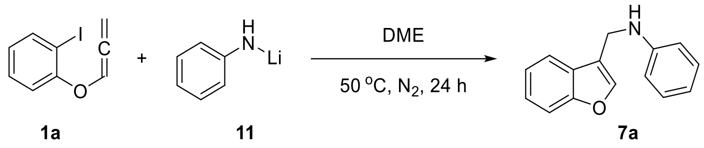

To a 10 mL anaerobic tube with a magnetic stir bar was added **1a** (0.10 mmol) and **11** (0.5 M in dry THF, 200 μL, 0.1 mmol) and 2 mL DME at 50 ℃ for 24 h under N_2. The reaction mixture was subjected to column chromatography on silica gel (300:1 PE / EA) to afford the desired product **7a** (3.9 mg, 17%).

8. Cyclic voltammetry

General procedure. Voltammetry experiments (CV, DPV) were performed using a CH Instruments 600E Electrochemical Analyzer/Workstation and the data were processed using CHI software v9.24. All experiments were performed under an inert atmosphere (nitrogen), in a glove box at room temperature using electrochemical cells that consisted of a 10 mL vial, Pt electrode working electrode, Calomel (KCl saturated) reference electrode, Pt wire auxiliary electrode.

For each session of measurements, fresh electrolyte was prepared in degassed DMF / DMSO (8 mL) using TBAHFP as the supporting salt (0.1 M). For calibration, the redox potential of ferrocene was measured at the beginning and at the end of every session, providing the average value used for determining $E_{1/2}$ vs. Fc/Fc+ (published potential in similar conditions: 0.45 V vs. SCE[6]. Cyclic voltammetry was measured at a scan rate of 50 mV per second.

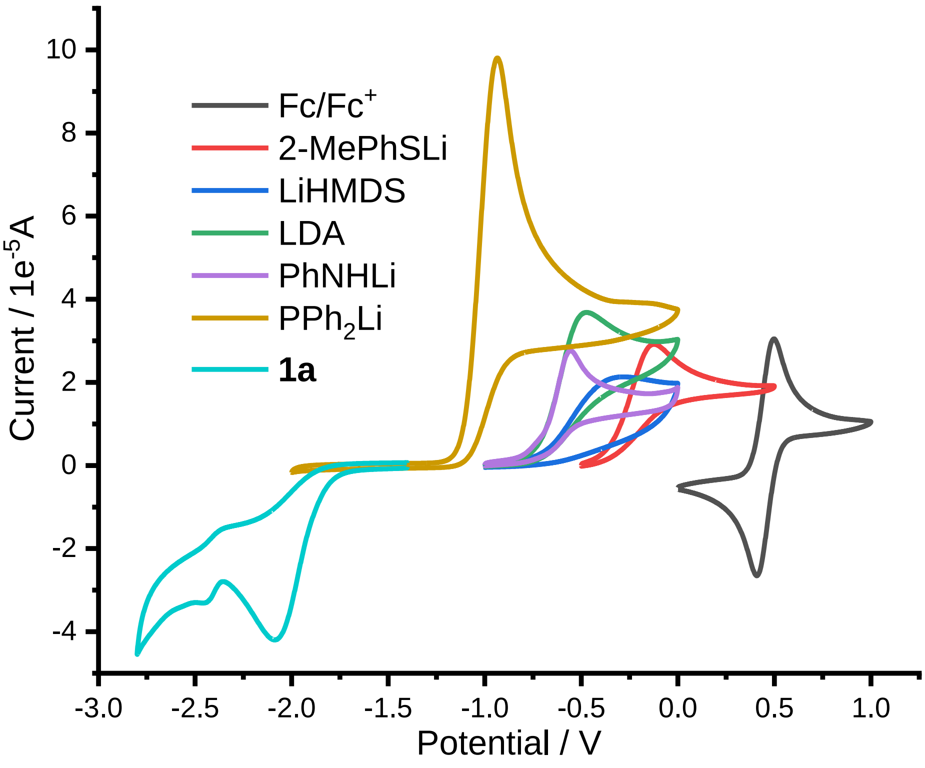

Supplementary Figure 13. Cyclic voltammogram

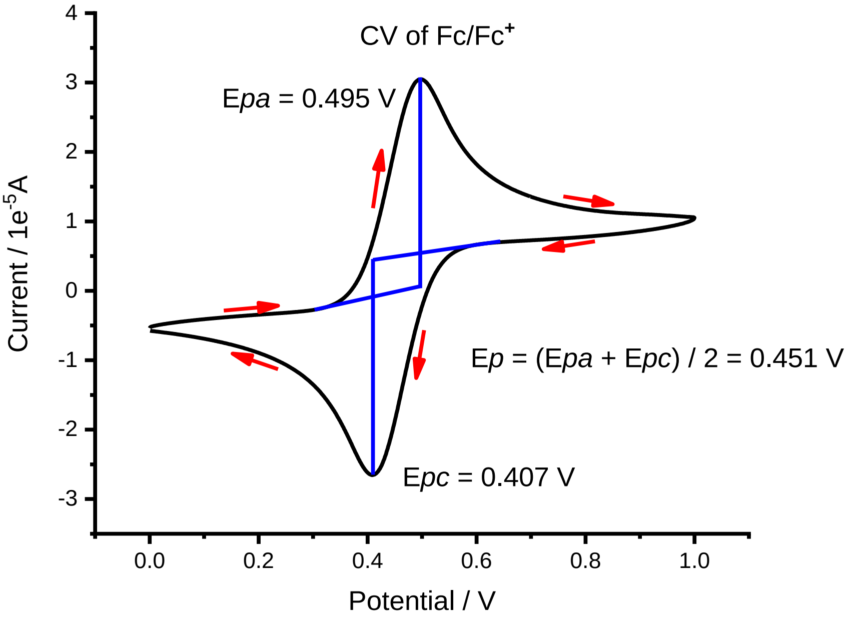

Supplementary Figure 14. Cyclic voltammogram of Fc/Fc^+ [2 mM]. E*p*: 0.45 V vs. SCE (DMF)

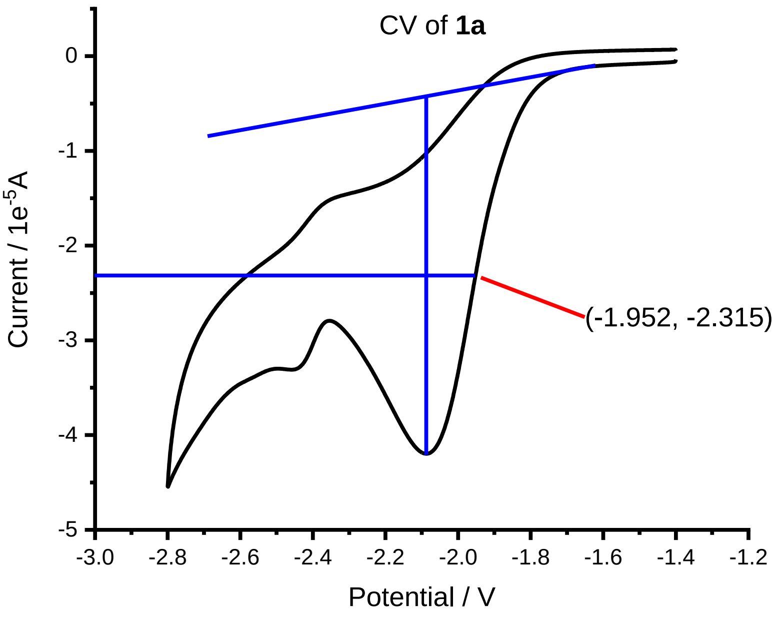

Supplementary Figure 15. Cyclic voltammogram of **1a** [2 mM]. $E^1_{1/2}$: -1.95 V vs. SCE (DMF).

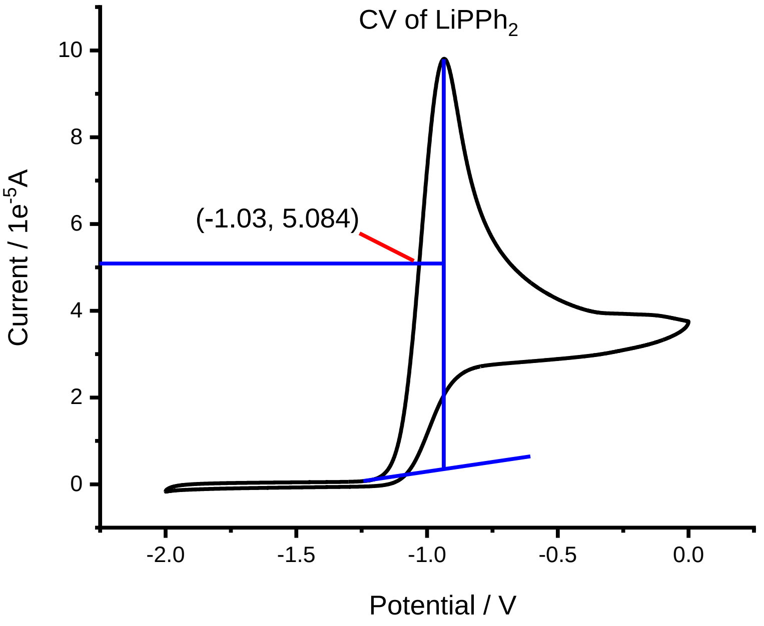

Supplementary Figure 16. Cyclic voltammogram of $LiPPh_2$ [0.02 M]. $E_{1/2}$: -1.03 V vs. SCE (DMF).

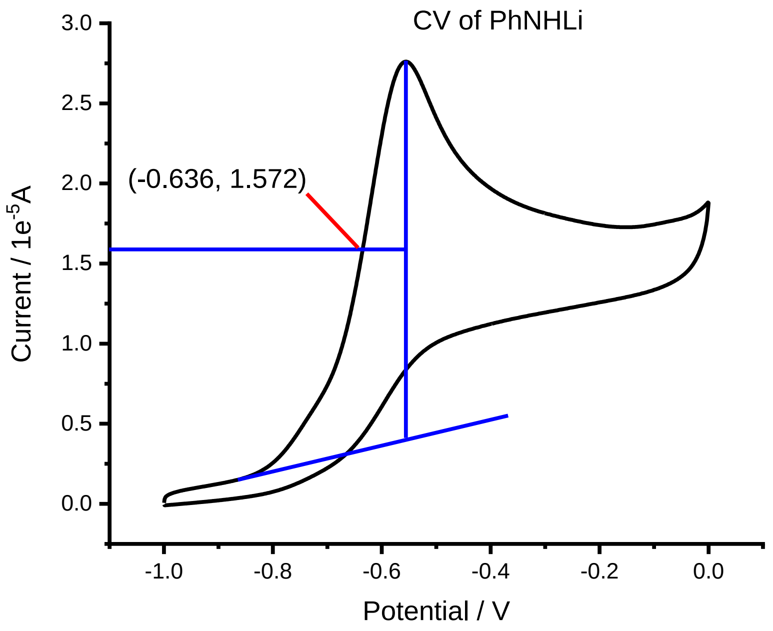

Supplementary Figure 17. Cyclic voltammogram of PhNHLi [0.02 M]. $E_{1/2}$: -0.64 V vs. SCE (DMSO)

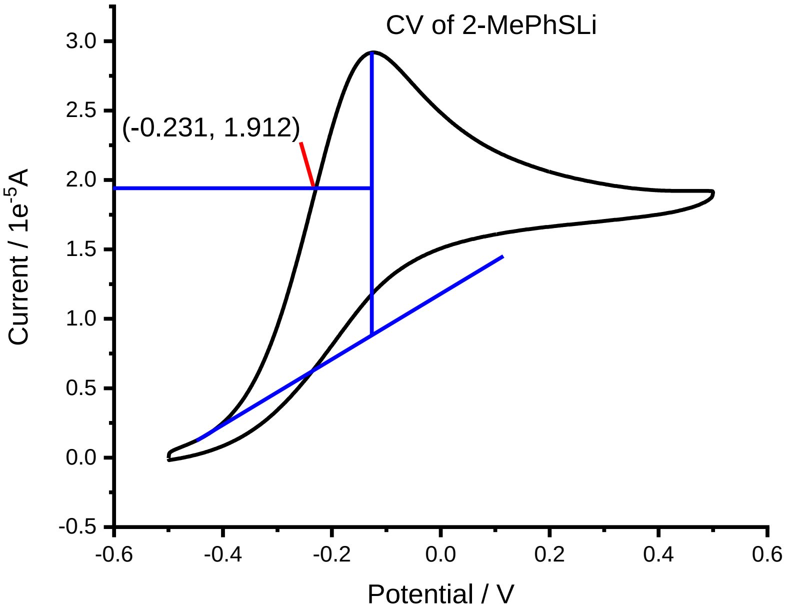

Supplementary Figure 18. Cyclic voltammogram of 2-MePhSLi [0.02 M]. $E_{1/2}$: -0.23 V vs. SCE (DMF)

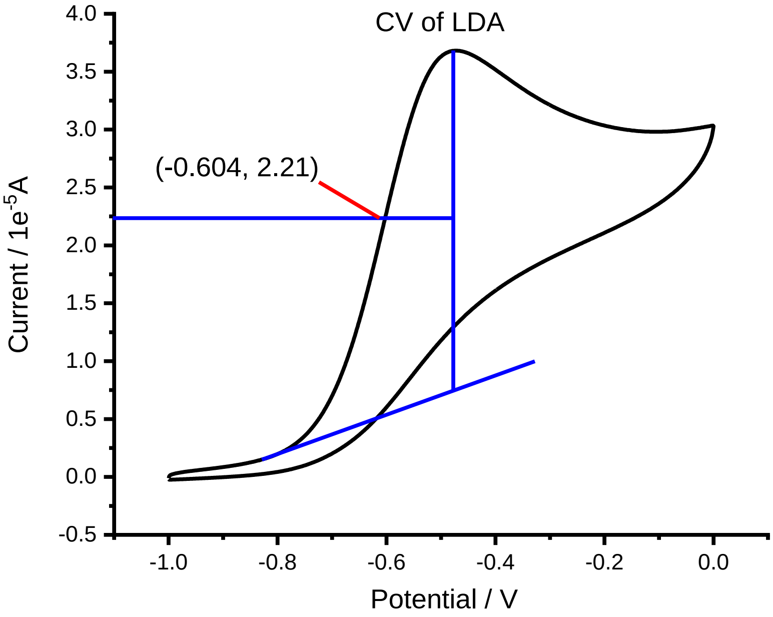

Supplementary Figure 19. Cyclic voltammogram of LDA [0.02 M]. $E_{1/2}$: -0.60 V vs. SCE (DMSO).

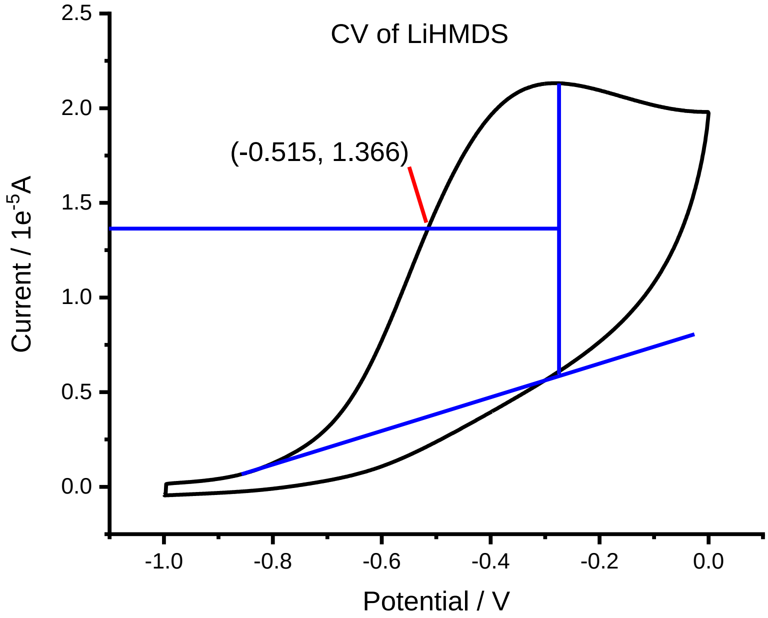

Supplementary Figure 20. Cyclic voltammogram of LiHMDS [0.02 M]. $E_{1/2}$: -0.52 V vs. SCE (DMSO).

Synthetic Transformations

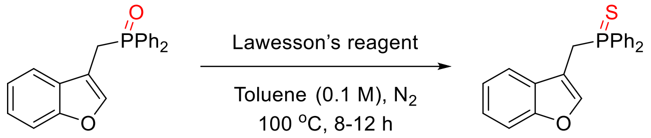

3a **13**, Yield: 99%

To a 10 mL anaerobic tube with a magnetic stir bar was added **3a** (0.10 mmol) and Lawesson's reagent (0.7 eq.) with 1 mL Toluene at 100 ℃ for 8 – 12 h under N_2. After cooling to room temperature, the reaction system was quenched by saturated sodium carbonate. The organic layers were then passed through a short $MgSO_4$ / diatomite / $MgSO_4$ column and eluted with EA, evaporated under reduced pressure. The reaction mixture was purified by silica gel chromatography (100:1 PE / EA as eluent) to give the desired product **13** (34.5 mg, 99%) as a white solid.

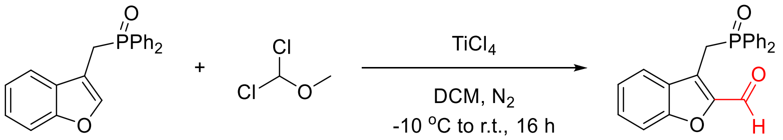

3a **14**, Yield: 60%

A solution of **3a** (500 mg) in dry DCM (30 mL) was cooled at −10 °C under an argon atmosphere. $TiCl_4$ (1M in DCM, 3.5 eq.) was added, followed by 1,1-dichlorodimethyl ether (3.5 eq.). The resulting mixture was allowed to warm up to rt and stirred for 16 h. The solution was then poured onto ice and stirred for 10 min. The organic phase was separated and the aqueous phase extracted with DCM (2 x 50 mL). The combined organic layers were washed with a saturated aqueous solution of $NaHCO_3$ (2 x 30 mL), H_2O (2 x 30 mL), and brine (2 x 30 mL), then dried over $MgSO_4$, filtered and concentrated under reduced pressure. The crude product was purified via silica gel column chromatography (8:1 PE / EA as eluent) to afford compound **14** (325.3 mg, 60% yield) as an amorphous white solid.

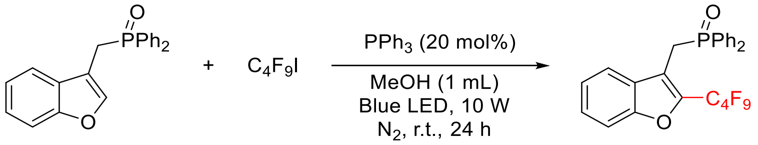

3a **15**, Yield: 74%

To a 4.0 mL vial equipped with a magnetic stir bar was added **3a** (0.10 mmol) and PPh_3 (20 mol%), put it in a glove box filled with nitrogen to replace the nitrogen, and then C_4F_9I (0.20 mmol) were added. then added CH_3OH (1.00 mL) via syringe, Outside the glovebox the reaction vial was transferred into the photoreactor (440 – 445 nm,10 W), the irradiation was started and the reaction mixture was allowed to stirred for 24 h at room temperature. After completion of the reaction, monitored by TLC plate, the mixture was concentrated under reduced pressure. The resulting crude residue was purified via column chromatography on silica gel (petroleum ether / ethyl acetate = 10:1) to afford the desired products **15** (40.7 mg, 74% yield) as a white solid.

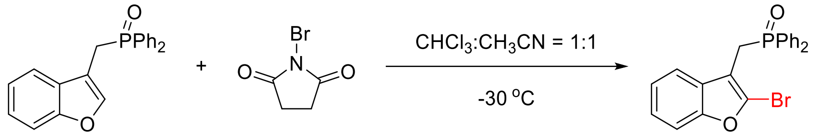

3a **16**, Yield: 95%

To a solution of **3a** (1.0 g, 3.01 mmol) in chloroform (20 mL) was added dropwise *N*-bromosuccinimide (0.49 g, 2.74 mmol) in CH_3CN (20 mL) at −30 ℃. The reaction mixture was stirred at the same temperature for 1 h. The reaction mixture was poured into water and extracted with chloroform. The organic layers were washed with brine and dried. The solvent was evaporated off, giving a residue which was purified by silica gel column chromatography (1:3 PE / EA) to give **16** (1.1 g, 95%) as a white solid.

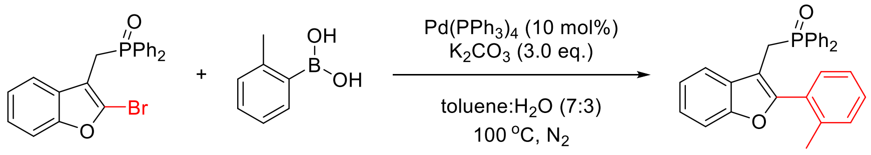

16 **17**, Yield: 62%

To a 10 mL anaerobic tube with a magnetic stir bar was added **16** (41.12 mg, 0.1 mmol), *o*-tolylboronic acid (27.19 mg, 0.2 mmol), K_2CO_3 (41.46 mg, 0.3 mmol), $Pd(Ph_3P)_4$ (3.66 mg, 10 mol%), toluene (1.4 mL) and H_2O (0.6 mL) at 100 ℃ for 8 h under N_2. The reaction mixture was filtered through celite pad and washed by EA, and the filtrate was concentrated. The residue was dissolved in EA (2 mL) and washed successively with water (5 mL) and saturated brine (5 mL). The organic phase was dried over Na_2SO_4 and concentrated under reduced pressure. The residue was purified by flash column chromatography (1:3 PE / EA) to afford **17** as a white solid (26.2 mg, 62%).

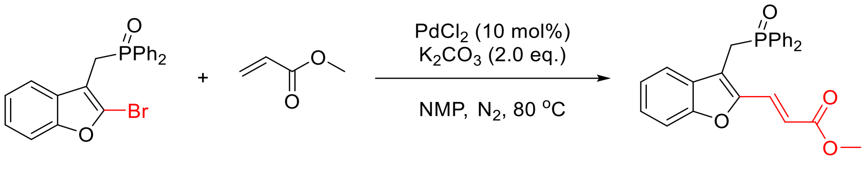

16 **18**, Yield: 63%

To a 10 mL anaerobic tube with a magnetic stir bar was added **16** (41.12 mg, 0.1 mmol), methyl acrylate (18 μL, 0.2 mmol), K_2CO_3 (20.16 mg, 0.2 mmol), $PdCl_2$ (1.29 mg, 10 mol%) and NMP (2 mL) at 80 ℃ for 12 h under N_2. The reaction mixture was filtered through celite pad and washed by EA, and the filtrate was concentrated. The residue was dissolved in EA (2 mL) and washed successively with water (5 mL) and saturated brine (5 mL). The organic phase was dried over Na_2SO_4 and concentrated under reduced pressure. The residue was purified by flash column chromatography (1:3 PE / EA) to afford **18** as a white solid (26.2 mg, 63%).

Antibacterial Activity in *Vitro*

Antibacterial activities of the title compounds **3**, **5**, **7**, **8**, **13** – **18** against *Xanthomonas oryzae* pv. *oryzae* (*Xoo*) and *Xanthomonas axonopodis* pv. *Citri* (*Xac*) were evaluated by using the turbidimeter test, commercial agricultural antibacterial thiodiazole-copper and bismerthiazol was used as control. The test compounds were dissolved in 80 μL of DMSO and diluted with 0.1% (V / V) Tween-20 to prepare two concentrations of 50 and 25 μg/mL. 1 mL of the liquid sample was added to the non-toxic nutrient broth (NB: 1.5 g of beef extract, 2.5 g of peptone, 0.5 g of yeast powder, 5.0 g of glucose and 500 mL of distilled water, pH = 7.0 – 7.2) liquid medium in 4 mL tubes. Then, 40 μL of NB containing *Xoo and Xac* was added to 5 mL of solvent NB containing the test compounds, thiodiazole-copper and bismerthiazol. The inoculated test tubes were incubated at (30 ± 1) °C under continuous shaking at 180 rpm / min for 38 (*Xac*) or 48 h (*Xoo*). The culture growth was monitored spectrophotometric ally by measuring the optical density at 595 nm (OD_{595}) and expressed as corrected turbidity. The relative inhibitory rate (%) compared with a blank assay was calculated according to the following formula:

$$\text{Inhibition rate (\%)} = (CK\text{-}T) / CK \times 100$$

“CK” means the value of corrected optical density of bacterial growth on untreated NB (negative control), and “T” means the value of corrected optical density of bacterial growth on treated NB. Similarly, the solvent of *Xoo and Xac* was SM (10.0 g of peptone, 5.0 g of glucose, 1.0 g of casein acid hydrolysate, 1000 mL of distilled water, pH = 7.0 – 7.2).

Based on the previous bioactivity, the antibacterial activities (expressed by EC_{50}) of the target compounds against *Xoo* and *Xac* were evaluated and calculated by using SPSS 17.0 software. Different small letters indicate the values of control efficiency with significant differences among different treatment groups at $P < 0.05$. Each experiment was repeated thrice. All the compounds tested possessed greater than 95% purity.

Supplementary Table 7. ***In vitro*** **antibacterial activity of 3, 8, 13-18**

Compounds	Inhibition Rate[a]/%			
	Xoo		*Xac*	
	50 μg/mL	25 μg/mL	50 μg/mL	25 μg/mL
**3a**	0	0	66.22 ± 0.31	25.21 ± 0.32
**3b**	32.02 ± 0.63	26.92 ± 1.05	18.24 ± 0.81	13.20 ± 0.68
**3c**	16.44 ± 1.93	12.08 ± 0.97	32.99 ± 2.43	21.41 ± 0.88
**3d**	18.62 ± 0.51	11.35 ± 0.71	42.61 ± 0.37	28.32 ± 1.05
**3e**	0	0	51.34 ± 0.94	42.31 ± 1.34
**3f**	12.62 ± 1.07	11.04 ± 2.03	33.38 ± 1.92	29.72 ± 1.86
**3g**	28.38 ± 0.87	0	41.21 ± 0.28	31.01 ± 2.19
**3h**	19.39 ± 1.13	13.24 ± 3.03	0	0
**3i**	0	0	14.50 ± 2.05	10.99 ± 0.43
**3j**	0	0	9.64 ± 1.28	7.29 ± 0.61
**3k**	18.92 ± 1.92	10.96 ± 1.90	19.65 ± 1.47	7.05 ± 1.03
**3l**	24.66 ± 0.70	16.72 ± 2.18	4.79 ± 1.38	2.98 ± 0.95
**3m**	20.92 ± 1.81	12.23 ± 2.36	17.32 ± 1.03	8.09 ± 0.47
**8**	28.91 ± 1.02	19.51 ± 1.09	0	0
**13**	28.38 ± 1.07	14.18 ± 0.54	0	0
**14**	22.86 ± 1.67	20.89 ± 1.82	15.33 ± 1.57	9.53 ± 1.48
**15**	19.91 ± 0.99	9.09 ± 1.01	19.15 ± 0.50	11.19 ± 0.81
**16**	28.22 ± 1.37	19.98 ± 1.73	9.74 ± 1.04	4.79 ± 0.48
**17**	23.50 ± 3.22	19.82 ± 1.21	45.34 ± 2.07	4.20 ± 0.92
**18**	5.88 ± 0.66	0	0	0
**BT**[b]	62.14 ± 1.27	50.77 ± 0.69	45.36 ± 1.37	25.07 ± 0.64
**TC**[c]	65.27 ± 1.25	48.11 ± 0.78	47.82 ± 0.26	19.59 ± 0.29

[a] The experiments were repeated three times.

[b]BT = Bismerthiazol.

[c]TC = Thiodiazole Copper.

Supplementary Table 8. ***In vitro*** **antibacterial activity of 5**

Compounds	Inhibition Rate[a]/%			
	Xoo		*Xac*	
	50 μg/mL	25 μg/mL	50 μg/mL	25 μg/mL
**5a**	23.08 ± 0.55	20.13 ± 0.41	67.35 ± 0.66	57.18 ± 0.59
**5b**	44.94 ± 0.86	39.31 ± 0.81	27.17 ± 0.79	18.09 ± 0.51
**5c**	31.44 ± 2.47	0	70.57 ± 2.15	68.56 ± 0.78
**5d**	32.31 ± 1.62	0	68.05 ± 2.79	65.79 ± 1.58
**5e**	55.55 ± 1.20	49.87 ± 0.61	31.69 ± 0.98	23.93 ± 1.40
**5f**	77.52 ± 0.40	58.69 ± 0.32	69.69±0.76	59.54±0.85
**5g**	0	0	44.05 ± 0.27	34.72 ± 0.52
**5h**	22.80 ± 1.66	16.32 ± 0.38	69.28 ± 1.51	66.88 ± 1.25
**5i**	62.74 ± 1.84	59.08 ± 0.70	0	0
**5j**	56.13 ± 0.50	55.57 ± 0.65	66.41 ± 1.37	57.15 ± 2.90
**5k**	59.04 ± 0.47	52.79 ± 1.12	55.57 ± 0.46	45.80 ± 0.35
**5l**	0	0	38.19 ± 0.48	25.71 ± 1.02
**5m**	97.53 ± 0.12	84.34 ± 0.62	86.32 ± 1.10	76.93 ± 1.35
**5n**	38.28 ± 1.50	26.13 ± 1.91	52.33 ± 1.09	21.73 ± 1.07
**5o**	81.63 ± 0.83	75.52 ± 0.50	78.51 ± 0.21	73.09 ± 0.15
**5p**	34.74 ± 0.22	23.28 ± 1.02	64.36 ± 1.99	57.05 ± 1.70
**5q**	0	0	59.70 ± 0.93	51.03 ± 0.49
**5r**	52.19 ± 1.31	46.01 ± 1.76	31.12 ± 0.20	27.96 ± 0.53
**5s**	91.70 ± 0.32	81.54 ± 1.04	81.09 ± 0.44	71.45 ± 0.50
**5t**	0	0	73.80 ± 0.70	70.25 ± 1.17
**5u**	0	0	74.58 ± 1.08	64.36 ± 1.26
**5v**	78.00 ± 1.17	66.85 ± 1.15	81.54 ± 0.98	77.88 ± 0.46
**5w**	81.40 ± 1.11	75.20 ± 0.51	76.68 ± 0.55	70.76 ± 0.91
**5x**	9.17 ± 0.73	6.02 ± 1.01	11.51 ± 1.04	3.58 ± 0.24
**5y**	16.13 ± 0.27	10.55 ± 0.32	9.83 ± 1.77	8.21 ± 1.27
**BT**[b]	62.14 ± 1.27	50.77 ± 0.69	45.36 ± 1.37	25.07 ± 0.64
**TC**[c]	65.27 ± 1.25	48.11 ± 0.78	47.82 ± 0.26	19.59 ± 0.29

[a] The experiments were repeated three times.

[b]BT = Bismerthiazol.

[c]TC = Thiodiazole Copper.

Supplementary Table 9. *In vitro* antibacterial activity of 7

Compounds	Inhibition Rate[a]/%			
	Xoo		*Xac*	
	50 μg/mL	25 μg/mL	50 μg/mL	25 μg/mL
**7a**	11.27 ± 0.85	8.78 ± 1.09	66.49 ± 2.62	58.41 ± 1.85
**7b**	58.33 ± 0.89	28.77 ± 1.91	45.25 ± 0.72	33.79 ± 0.39
**7c**	24.08 ± 1.18	16.02 ± 0.70	60.74 ± 0.94	59.25 ± 1.36
**7d**	81.77 ± 2.61	77.16 ± 0.97	79.58 ± 0.30	73.22 ± 1.42
**7e**	70.54 ± 0.75	48.72 ± 0.78	76.43 ± 1.10	67.29 ± 1.80
**7f**	1.77 ± 0.63	0	48.38 ± 1.03	37.24 ± 0.39
**7g**	48.01 ± 1.39	21.67 ± 0.95	21.32 ± 1.82	10.66 ± 0.99
**7h**	66.95 ± 0.21	53.47 ± 0.29	63.20 ± 0.99	40.26 ± 0.52
**7i**	66.43 ± 1.08	47.88 ± 1.72	74.73 ± 0.91	64.90 ± 2.10
**7j**	84.52 ± 0.25	67.55 ± 0.82	53.93 ± 1.17	45.49 ± 0.38
**7k**	29.85 ± 1.36	14.84 ± 0.35	22.25 ± 1.34	18.88 ± 1.77
**7l**	41.47 ± 2.24	39.84 ± 0.76	0	0
**7m**	15.85 ± 1.06	5.36 ± 1.72	72.19 ± 1.36	66.30 ± 2.91
**BT**[b]	62.14 ± 1.27	50.77 ± 0.69	45.36 ± 1.37	25.07 ± 0.64
**TC**[c]	65.27 ± 1.25	48.11 ± 0.78	47.82 ± 0.26	19.59 ± 0.29

[a] The experiments were repeated three times.

[b]BT = Bismerthiazol.

[c]TC = Thiodiazole Copper.

Supplementary Table 10. EC_{50} values of compounds with good antibacterial activities against *Xoo* and *Xac*.

Compounds	*Xoo*			*Xac*		
	Toxic regression equation	r	EC_{50} (μg/mL)[h]	Toxic regression equation	r	EC_{50} (μg/mL)[h]
**5f**	$y = 1.26x + 3.55$	0.98	17.23 ± 0.24ef	$y = 1.23x + 3.53$	0.98	15.67 ± 0.28e
**5j**	$y = 0.40x + 4.54$	0.96	17.90 ± 3.15e	$y = 1.18x + 3.49$	0.99	19.25 ± 0.46d
**5k**	$y = 0.50x + 4.38$	0.99	25.80 ± 1.52ab	$y = 0.90x + 3.62$	0.99	34.06 ± 0.88c
**5m**	$y = 1.87x + 3.57$	0.97	5.88 ± 0.19j	$y = 1.77x + 3.21$	0.99	10.33 ± 0.11f
**5o**	$y = 1.57x + 3.36$	0.99	11.07 ± 0.37i	$y = 1.45x + 3.54$	0.95	10.27 ± 0.21f
**5s**	$y = 1.65x + 3.57$	0.99	7.38 ± 0.33j	$y = 1.19x + 3.93$	0.97	7.86 ± 0.16g
**5v**	$y = 1.54x + 3.24$	0.98	14.01 ± 0.60gh	$y = 2.11x + 2.61$	0.97	13.72 ± 0.14e
**5w**	$y = 1.61x + 3.30$	0.99	11.48 ± 0.90i	$y = 1.37x + 3.56$	0.96	11.23 ± 0.63f
**7d**	$y = 1.57x + 3.41$	0.98	10.32 ± 0.40i	$y = 1.29x + 3.73$	0.96	9.76 ± 0.92fg
**7e**	$y = 1.40x + 3.15$	0.99	21.02 ± 0.46d	$y = 1.42x + 3.36$	0.99	14.51 ± 0.44e
**7h**	$y = 0.72x + 4.14$	0.99	15.43 ± 0.27fg	$y = 1.36x + 2.95$	0.99	32.03 ± 0.88c
**7i**	$y = 1.07x + 3.47$	0.96	26.50 ± 1.16a	$y = 1.51x + 3.07$	0.96	19.02 ± 0.73d
**7j**	$y = 1.67x + 3.13$	0.99	13.32 ± 0.26h	$y = 1.33x + 2.99$	0.98	32.03 ± 0.88c
**BT**[i]	$y = 1.22x + 3.32$	0.98	23.98 ± 0.96bc	$y = 1.52x + 2.38$	0.98	52.67 ± 0.34b
**TC**[j]	$y = 1.28x + 3.27$	0.95	22.71 ± 1.26cd	$y = 1.49x + 2.30$	0.98	65.10 ± 1.98a

[h] The experiments were repeated three times.

[i] BT = Bismerthiazol.

[j] TC = Thiodiazole Copper.

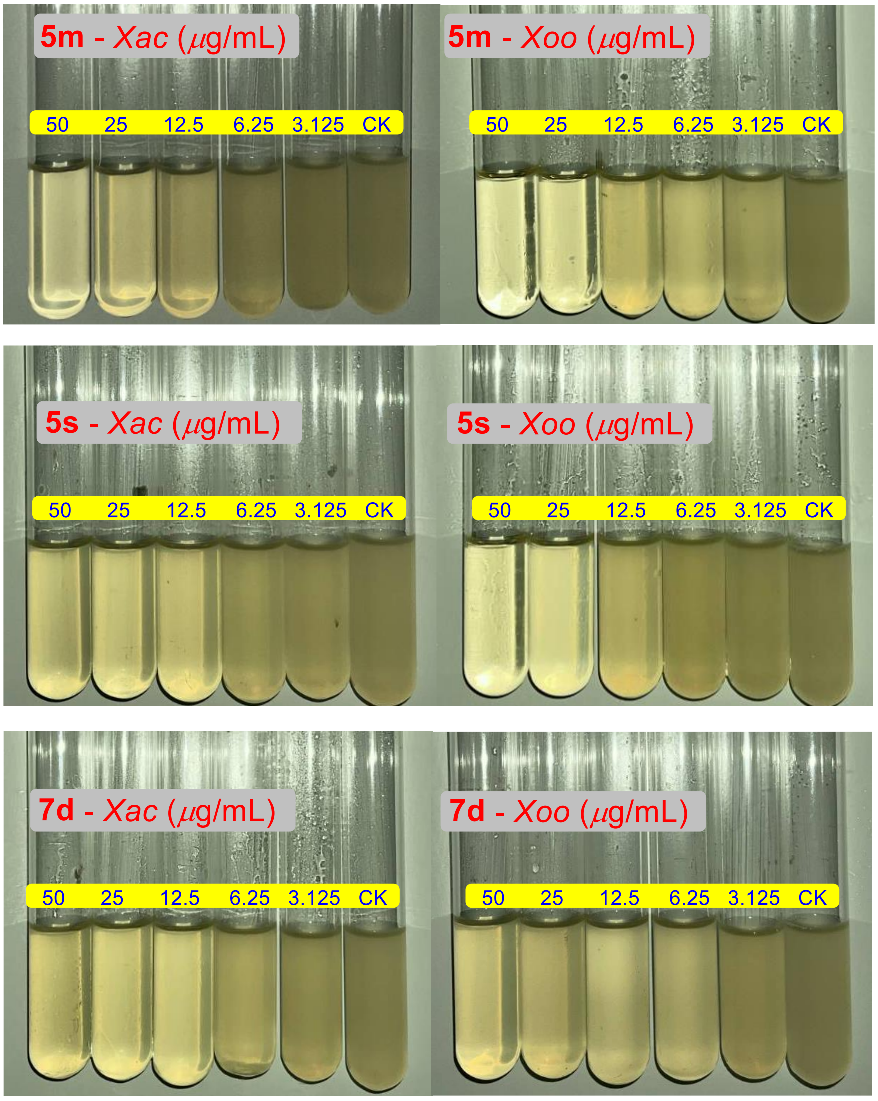

4

Supplementary Figure 21. Antibacterial activities of the compounds **5m**, **5s** and **7d** against *Xac* and *Xoo* test *in vitro* (CK = Control)

Cytotoxicity of the compounds against human cells

Moreover, to verify whether the compounds **5m**, **5s** and **7d** had potential mammalian toxicity, the structures of benzofuran and the compounds **5m**, **5s** and **7d** were submitted to the website (https://admetmesh.scbdd. com/). The predicted results showed that the toxicities of the 3-substituted benzofurans were significantly lower than the benzofuran. The results are displayed as follows:

(1) The hERG Blockers, Human Hepatotoxicity and Maximum Recommended Daily Dose (FDAMDD+) of all the benzofuran-derived molecules are low (**5m**: 0.208, 0.149, 0.083; **5s**: 0.014, 0.078, 0.046; **7d**: 0.081, 0.446, 0.261; benzofuran: 0.083, 0.058, 0.083).

(2) The AMES Toxicity and Eye Corrosion of **5m**, **5s** and **7d** are lower than the benzofuran (**5m**: 0.123, 0.016; **5s**: 0.106, 0.138; **7d**: 0.021, 0.007; benzofuran: 0.311, 0.912).

(3) The Rat Oral Acute Toxicity of the compound **5m**, **5s** and **7d** is lower than the benzofuran (**5m**: 0.689; **5s**: 0.112; **7d**: 0.482; benzofuran: 0.891).

Ⅱ. Supplementary Notes

Characterization of Products

1-iodo-2-(propa-1,2-dien-1-yloxy)benzene 1a:

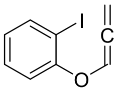

The reaction was performed following the literature procedures[1] with 2-iodophenol (10.00 g, 45.45 mmol) and propargyl bromide (6.49 g, 54.54 mmol). The crude product was purified by flash chromatography on silica gel (eluted with PE) to give the product **1a** (10.20 g, 87%) as a colorless oil. The ^{1}H and ^{13}C NMR spectra date for this compound match the literature date.[1]

^{1}H NMR (400 MHz, $CDCl_3$) δ 7.77 (dd, *J* = 7.8, 1.6 Hz, 1H), 7.29 (ddd, *J* = 8.2, 7.2, 1.6 Hz, 1H), 7.07 (dd, *J* = 8.2, 1.4 Hz, 1H), 6.84 – 6.75 (m, 2H), 5.43 (d, *J* = 5.8 Hz, 2H);

^{13}C NMR (101 MHz, $CDCl_3$) δ 202.4, 156.2, 139.7, 129.4, 124.8, 118.5, 116.8, 90.5, 87.3;

HRMS (APCI, m/z) calcd. for $C_9H_7ClIOH^+$: 258.9614, found: 258.9616.

1-chloro-2-iodo-3-(propa-1,2-dien-1-yloxy)benzene 1b:

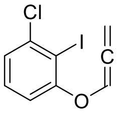

The reaction was performed following the literature procedures[1] with 3-chloro-2-iodophenol (500 mg, 1.97 mmol) and propargyl bromide (280.51 mg, 2.36 mmol). The crude product was purified by flash chromatography on silica gel (eluted with PE) to give the product **1b** (327.62 mg, 57%, m.p. 45 – 47 ^{o}C) as a white solid.

^{1}H NMR (400 MHz, $CDCl_3$) δ 7.29 – 7.13 (m, 2H), 6.94 (dd, *J* = 8.0, 1.6 Hz, 1H), 6.81 (t, *J* = 6.0 Hz, 1H), 5.46 (d, *J* = 6.0 Hz, 2H);

^{13}C NMR (101 MHz, $CDCl_3$) δ 202.3, 158.0, 140.0, 129.6, 124.0, 118.3, 114.2, 92.7, 90.7;

HRMS (ESI, m/z) calcd. for $C_9H_6ClIOH^+$: 292.9225, found: 292.9205.

4-fluoro-2-iodo-1-(propa-1,2-dien-1-yloxy)benzene 1c:

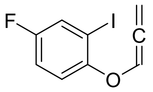

The reaction was performed following the literature procedures[1] with 4-fluoro-2-iodophenol (1.00 g, 4.20 mmol) and propargyl bromide (599.80 mg, 5.04 mmol). The crude product was purified by flash chromatography on silica gel (eluted with PE) to give the

product **1c** (848.10 g, 73%) as a pale yellow oil. The ^{1}H and ^{13}C NMR spectra date for this compound match the literature date.[7]

^{1}H NMR (400 MHz, $CDCl_3$) δ 7.53 – 7.46 (m, 1H), 7.07 – 7.00 (m, 2H), 6.81 (t, *J* = 5.8 Hz, 1H), 5.42 (d, *J* = 5.8 Hz, 2H);

^{13}C NMR (101 MHz, $CDCl_3$) δ 201.9, 158.2 (d, *J* = 246.9 Hz), 152.7 (d, *J* = 2.9 Hz), 126.1 (d, *J* = 25.3 Hz), 119.4, 118.1 (d, *J* = 8.0 Hz), 115.9 (d, *J* = 22.9 Hz), 91.0, 87.3 (d, *J* = 8.7 Hz);

^{19}F NMR (377 MHz, $CDCl_3$) δ -118.6;

HRMS (ESI, m/z) calcd. for $C_9H_6FIOH^+$: 276.9520, found: 276.9522.

4-chloro-2-iodo-1-(propa-1,2-dien-1-yloxy)benzene 1d:

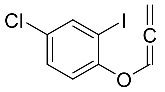

The reaction was performed following the literature procedures[1] with 4-chloro-2-iodophenol (300.00 mg, 1.18 mmol) and propargyl bromide (168.31 mg, 1.41 mmol). The crude product was purified by flash chromatography on silica gel (eluted with PE) to give the product **1d** (292.60 mg, 85%) as a colorless oil. The ^{1}H and ^{13}C NMR spectra date for this compound match the literature date.[7]

^{1}H NMR (400 MHz, $CDCl_3$) δ 7.75 (d, *J* = 2.4 Hz, 1H), 7.26 (dd, *J* = 8.6, 2.4 Hz, 1H), 6.99 (d, *J* = 8.6 Hz, 1H), 6.79 (t, *J* = 5.8 Hz, 1H), 5.44 (d, *J* = 5.8 Hz, 2H).

^{13}C NMR (101 MHz, $CDCl_3$) δ 202.1, 155.1, 138.7, 129.2, 128.8, 118.5, 117.4, 90.9, 87.6;

HRMS (ESI, m/z) calcd. for $C_9H_6ClIOH^+$: 292.9225, found: 292.9228.

2-iodo-1-(propa-1,2-dien-1-yloxy)-4-(trifluoromethyl)benzene 1e:

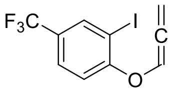

The reaction was performed following the literature procedures[1] with 2-iodo-4-(trifluoromethyl)phenol (300.00 mg, 1.04 mmol) and propargyl bromide (148.70 mg, 1.25 mmol). The crude product was purified by flash chromatography on silica gel (eluted with PE) to give the product **1e** (254.72 mg, 75%) as a colorless oil. The ^{1}H and ^{13}C NMR spectra date for this compound match the literature date.[7]

^{1}H NMR (400 MHz, $CDCl_3$) δ 8.03 (dd, *J* = 2.2, 0.8 Hz, 1H), 7.56 (ddd, *J* = 8.6, 2.2, 0.8 Hz, 1H), 7.12 (dd, *J* = 8.6, 0.8 Hz, 1H), 6.82 (t, *J* = 5.8 Hz, 1H), 5.49 (d, *J* = 5.8 Hz, 2H);

^{13}C NMR (101 MHz, $CDCl_3$) δ 202.4, 158.8 (d, *J* = 1.3 Hz), 136.9 (q, *J* = 3.8 Hz), 126.7 (q, *J* = 3.7 Hz), 126.4 (q, *J* = 33.2 Hz), 123.1 (q, *J* = 272.1 Hz), 117.3, 115.4, 90.8, 86.5;

^{19}F NMR (377 MHz, $CDCl_3$) δ -61.9;

HRMS (ESI, m/z) calcd. for $C_{10}H_6F_3IOH^+$: 326.9488, found: 326.9489.

2-iodo-4-methyl-1-(propa-1,2-dien-1-yloxy)benzene 1f:

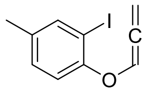

The reaction was performed following the literature procedures[1] with 2-iodo-4-methylphenol (300.00 mg, 1.28 mmol) and propargyl bromide (182.99 mg, 1.54 mmol). The crude product was purified by flash chromatography on silica gel (eluted with PE) to give the product **1f** (289.48 mg, 83%) as a colorless oil;

^{1}H NMR (400 MHz, $CDCl_3$) δ 7.60 (dd, *J* = 2.0, 0.8 Hz, 1H), 7.08 (ddd, *J* = 8.2, 2.2, 0.8 Hz, 1H), 6.95 (d, *J* = 8.2 Hz, 1H), 6.80 (t, *J* = 5.8 Hz, 1H), 5.41 (d, *J* = 5.8 Hz, 2H), 2.26 (s, 3H);

^{13}C NMR (101 MHz, $CDCl_3$) δ 202.3, 154.1, 139.8, 134.7, 129.9, 119.1, 117.0, 90.5, 87.3, 20.2;

HRMS (ESI, m/z) calcd. for $C_{10}H_9IOH^+$: 272.9771, found: 272.9769.

4-(*tert*-butyl)-2-iodo-1-(propa-1,2-dien-1-yloxy)benzene 1g:

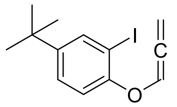

The reaction was performed following the literature procedures[1] with 4-(*tert*-butyl)-2-iodophenol (300.00 mg, 1.09 mmol) and propargyl bromide (155.10 mg, 1.30 mmol). The crude product was purified by flash chromatography on silica gel (eluted with PE) to give the product **1g** (256.01 mg, 75%) as a colorless oil. The ^{1}H and ^{13}C NMR spectra date for this compound match the literature date.[7]

^{1}H NMR (400 MHz, $CDCl_3$) δ 7.76 (d, *J* = 2.4 Hz, 1H), 7.30 (dd, *J* = 8.6, 2.4 Hz, 1H), 6.99 (d, *J* = 8.5 Hz, 1H), 6.80 (t, *J* = 5.9 Hz, 1H), 5.43 (d, *J* = 5.9 Hz, 2H), 1.28 (s, 9H);

^{13}C NMR (101 MHz, $CDCl_3$) δ 202.4, 154.0, 148.0, 136.6, 126.4, 118.7, 116.3, 90.4, 87.1, 34.2, 31.4;

HRMS (ESI, m/z) calcd. for $C_{13}H_{16}IOH^+$: 315.0240, found: 315.0241.

1-iodo-2-(propa-1,2-dien-1-yloxy)naphthalene 1h:

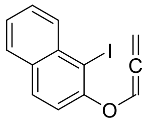

The reaction was performed following the literature procedures[1] with 1-iodonaphthalen-2-ol (300.00 mg, 1.11 mmol) and propargyl bromide (158.57 mg, 1.33 mmol). The crude product was purified by flash chromatography on silica gel (eluted with PE) to give the product **1h** (284.08 mg, 83%, m.p. 50 – 52 °C) as a yellow solid. The ^{1}H and ^{13}C NMR spectra date for this compound match the literature date.[7]

^{1}H NMR (400 MHz, $CDCl_3$) δ 8.15 (dq, *J* = 8.4, 0.8 Hz, 1H), 7.81 – 7.69 (m, 2H), 7.55 (ddd, *J* = 8.4, 6.8, 1.4 Hz, 1H), 7.42 (ddd, *J* = 8.0, 6.8, 1.2 Hz, 1H), 7.32 (d, *J* = 8.8 Hz, 1H), 6.95 (t, *J* = 5.8 Hz, 1H), 5.40 (d, *J* = 5.8 Hz, 2H);

^{13}C NMR (101 MHz, $CDCl_3$) δ 202.1, 154.7, 135.5, 131.8, 131.1, 130.1, 128.3, 128.2, 125.4, 119.6, 118.1, 91.0, 90.6;

HRMS (ESI, m/z) calcd. for $C_{13}H_9IOH^+$: 308.9771, found: 308.9770.

1-iodo-4-methoxy-2-(propa-1,2-dien-1-yloxy)benzene 1i:

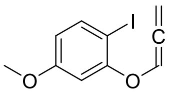

The reaction was performed following the literature procedures[1] with 2-iodo-5-methoxyphenol (300.00 mg, 1.20 mmol) and propargyl bromide (171.28 mg, 1.44 mmol). The crude product was purified by flash chromatography on silica gel (eluted with PE / EA 300:1) to give the product **1i** (279.98 mg, 81%) as a colorless oil. The ^{1}H and ^{13}C NMR spectra date for this compound match the literature date.[7]

^{1}H NMR (400 MHz, $CDCl_3$) δ 7.62 (d, *J* = 8.6 Hz, 1H), 6.79 (t, *J* = 5.8 Hz, 1H), 6.67 (d, *J* = 2.8 Hz, 1H), 6.43 (dd, *J* = 8.6, 2.8 Hz, 1H), 5.45 (d, *J* = 5.8 Hz, 2H), 3.79 (s, 3H);

^{13}C NMR (101 MHz, $CDCl_3$) δ 202.5, 161.1, 157.0, 139.4, 118.1, 110.3, 103.7, 90.4, 75.6, 55.6;

HRMS (ESI, m/z) calcd. for $C_{10}H_9IO_2H^+$: 288.9720, found: 288.9718.

1-bromo-3-iodo-2-(propa-1,2-dien-1-yloxy)benzene 1j:

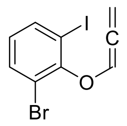

The reaction was performed following the literature procedures[1] with 2-bromo-6-iodophenol (300 mg, 1.00 mmol) and propargyl bromide (143.28 mg, 1.20 mmol). The crude product was purified by flash chromatography on silica gel (eluted with PE) to give the product **1j** (280.70 mg, 83%) as a colorless oil;

^{1}H NMR (400 MHz, $CDCl_3$) δ 7.72 (dd, *J* = 8.0, 1.4 Hz, 1H), 7.53 (dd, *J* = 8.0, 1.4 Hz, 1H), 6.94 (t, *J* = 6.0 Hz, 1H), 6.75 (t, *J* = 8.0 Hz, 1H), 5.31 (d, *J* = 6.0 Hz, 2H);

^{13}C NMR (101 MHz, $CDCl_3$) δ 200.2, 152.8, 138.6, 133.6, 127.8, 121.1, 117.0, 92.6, 92.2;

HRMS (ESI, m/z) calcd. for $C_9H_6BrIOH^+$: 336.8719, found: 336.8714.

1-(buta-2,3-dien-2-yloxy)-2-iodobenzene 1k:

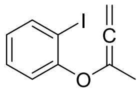

The reaction was performed following the literature procedures[2] with 2-iodophenol (1.00 g, 4.55 mmol) and but-3-yn-2-ol (318.58 mg, 4.55 mmol). The crude product was purified by flash chromatography on silica gel (eluted with PE) to give the product **1k** (1.08 g, 87%) as a colorless oil. The ^{1}H and ^{13}C NMR spectra date for this compound match the literature date.[7]

^{1}H NMR (400 MHz, $CDCl_3$) δ 7.76 (dd, *J* = 7.8, 1.4 Hz, 1H), 7.26 (ddd, *J* = 8.0, 7.2, 1.6 Hz, 1H), 7.06 (dd, *J* = 8.0, 1.4 Hz, 1H), 6.81 (ddd, *J* = 7.8, 7.2, 1.4 Hz, 1H), 5.12 (q, *J* = 3.0 Hz, 2H), 2.11 (t, *J* = 3.0 Hz, 3H);

^{13}C NMR (101 MHz, $CDCl_3$)δ 200.0, 155.7, 139.3, 129.1, 129.1, 125.5, 120.5, 89.7, 89.1, 18.1；

HRMS (ESI, m/z) calcd. for $C_{10}H_9IO_2H^+$: 272.9771, found: 272.9769.

1-iodo-2-(octa-1,2-dien-3-yloxy)benzene 1l:

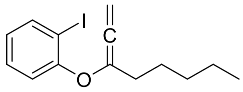

The reaction was performed following the literature procedures[2] with 2-iodophenol (1.00 g, 4.55 mmol) and oct-1-yn-3-ol (573.61 mg, 4.55 mmol). The crude product was purified by flash chromatography on silica gel (eluted with PE) to give the product **1l** (1.27 g, 85%) as a colorless oil;

^{1}H NMR (400 MHz, $CDCl_3$) δ 7.75 (dd, *J* = 8.0, 1.6 Hz, 1H), 7.30 – 7.21 (m, 1H), 7.05 (dd, *J* = 8.0, 1.6 Hz, 1H), 6.79 (td, *J* = 7.6, 1.6 Hz, 1H), 5.16 (t, *J* = 3.0 Hz, 2H), 2.37 (tt, *J* = 7.8, 2.8 Hz, 2H), 1.61 (m, *J* = 15.0, 8.0, 6.0 Hz, 2H), 1.45 – 1.29 (m, 4H), 0.91 (t, *J* = 7.0 Hz, 3H);

^{13}C NMR (101 MHz, $CDCl_3$) δ 199.7, 155.7, 139.3, 133.0, 129.0, 125.3, 120.3, 89.8, 89.5, 31.5, 31.4, 26.1, 22.5, 14.1;

HRMS (ESI, m/z) calcd. for $C_{14}H_{17}IOH^+$: 329.0397, found: 329.0390.

1-((4-ethylocta-1,2-dien-3-yl)oxy)-2-iodobenzene 1m:

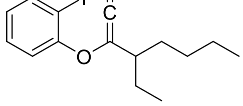

The reaction was performed following the literature procedures[2] with 2-iodophenol (1.00 g, 4.55 mmol) and 4-ethyloct-1-yn-3-ol (701.12 mg, 4.55 mmol). The crude product was purified by flash chromatography on silica gel (eluted with PE) to give the product **1m** (1.31 g, 81%) as a colorless oil;

^{1}H NMR (400 MHz, $CDCl_3$) δ 7.75 (dd, *J* = 8.0, 1.6 Hz, 1H), 7.30 – 7.22 (m, 1H), 7.06 (dd, *J* = 8.2, 1.6 Hz, 1H), 6.80 (td, *J* = 7.6, 1.6 Hz, 1H), 5.17 (d, *J* = 1.4 Hz, 2H), 2.24 – 2.15 (m, 1H), 1.76 – 1.43 (m, 5H), 1.42 – 1.29 (m, 3H), 1.01 (t, *J* = 7.4 Hz, 3H), 0.92 (t, *J* = 7.2 Hz, 3H);

^{13}C NMR (101 MHz, $CDCl_3$) δ 199.5, 155.7, 139.3, 134.9, 128.9, 125.2, 120.6, 89.8, 89.6, 43.8, 31.8, 29.5, 25.4, 22.8, 14.2, 11.8;

HRMS (ESI, m/z) calcd. for $C_{16}H_{21}IOH^+$: 357.0710, found: 357.0705.

(benzofuran-3-ylmethyl)diphenylphosphine oxide 3a:

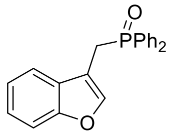

The reaction was performed with **1a** (25.81 mg, 0.10 mmol) and $HPPh_2$ (37.24 mg, 0.20 mmol). The crude product was purified by flash chromatography on silica gel (eluted with PE / EA = 3:1) to give the product **3a** (26.58 mg, 80% yield, m.p. 135 – 137 °C) as a white solid;

^{1}H NMR (400 MHz, $CDCl_3$) δ 7.80 – 7.70 (m, 4H), 7.55 – 7.48 (m, 3H), 7.47 – 7.35 (m, 6H), 7.23 (dd, *J* = 8.2, 1.2 Hz, 1H), 7.18 – 7.11 (m, 1H), 3.69 (dd, *J* = 13.2, 0.8 Hz, 2H);

^{13}C NMR (101 MHz, $CDCl_3$) δ 154.8, 143.6 (d, *J* = 7.3 Hz), δ132.2 (d, *J* = 99.9 Hz), 132.1 (d, *J* = 2.6 Hz), 131.0 (d, *J* = 9.3 Hz), 128.7 (d, *J* = 11.8 Hz), 128.0 (d, *J* = 5.1 Hz),124.4, 122.6, 119.5, 111.4, 110.3 (d, *J* = 8.1 Hz), 26.1 (d, *J* = 70.1 Hz);

^{31}P NMR (162 MHz, $CDCl_3$) δ 29.2;

HRMS (ESI, m/z) calcd. for $C_{21}H_{17}O_2PH^+$: 333.1044, found: 333.1046.

((4-chlorobenzofuran-3-yl)methyl)diphenylphosphine oxide 3b:

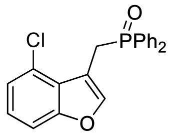

The reaction was performed with **1b** (29.25 mg, 0.10 mmol) and $HPPh_2$ (37.24 mg, 0.20 mmol). The crude product was purified by flash chromatography on silica gel (eluted with PE / EA = 3:1) to give the product **3b** (23.48 mg, 64% yield, m.p. 135 – 137 °C) as a white solid;

^{1}H NMR (400 MHz, $CDCl_3$) δ 7.86 – 7.69 (m, 5H), 7.52 – 7.41 (m, 6H), 7.33 (dd, *J* = 7.2, 1.6 Hz, 1H), 7.14 (p, *J* = 7.8 Hz, 2H), 4.08 (d, *J* = 12.8 Hz, 2H);

^{13}C NMR (101 MHz, $CDCl_3$) δ 155.6, 145.1 (d, *J* = 6.5 Hz), 132.2 (d, *J* = 100.2 Hz), 132.0 (d, *J* = 2.7 Hz), 131.0 (d, *J* = 9.3 Hz), 128.7 (d, *J* = 11.8 Hz), 126.0, 124.9 (d, *J* = 6.4 Hz), 124.9, 123.8, 110.6, 110.3 (d, *J* = 7.4 Hz), 25.8 (d, *J* = 69.3 Hz);

^{31}P NMR (162 MHz, $CDCl_3$) δ 29.8;

HRMS (ESI, m/z) calcd. for $C_{21}H_{16}ClO_2PH^+$: 367.0655, found: 367.0652.

((5-fluorobenzofuran-3-yl)methyl)diphenylphosphine oxide 3c:

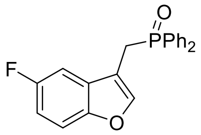

The reaction was performed with **1c** (27.60 mg, 0.10 mmol) and $HPPh_2$ (37.24 mg, 0.20 mmol). The crude product was purified by flash chromatography on silica gel (eluted with PE / EA = 3:1) to give the product **3c** (22.07 mg, 63% yield, m.p. 99 – 101 °C) as a white solid;

^{1}H NMR (400 MHz, $CDCl_3$) δ 7.80 – 7.71 (m, 4H), 7.59 – 7.50 (m, 3H), 7.49 – 7.41 (m, 4H), 7.32 (dd, *J* = 8.8, 4.0 Hz, 1H), 7.00 (dd, *J* = 8.6, 2.6 Hz, 1H), 6.94 (m, 1H), 3.64 (dd, *J* = 13.0, 0.8 Hz, 2H);

^{13}C NMR (101 MHz, $CDCl_3$) δ 159.1 (d, *J* = 238.4 Hz), 151.0, 145.4 (d, *J* = 7.3 Hz), 132.2 (d, *J* = 2.8 Hz), 132.0 (d, *J* = 100.0 Hz), 131.0 (d, *J* = 9.3 Hz), 128.8 (dd, *J* = 10.4 Hz), 128.8 (d, *J* = 11.8 Hz), 112.2 (d, *J* = 32.6 Hz), 112.1 (d, *J* = 3.4 Hz), 110.7 (dd, *J* = 8.0, 4.2 Hz), 105.3 (d, *J* = 25.4 Hz), 26.2 (d, *J* = 69.8 Hz).

^{19}F NMR (376 MHz, $CDCl_3$) δ -120.9 – -121.0 (m);

^{31}P NMR (162 MHz, $CDCl_3$) δ 28.9;

HRMS (ESI, m/z) calcd. for $C_{21}H_{16}FO_2PH^+$: 351.0950, found: 351.0955.

((5-chlorobenzofuran-3-yl)methyl)diphenylphosphine oxide 3d:

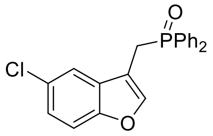

The reaction was performed with **1d** (29.25 mg, 0.10 mmol) and $HPPh_2$ (37.24 mg, 0.20 mmol). The crude product was purified by flash chromatography on silica gel (eluted with PE / EA = 3:1) to give the product **3d** (24.58 mg, 67% yield, m.p. 182 – 184 °C) as a white solid;

^{1}H NMR (400 MHz, $CDCl_3$) δ 7.79 – 7.71 (m, 4H), 7.59 – 7.50 (m, 3H), 7.48 – 7.43 (m, 4H), 7.31 (d, *J* = 8.8 Hz, 1H), 7.24 (d, *J* = 2.0 Hz, 1H), 7.18 (dd, *J* = 8.8, 2.0 Hz, 1H), 3.64 (d, *J* = 13.0 Hz, 2H);

^{13}C NMR (101 MHz, $CDCl_3$) δ 153.2, 145.1 (d, *J* = 7.3 Hz), 132.2 (d, *J* = 2.7 Hz), 132.0 (d, *J* = 99.9 Hz), 131.0 (d, *J* = 9.2 Hz), 129.3 (d, *J* = 4.5 Hz), 128.8 (d, *J* = 11.9 Hz), 128.3, 124.7, 119.3, 112.4, 110.2 (d, *J* = 8.1 Hz), 26.2 (d, *J* = 69.7 Hz);

^{31}P NMR (162 MHz, $CDCl_3$) δ 28.6;

HRMS (ESI, m/z) calcd. for $C_{21}H_{16}ClO_2PH^+$: 367.0655, found: 367.0657.

diphenyl((5-(trifluoromethyl)benzofuran-3-yl)methyl)phosphine oxide 3e:

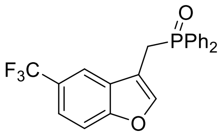

The reaction was performed with **1e** (32.61 mg, 0.10 mmol) and $HPPh_2$ (37.24 mg, 0.20 mmol). The crude product was purified by flash chromatography on silica gel (eluted with PE / EA = 3:1) to give the product **3e** (26.02 mg, 65% yield, m.p. 123 – 125 °C) as a yellow solid;

^{1}H NMR (400 MHz, $CDCl_3$) δ 7.80 – 7.72 (m, 4H), 7.68 (d, *J* = 3.2 Hz, 1H), 7.57 – 7.39 (m, 9H), 3.71 (d, *J* = 12.8 Hz, 2H);

^{13}C NMR (101 MHz, $CDCl_3$) δ156.2, 145.5 (d, *J* = 7.3 Hz), 132.3 (d, *J* = 2.7 Hz), 131.9 (d, *J* = 100.1 Hz), 131.0 (d, *J* = 9.4 Hz), 128.8 (d, *J* = 12.0 Hz), 128.0 (d, *J* = 4.5 Hz), 125.2 (q, *J* = 32.1 Hz), 124.5 (q, *J* = 271.8 Hz), 121.5 (q, *J* = 3.5 Hz), 117.3 (q, *J* = 4.1 Hz), 111.9, 110.8 (d, *J* = 8.1 Hz), 26.2 (d, *J* = 69.2 Hz);

^{19}F NMR (377 MHz, $CDCl_3$) δ -60.7;

^{31}P NMR (162 MHz, $CDCl_3$) δ 28.5;

HRMS (ESI, m/z) calcd. for $C_{22}H_{16}F_3O_2PH^+$: 401.0918, found: 401.0917.

((5-methylbenzofuran-3-yl)methyl)diphenylphosphine oxide 3f:

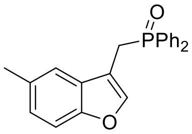

The reaction was performed with **1f** (27.21 mg, 0.10 mmol) and $HPPh_2$ (37.24 mg, 0.20 mmol). The crude product was purified by flash chromatography on silica gel (eluted with PE / EA = 3:1) to give the product **3f** (30.14 mg, 87% yield, m.p. 173 – 175 °C) as a white solid;

^{1}H NMR (400 MHz, $CDCl_3$) δ 7.81 – 7.70 (m, 4H), 7.55 – 7.48 (m, 2H), 7.46 – 7.42 (m, 5H), 7.26 (d, *J* = 1.6 Hz, 1H), 7.10 (s, 1H), 7.04 (d, *J* = 8.4 Hz, 1H), 3.66 (d, *J* = 13.2 Hz, 2H), 2.36 (s, 3H);

^{13}C NMR (101 MHz, $CDCl_3$) δ153.3, 143.7 (d, *J* = 7.3 Hz), 132.4 (d, *J* = 99.6 Hz), 132.0 (d, *J* = 2.9 Hz), 131.1 (d, *J* = 9.1 Hz), 128.6 (d, *J* = 11.9 Hz), 128.0 (d, *J* = 4.6 Hz), 125.7, 119.4, 110.9, 110.0 (d, *J* = 8.1 Hz), 26.2 (d, *J* = 70.2 Hz), 21.3;

^{31}P NMR (162 MHz, $CDCl_3$) δ 29.1;

HRMS (ESI, m/z) calcd. for $C_{22}H_{19}O_2PH^+$: 347.1201, found: 347.1199.

((5-(tert-butyl)benzofuran-3-yl)methyl)diphenylphosphine oxide 3g:

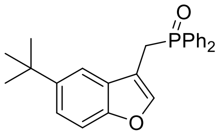

The reaction was performed with **1g** (31.42 mg, 0.10 mmol) and $HPPh_2$ (37.24 mg, 0.20 mmol). The crude product was purified by flash chromatography on silica gel (eluted with PE / EA = 3:1) to give the product **3g** (25.63 mg, 66% yield, m.p. 116 – 118 °C) as a yellow solid;

^{1}H NMR (400 MHz, $CDCl_3$) δ 7.80 – 7.70 (m, 4H), 7.54 – 7.47 (m, 3H), 7.46 – 7.40 (m, 4H), 7.34 – 7.29 (m, 2H), 7.25 (d, *J* = 1.4 Hz, 1H), 3.71 (dd, *J* = 13.4, 0.8 Hz, 2H), 1.30 (s, 9H);

^{13}C NMR (101 MHz, $CDCl_3$) δ 153.0, 145.5, 143.9 (d, *J* = 7.3 Hz), 132.3 (d, *J* = 99.7 Hz), 132.0 (d, *J* = 2.7 Hz), 131.1 (d, *J* = 9.4 Hz), 128.6 (d, *J* = 11.8 Hz), 127.5 (d, *J* = 5.0 Hz), 122.4, 115.3, 110.7, 110.2 (d, *J* = 7.9 Hz),34.7, 31.8, 26.0 (d, *J* = 70.1 Hz);

^{31}P NMR (162 MHz, $CDCl_3$) δ 29.0;

HRMS (ESI, m/z) calcd. for $C_{25}H_{25}O_2PH^+$: 389.1670, found: 389.1671.

(naphtho[2,1-b]furan-1-ylmethyl)diphenylphosphine oxide 3h:

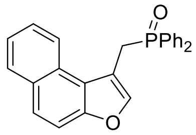

The reaction was performed with **1h** (30.81 mg, 0.10 mmol) and $HPPh_2$ (37.24 mg, 0.20 mmol). The crude product was purified by flash chromatography on silica gel (eluted with PE / EA = 3:1) to give the product **3h** (28.30 mg, 74% yield, m.p. 231 – 233 °C) as a white solid;

^{1}H NMR (400 MHz, $CDCl_3$) δ 8.31 (d, *J* = 8.2 Hz, 1H), 7.93 (d, *J* = 7.7 Hz, 1H), 7.86 – 7.73 (m, 5H), 7.70 (d, *J* = 9.2 Hz, 1H), 7.59 (d, *J* = 8.8 Hz, 1H), 7.55 – 7.38 (m, 8H), 4.13 (dd, *J* = 13.2, 0.8 Hz, 2H);

^{13}C NMR (101 MHz, $CDCl_3$) δ 152.9, 143.4 (d, *J* = 7.4 Hz), 132.4 (d, *J* = 100.1 Hz), 132.1 (d, *J* = 2.6 Hz), 131.1 (d, *J* = 9.3 Hz), 130.8, 129.2, 128.8 (d, *J* = 12.0 Hz), 128.5, 126.3, 125.9, 124.2, 122.6, 121.1 (d, *J* = 6.6 Hz), 112.7, 112.0 (d, *J* = 7.1 Hz), 28.0 (d, *J* = 69.5 Hz);

^{31}P NMR (162 MHz, $CDCl_3$) δ 29.5;

HRMS (ESI, m/z) calcd. for $C_{25}H_{19}O_2PH^+$: 383.1201, found: 383.1202.

((6-methoxybenzofuran-3-yl)methyl)diphenylphosphine oxide 3i:

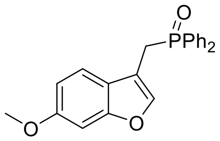

The reaction was performed with **1i** (28.81 mg, 0.10 mmol) and $HPPh_2$ (37.24 mg, 0.20 mmol). The crude product was purified by flash chromatography on silica gel (eluted with PE / EA = 3:1) to give the product **3i** (18.12 mg, 50% yield, m.p. 147 – 149 °C) as a yellow solid;

^{1}H NMR (400 MHz, $CDCl_3$) δ 7.78 – 7.69 (m, 4H), 7.54 – 7.48 (m, 2H), 7.47 – 7.39 (m, 5H), 7.24 (d, *J* = 8.6 Hz, 1H), 6.93 (d, *J* = 2.4 Hz, 1H), 6.78 (dd, *J* = 8.6, 2.2 Hz, 1H), 3.81 (s, 3H), 3.65 (d, *J* = 13.2 Hz, 2H);

^{13}C NMR (101 MHz, $CDCl_3$) δ158.1, 155.9, 142.6 (d, *J* = 7.6 Hz), 132.3 (d, *J* = 99.8 Hz), 132.0 (d, *J* = 2.7 Hz), 131.0 (d, *J* = 9.1 Hz), 128.7 (d, *J* = 11.7 Hz), 121.3 (d, *J* = 5.0 Hz), 119.8, 111.7, 110.2 (d, *J* = 8.1 Hz), 95.8, 55.7, 26.3 (d, *J* = 70.3 Hz);

^{31}P NMR (162 MHz, $CDCl_3$) δ 29.2;

HRMS (ESI, m/z) calcd. for $C_{22}H_{19}O_3PH^+$: 363.1150, found: 363.1150.

((7-bromobenzofuran-3-yl)methyl)diphenylphosphine oxide 3j:

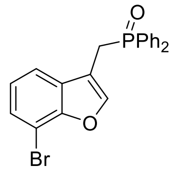

The reaction was performed with **1j** (33.70 mg, 0.10 mmol) and $HPPh_2$ (37.24 mg, 0.20 mmol). The crude product was purified by flash chromatography on silica gel (eluted with PE / EA = 3:1) to give the product **3j** (21.82 mg, 63% yield, m.p. 139 – 141 ^{o}C) as a white solid;

^{1}H NMR (400 MHz, $CDCl_3$) δ 7.81 – 7.70 (m, 4H), 7.55 – 7.49 (m, 3H), 7.46 – 7.38 (m, 5H), 7.24 – 7.03 (m, 2H), 3.69 (d, *J* = 13.2 Hz, 2H);

^{13}C NMR (101 MHz, $CDCl_3$) δ 154.8, 143.6 (d, *J* = 7.3 Hz), 132.3 (d, *J* = 99.6 Hz), 132.0 (d, *J* = 2.6 Hz), 131.0 (d, *J* = 9.1 Hz), 128.7 (d, *J* = 11.7 Hz), 128.0 (d, *J* = 4.9 Hz), 124.4, 122.6, 119.5, 111.4, 110.3 (d, *J* = 7.9 Hz), 26.1 (d, *J* = 70.1 Hz);

^{31}P NMR (162 MHz, $CDCl_3$) δ 29.1;

HRMS (ESI, m/z) calcd. for $C_{21}H_{16}BrO_2PH^+$: 411.0150, found: 411.0142.

((2-methylbenzofuran-3-yl)methyl)diphenylphosphine oxide 3k:

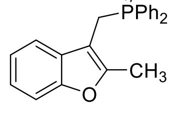

The reaction was performed with **1k** (27.21 mg, 0.10 mmol) and $HPPh_2$ (37.24 mg, 0.20 mmol). The crude product was purified by flash chromatography on silica gel (eluted with PE / EA = 3:1) to give the product **3k** (31.18 mg, 90% yield, m.p. 196 – 198 ^{o}C) as a white solid;

^{1}H NMR (400 MHz, $CDCl_3$) δ 7.78 – 7.66 (m, 4H), 7.54 – 7.47 (m, 2H), 7.41 (tt, *J* = 7.6, 2.0 Hz, 4H), 7.30 (d, *J* = 8.2 Hz, 1H), 7.21 – 7.08 (m, 2H), 7.02 (t, *J* = 7.6 Hz, 1H), 3.60 (dd, *J* = 13.0, 2.2 Hz, 2H), 2.14 (dd, *J* = 3.2, 1.8 Hz, 3H);

^{13}C NMR (101 MHz, $CDCl_3$) δ153.7, 153.3 (d, *J* = 8.1 Hz), 132.5 (d, *J* = 97.7 Hz), 131.9 (d, *J* = 2.9 Hz), 131.2 (d, *J* = 9.3 Hz), 129.3 (d, *J* = 1.9 Hz), 128.6 (d, *J* = 11.6 Hz), 123.2, 122.2, 119.4, 110.4, 104.9 (d, *J* = 8.8 Hz), 27.4 (d, *J* = 70.3 Hz), 12.1 (d, *J* = 2.1 Hz);

^{31}P NMR (162 MHz, $CDCl_3$) δ 28.6;

HRMS (ESI, m/z) calcd. for $C_{22}H_{19}O_2PNa^+$: 369.1015, found: 369.1017.

((2-pentylbenzofuran-3-yl)methyl)diphenylphosphine oxide 3l:

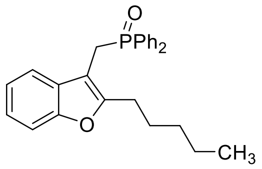

The reaction was performed with **1l** (32.82 mg, 0.10 mmol) and $HPPh_2$ (37.24 mg, 0.20 mmol). The crude product was purified by flash chromatography on silica gel (eluted with PE / EA = 3:1) to give the product **3l** (30.19 mg, 75% yield, m.p. 151 – 153 °C) as a white solid;

^{1}H NMR (400 MHz, $CDCl_3$) δ 7.72 (ddt, *J* = 11.4, 7.0, 1.4 Hz, 4H), 7.50 (td, *J* = 7.4, 1.6 Hz, 2H), 7.42 (td, *J* = 7.6, 3.0 Hz, 4H), 7.31 (d, *J* = 8.2 Hz, 1H), 7.22 (d, *J* = 7.8 Hz, 1H), 7.13 (td, *J* = 8.2, 7.8, 1.4 Hz, 1H), 7.06 – 6.99 (m, 1H), 3.63 (d, *J* = 13.0 Hz, 2H), 2.47 (td, *J* = 7.8, 2.2 Hz, 2H), 1.49 (p, *J* = 7.6 Hz, 2H), 1.27 – 1.17 (m, 4H), 0.86 (t, *J* = 7.0 Hz, 3H);

^{13}C NMR (101 MHz, $CDCl_3$) δ 157.1 (d, *J* = 8.4 Hz), 153.8, 132.5 (d, *J* = 98.1 Hz), 131.9 (d, *J* = 2.7 Hz), 131.2 (d, *J* = 8.8 Hz), 129.3 (d, *J* = 1.6 Hz), 128.6 (d, *J* = 11.6 Hz), 123.2, 122.2, 119.7, 110.4, 104.4 (d, *J* = 8.5 Hz), 31.6, 27.5 (d, *J* = 1.3 Hz), 27.4 (d, *J* = 70.5 Hz), 26.5 (d, *J* = 1.2 Hz), 22.4, 14.0;

^{31}P NMR (162 MHz, $CDCl_3$) δ 28.3;

HRMS (ESI, m/z) calcd. for $C_{26}H_{27}O_2PNa^+$: 425.1641, found: 425.1653.

((2-(heptan-3-yl)benzofuran-3-yl)methyl)diphenylphosphine oxide 3m:

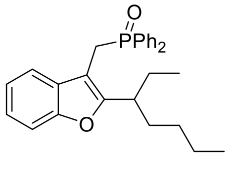

The reaction was performed with **1m** (35.62 mg, 0.10 mmol) and $HPPh_2$ (37.24 mg, 0.20 mmol). The crude product was purified by flash chromatography on silica gel (eluted with PE / EA = 3:1) to give the product **3m** (27.98 mg, 65% yield, m.p. 161 – 163 °C) as a white solid;

^{1}H NMR (400 MHz, $CDCl_3$) δ 7.80 – 7.68 (m, 4H), 7.53 – 7.45 (m, 2H), 7.45 – 7.36 (m, 4H), 7.35 – 7.29 (m, 2H), 7.13 (ddd, *J* = 8.4, 7.2, 1.4 Hz, 1H), 7.04 (td, *J* = 7.6, 1.2 Hz, 1H), 3.66 (d, *J* = 13.0 Hz, 2H), 2.67 (tt, *J* = 8.4, 6.0 Hz, 1H), 1.64 – 1.42 (m, 4H), 1.29 – 1.06 (m, 3H), 1.05 – 0.92 (m, 1H), 0.81 (t, *J* = 7.2 Hz, 3H), 0.73 (t, *J* = 7.4 Hz, 3H);

^{13}C NMR (101 MHz, $CDCl_3$) δ 159.4 (d, J = 8.7 Hz), 153.8, 132.7 (dd, J = 98.1, 3.5 Hz), 131.9 (d, J = 2.8 Hz), 131.2 (d, J = 8.9 Hz), 129.2 (d, J = 1.5 Hz), 128.6 (dd, J = 11.6, 2.9 Hz), 123.1, 122.1, 120.0, 110.5, 105.3 (d, J = 8.1 Hz), 39.1, 33.2, 29.7, 27.4 (d, J = 70.7 Hz), 26.8 (d, J = 1.4 Hz), 22.9, 14.0, 12.0;

^{31}P NMR (162 MHz, $CDCl_3$) δ 28.1;

HRMS (ESI, m/z) calcd. for $C_{28}H_{31}O_2PNa^+$: 453.1954, found: 453.1959.

3-((*o*-tolylthio)methyl)benzofuran 5a:

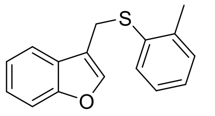

The reaction was performed with 2-methylbenzenethiol (12.42 mg, 0.10 mmol) and **1a** (38.71 mg, 0.15 mmol). The crude product was purified by flash chromatography on silica gel (eluted with PE / EA = 300:1) to give the product **5a** (24.16 mg, 95% yield) as a yellow oil;

^{1}H NMR (400 MHz, $CDCl_3$) δ 7.71 – 7.62 (m, 1H), 7.53 – 7.38 (m, 2H), 7.33 – 7.24 (m, 3H), 7.20 – 7.07 (m, 3H), 4.16 (d, J = 1.0 Hz, 2H), 2.33 (s, 3H);

^{13}C NMR (101 MHz, $CDCl_3$) δ 155.5, 142.8, 138.5, 135.3, 130.2, 129.6, 127.2, 126.5, 124.6, 122.6, 119.9, 116.5, 111.6, 27.4, 20.4;

HRMS (APCI, m/z) calcd. for $C_{16}H_{14}OSH^+$: 255.0838, found: 255.0839.

3-(((2-methoxyphenyl)thio)methyl)benzofuran 5b:

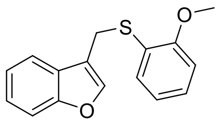

The reaction was performed with 2-methoxybenzenethiol (14.02 mg, 0.10 mmol) and **1a** (38.71 mg, 0.15 mmol). The crude product was purified by flash chromatography on silica gel (eluted with PE / EA = 300:1) to give the product **5b** (26.22 mg, 97% yield) as a yellow oil;

^{1}H NMR (400 MHz, $CDCl_3$) δ 7.69 – 7.61 (m, 1H), 7.45 – 7.39 (m, 2H), 7.30 – 7.16 (m, 4H), 6.87 – 6.80 (m, 2H), 4.16 (d, J = 1.0 Hz, 2H), 3.84 (s, 3H);

^{13}C NMR (101 MHz, $CDCl_3$) δ 158.1, 155.5, 142.8, 131.5, 128.3, 127.3, 124.5, 123.7, 122.6, 121.1, 120.1, 116.8, 111.5, 110.7, 55.8, 26.5;

HRMS (ESI, m/z) calcd. for $C_{16}H_{14}O_2SNa^+$: 293.0607, found: 293.0605.

3-(((2-fluorophenyl)thio)methyl)benzofuran 5c:

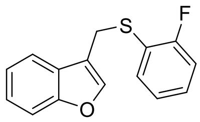

The reaction was performed with 2-fluorobenzenethiol (12.82 mg, 0.10 mmol) and **1a** (38.71 mg, 0.15 mmol). The crude product was purified by flash chromatography on silica gel (eluted with PE / EA = 300:1) to give the product **5c** (13,43 mg, 52% yield) as a yellow oil;

^{1}H NMR (400 MHz, $CDCl_3$) δ 7.75 – 7.61 (m, 1H), 7.51 – 7.36 (m, 2H), 7.37 – 7.15 (m, 4H), 7.13 – 6.94 (m, 2H), 4.19 (d, *J* = 1.0 Hz, 2H);

^{13}C NMR (101 MHz, $CDCl_3$) δ 162.0 (d, *J* = 245.7 Hz), 155.5, 142.8, 133.7 (d, *J* = 2.0 Hz), 129.3 (d, *J* = 7.8 Hz), 127.0, 124.6, 124.4 (d, *J* = 3.7 Hz), 122.6, 122.2 (d, *J* = 17.6 Hz), 120.0, 116.4, 115.8 (d, *J* = 22.5 Hz), 111.6, 27.6 (d, *J* = 3.2 Hz);

^{19}F NMR (377 MHz, CDCl3) δ -108.8.

HRMS (APCI, m/z) calcd. for $C_{15}H_{11}FOSH^+$: 259.0587, found: 259.0599.

3-(((2-chlorophenyl)thio)methyl)benzofuran 5d:

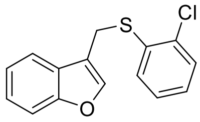

The reaction was performed with 2-chlorobenzenethiol (14.46 mg, 0.10 mmol) and **1a** (38.71 mg, 0.15 mmol). The crude product was purified by flash chromatography on silica gel (eluted with PE / EA = 300:1) to give the product **5d** (14.84 mg, 54% yield) as a yellow oil;

^{1}H NMR (400 MHz, $CDCl_3$) δ 7.72 – 7.64 (m, 1H), 7.52 – 7.42 (m, 2H), 7.42 – 7.35 (m, 1H), 7.34 – 7.23 (m, 3H), 7.18 – 7.09 (m, 2H), 4.23 (d, *J* = 1.0 Hz, 2H);

^{13}C NMR (101 MHz, $CDCl_3$) δ 155.5, 143.0, 135.0, 134.6, 130.4, 129.8, 127.5, 127.2, 127.1, 124.7, 122.7, 119.9, 115.9, 111.6, 26.8;

HRMS (ESI, m/z) calcd. for $C_{15}H_{11}ClOSNa^+$: 297.0111, found: 297.0052.

3-(((2-bromophenyl)thio)methyl)benzofuran 5e:

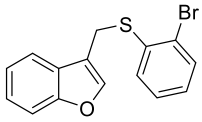

The reaction was performed with 2-bromobenzenethiol (18.91 mg, 0.10 mmol) and **1a** (38.71 mg, 0.15 mmol). The crude product was purified by flash chromatography on silica gel (eluted with PE / EA = 300:1) to give the product **5e** (20.11 mg, 63% yield, m.p. 81 – 82 ^{o}C) as a white solid;

^{1}H NMR (400 MHz, $CDCl_3$) δ 7.72 – 7.64 (m, 1H), 7.55 (dd, *J* = 8.0, 1.4 Hz, 1H), 7.50 (d, *J* = 1.2 Hz, 1H), 7.48 – 7.43 (m, 1H), 7.34 – 7.22 (m, 3H), 7.19 (td, *J* = 7.6, 1.4 Hz, 1H), 7.03 (td, *J* = 7.6, 1.8 Hz, 1H), 4.22 (d, *J* = 1.0 Hz, 2H);

^{13}C NMR (101 MHz, $CDCl_3$) δ 155.5, 143.1, 137.2, 133.1, 129.9, 127.8, 127.5, 127.1, 124.7, 122.7, 120.0, 116.8, 115.8, 111.6, 27.2;

HRMS (ESI, m/z) calcd. for $C_{15}H_{11}BrOSH^+$: 318.9787, found: 318.9788.

3-((*m*-tolylthio)methyl)benzofuran 5f:

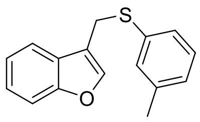

The reaction was performed with 3-methylbenzenethiol (12.42 mg, 0.10 mmol) and **1a** (38.71 mg, 0.15 mmol). The crude product was purified by flash chromatography on silica gel (eluted with PE / EA = 300:1) to give the product **5f** (23.64 mg, 93% yield) as a yellow oil;

^{1}H NMR (400 MHz, $CDCl_3$) δ 7.68 – 7.58 (m, 1H), 7.52 – 7.38 (m, 2H), 7.32 – 7.19 (m, 2H), 7.17 – 7.10 (m, 3H), 7.02 – 6.94 (m, 1H), 4.17 (d, *J* = 1.0 Hz, 2H), 2.28 (s, 3H);

^{13}C NMR (101 MHz, $CDCl_3$) δ 155.5, 142.8, 138.8, 135.7, 131.0, 128.8, 127.6, 127.2, 127.2, 124.6, 122.6, 120.0, 116.8, 111.6, 28.2, 21.3;

HRMS (APCI, m/z) calcd. for $C_{16}H_{14}OSH^+$: 255.0838, found: 255.0834.

3-(((3-methoxyphenyl)thio)methyl)benzofuran 5g:

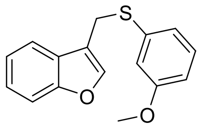

The reaction was performed with 3-methoxybenzenethiol (14.02 mg, 0.10 mmol) and **1a** (38.71 mg, 0.15 mmol). The crude product was purified by flash chromatography on silica gel (eluted with PE / EA = 300:1) to give the product **5g** (25.68 mg, 95% yield) as a yellow oil;

^{1}H NMR (400 MHz, $CDCl_3$) δ 7.66 – 7.61 (m, 1H), 7.45 (dt, *J* = 8.0, 0.8 Hz, 2H), 7.32 – 7.21 (m, 2H), 7.19 – 7.14 (m, 1H), 6.92 (ddd, J = 7.8, 1.6, 1.0 Hz, 1H), 6.85 (dd, *J* = 2.6, 1.8 Hz, 1H), 6.73 (ddd, *J* = 8.2, 2.6, 0.8 Hz, 1H), 4.18 (d, *J* = 1.0 Hz, 2H), 3.70 (s, 3H);

^{13}C NMR (101 MHz, $CDCl_3$) δ 159.8, 155.5, 142.9, 137.3, 129.8, 127.1, 124.6, 122.7, 122.3, 120.0, 116.7, 115.5, 112.5, 111.6, 55.3, 28.0;

HRMS (APCI, m/z) calcd. for $C_{16}H_{14}O_2SH^+$: 271.0787, found: 271.0783.

3-(((3-chlorophenyl)thio)methyl)benzofuran 5h:

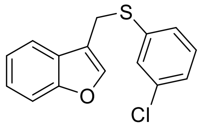

The reaction was performed with 3-chlorobenzenethiol (14.46 mg, 0.10 mmol) and **1a** (38.71 mg, 0.15 mmol). The crude product was purified by flash chromatography on silica gel (eluted with PE / EA = 300:1) to give the product **5h** (11.82 mg, 43% yield) as a yellow oil;

^{1}H NMR (400 MHz, $CDCl_3$) δ 7.70 – 7.60 (m, 1H), 7.47 (t, *J* = 4.0 Hz, 2H), 7.36 – 7.24 (m, 3H), 7.17 (d, *J* = 1.4 Hz, 3H), 4.20 (d, *J* = 1.0 Hz, 2H);

^{13}C NMR (101 MHz, $CDCl_3$) δ 155.5, 142.9, 138.1, 134.7, 129.9, 129.4, 127.9, 126.9, 126.7, 124.7, 122.7, 119.9, 116.1, 111.7, 28.0;

HRMS (ESI, m/z) calcd. for $C_{15}H_{11}ClOSK^+$: 312.9851, found: 312.9868.

3-(((4-methoxyphenyl)thio)methyl)benzofuran 5i:

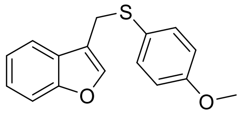

The reaction was performed with 4-methoxybenzenethiol (14.02 mg, 0.10 mmol) and **1a** (38.71 mg, 0.15 mmol). The crude product was purified by flash chromatography on silica gel (eluted with PE / EA = 300:1) to give the product **5i** (17.30 mg, 64% yield) as a yellow oil;

^{1}H NMR (400 MHz, $CDCl_3$) δ 7.67 – 7.60 (m, 1H), 7.45 (dt, *J* = 8.2, 0.8 Hz, 1H), 7.32 – 7.22 (m, 5H), 6.82 – 6.73 (m, 2H), 4.05 (d, *J* = 1.0 Hz, 2H), 3.76 (s, 3H);

^{13}C NMR (101 MHz, $CDCl_3$) δ 159.5, 155.5, 142.7, 134.6, 127.1, 125.8, 124.5, 122.6, 120.1, 117.0, 114.5, 111.5, 55.3, 30.3;

HRMS (APCI, m/z) calcd. for $C_{16}H_{14}O_2SH^+$: 271.0787, found: 271.0782.

3-(((4-(*tert*-butyl)phenyl)thio)methyl)benzofuran 5j:

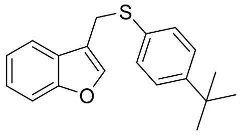

The reaction was performed with 4-(*tert*-butyl)benzenethiol (16.63 mg, 0.10 mmol) and **1a** (38.71 mg, 0.15 mmol). The crude product was purified by flash chromatography on silica gel (eluted with PE / EA = 300:1) to give the product **5j** (19.86 mg, 67% yield) as a yellow oil;

^{1}H NMR (400 MHz, $CDCl_3$) δ 7.62 (ddd, *J* = 7.6, 1.6, 0.8 Hz, 1H), 7.48 – 7.41 (m, 2H), 7.33 – 7.21 (m, 6H), 4.16 (d, *J* = 1.0 Hz, 2H), 1.29 (s, 9H);

^{13}**C NMR** (101 MHz, $CDCl_3$) δ 155.5, 150.1, 142.8, 132.3, 130.6, 127.2, 126.0, 124.5, 122.6, 120.0, 117.0, 111.5, 34.5, 31.3, 28.6;

HRMS (APCI, m/z) calcd. for $C_{19}H_{20}OSH^+$: 297.1308, found: 297.1304.

3-(((2,4-dimethylphenyl)thio)methyl)benzofuran 5k:

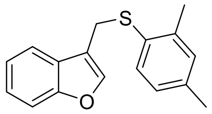

The reaction was performed with 2,4-dimethylbenzenethiol (13.82 mg, 0.10 mmol) and **1a** (38.71 mg, 0.15 mmol). The crude product was purified by flash chromatography on silica gel (eluted with PE / EA = 300:1) to give the product **5k** (19.05 mg, 71% yield) as a yellow oil;

^{1}**H NMR** (400 MHz, $CDCl_3$) δ 7.68 – 7.62 (m, 1H), 7.44 (dt, *J* = 8.2, 0.8 Hz, 1H), 7.36 (d, *J* = 1.0 Hz, 1H), 7.32 – 7.16 (m, 3H), 7.01 – 6.97 (m, 1H), 6.95 – 6.89 (m, 1H), 4.09 (d, *J* = 1.0 Hz, 2H), 2.29 (s, 3H), 2.27 (s, 3H);

^{13}**C NMR** (101 MHz, $CDCl_3$) δ 155.5, 142.7, 139.1, 136.9, 131.4, 131.2, 131.2, 131.1, 127.3, 124.5, 122.6, 120.0, 116.8, 111.6, 28.1, 21.0, 20.4;

HRMS (APCI, m/z) calcd. for $C_{17}H_{16}OSH^+$: 269.0995, found: 269.0994.

3-((naphthalen-1-ylthio)methyl)benzofuran 5l:

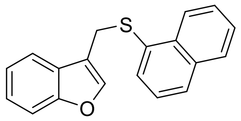

The reaction was performed with naphthalene-1-thiol (16.02 mg, 0.10 mmol) and **1a** (38.71 mg, 0.15 mmol). The crude product was purified by flash chromatography on silica gel (eluted with PE / EA = 300:1) to give the product **5l** (19.46 mg, 67% yield) as a yellow oil;

^{1}**H NMR** (400 MHz, $CDCl_3$) δ 8.45 – 8.38 (m, 1H), 7.86 – 7.78 (m, 1H), 7.74 (dt, *J* = 8.4, 1.0 Hz, 1H), 7.68 – 7.61 (m, 1H), 7.54 – 7.46 (m, 3H), 7.46 – 7.39 (m, 1H), 7.36 – 7.18 (m, 4H), 4.20 (d, *J* = 1.0 Hz, 2H);

^{13}**C NMR** (101 MHz, $CDCl_3$) δ 155.5, 142.8, 134.0, 133.3, 132.9, 130.3, 128.7, 128.2, 127.2, 126.6, 126.3, 125.6, 125.2, 124.6, 122.6, 120.0, 116.6, 111.6, 28.6;

HRMS (APCI, m/z) calcd. for $C_{19}H_{14}OSH^+$: 291.0838, found: 291.0837.

4-((benzofuran-3-ylmethyl)thio)pyridine 5m:

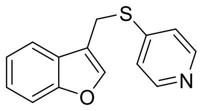

The reaction was performed with pyridine-4-thiol (11.12 mg, 0.10 mmol) and **1a** (38.71 mg, 0.15 mmol). The crude product was purified by flash chromatography on silica gel (eluted with PE / EA = 300:1) to give the product **5m** (19.06 mg, 79% yield, m.p. 88 – 89 ^{o}C) as a yellow solid;

^{1}H NMR (400 MHz, $CDCl_3$) δ 8.43 – 8.35 (m, 2H), 7.70 – 7.63 (m, 1H), 7.62 – 7.57 (m, 1H), 7.49 (dt, *J* = 8.4, 1.0 Hz, 1H), 7.37 – 7.24 (m, 2H), 7.18 – 7.13 (m, 2H), 4.30 (d, *J* = 1.0 Hz, 2H);

^{13}C NMR (101 MHz, $CDCl_3$) δ 155.6, 149.4, 148.5, 143.1, 126.8, 124.9, 122.9, 121.0, 119.7, 115.2, 111.8, 25.0;

HRMS (ESI, m/z) calcd. for $C_{14}H_{11}NOSH^{+}$: 242.0634, found: 242.0637.

3-(((2-methylfuran-3-yl)thio)methyl)benzofuran 5n:

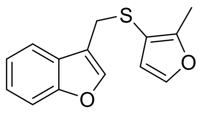

The reaction was performed with 2-methylfuran-3-thiol (11.42 mg, 0.10 mmol) and **1a** (38.71 mg, 0.15 mmol). The crude product was purified by flash chromatography on silica gel (eluted with PE / EA = 300:1) to give the product **5n** (12.95 mg, 53% yield) as a yellow oil;

^{1}H NMR (400 MHz, $CDCl_3$) δ 7.69 – 7.56 (m, 1H), 7.45 (dt, *J* = 8.4, 0.8 Hz, 1H), 7.36 – 7.16 (m, 4H), 6.23 (d, *J* = 2.0 Hz, 1H), 3.86 (d, *J* = 1.0 Hz, 2H), 1.96 (s, 3H);

^{13}C NMR (101 MHz, $CDCl_3$) δ 156.4, 155.5, 142.5, 140.6, 127.1, 124.5, 122.5, 120.0, 117.1, 115.1, 111.5, 109.6, 29.6, 11.4;

HRMS (APCI, m/z) calcd. for $C_{14}H_{12}O_2SH^{+}$: 245.0631, found: 245.0630.

3-((thiophen-2-ylthio)methyl)benzofuran 5o:

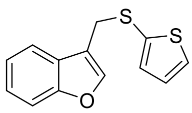

The reaction was performed with thiophene-2-thiol (11.62 mg, 0.10 mmol) and **1a** (38.71 mg, 0.15 mmol). The crude product was purified by flash chromatography on silica gel (eluted with PE / EA = 300:1) to give the product **5o** (20.19 mg, 82% yield) as a yellow oil;

^{1}H NMR (400 MHz, $CDCl_3$) δ 7.62 (dd, *J* = 7.5, 1.5 Hz, 1H), 7.49 – 7.40 (m, 1H), 7.35 – 7.17 (m, 4H), 6.95 – 6.85 (m, 2H), 4.04 (d, *J* = 0.9 Hz, 2H);

^{13}C NMR (101 MHz, $CDCl_3$) δ 155.5, 143.0, 134.9, 133.4, 130.1, 127.6, 126.9, 124.6, 122.7, 120.0, 116.6, 111.6, 32.8;

HRMS (APCI, m/z) calcd. for $C_{13}H_{10}OS_2H^+$: 247.0246, found: 247.0241.

3-((benzylthio)methyl)benzofuran 5p:

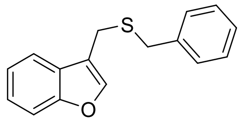

The reaction was performed with phenylmethanethiol (12.42 mg, 0.10 mmol) and **1a** (38.71 mg, 0.15 mmol). The crude product was purified by flash chromatography on silica gel (eluted with PE / EA = 300:1) to give the product **5p** (15.51 mg, 61% yield) as a yellow oil;

^{1}H NMR (400 MHz, $CDCl_3$) δ 7.65 – 7.60 (m, 1H), 7.49 – 7.43 (m, 2H), 7.34 – 7.21 (m, 7H), 3.67 (d, *J* = 1.0 Hz, 2H), 3.64 (s, 2H);

^{13}C NMR (101 MHz, $CDCl_3$) δ 155.7, 142.7, 137.9, 129.1, 128.6, 127.2, 127.1, 124.6, 122.6, 120.2, 116.8, 111.6, 35.7, 24.2;

HRMS (APCI, m/z) calcd. for $C_{16}H_{14}OSH^+$: 255.0838, found: 255.0835.

3-(((3-methylbenzyl)thio)methyl)benzofuran 5q:

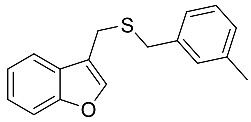

The reaction was performed with *m*-tolylmethanethiol (13.82 mg, 0.10 mmol) and **1a** (38.71 mg, 0.15 mmol). The crude product was purified by flash chromatography on silica gel (eluted with PE / EA = 300:1) to give the product **5q** (16.11 mg, 60% yield) as a yellow oil;

^{1}H NMR (400 MHz, $CDCl_3$) δ 7.65 – 7.59 (m, 1H), 7.47 (dt, *J* = 9.0, 1.0 Hz, 2H), 7.34 – 7.15 (m, 3H), 7.12 – 7.02 (m, 3H), 3.68 (d, *J* = 1.0 Hz, 2H), 3.61 (s, 2H), 2.32 (d, *J* = 0.8 Hz, 3H);

^{13}C NMR (101 MHz, $CDCl_3$) δ 155.7, 142.7, 138.2, 137.8, 129.8, 128.4, 127.9, 127.3, 126.1, 124.6, 122.6, 120.3, 116.9, 111.6, 35.7, 24.2, 21.4;

HRMS (APCI, m/z) calcd. for $C_{17}H_{16}OSH^+$: 269.0995, found: 269.0993.

3-((phenethylthio)methyl)benzofuran 5r:

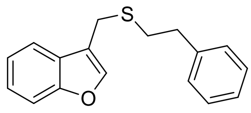

The reaction was performed with 2-phenylethane-1-thiol (13.82 mg, 0.10 mmol) and **1a** (38.71 mg, 0.15 mmol). The crude product was purified by flash chromatography on silica gel (eluted with PE / EA = 300:1) to give the product **5r** (26.30 mg, 98% yield) as a yellow oil;

^{1}H NMR (400 MHz, $CDCl_3$) δ 7.71 – 7.63 (m, 1H), 7.52 – 7.40 (m, 2H), 7.33 – 7.09 (m, 7H), 3.76 (d, *J* = 1.0 Hz, 2H), 2.84 (dd, *J* = 9.0, 6.4 Hz, 2H), 2.73 – 2.64 (m, 2H);

^{13}C NMR (101 MHz, $CDCl_3$) δ 155.7, 142.5, 140.5, 128.6, 128.6, 127.2, 126.5, 124.6, 122.6, 120.3, 117.1, 111.6, 36.1, 33.0, 25.2;

HRMS (ESI, m/z) calcd. for $C_{17}H_{16}OSNa^+$: 291.0814, found: 291.0807.

3-((allylthio)methyl)benzofuran 5s:

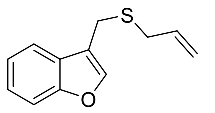

The reaction was performed with prop-2-ene-1-thiol (7.41 mg, 0.10 mmol) and **1a** (38.71 mg, 0.15 mmol). The crude product was purified by flash chromatography on silica gel (eluted with PE / EA = 300:1) to give the product **5s** (19.61 mg, 96% yield) as a yellow oil;

^{1}H NMR (400 MHz, $CDCl_3$) δ 7.75 – 7.65 (m, 1H), 7.51 (d, *J* = 1.0 Hz, 1H), 7.49 – 7.44 (m, 1H), 7.35 – 7.20 (m, 2H), 5.80 (ddt, *J* = 17.0, 10.0, 7.2 Hz, 1H), 5.20 – 5.02 (m, 2H), 3.75 (d, *J* = 1.0 Hz, 2H), 3.08 (dt, *J* = 7.0, 1.2 Hz, 2H);

^{13}C NMR (101 MHz, $CDCl_3$) δ 155.7, 142.6, 134.1, 127.2, 124.6, 122.6, 120.2, 117.5, 116.9, 111.6, 34.2, 23.5;

HRMS (APCI, m/z) calcd. for $C_{12}H_{12}OSH^+$: 205.0682, found: 205.0680.

3-((cyclohexylthio)methyl)benzofuran 5t:

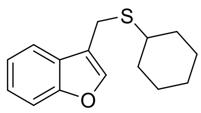

The reaction was performed with cyclohexanethiol (11.62 mg, 0.10 mmol) and **1a** (38.71 mg, 0.15 mmol). The crude product was purified by flash chromatography on silica gel (eluted with PE / EA = 300:1) to give the product **5t** (22.42 mg, 91% yield) as a yellow oil;

^{1}H NMR (400 MHz, $CDCl_3$) δ 7.71 – 7.65 (m, 1H), 7.53 (d, *J* = 1.2 Hz, 1H), 7.46 (dt, *J* = 8.4, 0.8 Hz, 1H), 7.33 – 7.20 (m, 2H), 3.83 (d, *J* = 1.0 Hz, 2H), 2.62 (tt, *J* = 10.6, 3.6 Hz, 1H), 2.02 – 1.90 (m, 2H), 1.75 (ddt, *J* = 9.8, 7.6, 3.2 Hz, 2H), 1.62 – 1.54 (m, 1H), 1.41 – 1.29 (m, 2H), 1.25 (dddd, *J* = 9.0, 7.2, 3.2, 1.2 Hz, 3H);

^{13}C NMR (101 MHz, $CDCl_3$) δ 153.8, 140.5, 125.5, 122.6, 120.7, 118.4, 115.9, 109.7, 41.4, 31.5, 24.2, 24.0, 21.4;

HRMS (APCI, m/z) calcd. for $C_{15}H_{18}OSH^+$: 247.1151, found: 247.1150.

3-((*tert*-butylthio)methyl)benzofuran 5u:

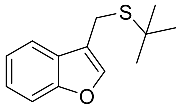

The reaction was performed with 2-methylpropane-2-thiol (9.02 mg, 0.10 mmol) and **1a** (38.71 mg, 0.15 mmol). The crude product was purified by flash chromatography on silica gel (eluted with PE / EA = 300:1) to give the product **5u** (19.39 mg, 88% yield) as a yellow oil;

^{1}H NMR (400 MHz, $CDCl_3$) δ 7.70 – 7.65 (m, 1H), 7.57 (t, *J* = 1.0 Hz, 1H), 7.47 – 7.43 (m, 1H), 7.32 – 7.22 (m, 2H), 3.86 (d, *J* = 1.0 Hz, 2H), 1.40 (s, 9H);

^{13}C NMR (101 MHz, $CDCl_3$) δ 155.5, 142.4, 127.5, 124.4, 122.5, 120.0, 117.5, 111.5, 42.8, 30.8, 21.9;

HRMS (APCI, m/z) calcd. for $C_{13}H_{16}OSH^+$: 221.0995, found: 221.0990.

3-((isobutylthio)methyl)benzofuran 5v:

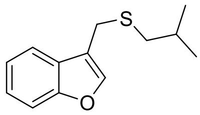

The reaction was performed with 2-methylpropane-1-thiol (9.02 mg, 0.10 mmol) and **1a** (38.71 mg, 0.15 mmol). The crude product was purified by flash chromatography on silica gel (eluted with PE / EA = 300:1) to give the product **5v** (17.62 mg, 80% yield) as a yellow oil;

^{1}H NMR (400 MHz, $CDCl_3$) δ 7.72 – 7.64 (m, 1H), 7.53 (d, *J* = 1.0 Hz, 1H), 7.46 (dt, *J* = 8.4, 0.8 Hz, 1H), 7.33 – 7.21 (m, 2H), 3.78 (d, *J* = 1.0 Hz, 2H), 2.35 (d, *J* = 6.8 Hz, 2H), 1.79 (dp, *J* = 13.4, 6.8 Hz, 1H), 0.96 (d, *J* = 6.8 Hz, 6H);

^{13}C NMR (101 MHz, $CDCl_3$) δ 155.7, 142.4, 127.3, 124.5, 122.5, 120.2, 117.4, 111.5, 40.9, 28.3, 25.5, 22.1;

HRMS (APCI, m/z) calcd. for $C_{13}H_{16}OSH^+$: 221.0995, found: 221.0987.

3-((propylthio)methyl)benzofuran 5w:

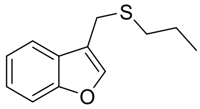

The reaction was performed with propane-1-thiol (7.62 mg, 0.10 mmol) and **1a** (38.71 mg, 0.15 mmol). The crude product was purified by flash chromatography on silica gel (eluted with PE / EA = 300:1) to give the product **5w** (15.06 mg, 73% yield) as a yellow oil;

^{1}H NMR (400 MHz, $CDCl_3$) δ 7.73 – 7.65 (m, 1H), 7.57 – 7.51 (m, 1H), 7.50 – 7.43 (m, 1H), 7.33 – 7.23 (m, 2H), 3.80 (d, *J* = 1.0 Hz, 2H), 2.50 – 2.37 (m, 2H), 1.60 (h, *J* = 7.4 Hz, 2H), 0.96 (t, *J* = 7.4 Hz, 3H);

^{13}C NMR (101 MHz, $CDCl_3$) δ 155.6, 142.4, 127.2, 124.5, 122.5, 120.2, 117.3, 111.5, 33.7, 24.9, 22.5, 13.5;

HRMS (APCI, m/z) calcd. for $C_{12}H_{14}OSH^+$: 207.0838, found: 207.0836.

3-((dodecylthio)methyl)benzofuran 5x:

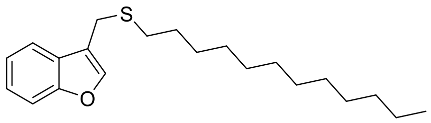

The reaction was performed with dodecane-1-thiol (20.24 mg, 0.10 mmol) and **1a** (38.71 mg, 0.15 mmol). The crude product was purified by flash chromatography on silica gel (eluted with PE / EA = 300:1) to give the product **5x** (32.59 mg, 98% yield) as a yellow oil;

^{1}H NMR (400 MHz, $CDCl_3$) δ 7.53 (dd, *J* = 7.4, 1.6 Hz, 1H), 7.37 (s, 1H), 7.30 (dd, *J* = 7.6, 1.2 Hz, 1H), 7.17 – 7.03 (m, 2H), 3.64 (s, 2H), 2.34 – 2.23 (m, 2H), 1.41 (q, *J* = 7.0 Hz, 2H), 1.13 – 1.03 (m, 18H), 0.72 (t, *J* = 6.8 Hz, 3H);

^{13}C NMR (101 MHz, $CDCl_3$) δ 155.7, 142.4, 127.3, 124.5, 122.5, 120.2, 117.3, 111.5, 32.0, 31.7, 29.7, 29.7, 29.6, 29.5, 29.4, 29.3, 29.2, 28.9, 25.0, 22.7, 14.2;

HRMS (APCI, m/z) calcd. for $C_{21}H_{32}OSH^+$: 333.2247, found: 333.2241.

3-((octadecylthio)methyl)benzofuran 5y:

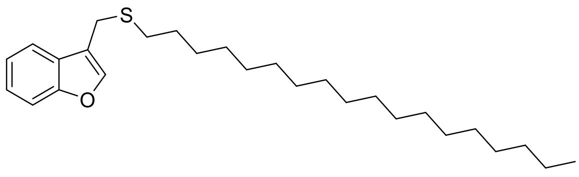

The reaction was performed with octadecane-1-thiol (28.66 mg, 0.10 mmol) and **1a** (38.71 mg, 0.15 mmol). The crude product was purified by flash chromatography on silica gel (eluted with PE / EA = 300:1) to give the product **5y** (40.84 mg, 98% yield, m.p. 51 – 52 ^{o}C) as a white solid;

^{1}H NMR (400 MHz, $CDCl_3$) δ 7.59 – 7.47 (m, 1H), 7.37 (s, 1H), 7.30 (d, *J* = 8.0 Hz, 1H), 7.17 – 7.04 (m, 2H), 3.63 (s, 2H), 2.29 (t, *J* = 7.4 Hz, 2H), 1.45 – 1.37 (m, 2H), 1.09 (d, *J* = 7.4 Hz, 30H), 0.72 (t, *J* = 6.6 Hz, 3H);

^{13}C NMR (101 MHz, $CDCl_3$) δ 155.7, 142.4, 127.3, 124.5, 122.5, 120.2, 117.3, 111.5, 32.0, 31.7, 29.8, 29.8, 29.8, 29.7, 29.7, 29.7, 29.7, 29.6, 29.6, 29.6, 29.4, 29.3, 29.2, 28.9, 25.0, 22.7, 14.2;

HRMS (ESI, m/z) calcd. for $C_{27}H_{44}OSH^+$: 417.3187, found: 417.3192.

***N*-(benzofuran-3-ylmethyl)aniline 7a:**

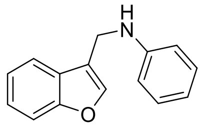

The reaction was performed with **1a** (25.81 mg, 0.10 mmol) and benzenethiol (9.31 mg, 0.10 mmol). The crude product was purified by flash chromatography on silica gel (eluted with PE / EA = 300:1) to give the product **7a** (10.72 mg, 48% yield) as a yellow oil;

^{1}H NMR (400 MHz, $CDCl_3$) δ 7.59 (dt, *J* = 7.6, 1.0 Hz, 1H), 7.55 (d, *J* = 1.2 Hz, 1H), 7.48 (dt, *J* = 8.2, 0.8 Hz, 1H), 7.30 (ddd, *J* = 8.4, 7.2, 1.4 Hz, 1H), 7.26 – 7.15 (m, 3H), 6.74 (tt, *J* = 7.2, 1.0 Hz, 1H), 6.71 – 6.64 (m, 2H), 4.39 (d, *J* = 1.2 Hz, 2H), 3.87 (s, 1H);

^{13}C NMR (101 MHz, $CDCl_3$) δ 155.6, 148.1, 142.6, 129.4, 127.1, 124.6, 122.7, 119.8, 118.5, 118.0, 113.0, 111.7, 38.7;

HRMS (ESI, m/z) calcd. for $C_{15}H_{13}NOH^+$: 224.1070, found: 224.1059.

***N*-(benzofuran-3-ylmethyl)-4-methoxyaniline 7b:**

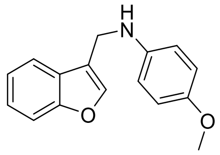

The reaction was performed with **1a** (25.81 mg, 0.10 mmol) and 4-methoxyaniline (12.32 mg, 0.10 mmol). The crude product was purified by flash chromatography on silica gel (eluted with PE / EA = 300:1) to give the product **7b** (12.67 mg, 50% yield) as a yellow oil;

^{1}H NMR (400 MHz, $CDCl_3$) δ 7.62 (ddd, *J* = 7.6, 1.4, 0.6 Hz, 1H), 7.58 (t, *J* = 1.2 Hz, 1H), 7.49 (dt, *J* = 8.2, 0.8 Hz, 1H), 7.31 (ddd, *J* = 8.2, 7.2, 1.4 Hz, 1H), 7.25 (td, *J* = 7.4, 1.0 Hz, 1H), 6.85 – 6.76 (m, 2H), 6.71 – 6.58 (m, 2H), 4.38 (d, *J* = 1.0 Hz, 2H), 3.75 (s, 3H);

^{13}C NMR (101 MHz, $CDCl_3$) δ 155.6, 152.5, 142.5, 142.3, 127.1, 124.6, 122.7, 119.9, 118.7, 115.0, 114.4, 111.6, 55.8, 39.6;

HRMS (APCI, m/z) calcd. for $C_{16}H_{15}NO_2H^+$: 254.1176, found: 254.1170.

***N*-(benzofuran-3-ylmethyl)-3-methylaniline 7c:**

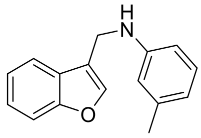

The reaction was performed with **1a** (25.81 mg, 0.10 mmol) and *m*-toluidine (10.72 mg, 0.10 mmol). The crude product was purified by flash chromatography on silica gel (eluted with PE / EA = 300:1) to give the product

7c (11.42 mg, 48% yield) as a yellow oil;

^{1}H NMR (400 MHz, $CDCl_3$) δ 7.65 – 7.56 (m, 2H), 7.49 (dt, *J* = 8.2, 0.8 Hz, 1H), 7.31 (ddd, *J* = 8.2, 7.2, 1.4 Hz, 1H), 7.28 – 7.22 (m, 1H), 7.09 (td, *J* = 7.4, 1.2 Hz, 1H), 6.61 – 6.55 (m, 1H), 6.51 (d, *J* = 7.2 Hz, 2H), 4.41 (d, *J* = 1.2 Hz, 2H), 3.84 (s, 1H), 2.29 (s, 3H);

^{13}C NMR (101 MHz, $CDCl_3$) δ 155.6, 148.1, 142.5, 139.2, 129.3, 127.1, 124.6, 122.7, 119.9, 118.9, 118.6, 113.8, 111.7, 110.2, 38.7, 21.7;

HRMS (APCI, m/z) calcd. for $C_{16}H_{15}NOH^+$: 238.1226, found: 238.1220.

***N*-(benzofuran-3-ylmethyl)-3-(trifluoromethyl)aniline 7d:**

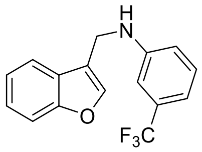

The reaction was performed with **1a** (25.81 mg, 0.10 mmol) and 3-(trifluoromethyl)aniline (16.11 mg, 0.10 mmol). The crude product was purified by flash chromatography on silica gel (eluted with PE / EA = 300:1) to give the product **7d** (12.01 mg, 41% yield) as a yellow oil;

^{1}H NMR (400 MHz, $CDCl_3$) δ 7.60 (dt, *J* = 6.8, 1.0 Hz, 2H), 7.51 (dd, *J* = 8.0, 1.0 Hz, 1H), 7.33 (ddd, *J* = 8.2, 7.2, 1.4 Hz, 1H), 7.28 – 7.24 (m, 1H), 7.10 (t, *J* = 8.0 Hz, 1H), 6.78 – 6.63 (m, 2H), 6.55 (ddd, *J* = 8.2, 2.4, 0.8 Hz, 1H), 4.62 – 4.31 (m, 2H), 4.01 (s, 1H);

^{13}C NMR (126 MHz, $CDCl_3$) 155.7, 148.2, 142.7, 131.7 (q, *J* = 31.8 Hz), 129.8, 126.9, 124.9, 124.4 (q, *J* = 272.4 Hz), 122.9, 119.8, 117.8, 116.0, 114.4 (q, *J* = 4.0 Hz), 111.9, 109.2 (q, *J* = 3.9 Hz), 38.5;

^{19}F NMR (377 MHz, CDC13) δ -62.8;

HRMS (ESI, m/z) calcd. for $C_{16}H_{12}F_3NOH^+$: 292.0944, found: 292.0932.

***N*-(benzofuran-3-ylmethyl)-3-chloroaniline 7e:**

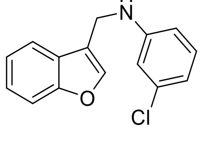

The reaction was performed with **1a** (25.81 mg, 0.10 mmol) and 3-chloroaniline (12.76 mg, 0.10 mmol). The crude product was purified by flash chromatography on silica gel (eluted with PE / EA = 300:1) to give the product **7e** (11.06 mg, 43% yield) as a yellow oil;

^{1}H NMR (400 MHz, $CDCl_3$) δ 7.60 (dt, *J* = 7.8, 1.0 Hz, 2H), 7.50 (dt, *J* = 8.2, 0.8 Hz, 1H), 7.33 (ddd, *J* = 8.2, 7.2, 1.4 Hz, 1H), 7.26 (td, *J* = 7.4, 1.0 Hz, 1H), 7.10 (t, *J* = 8.0 Hz, 1H), 6.75 – 6.64 (m, 2H), 6.55 (ddd, *J* = 8.2, 2.2, 0.8 Hz, 1H), 4.41 (d, *J* = 1.0 Hz, 2H), 4.00 (s, 1H);

^{13}C NMR (101 MHz, $CDCl_3$) δ 155.6, 149.1, 142.6, 135.1, 130.3, 126.9, 124.8, 122.8, 119.7, 117.9, 117.8, 112.6, 111.7, 111.3, 38.5;

HRMS (APCI, m/z) calcd. for $C_{15}H_{12}ClNOH^+$: 258.0680, found: 258.0675.

***N*-(benzofuran-3-ylmethyl)-2-bromoaniline 7f:**

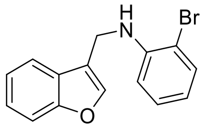

The reaction was performed with **1a** (25.81 mg, 0.10 mmol) and 2-bromoaniline (17.20 mg, 0.10 mmol). The crude product was purified by flash chromatography on silica gel (eluted with PE / EA = 300:1) to give the product **7f** (8.79 mg, 29% yield) as a yellow oil;

^{1}H NMR (400 MHz, $CDCl_3$) δ 7.67 – 7.61 (m, 1H), 7.61 – 7.59 (m, 1H), 7.50 (dt, *J* = 8.2, 0.8 Hz, 1H), 7.45 (dd, *J* = 7.8, 1.4 Hz, 1H), 7.33 (ddd, *J* = 8.2, 7.2, 1.4 Hz, 1H), 7.30 – 7.23 (m, 1H), 7.19 (ddd, *J* = 8.0, 7.2, 1.4 Hz, 1H), 6.76 (dd, *J* = 8.0, 1.4 Hz, 1H), 6.61 (td, *J* = 7.6, 1.4 Hz, 1H), 4.65 (s, 1H), 4.55 – 4.44 (m, 2H);

^{13}C NMR (101 MHz, $CDCl_3$) δ 155.7, 144.7, 142.6, 132.5, 128.6, 126.9, 124.7, 122.8, 119.7, 118.3, 117.9, 111.7, 111.6, 109.9, 38.7;

HRMS (APCI, m/z) calcd. for $C_{15}H_{12}BrNOH^+$: 302.0175, found: 302.0172.

***N*-(benzofuran-3-ylmethyl)-2-methylaniline7g:**

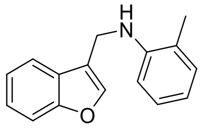

The reaction was performed with **1a** (25.81 mg, 0.10 mmol) and *o*-toluidine (10.72 mg, 0.10 mmol). The crude product was purified by flash chromatography on silica gel (eluted with PE / EA = 300:1) to give the product **7g** (4.75 mg, 20% yield) as a yellow oil;

^{1}H NMR (400 MHz, $CDCl_3$) δ 7.68 – 7.58 (m, 2H), 7.51 (dt, *J* = 8.2, 0.8 Hz, 1H), 7.33 (ddd, *J* = 8.2, 7.2, 1.4 Hz, 1H), 7.29 – 7.23 (m, 1H), 7.16 (td, *J* = 7.76, 1.6 Hz, 1H), 7.09 (ddd, *J* = 7.4, 1.8, 0.8 Hz, 1H), 6.79 – 6.66 (m, 2H), 4.48 (d, *J* = 1.0 Hz, 2H), 3.76 (s, 1H), 2.13 (s, 3H);

^{13}C NMR (101 MHz, $CDCl_3$) δ 155.7, 146.0, 142.6, 130.2, 127.2, 127.2, 124.6, 122.7, 122.3, 119.8, 118.5, 117.5, 111.7, 110.0, 38.7, 17.5;

HRMS (ESI, m/z) calcd. for $C_{16}H_{15}NOH^+$: 238.1226, found: 238.1220.

***N*-(benzofuran-3-ylmethyl)-N-methylaniline 7h:**

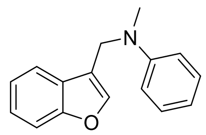

The reaction was performed with **1a** (25.81 mg, 0.10 mmol) and *N*-methylaniline (10.72 mg, 0.10 mmol). The crude product was purified by flash chromatography on silica gel (eluted with PE / EA = 300:1) to give the product **7h** (12.36 mg, 52% yield) as a yellow oil;

^{1}H NMR (400 MHz, $CDCl_3$) δ 7.55 – 7.41 (m, 3H), 7.31 – 7.18 (m, 4H), 6.90 – 6.82 (m, 2H), 6.76 (tt, *J* = 7.2, 1.0 Hz, 1H), 4.58 (d, *J* = 1.2 Hz, 2H), 2.96 (s, 3H);

^{13}C NMR (101 MHz, $CDCl_3$) δ 155.7, 149.8, 142.5, 129.3, 127.3, 124.5, 122.6, 120.0, 117.8, 117.3, 113.3, 111.6, 47.5, 38.1;

HRMS (ESI, m/z) calcd. for $C_{16}H_{15}NOH^+$: 238.1226, found: 238.1224.

***N*-(benzofuran-3-ylmethyl)-2-fluoro-*N*-methylaniline 7i:**

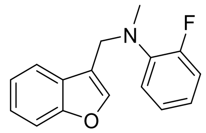

The reaction was performed with **1a** (25.81 mg, 0.10 mmol) and 2-fluoro-*N*-methylaniline (12.51 mg, 0.10 mmol). The crude product was purified by flash chromatography on silica gel (eluted with PE / EA = 300:1) to give the product **7i** (10.28 mg, 40% yield) as a yellow oil;

^{1}H NMR (400 MHz, $CDCl_3$) δ 7.58 – 7.53 (m, 1H), 7.50 (d, *J* = 1.0 Hz, 1H), 7.45 (dt, *J* = 8.2, 0.8 Hz, 1H), 7.27 (ddd, *J* = 8.2, 7.2, 1.4 Hz, 1H), 7.19 (td, *J* = 7.4, 1.0 Hz, 1H), 7.12 – 6.99 (m, 2H), 6.95 – 6.87 (m, 2H), 4.40 (d, *J* = 1.0 Hz, 2H), 2.77 (s, 3H);

^{13}C NMR (101 MHz, $CDCl_3$) δ 155.7 (d, *J* = 244.8 Hz), 155.6, 143.1, 140.0 (d, *J* = 8.7 Hz), 127.6, 124.4 (d, *J* = 3.6 Hz), 124.3, 122.6, 121.9 (d, *J* = 7.9 Hz), 120.5 (d, *J* = 2.1 Hz), 119.8 (d, *J* = 3.0 Hz), 117.4, 116.3 (d, *J* = 21.0 Hz), 111.4, 49.2 (d, *J* = 6.5 Hz), 39.1;

^{19}F NMR (377 MHz, CDCl3) δ -121.8;

HRMS (ESI, m/z) calcd. for $C_{16}H_{14}FNONa^+$: 278.0952, found: 278.0947.

***N*-(benzofuran-3-ylmethyl)-2-chloro-N-methylaniline 7j:**

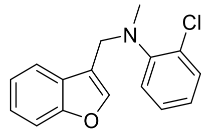

The reaction was performed with **1a** (25.81 mg, 0.10 mmol) and 2-chloro-*N*-methylaniline (14.16 mg, 0.10 mmol). The crude product was purified by flash chromatography on silica gel (eluted with PE / EA = 300:1) to give the product **7j** (12.81 mg, 47% yield) as a yellow oil;

^{1}H NMR (400 MHz, $CDCl_3$) δ 7.60 – 7.50 (m, 2H), 7.49 – 7.38 (m, 2H), 7.27 (ddd, *J* = 8.6, 7.4, 1.4 Hz, 1H), 7.21 – 7.12 (m, 2H), 7.06 – 6.95 (m, 2H), 4.35 (d, *J* = 1.0 Hz, 2H), 2.74 (s, 3H);

^{13}C NMR (101 MHz, $CDCl_3$) δ 155.5, 149.5, 143.3, 130.8, 129.2, 127.7, 127.4, 124.3, 123.9, 122.5, 121.8, 120.7, 117.2, 111.3, 49.6, 40.4;

HRMS (ESI, m/z) calcd. for $C_{16}H_{14}ClNONa^+$: 272.0837, found: 272.0830.

***N*-(benzofuran-3-ylmethyl)-2-bromo-*N*-methylaniline 7k:**

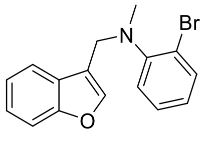

The reaction was performed with **1a** (25.81 mg, 0.10 mmol) and 2-bromo-*N*-methylaniline (18.61 mg, 0.10 mmol). The crude product was purified by flash chromatography on silica gel (eluted with PE / EA = 300:1) to give the product **7k** (12.90 mg, 41% yield) as a yellow oil;

^{1}H NMR (400 MHz, $CDCl_3$) δ 7.65 – 7.53 (m, 3H), 7.45 (dt, *J* = 8.2, 0.8 Hz, 1H), 7.30 – 7.15 (m, 3H), 7.06 (dd, *J* = 8.0, 1.6 Hz, 1H), 6.93 (ddd, *J* = 7.8, 7.2, 1.6 Hz, 1H), 4.33 (d, *J* = 1.0 Hz, 2H), 2.74 (s, 3H);

^{13}C NMR (101 MHz, $CDCl_3$) δ 155.5, 150.9, 143.4, 134.0, 128.1, 127.7, 124.6, 124.3, 122.5, 122.3, 120.7, 120.3, 117.1, 111.3, 50.0, 40.9;

HRMS (ESI, m/z) calcd. for $C_{16}H_{14}BrNOH^+$: 316.0332, found: 316.0323.

***N*-(benzofuran-3-ylmethyl)-*N*-methylnaphthalen-1-amine 7l:**

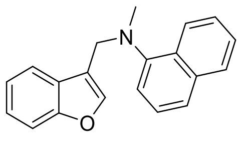

The reaction was performed with **1a** (25.81 mg, 0.10 mmol) and *N*-methylnaphthalen-1-amine (15.72 mg, 0.10 mmol). The crude product was purified by flash chromatography on silica gel (eluted with PE / EA = 300:1) to give the product **7l** (13.83 mg, 48% yield) as a yellow oil;

^{1}H NMR (400 MHz, $CDCl_3$) δ 8.36 (dddd, *J* = 8.0, 4.2, 3.0, 1.6 Hz, 1H), 7.90 – 7.77 (m, 1H), 7.61 – 7.53 (m, 2H), 7.52 – 7.44 (m, 4H), 7.38 (dd, *J* = 8.2, 7.4 Hz, 1H), 7.31 – 7.22 (m, 1H), 7.17 (td, *J* = 7.4, 1.0 Hz, 1H), 7.11 (dd, *J* = 7.4, 1.0 Hz, 1H), 4.41 (d, *J* = 1.0 Hz, 2H), 2.84 (s, 3H);

^{13}C NMR (101 MHz, $CDCl_3$) δ 155.6, 150.0, 143.1, 134.9, 129.4, 128.5, 127.8, 125.9, 125.8, 125.5, 124.3, 123.9, 123.5, 122.5, 120.4, 117.8, 115.8, 111.5, 51.1, 42.1;

HRMS (ESI, m/z) calcd. for $C_{20}H_{17}NOH^+$: 288.1383, found: 288.1384.

***N*-(benzofuran-3-ylmethyl)-*N*-phenylaniline 7m:**

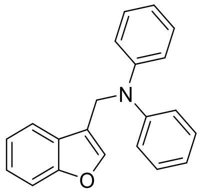

The reaction was performed with **1a** (25.81 mg, 0.10 mmol) and diphenylamine (16.92 mg, 0.10 mmol). The crude product was purified by flash chromatography on silica gel (eluted with PE / EA = 300:1) to give the product **7m** (6.04 mg, 20% yield) as a yellow oil;

^{1}H NMR (400 MHz, $CDCl_3$) δ 7.53 – 7.41 (m, 3H), 7.31 – 7.19 (m, 6H), 7.14 – 7.05 (m, 4H), 6.95 (tt, *J* = 7.2, 1.2 Hz, 2H), 5.05 (d, *J* = 1.4 Hz, 2H);

^{13}C NMR (101 MHz, $CDCl_3$) δ 155.5, 147.8, 142.8, 129.3, 124.4, 122.5, 121.7, 120.9, 119.6, 118.0, 117.8, 111.6, 47.4;

HRMS (ESI, m/z) calcd. for $C_{21}H_{17}NONa^+$: 322.1202, found: 322.1191.

***N,N*-bis(benzofuran-3-ylmethyl)aniline 7a':**

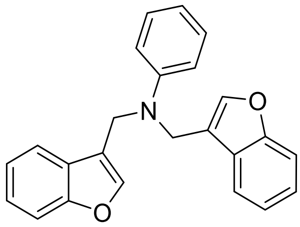

The reaction was performed with **1a** (25.81 mg, 0.10 mmol) and aniline **6a** (9.31 mg, 0.10 mmol). The crude product was purified by flash chromatography on silica gel (eluted with PE) to give the product **7a'** (2.11 mg, 6% yield) as a yellow oil;

The reaction was performed with **1a** (25.81 mg, 0.10 mmol) and **7a** (22.33 mg, 0.10 mmol). The crude product was purified by flash chromatography on silica gel (eluted with PE) to give the product **7a'** (7.07 mg, 20% yield) as a yellow oil;

^{1}H NMR (400 MHz, $CDCl_3$) δ 7.45 (ddt, *J* = 16.8, 8.4, 1.0 Hz, 6H), 7.32 – 7.17 (m, 6H), 7.02 – 6.91 (m, 2H), 6.81 (tt, *J* = 7.2, 1.0 Hz, 1H), 4.65 (d, *J* = 1.2 Hz, 4H);

^{13}C NMR (101 MHz, $CDCl_3$) δ 155.6, 149.0, 142.7, 129.4, 127.1, 124.6, 122.6, 119.9, 118.1, 117.4, 114.4, 111.6, 45.0;

HRMS (ESI, m/z) calcd. for $C_{24}H_{19}NO_2H^+$: 354.1489, found: 354.1474.

(benzofuran-3-ylmethyl)diphenylphosphane-borane complex 8:

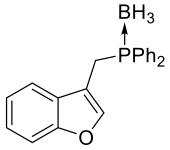

The reaction was performed with **1a** (1.0 g, 3.88 mmol) and $HPPh_2$ (1.44 g, 7.75 mmol). The crude product was purified by flash chromatography on silica gel (eluted with PE / EA = 100:1) to give the product **8** (1.04 g, 81% yield, m.p. 84 – 86 ^oC) as a white solid;

^{1}H NMR (400 MHz, $CDCl_3$) δ 7.74 – 7.60 (m, 4H), 7.52 – 7.45 (m, 2H), 7.44 – 7.35 (m, 5H), 7.27 – 7.18 (m, 4H), 7.10 (td, *J* = 7.2, 0.8 Hz, 1H), 3.64 (d, *J* = 11.2 Hz, 2H), 1.27 – 0.52 (m, 3H);

^{13}C NMR (101 MHz, $CDCl_3$) δ 154.7, 143.6 (d, *J* = 7.0 Hz), 132.6 (d, *J* = 8.9 Hz), 131.5 (d, *J* = 2.5 Hz), 128.9 (d, *J* = 9.9 Hz), 128.7 (d, *J* = 54.0 Hz), 127.9 (d, *J* = 3.6 Hz), 124.3, 122.5, 119.7, 111.4, 111.2 (d, *J* = 4.4 Hz), 22.3 (d, *J* = 35.7 Hz);

^{31}P NMR (162 MHz, $CDCl_3$) δ 17.4;

HRMS (ESI, m/z) calcd. for $C_{21}H_{20}BOPNa^+$: 353.1237, found: 353.1234.

3-((phenylthio)methyl)benzofuran S12:

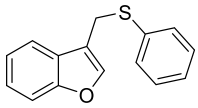

The reaction was performed with **1a** (77.42 mg, 0.3 mmol) and 1,2-diphenyldisulfane (21.83 mg, 0.1 mmol). The crude product was purified by flash chromatography on silica gel (eluted with PE / EA = 300:1) to give the product **S12** (*t*-BuOK: 2.6 mg, 11% yield. LiHMDS: 22.61 mg, 94% yield) as a yellow oil;

^{1}H NMR (400 MHz, $CDCl_3$) δ 7.69 – 7.60 (m, 1H), 7.48 – 7.40 (m, 2H), 7.35 – 7.23 (m, 6H), 7.22 – 7.17 (m, 1H), 4.18 (d, *J* = 1.0 Hz, 2H);

^{13}C NMR (101 MHz, $CDCl_3$) δ 155.5, 142.8, 135.9, 130.8, 130.4, 129.0, 126.8, 124.6, 122.7, 120.0, 116.7, 111.6, 28.3;

HRMS (APCI, m/z) calcd. for $C_{15}H_{12}OSH^+$: 241.0682, found: 241.0696.

(benzofuran-3-ylmethyl)diphenylphosphine sulfide 13:

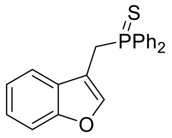

The reaction was performed with **3a** (33.23 mg, 0.1 mmol) and Lawesson's reagent (28.31 mg, 0.07 mmol). The crude product was purified by flash chromatography on silica gel (eluted with PE / EA = 100:1) to give the product **13** (34.53 mg, 99% yield, m.p. 93 – 95 °C) as a white solid;

^{1}H NMR (400 MHz, $CDCl_3$) δ 7.91 – 7.76 (m, 4H), 7.51 – 7.45 (m, 3H), 7.44 – 7.34 (m, 5H), 7.20 (td, *J* = 8.4, 1.6 Hz, 2H), 7.10 – 7.03 (m, 1H), 3.91 (dd, *J* = 13.2, 1.0 Hz, 2H);

^{13}C NMR (101 MHz, $CDCl_3$) δ 154.7, 143.9 (d, *J* = 8.0 Hz), 132.3 (d, *J* = 80.2 Hz), 131.8 (d, *J* = 2.9 Hz), 131.5 (d, *J* = 9.7 Hz), 128.7 (d, *J* = 11.8 Hz), 128.0 (d, *J* = 4.4 Hz), 124.3, 122.4, 119.5, 111.4, 110.2 (d, *J* = 7.4 Hz), 30.0 (d, *J* = 54.7 Hz);

^{31}P NMR (162 MHz, $CDCl_3$) δ 41.0;

HRMS (ESI, m/z) calcd. for $C_{21}H_{17}OPSNa^+$: 371.0630, found: 371.0627.

3-((diphenylphosphoryl)methyl)benzofuran-2-carbaldehyde 14:

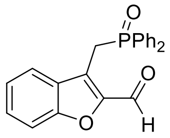

The reaction was performed with **3a** (500 mg, 1.50 mmol) and 1,1-dichlorodimethyl ether (605.31 mg, 5.27 mmol). The crude product was purified by flash chromatography on silica gel (eluted with PE / EA = 8:1) to give the product **14** (325.28 mg, 60% yield, m.p. 210 – 212 °C) as a white solid;

^{1}H NMR (400 MHz, $CDCl_3$) δ 9.75 (s, 1H), 7.94 (d, *J* = 8.0 Hz, 1H), 7.81 – 7.74 (m, 4H), 7.54 – 7.46 (m, 4H), 7.46 – 7.40 (m, 4H), 7.33 – 7.28 (m, 1H), 4.24 (d, *J* = 14.2 Hz, 2H);

^{13}C NMR (101 MHz, $CDCl_3$) δ 181.5, 155.3, 148.5 (d, *J* = 7.4 Hz), 132.2 (d, *J* = 2.7 Hz), 132.1, 131.1, 131.1 (d, *J* = 9.4 Hz), 129.3, 128.6 (d, *J* = 11.7 Hz), 128.1 (d, *J* = 2.4 Hz), 124.0 (d, *J* = 6.9 Hz), 121.6 (d, *J* = 9.1 Hz), 112.1, 27.8 (d, *J* = 65.4 Hz);

^{31}P NMR (162 MHz, $CDCl_3$) δ 29.1;

HRMS (ESI, m/z) calcd. for $C_{22}H_{17}O_3PNa^+$: 383.0808, found: 383.0808.

((2-(perfluorobutyl)benzofuran-3-yl)methyl)diphenylphosphine oxide 15:

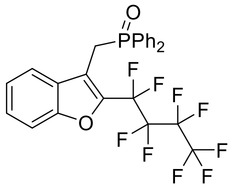

The reaction was performed with **3a** (33.23 mg, 0.1 mmol) and Perfluorobutyl iodide (69.19 mg, 0.2 mmol). The crude product was purified by flash chromatography on silica gel (eluted with PE / EA = 10:1) to give the product **15** (40.71 mg, 74% yield, m.p. 165 – 166 °C) as a white solid;

^{1}H NMR (400 MHz, $CDCl_3$) δ 7.93 (d, *J* = 7.8 Hz, 1H), 7.74 – 7.66 (m, 4H), 7.57 – 7.51 (m, 2H), 7.49 – 7.40 (m, 6H), 7.32 – 7.27 (m, 1H), 3.90 (d, *J* = 13.8 Hz, 2H).;

^{13}C NMR (101 MHz, $CDCl_3$) δ 154.8, 138.6 – 137.5 (m), 132.2 (d, *J* = 2.9 Hz), 132.1, 131.1 (d, *J* = 9.3 Hz), 128.6 (d, *J* = 11.7 Hz), 127.5, 127.4, 123.8, 123.4, 117.0 (d, *J* = 9.0 Hz), 111.5, 27.9 (d, *J* = 66.7 Hz);

^{19}F NMR (377 MHz, CDCl3) δ -80.9 (t, *J* = 9.7 Hz), -110.5 – -110.5 (m), -123.1 (q, *J* = 9.5 Hz), -126.2 (td, *J* = 12.3, 5.1 Hz);

^{31}P NMR (162 MHz, $CDCl_3$) δ 28.1;

HRMS (ESI, m/z) calcd. for $C_{25}H_{16}F_9O_2PH^+$: 551.0817, found: 551.0809.

((2-bromobenzofuran-3-yl)methyl)diphenylphosphine oxide 16:

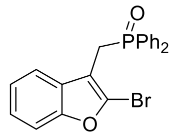

The reaction was performed with **3a** (1.0 g, 3.01 mmol) and NBS (0.49 mg, 2.74 mmol). The crude product was purified by flash chromatography on silica gel (eluted with PE / EA = 1:3) to give the product **16** (1.11 g, 95% yield, m.p. 146 – 148 °C) as a colorless solid;

^{1}H NMR (400 MHz, $CDCl_3$) δ 7.81 – 7.70 (m, 4H), 7.68 – 7.60 (m, 1H), 7.57 – 7.49 (m, 2H), 7.48 – 7.41 (m, 4H), 7.38 – 7.31 (m, 1H), 7.25 – 7.14 (m, 2H), 3.67 (d, *J* = 12.8 Hz, 2H);

^{13}C NMR (101 MHz, $CDCl_3$) δ 155.2, 132.1 (d, *J* = 2.5 Hz), 132.0 (d, *J* = 99.5 Hz), 131.1 (d, *J* = 9.4 Hz), 128.7 (d, *J* = 11.7 Hz), 128.5, 128.25 (d, *J* = 1.9 Hz), 123.4, 120.9, 111.2, 111.1, 110.6, 28.5 (d, *J* = 69.6 Hz);

^{31}P NMR (162 MHz, $CDCl_3$) δ 28.6;

HRMS (ESI, m/z) calcd. for $C_{21}H_{16}BrO_2PH^+$: 411.0144, found: 411.0135.

diphenyl((2-(4,4,5,5-tetramethyl-1,3,2-dioxaborolan-2-yl)benzofuran-3-yl)methyl)phosphine oxide 17:

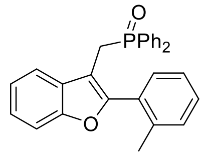

The reaction was performed with **16** (41.12 mg, 0.1 mmol) and *o*-tolylboronic acid (27.19 mg, 0.2 mmol). The crude product was purified by flash chromatography on silica gel (eluted with PE / EA = 1:3) to give the product **17** (26.22 mg, 62% yield, m.p. 150 – 151 °C) as a white solid;

^{1}H NMR (400 MHz, $CDCl_3$) δ 7.70 (dd, *J* = 7.6, 1.2 Hz, 1H), 7.58 – 7.50 (m, 4H), 7.49 – 7.42 (m, 2H), 7.39 (d, *J* = 8.0 Hz, 1H), 7.37 – 7.27 (m, 5H), 7.26 – 7.10 (m, 5H), 3.77 (dd, *J* = 13.2, 1.2 Hz, 2H), 1.96 (s, 3H);

^{13}C NMR (101 MHz, $CDCl_3$) δ 154.2, 154.1 (d, *J* = 9.0 Hz), 138.9, 132.7, 131.8 (d, *J* = 3.0 Hz), 131.0 (d, *J* = 9.4 Hz), 130.1 (d, *J* = 133.0 Hz), 130.4, 129.5 (d, *J* = 2.2 Hz), 129.1, 128.6 (d, *J* = 11.6 Hz), 125.7, 124.4, 122.7, 121.6, 110.8, 107.6 (d, *J* = 8.7 Hz), 27.4 (d, *J* = 70.3 Hz), 20.0;

^{31}P NMR (162 MHz, $CDCl_3$) δ 30.1;

HRMS (ESI, m/z) calcd. for $C_{28}H_{22}O_2PH^+$: 423.1508, found: 423.1504.

methyl (*E*)-3-(3-((diphenylphosphoryl)methyl)benzofuran-2-yl)acrylate 18:

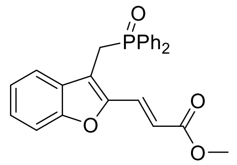

The reaction was performed with **16** (41.12 mg, 0.1 mmol) and methyl acrylate (17.22 mg, 18 μL, 0.2 mmol). The crude product was purified by flash chromatography on silica gel (eluted with PE / EA = 1:3) to give the product **18** (26.23 mg, 63% yield, m.p. 208 – 210 °C) as a white solid;

^{1}H NMR (400 MHz, $CDCl_3$) δ 7.79 – 7.68 (m, 4H), 7.57 – 7.48 (m, 3H), 7.47 – 7.40 (m, 4H), 7.38 (d, *J* = 8.2 Hz, 1H), 7.32 (dd, *J* = 7.2, 1.2 Hz, 1H), 7.22 – 7.12 (m, 2H), 6.39 (dd, *J* = 15.4, 1.2 Hz, 1H), 3.80 (d, *J* = 14.8 Hz, 2H), 3.80 (s, 3H);

^{13}C NMR (101 MHz, $CDCl_3$) δ 167.0, 154.7, 149.6 (d, *J* = 8.0 Hz), 132.2 (d, *J* = 2.0 Hz), 131.1 (d, *J* = 9.1 Hz), 129.0 (dd, *J* = 140.0, 1.7 Hz), 128.7 (d, *J* = 11.7 Hz), 128.6, 128.6, 126.8, 123.2, 121.5, 117.8 (d, *J* = 1.8 Hz), 114.9 (d, *J* = 9.5 Hz), 111.1, 51.7, 27.9 (d, *J* = 67.9 Hz);

^{31}P NMR (162 MHz, $CDCl_3$) δ 27.9;

HRMS (ESI, m/z) calcd. for $C_{25}H_{20}O_4PNa^+$: 439.1070, found: 439.1071.

Ⅲ. Supplementary Figures

Stereochemistry Determination by *X*-ray Crystallographic Analysis

A colorless brock crystal of **3c** was obtained by vaporization of its DCM / PE solution. A colorless brock crystal of **14** was obtained by vaporization of its MeOH / *n*-hexane solution.

CCDC **2209970** and **2209975** contain the supplementary *X*-ray crystallographic data of **3c** and **14**, respectively. These data can be obtained free of charge from The Cambridge Crystallographic Data Centre via www.ccdc.cam.ac.uk / data request / cif.

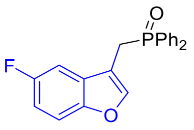<br>**3c**	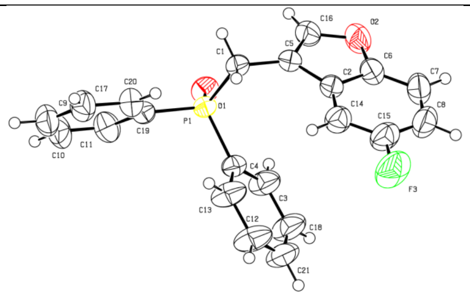<br>**CCDC: 2209970**
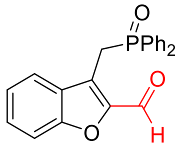<br>**14**	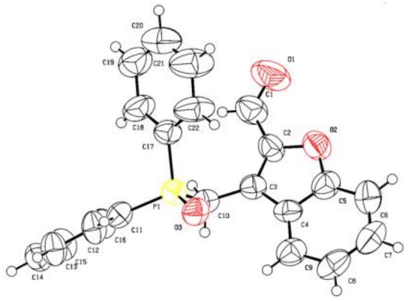<br>**CCDC: 2209975**

^{1}H NMR, ^{13}C NMR, ^{19}F NMR and ^{31}P NMR Spectra

Supplementary Figure 22. ^{1}H and ^{13}C NMR spectra of **1a**.

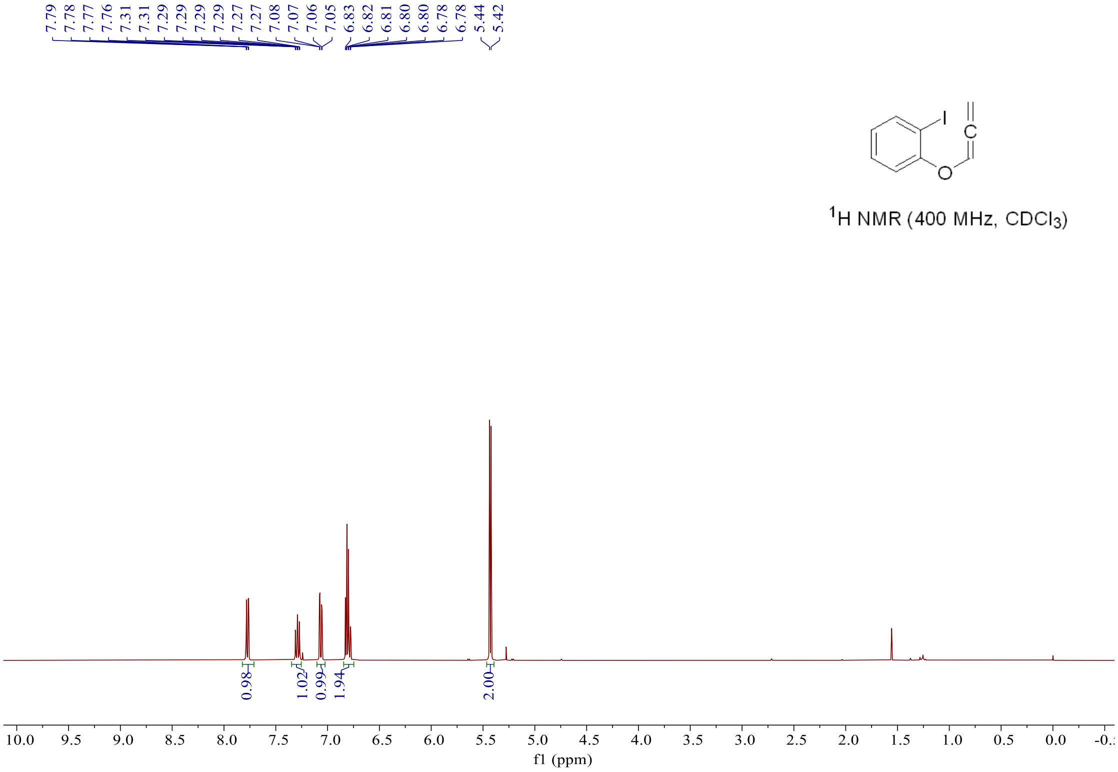

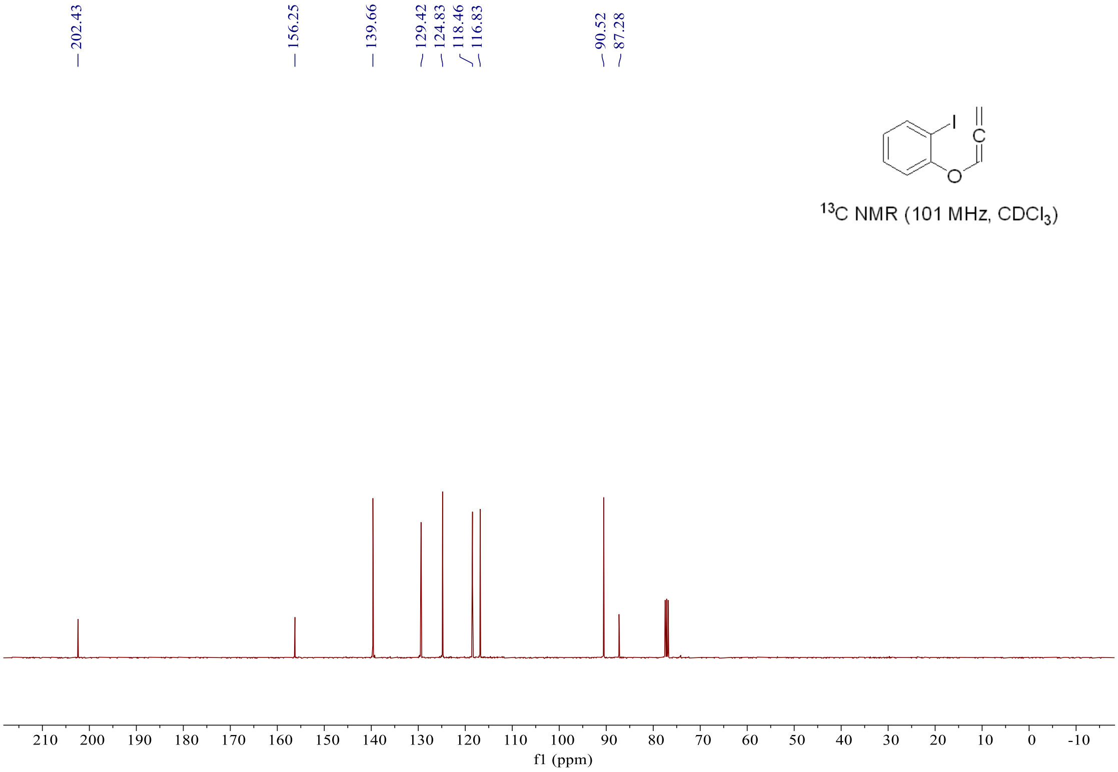

Supplementary Figure 23. ^1H and ^{13}C NMR spectra of **1b**.

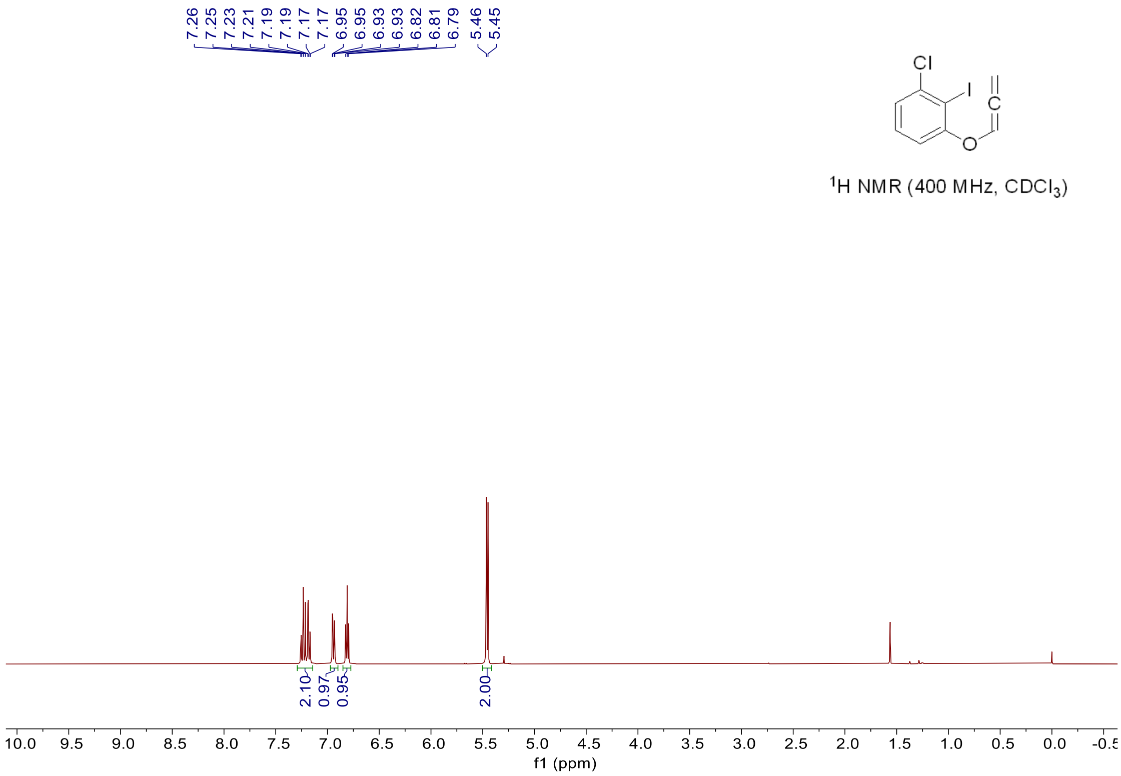

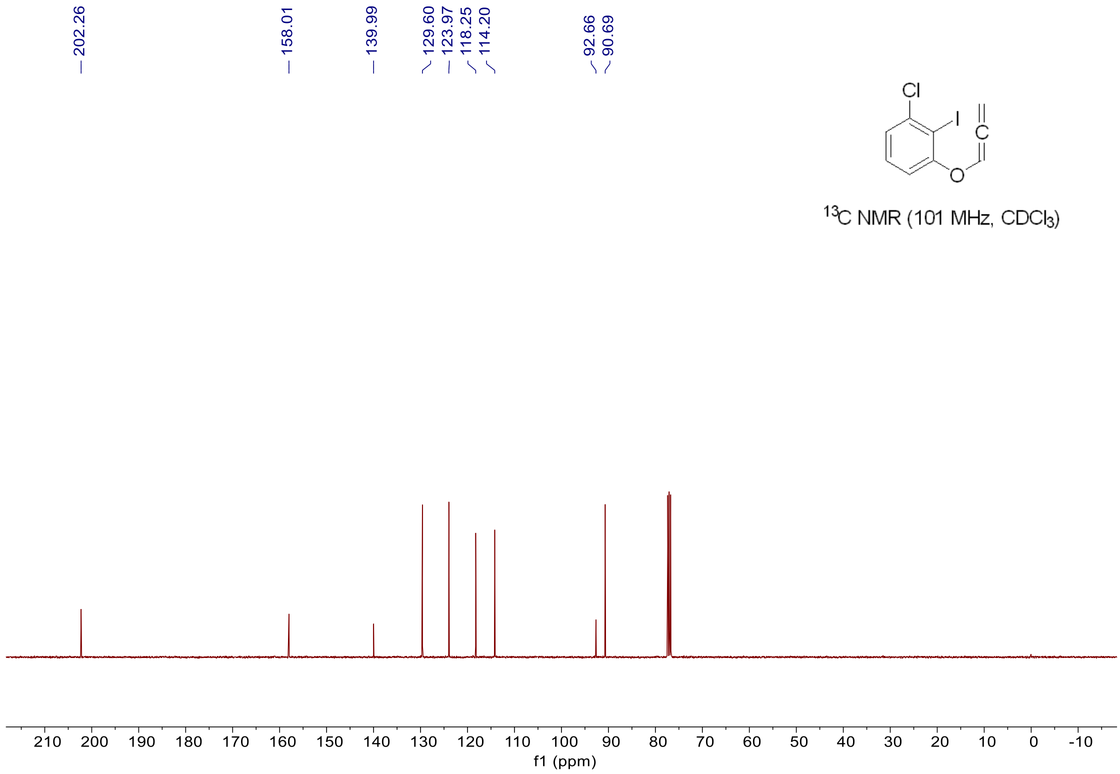

Supplementary Figure 24. ^{1}H, ^{13}C and ^{19}F NMR spectra of **1c**.

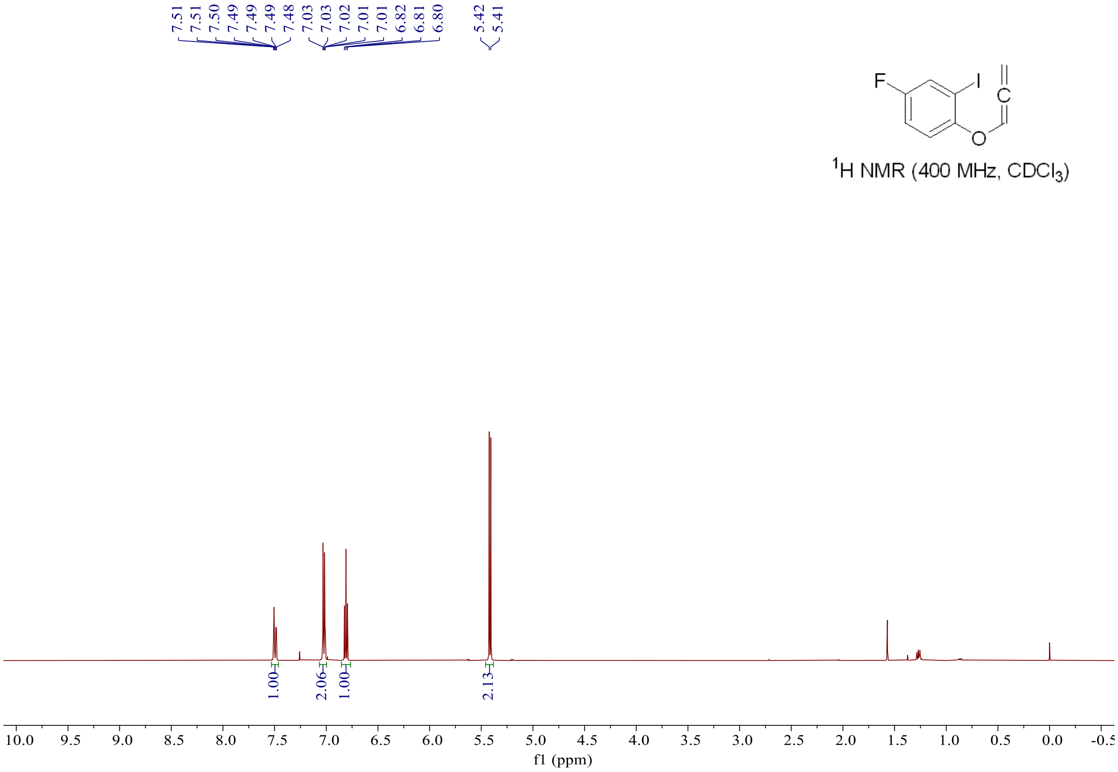

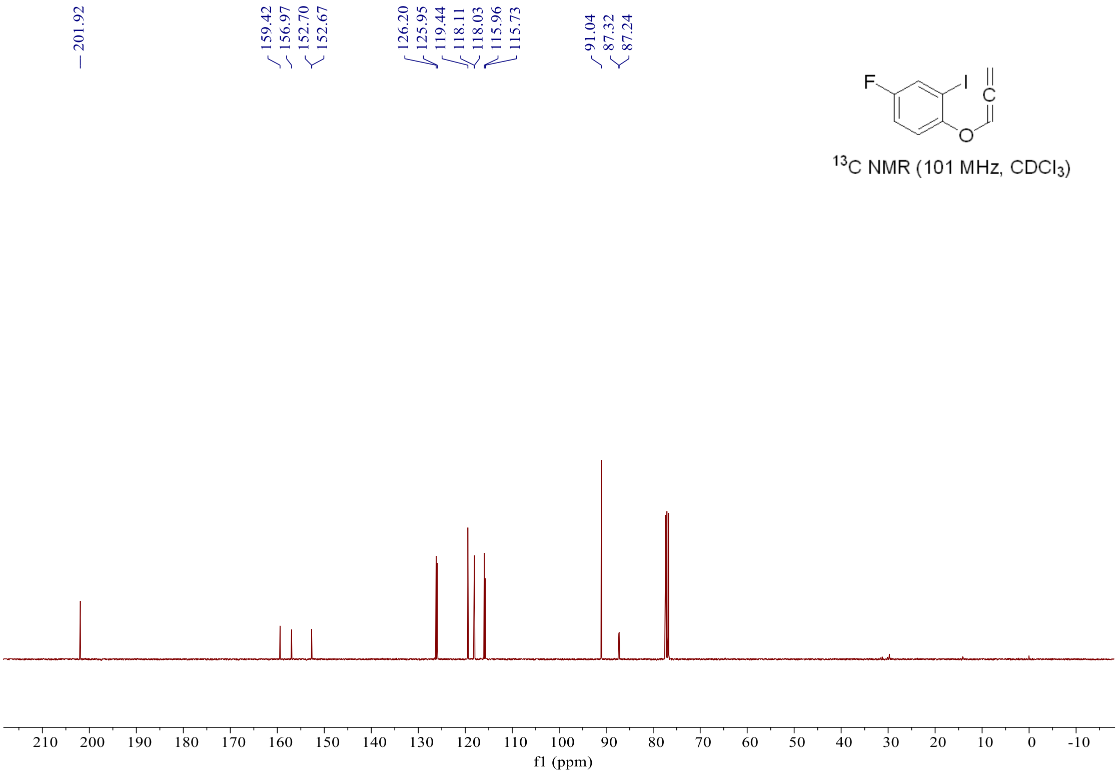

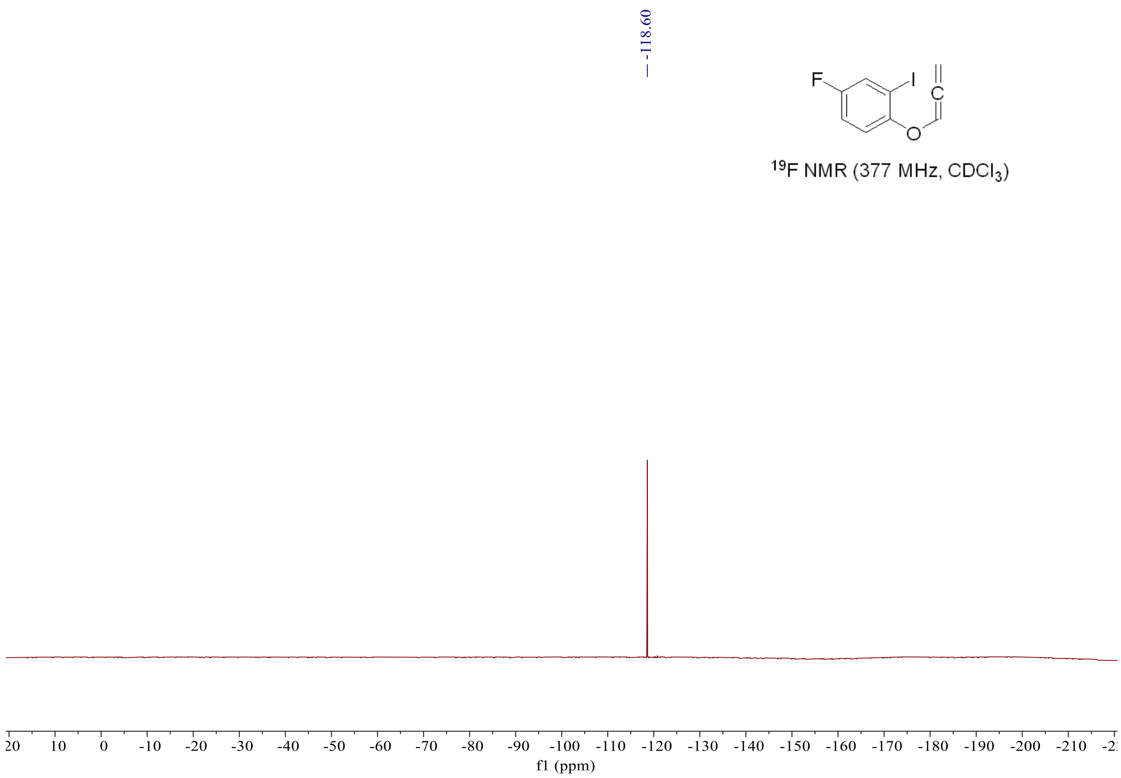
F
I
C
O
19F NMR (377 MHz, CDCl3)
-118.60
f1 (ppm)

Supplementary Figure 25. ^{1}H and ^{13}C NMR spectra of **1d**.

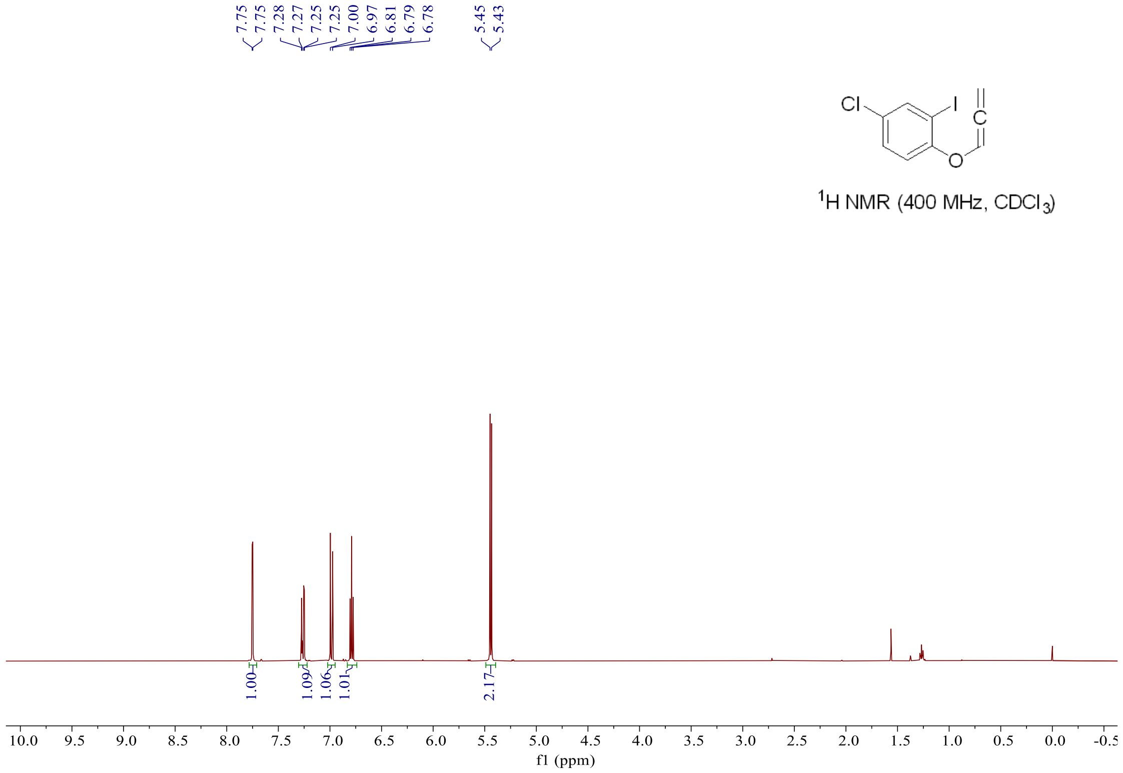

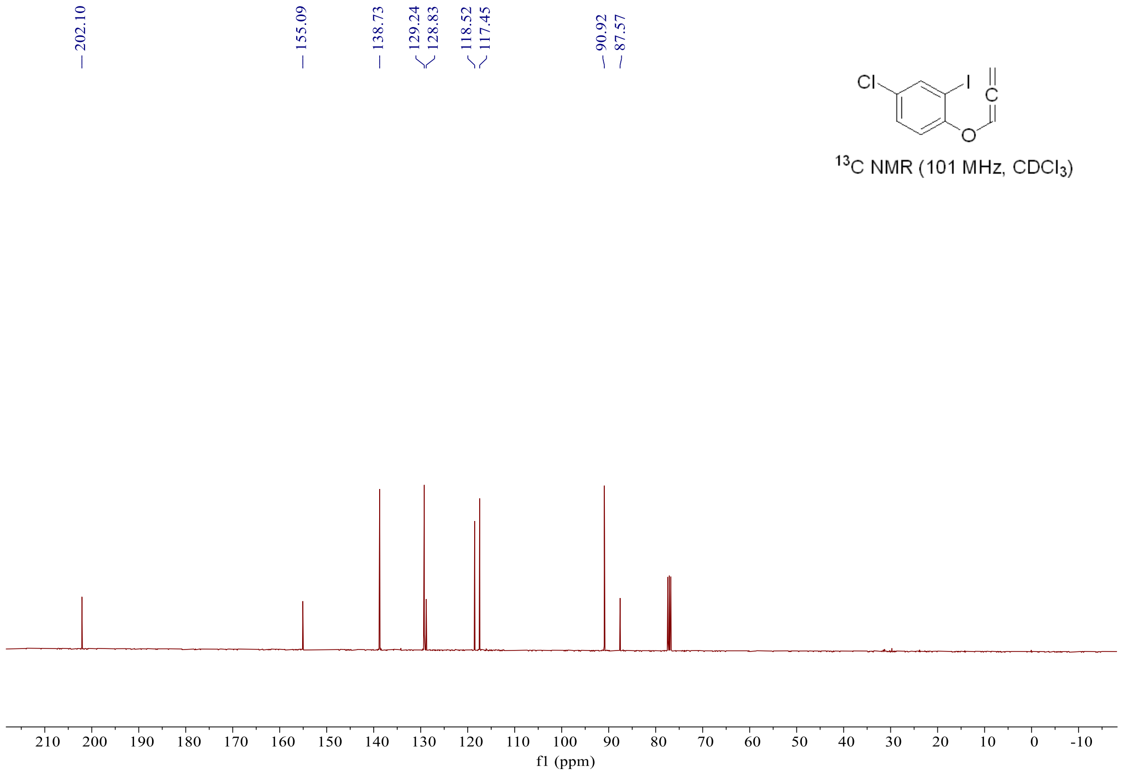

Supplementary Figure 26. ^{1}H, ^{13}C and ^{19}F NMR spectra of **1e.**

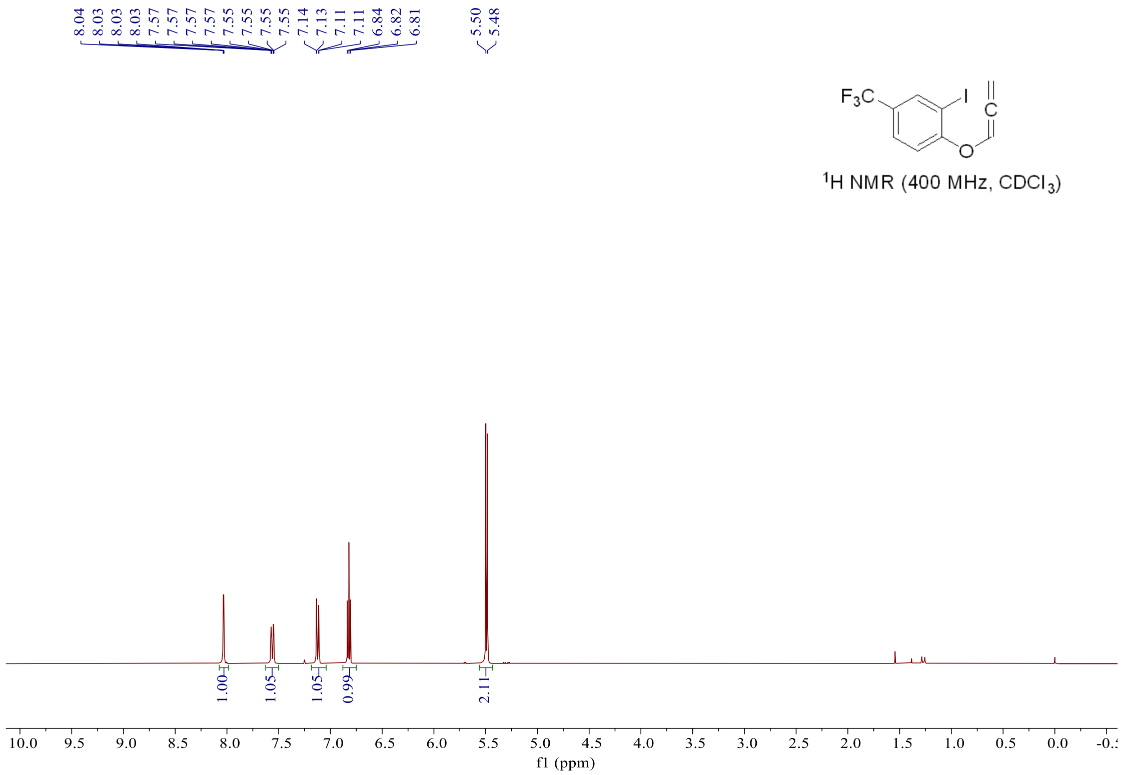

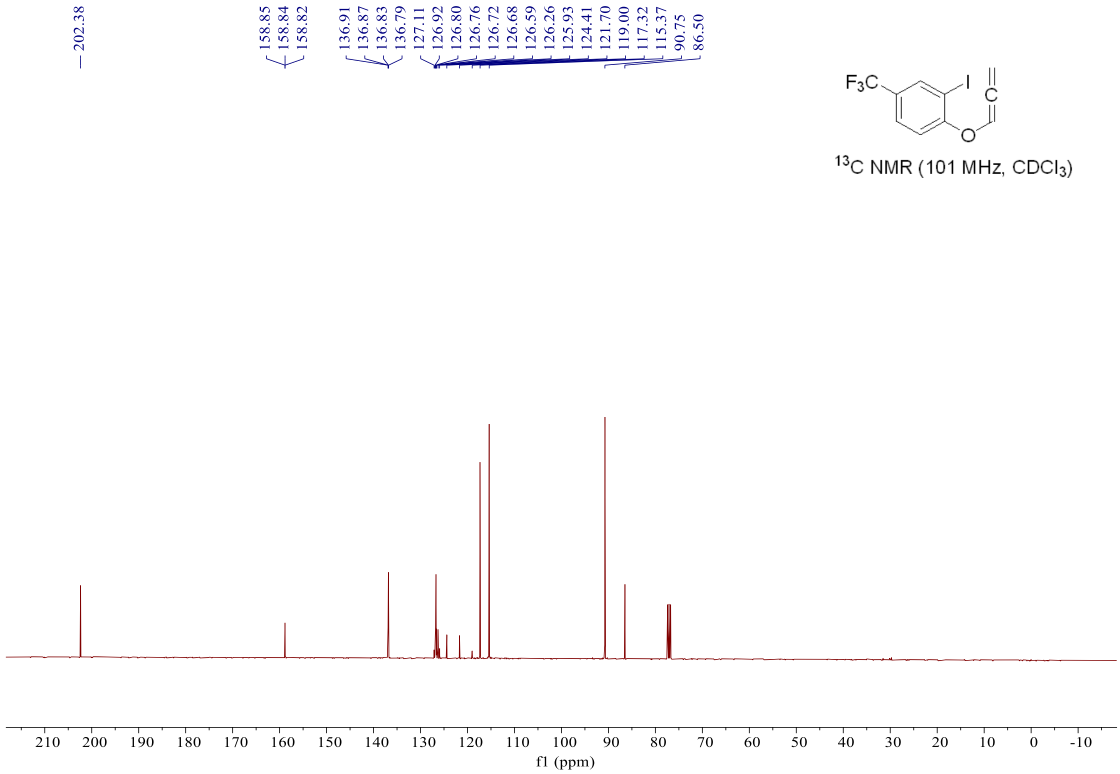

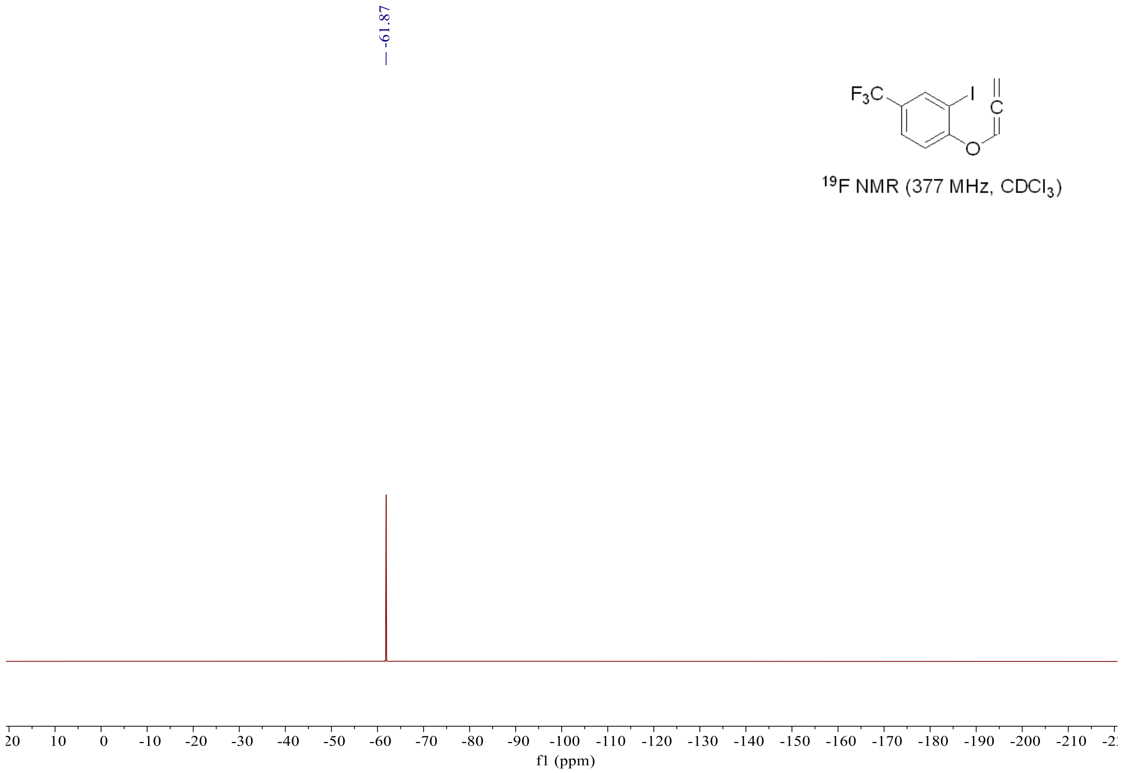

^{19}F NMR (377 MHz, $CDCl_3$)

Supplementary Figure 27. ^{1}H and ^{13}C NMR spectra of **1f.**

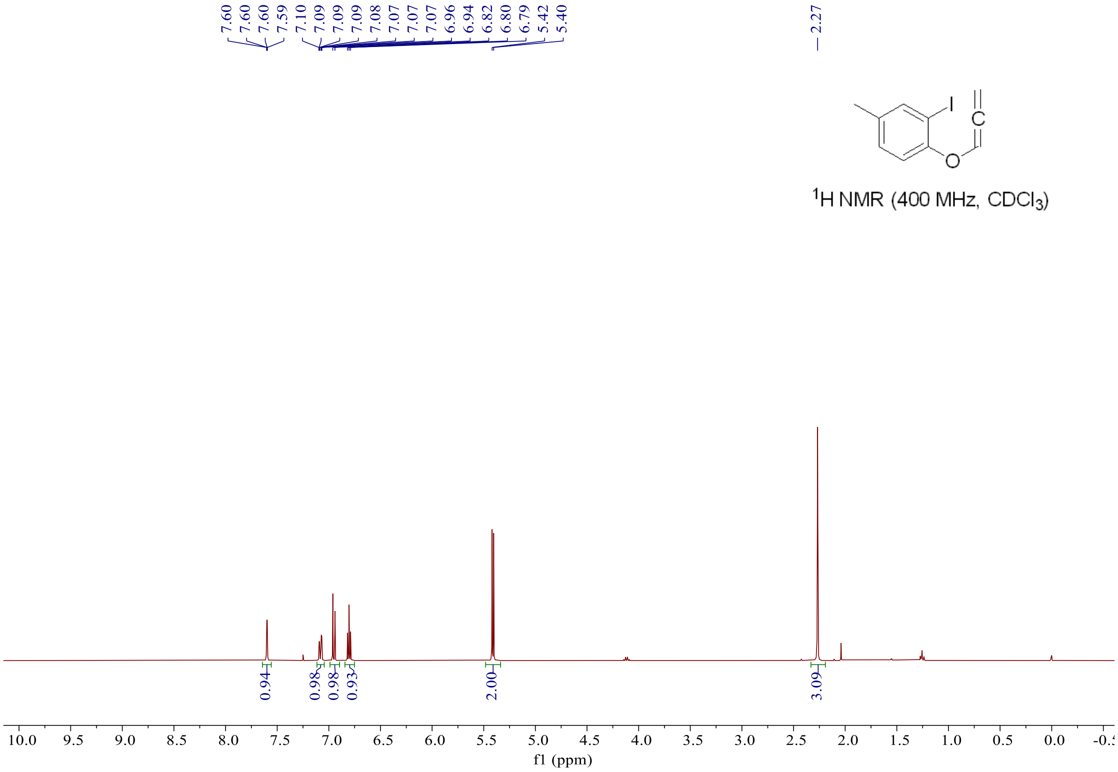

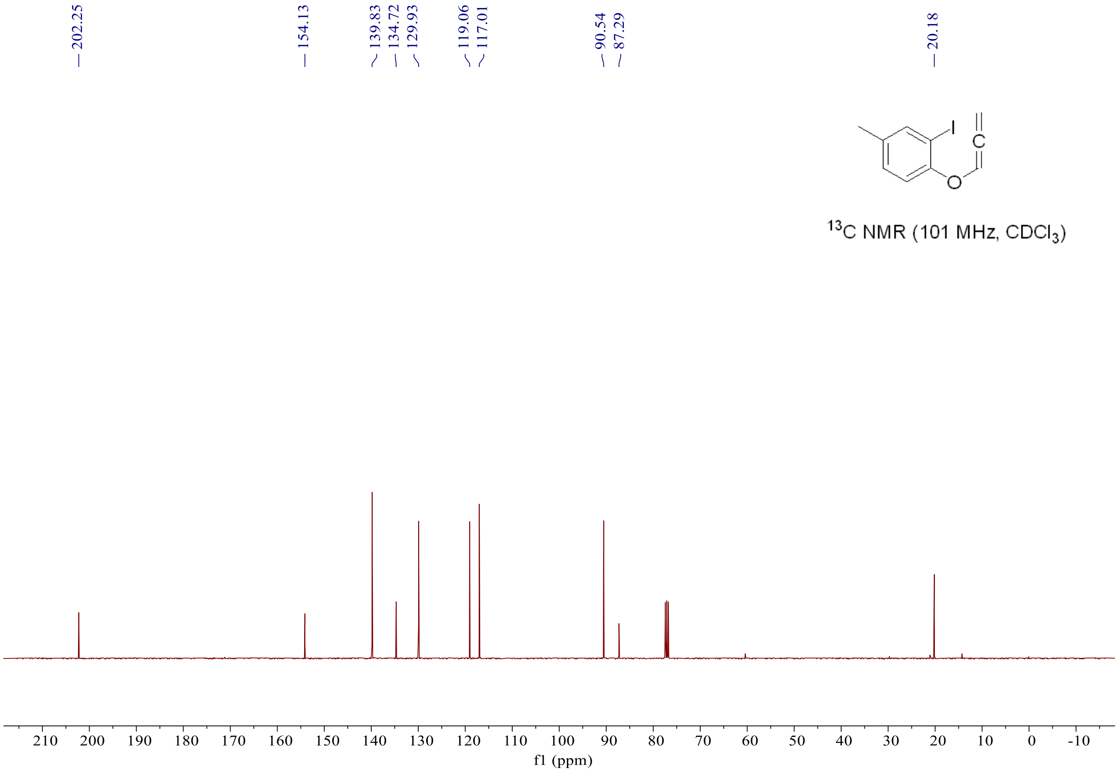

Supplementary Figure 28. ^{1}H and ^{13}C NMR spectra of **1g**.

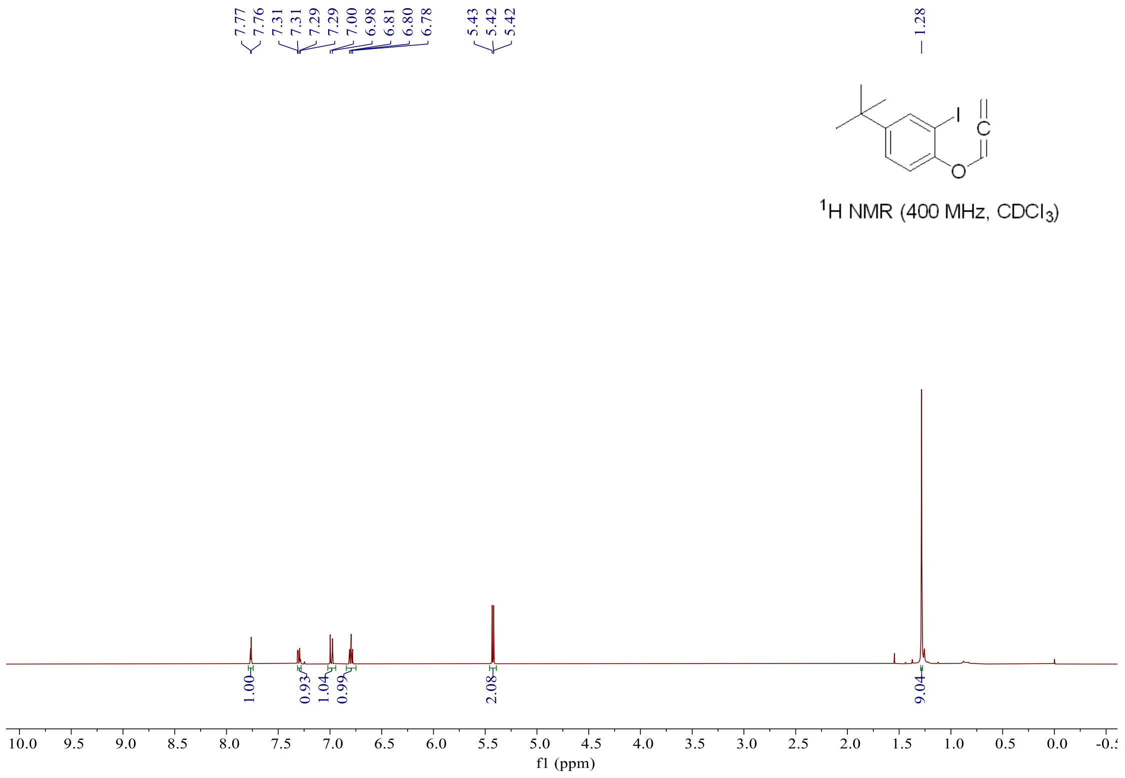

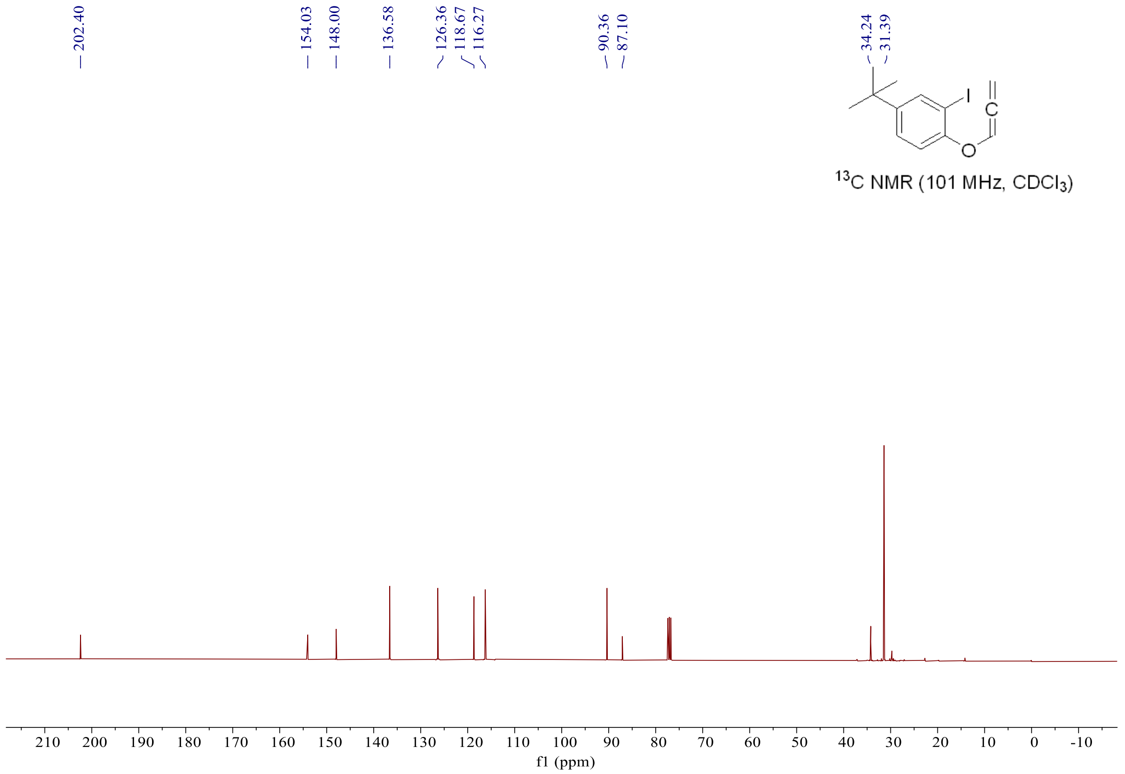

Supplementary Figure 29. ^{1}H and ^{13}C NMR spectra of **1h**.

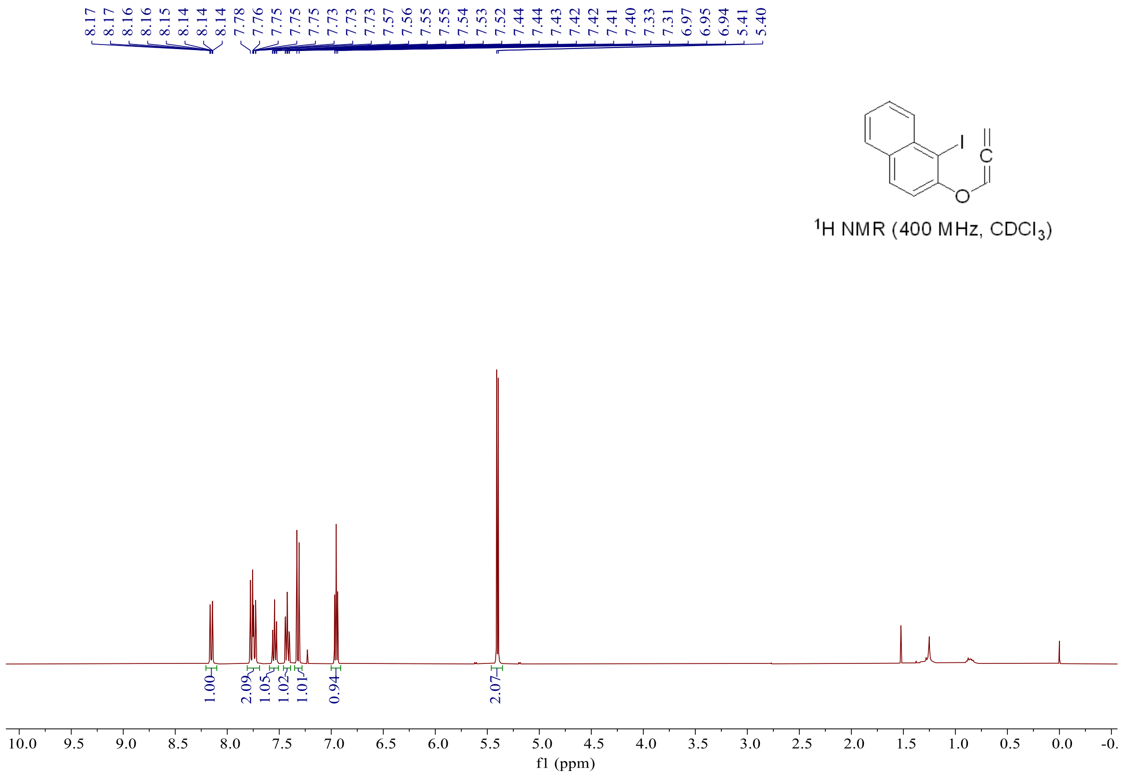

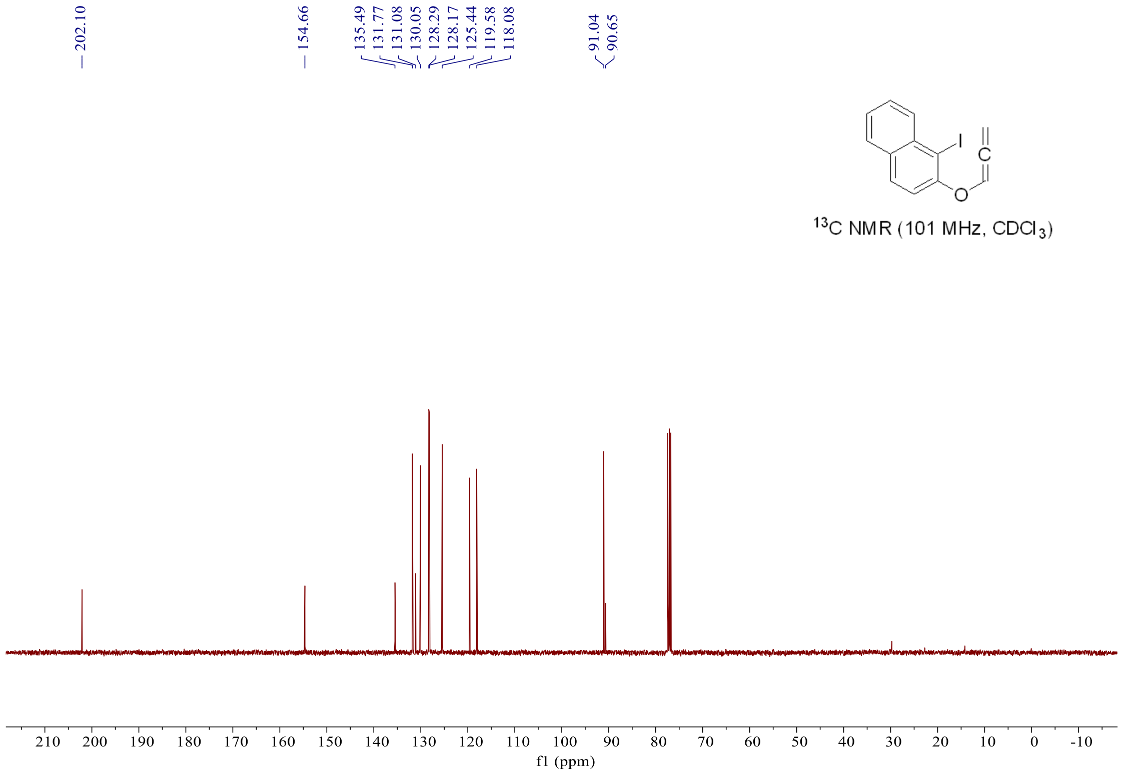

Supplementary Figure 30. ^{1}H and ^{13}C NMR spectra of **1i.**

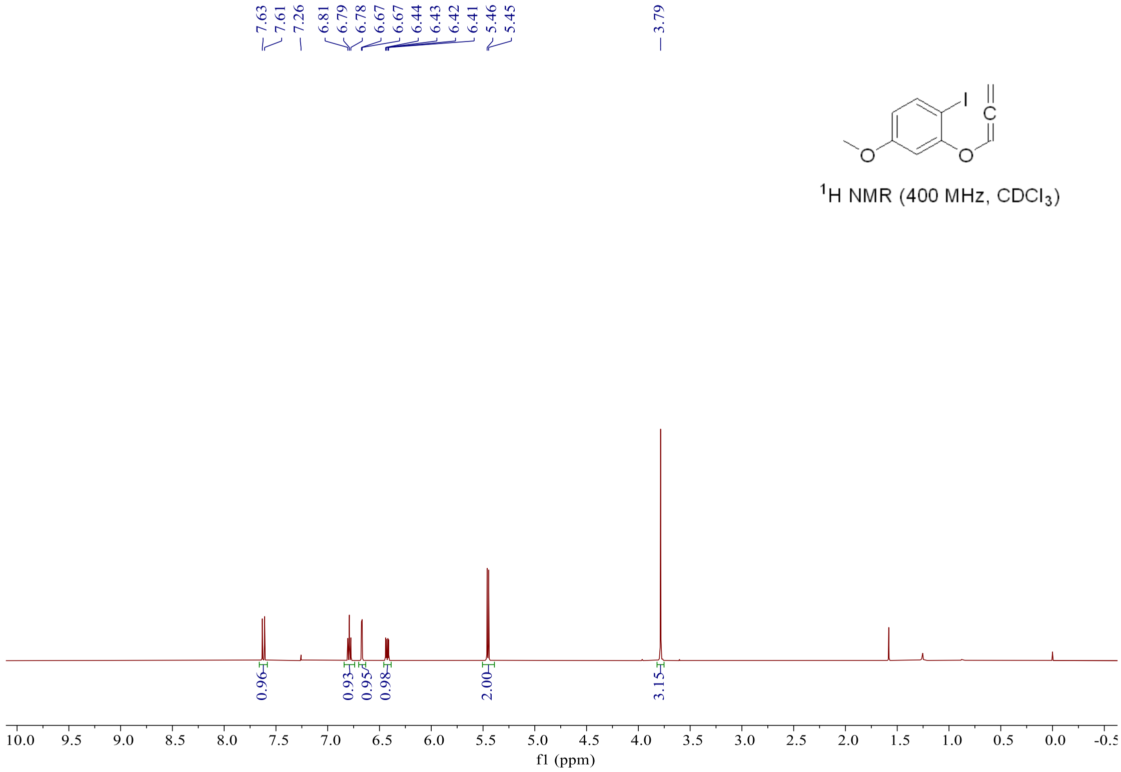

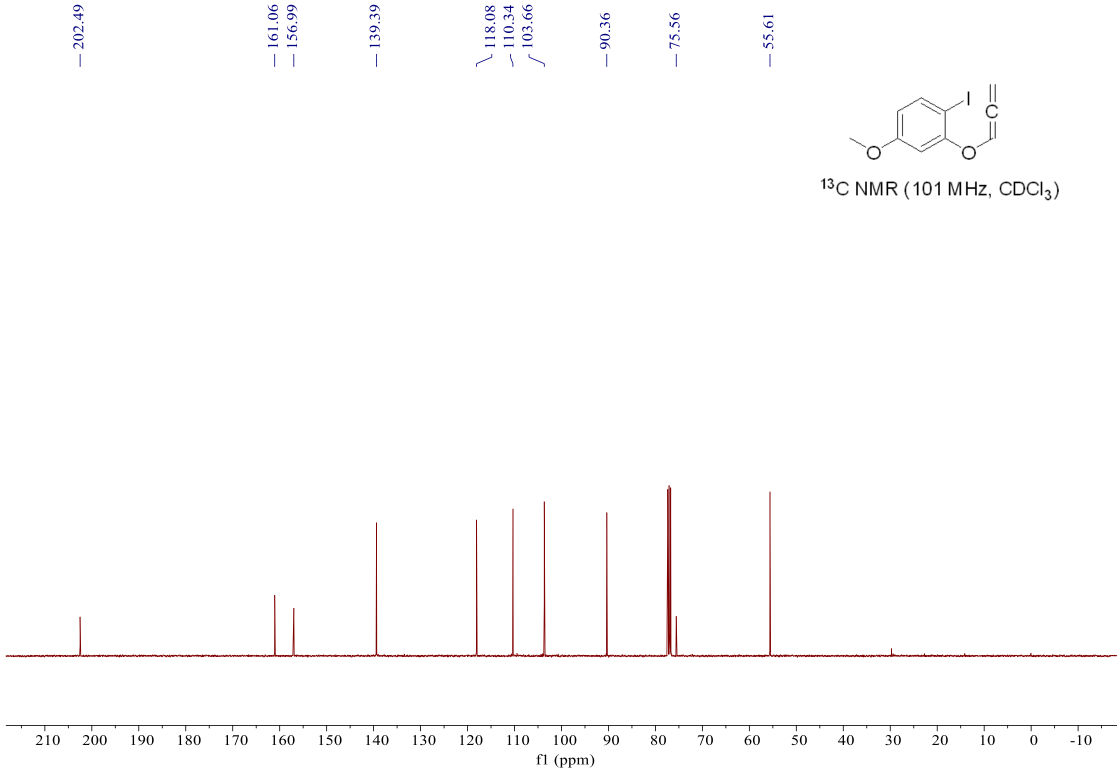

Supplementary Figure 31. ^{1}H and ^{13}C NMR spectra of **1j**.

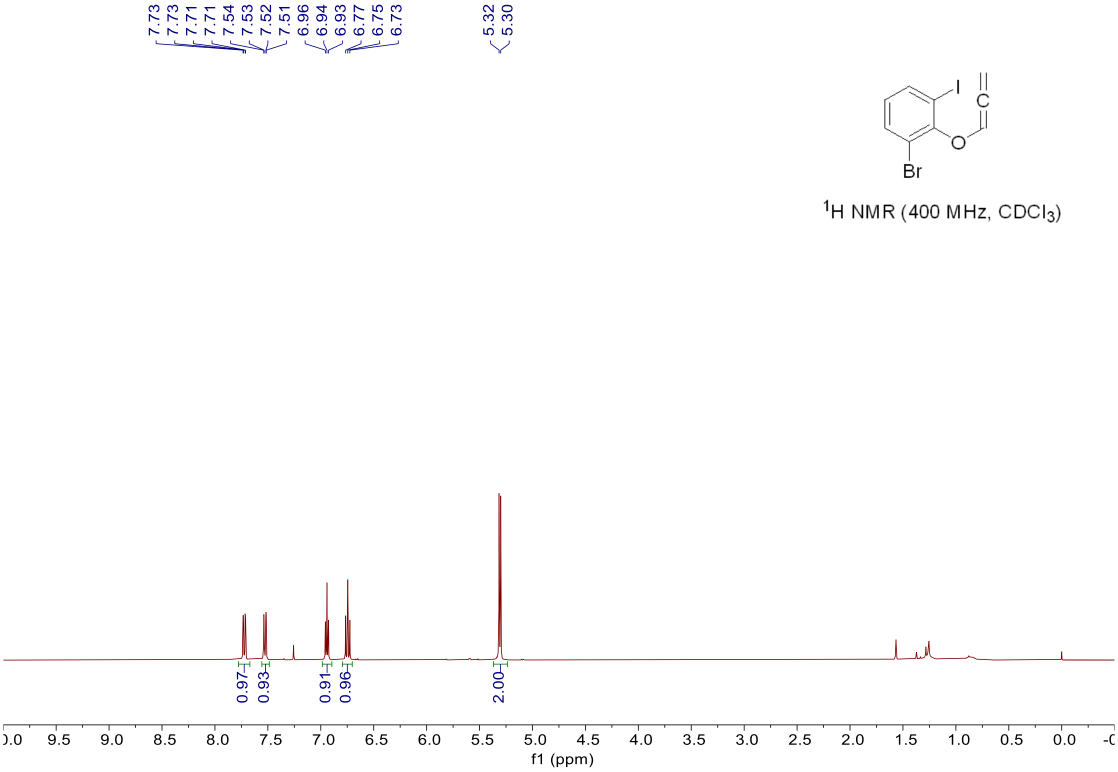

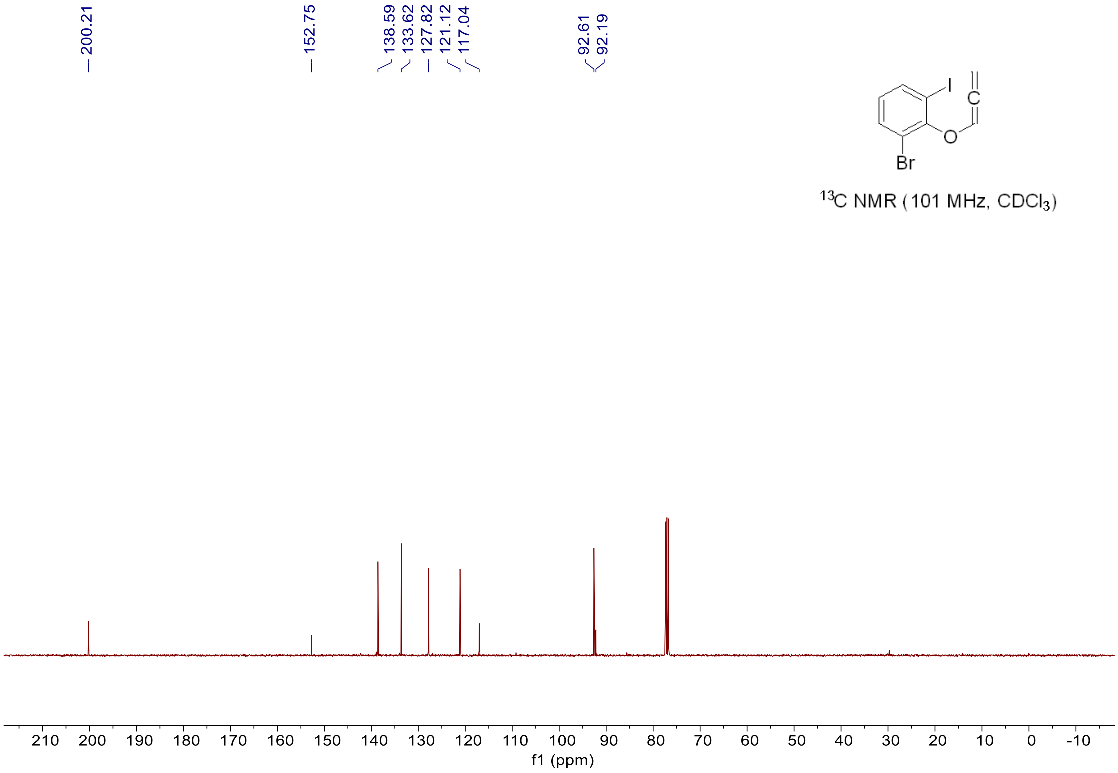

Supplementary Figure 32. ^{1}H and ^{13}C NMR spectra of **1k**.

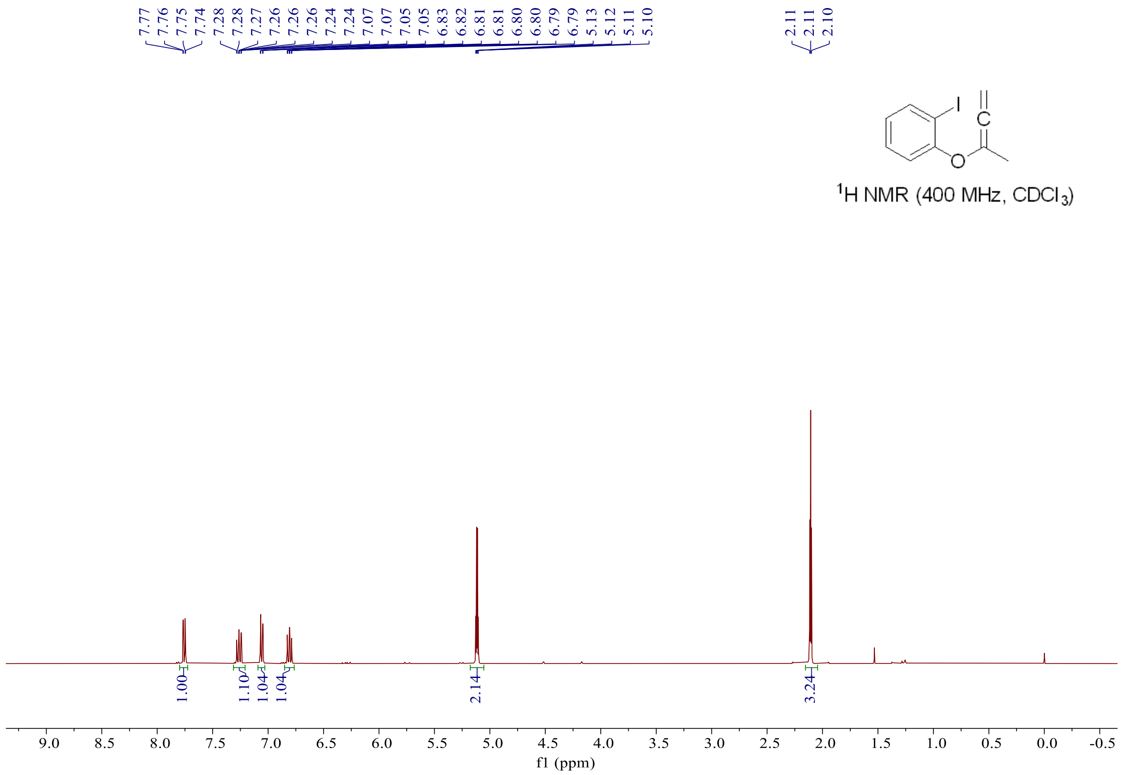

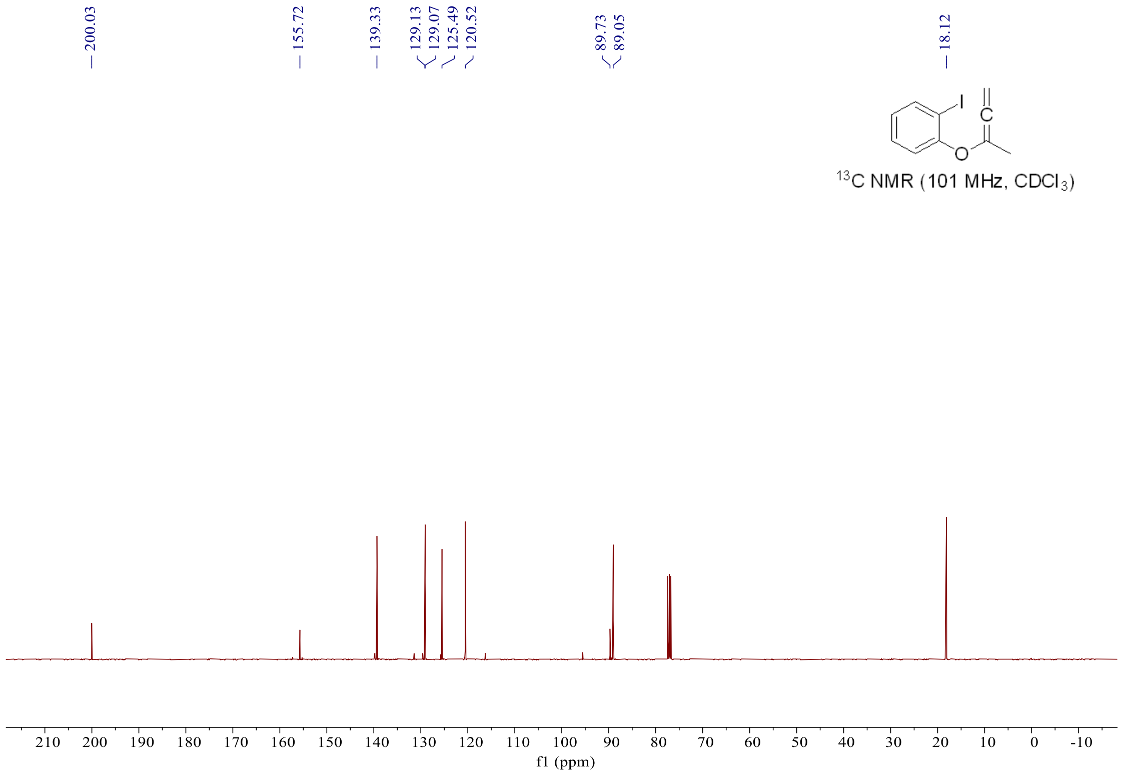

Supplementary Figure 33. ^{1}H and ^{13}C NMR spectra of **1l.**

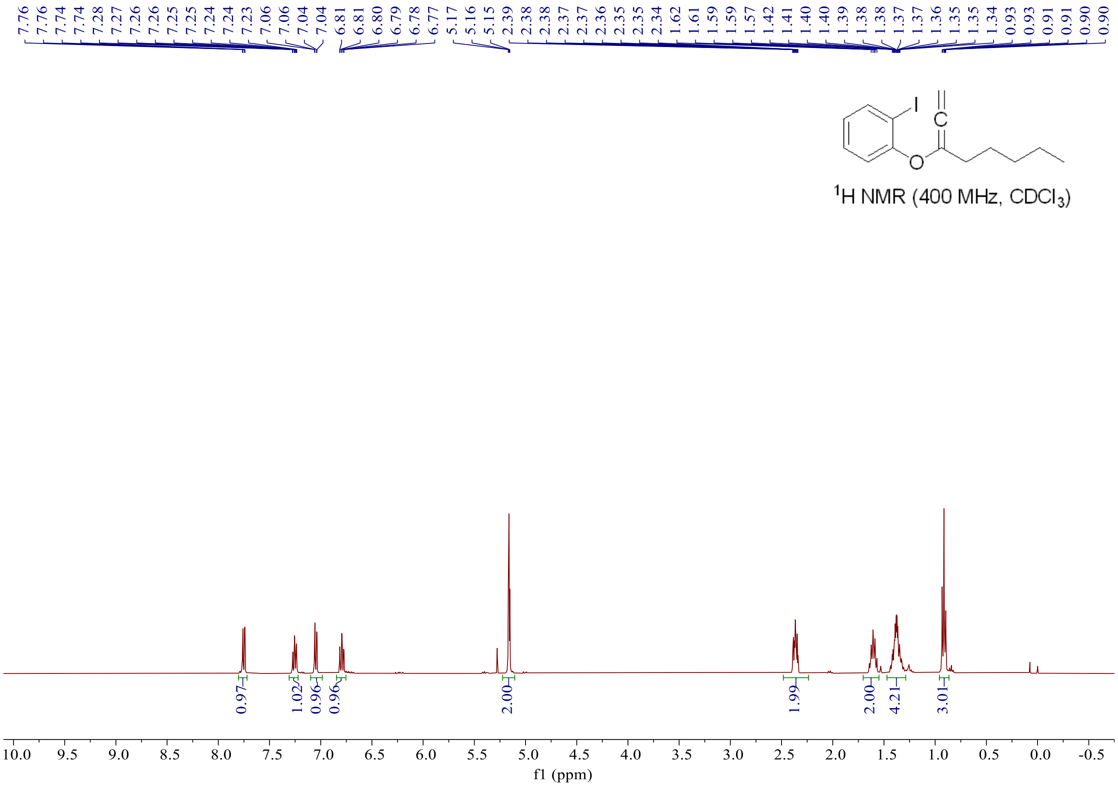

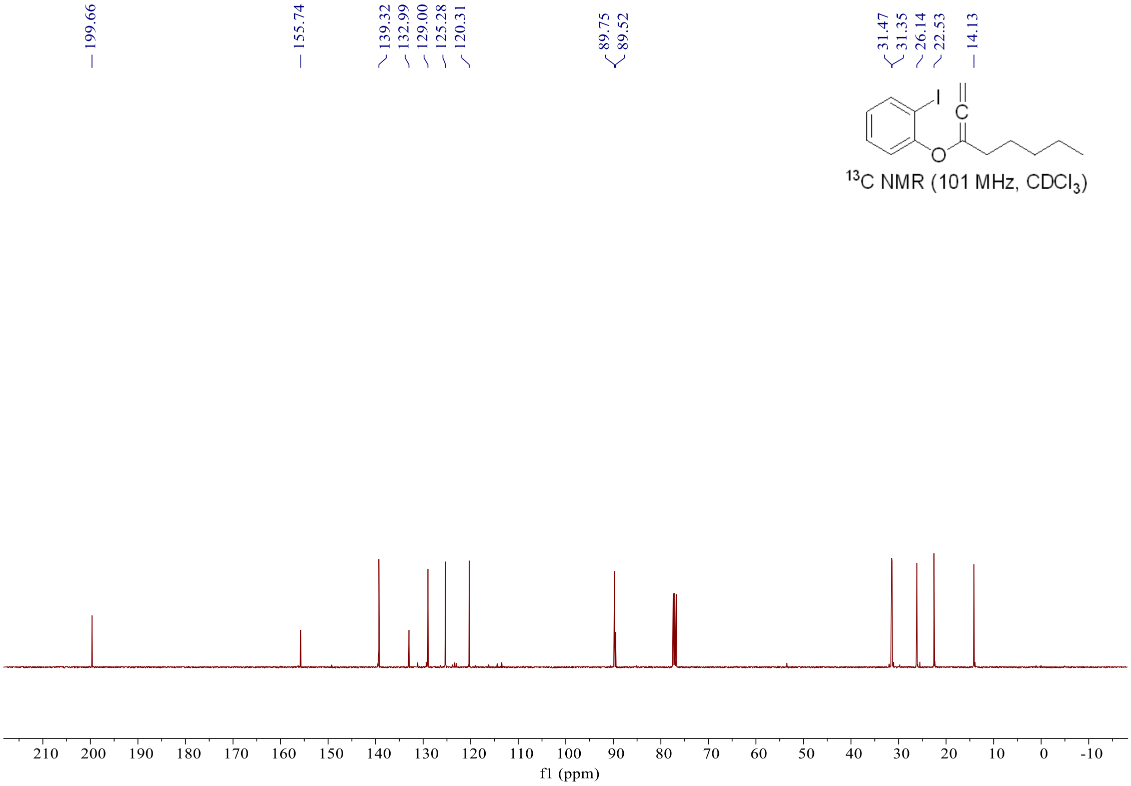

Supplementary Figure 34. ^{1}H and ^{13}C NMR spectra of **1m**.

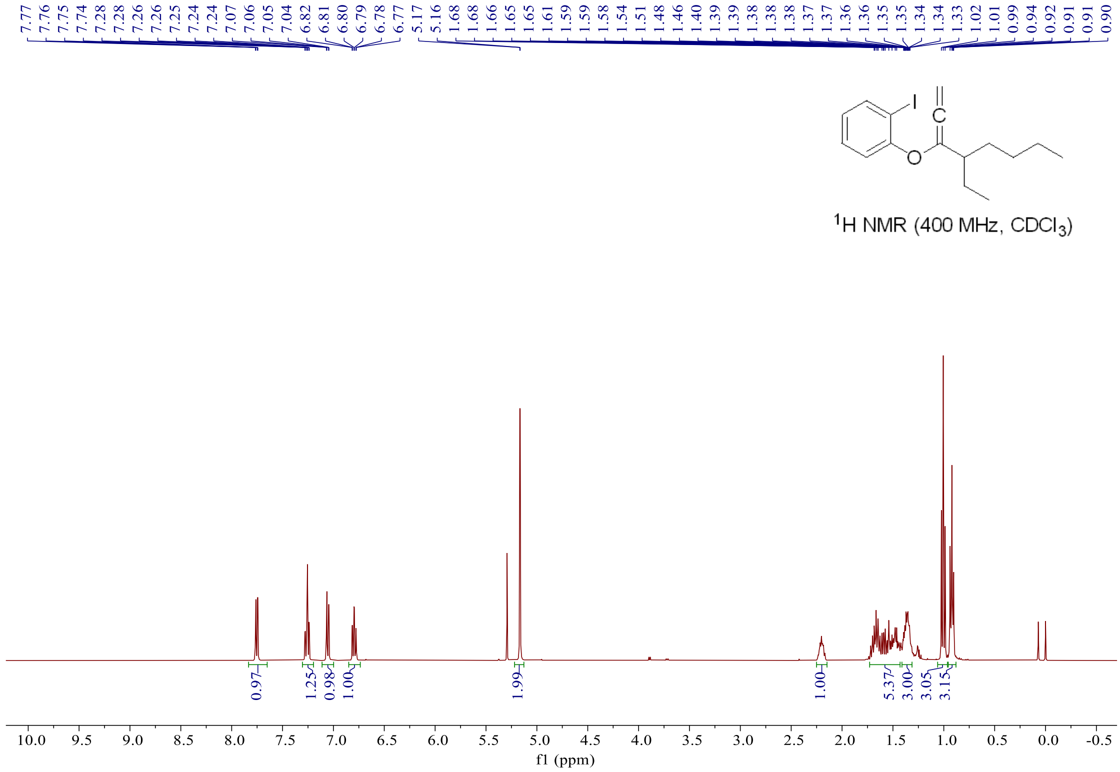

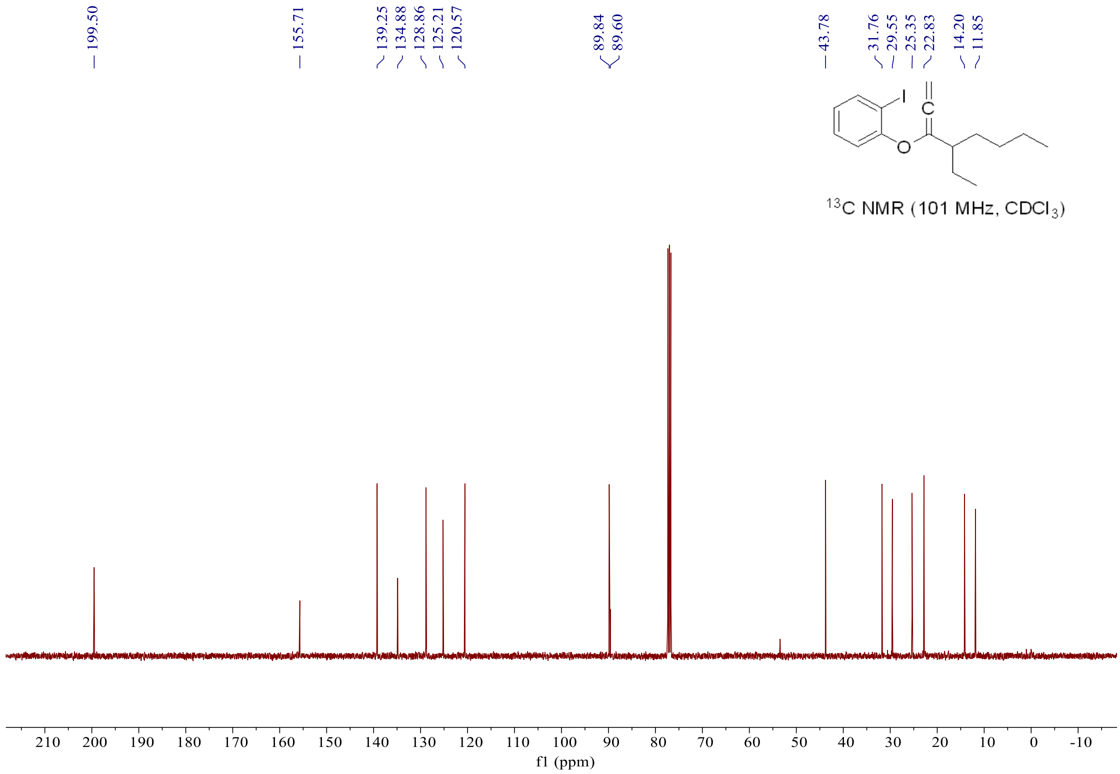

Supplementary Figure 35. ^{1}H, ^{13}C and ^{31}P NMR spectra of **3a**.

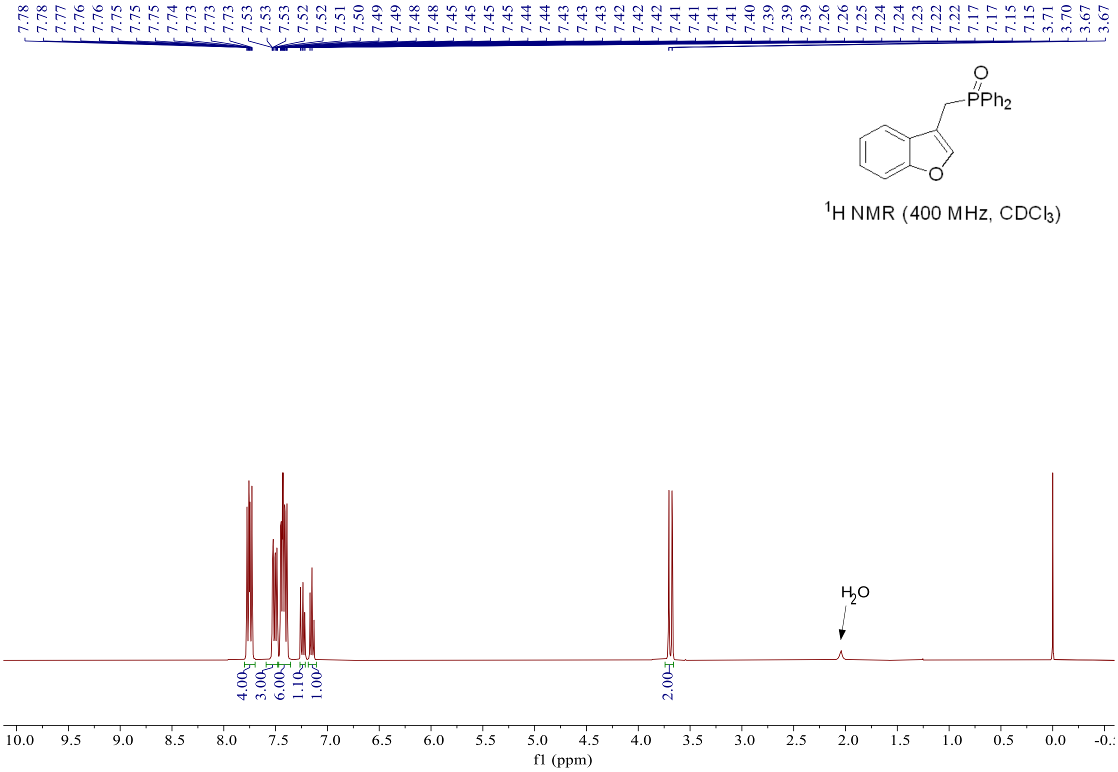

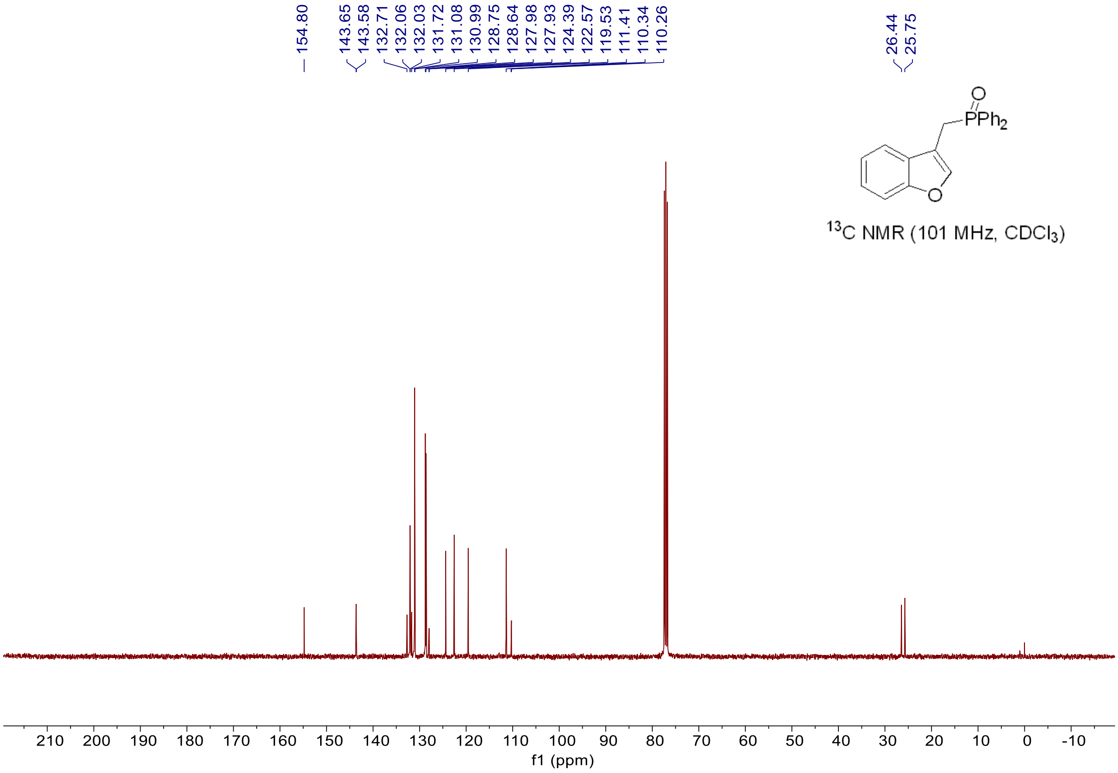

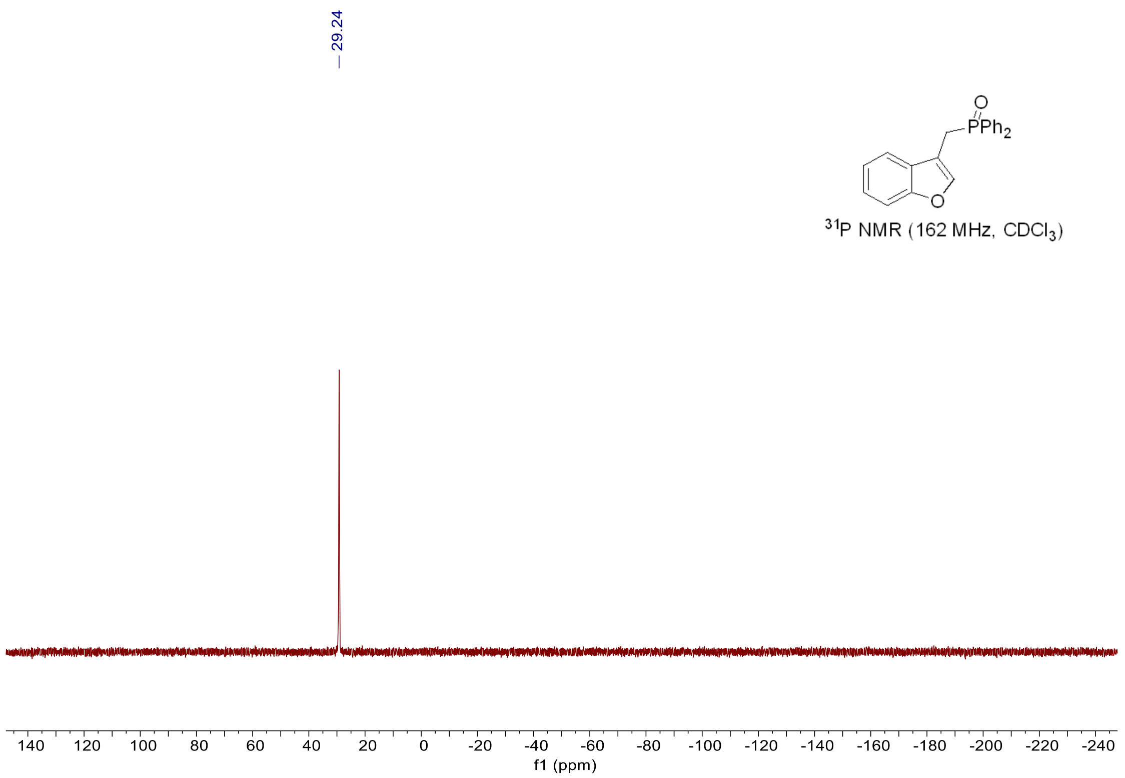

The tall peak around 2 is from water

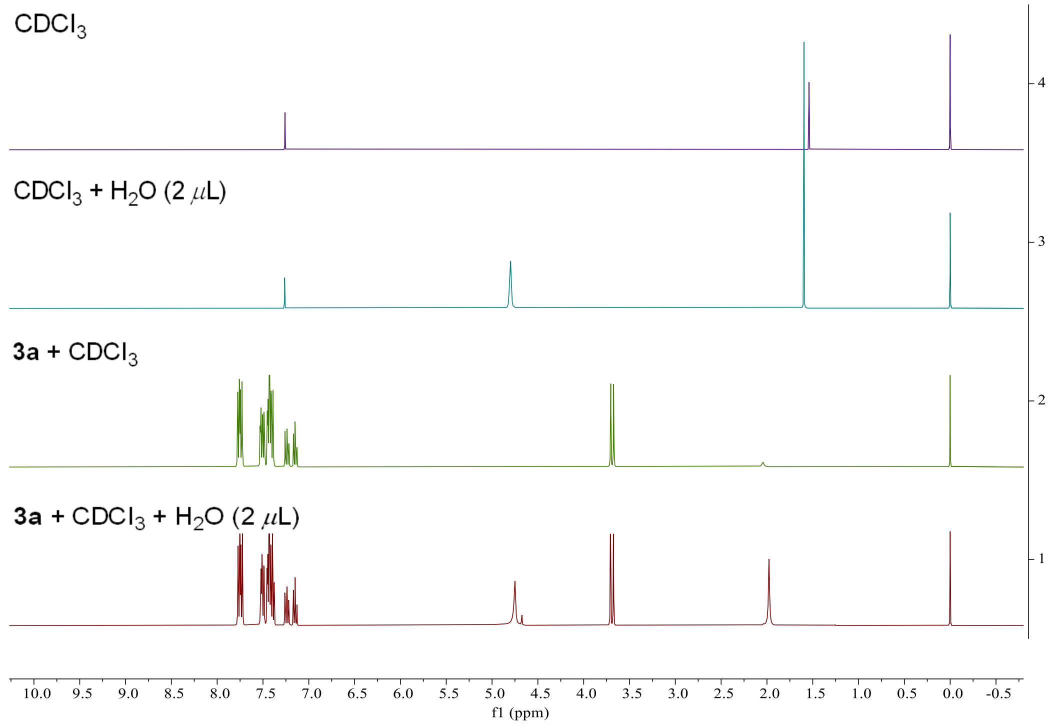

Supplementary Figure 36. ^{1}H, ^{13}C and ^{31}P NMR spectra of **3b**.

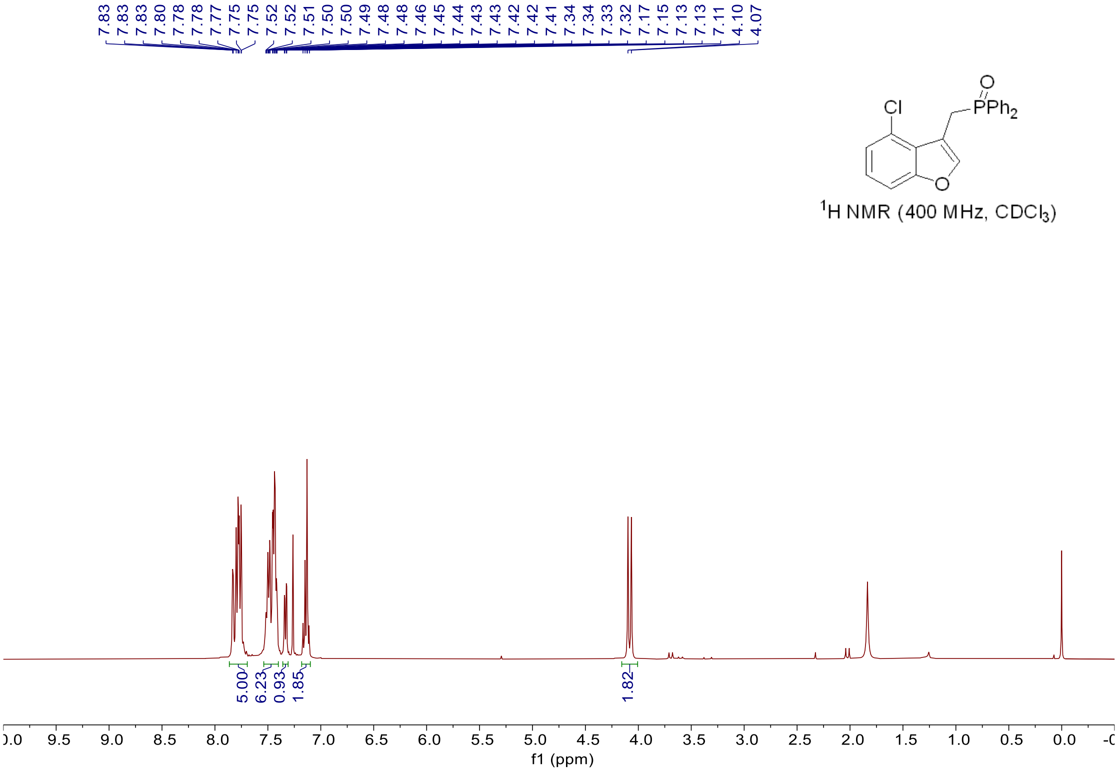

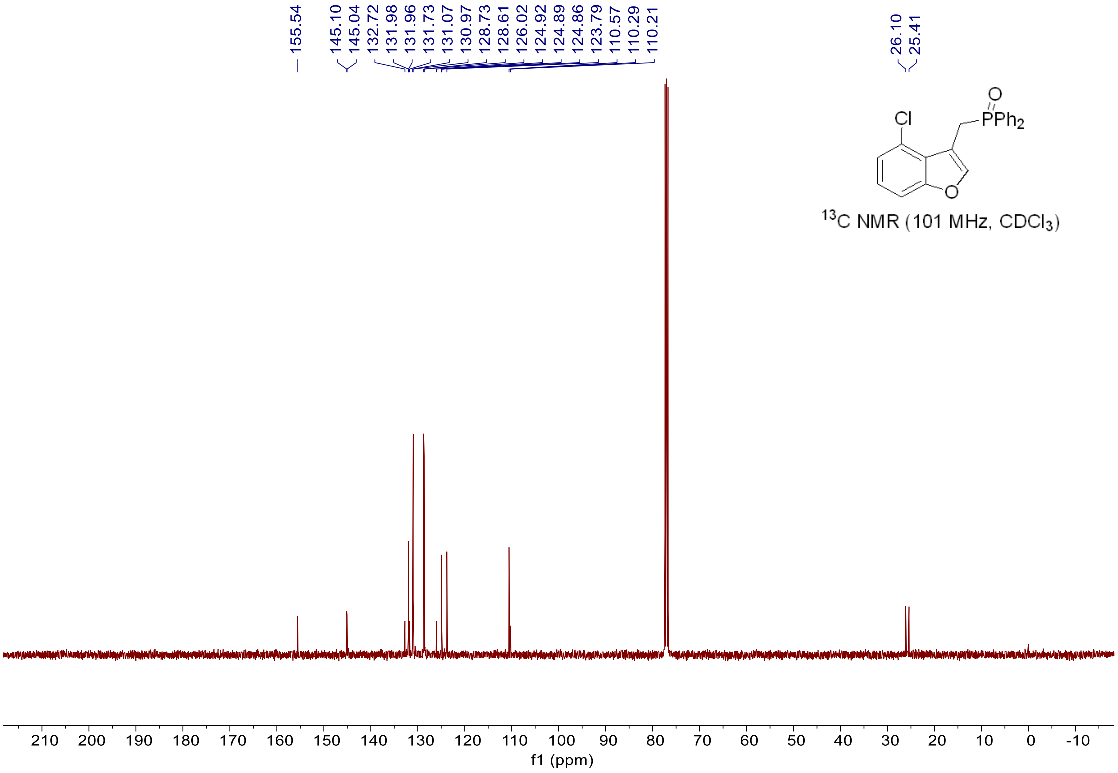

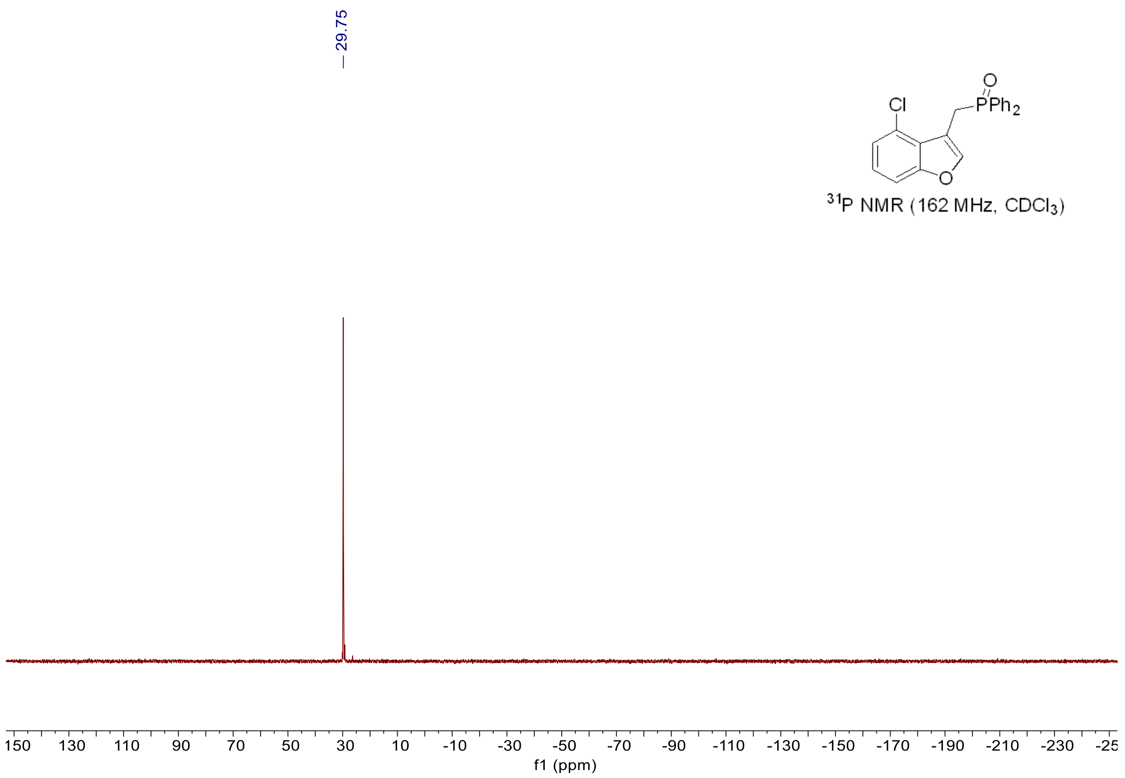
29.75
Cl
O
PPh2
O
31P NMR (162 MHz, CDCl3)
150
130
110
90
70
50
30
10
-10
-30
-50
-70
-90
-110
-130
-150
-170
-190
-210
-230
f1 (ppm)

Supplementary Figure 37. ^{1}H, ^{13}C, ^{19}F and ^{31}P NMR spectra of **3c**.

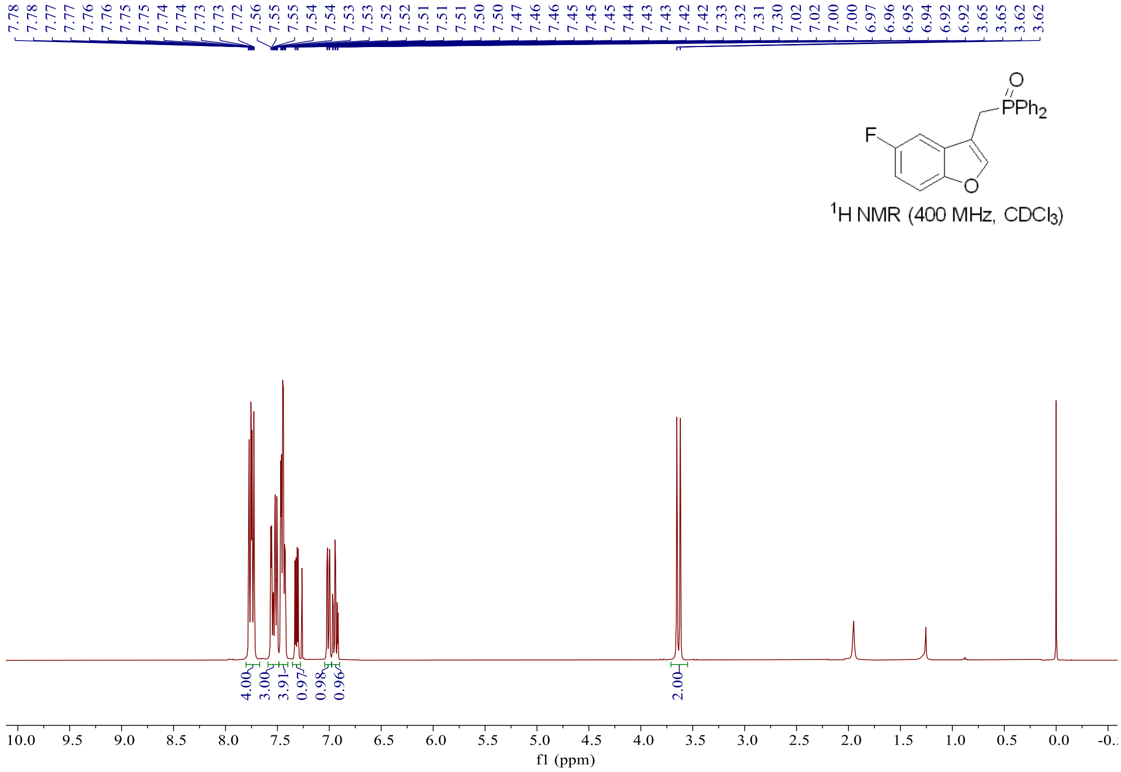

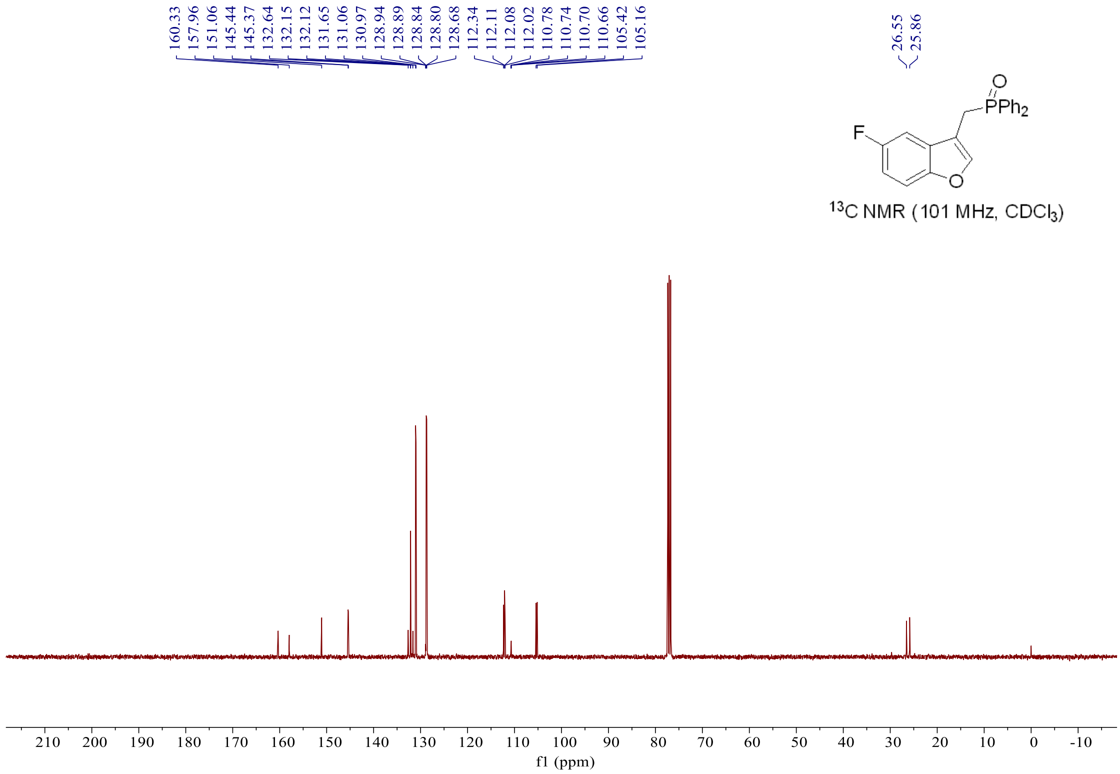

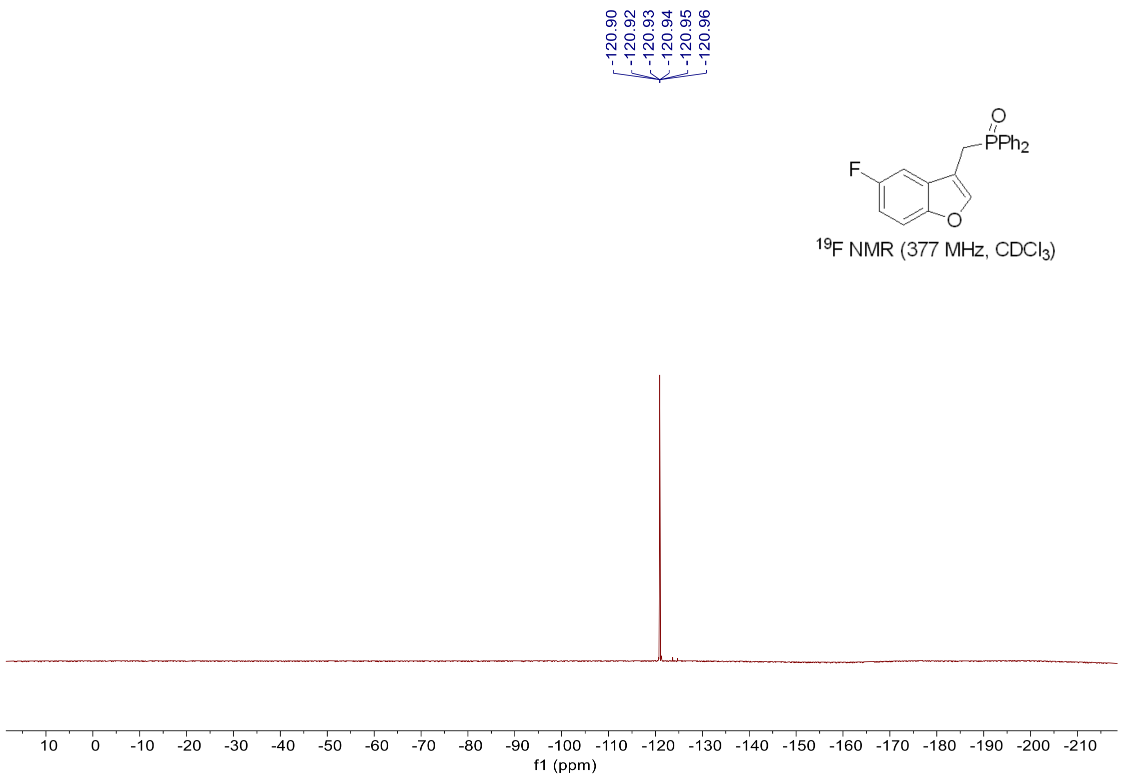

-120.90
-120.92
-120.93
-120.94
-120.95
-120.96
O
PPh2
F
O
19F NMR (377 MHz, CDCl3)
10 0 -10 -20 -30 -40 -50 -60 -70 -80 -90 -100 -110 -120 -130 -140 -150 -160 -170 -180 -190 -200 -210
f1 (ppm)

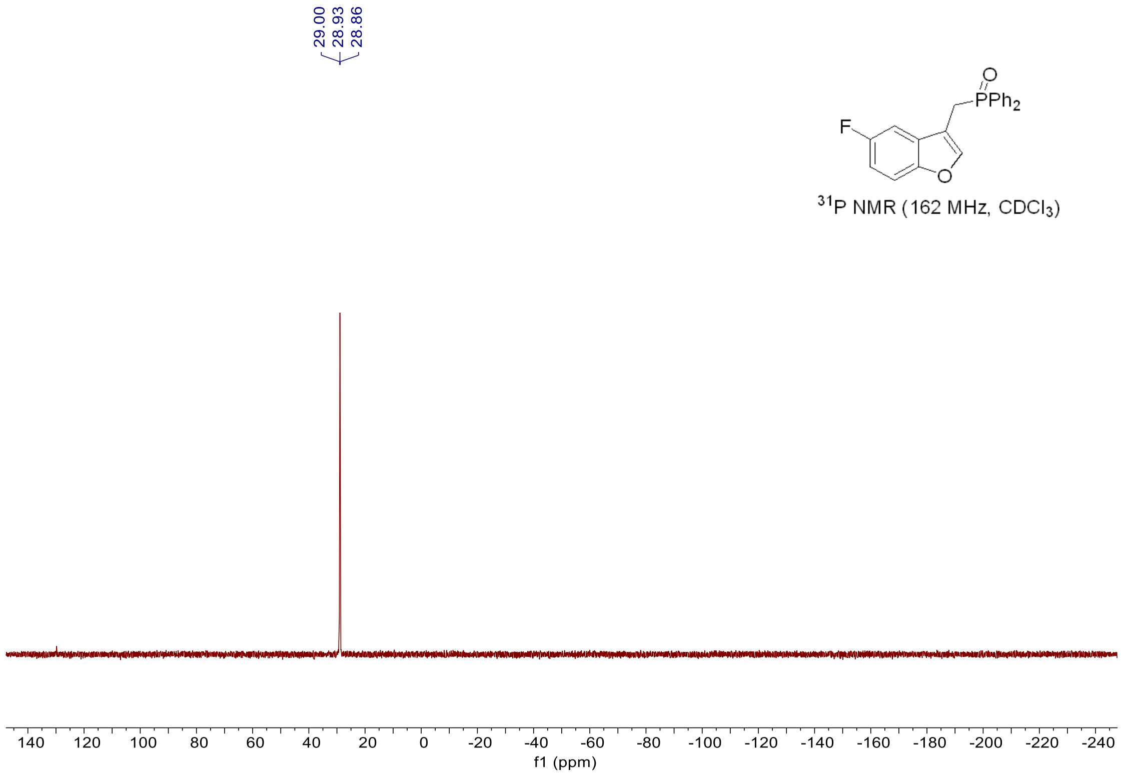

29.00
28.93
28.86
O
PPh2
F
O
31P NMR (162 MHz, CDCl3)
140 120 100 80 60 40 20 0 -20 -40 -60 -80 -100 -120 -140 -160 -180 -200 -220 -240
f1 (ppm)

Supplementary Figure 38. ^{1}H, ^{13}C and ^{31}P NMR spectra of **3d**.

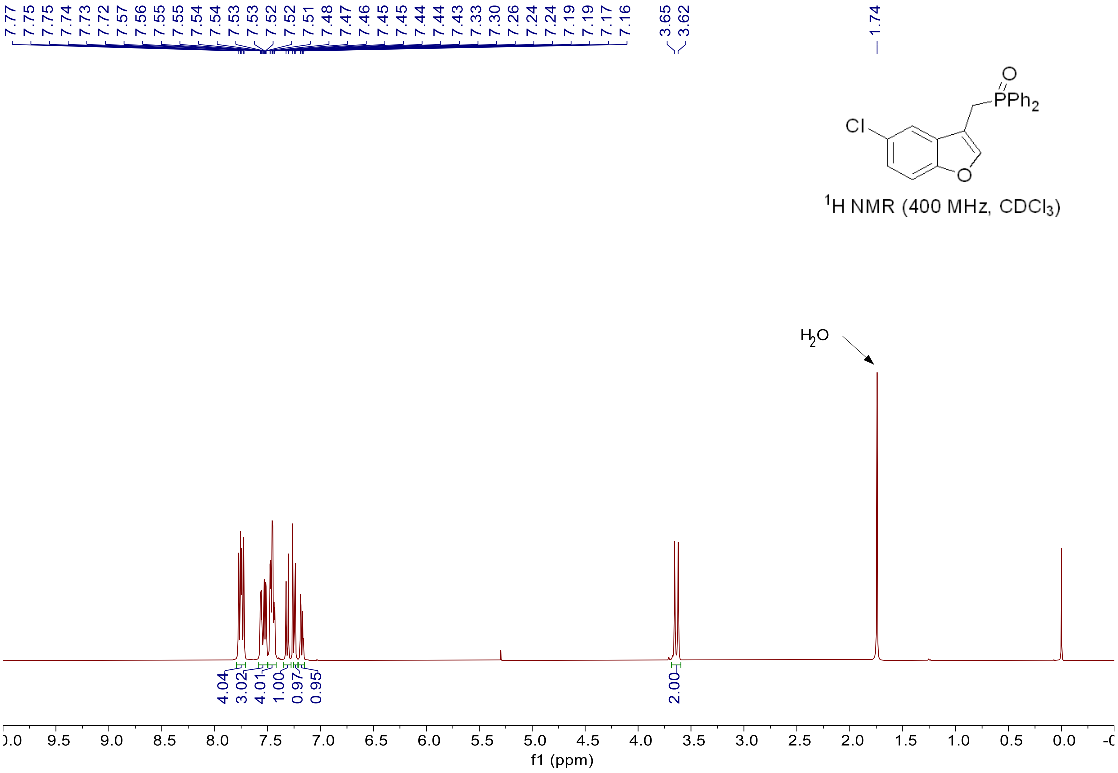

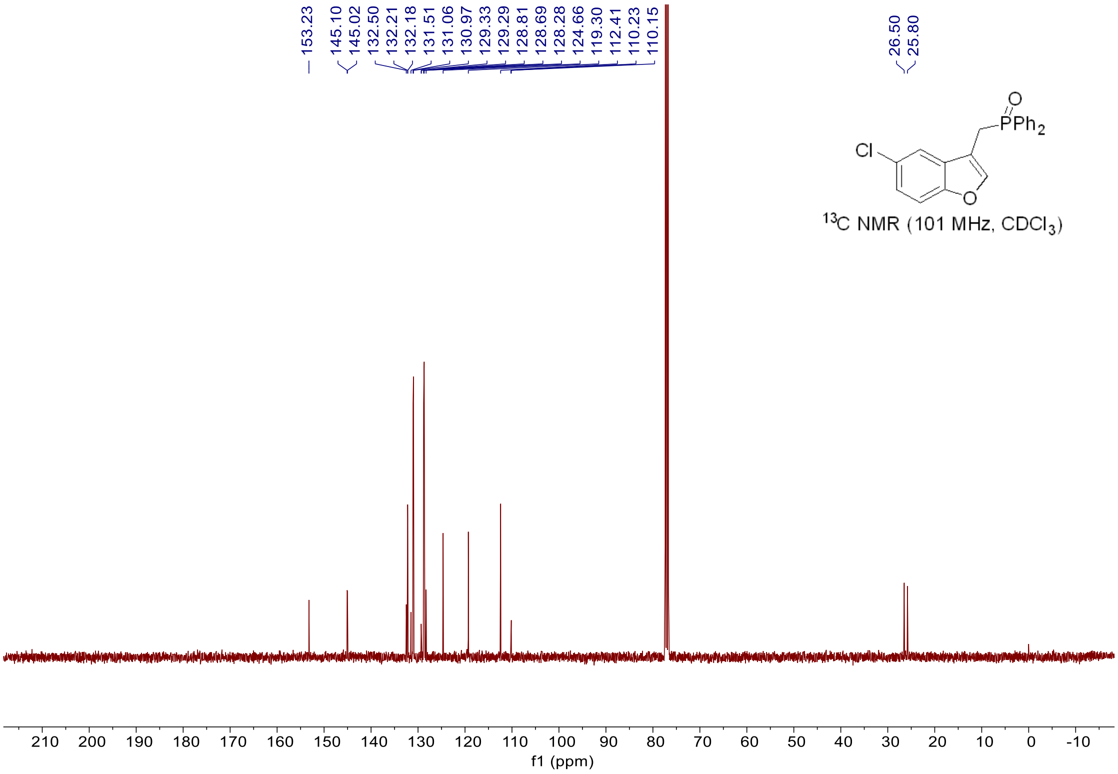

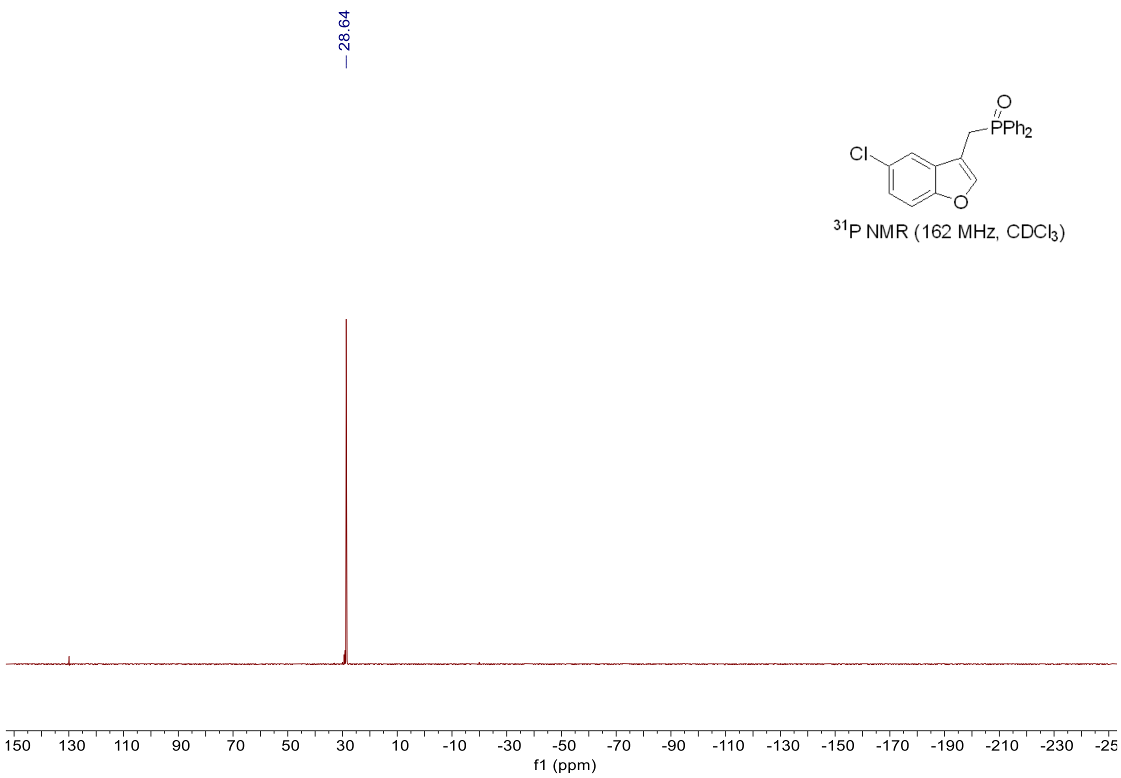

28.64
O
PPh2
Cl
O
^{31}P NMR (162 MHz, CDCl3)
150 130 110 90 70 50 30 10 -10 -30 -50 -70 -90 -110 -130 -150 -170 -190 -210 -230 -25
f1 (ppm)

Supplementary Figure 39. ^{1}H, ^{13}C, ^{19}F and ^{31}P NMR spectra of **3e**.

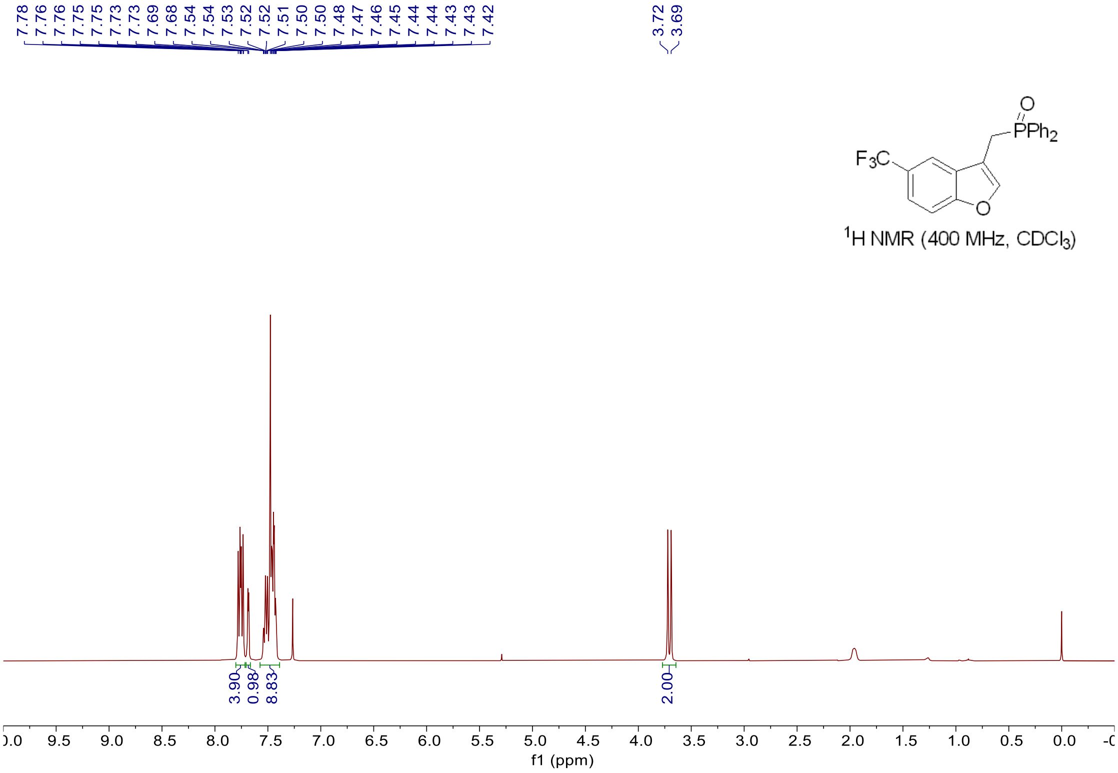

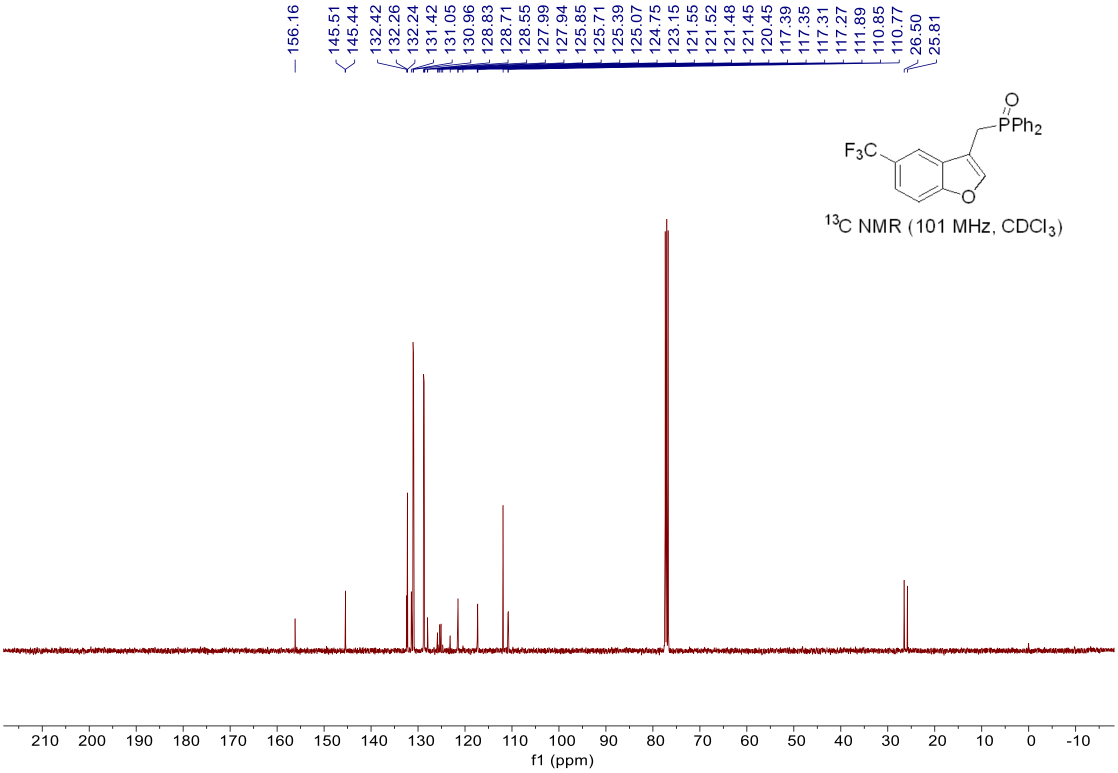

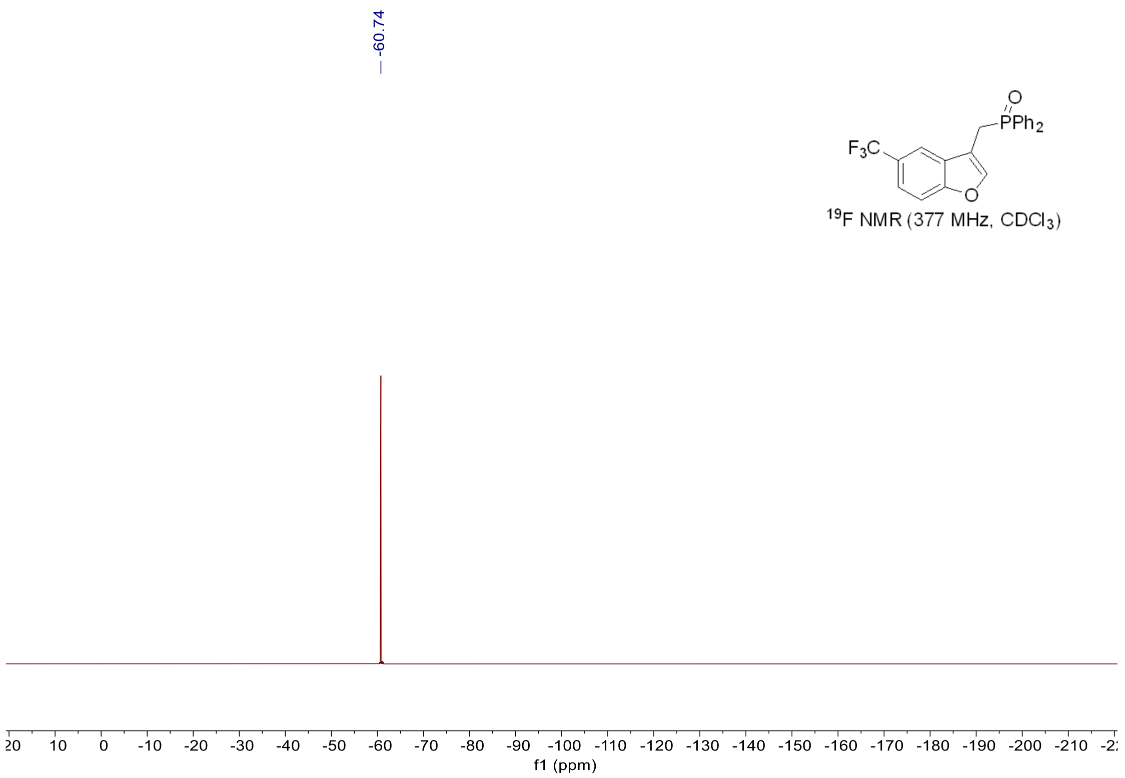
-60.74
F_3C
PPh2
O
19F NMR (377 MHz, CDCl3)
f1 (ppm)

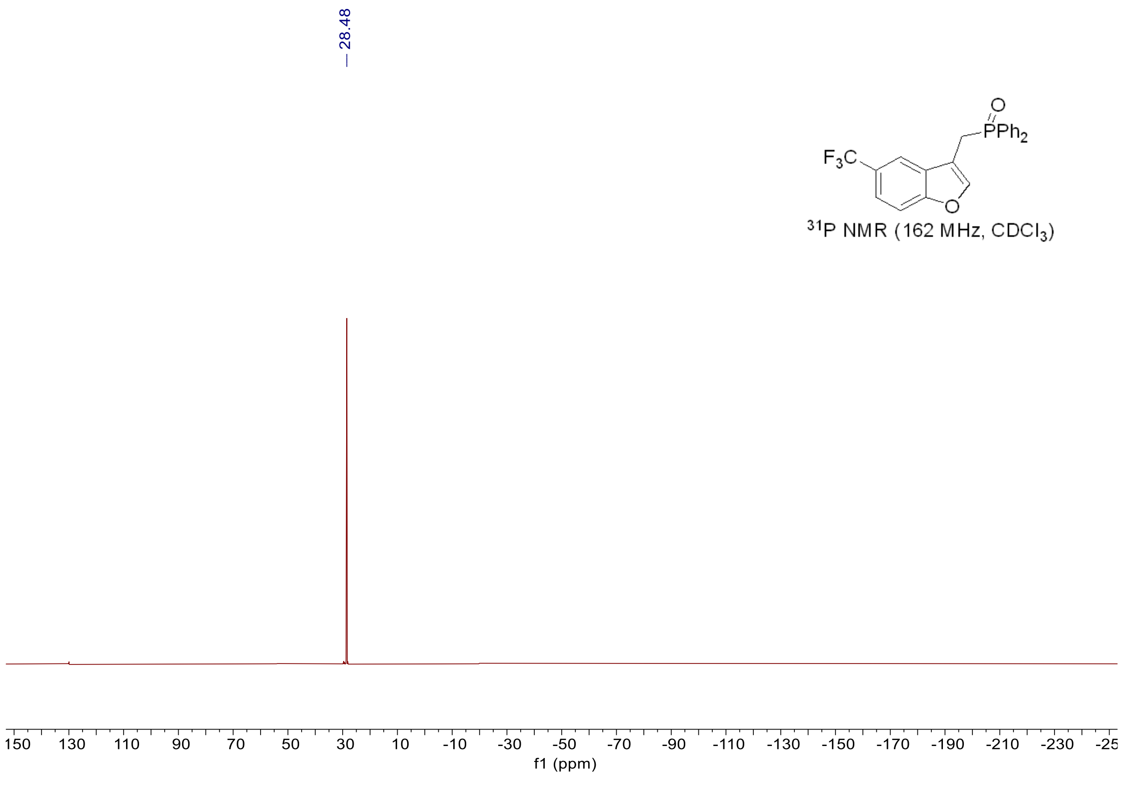
28.48
F_3C
PPh2
O
31P NMR (162 MHz, CDCl3)
f1 (ppm)

Supplementary Figure 40. ^{1}H, ^{13}C and ^{31}P NMR spectra of **3f.**

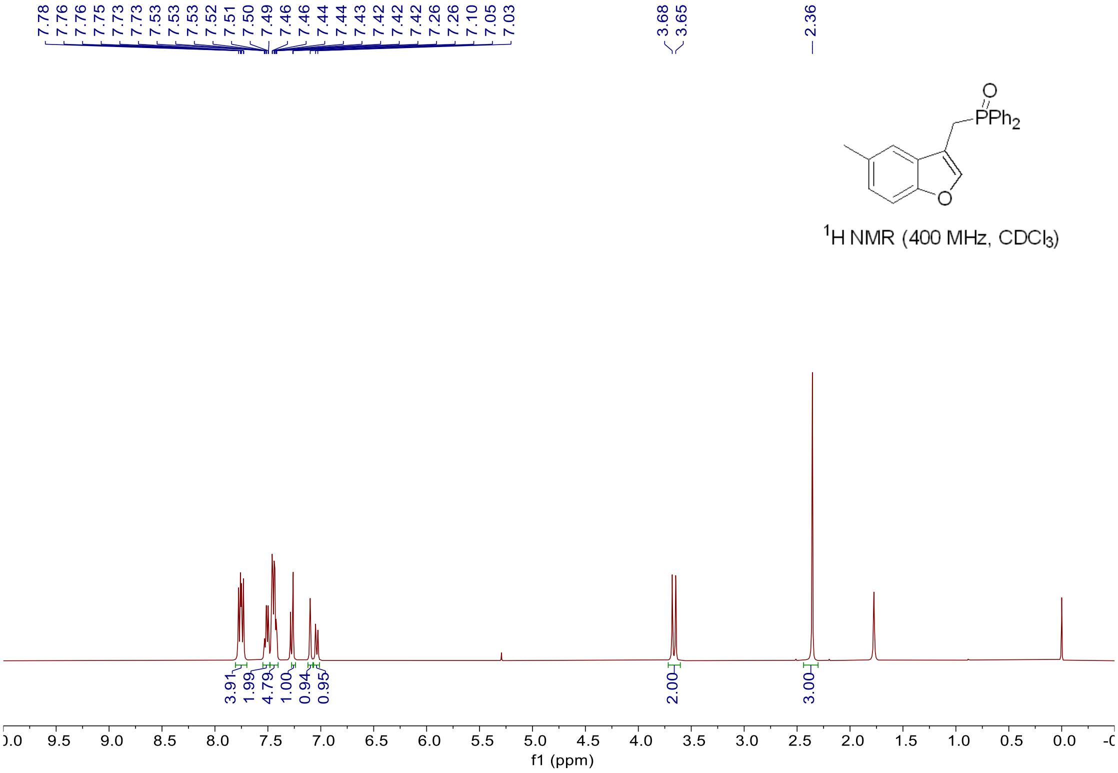

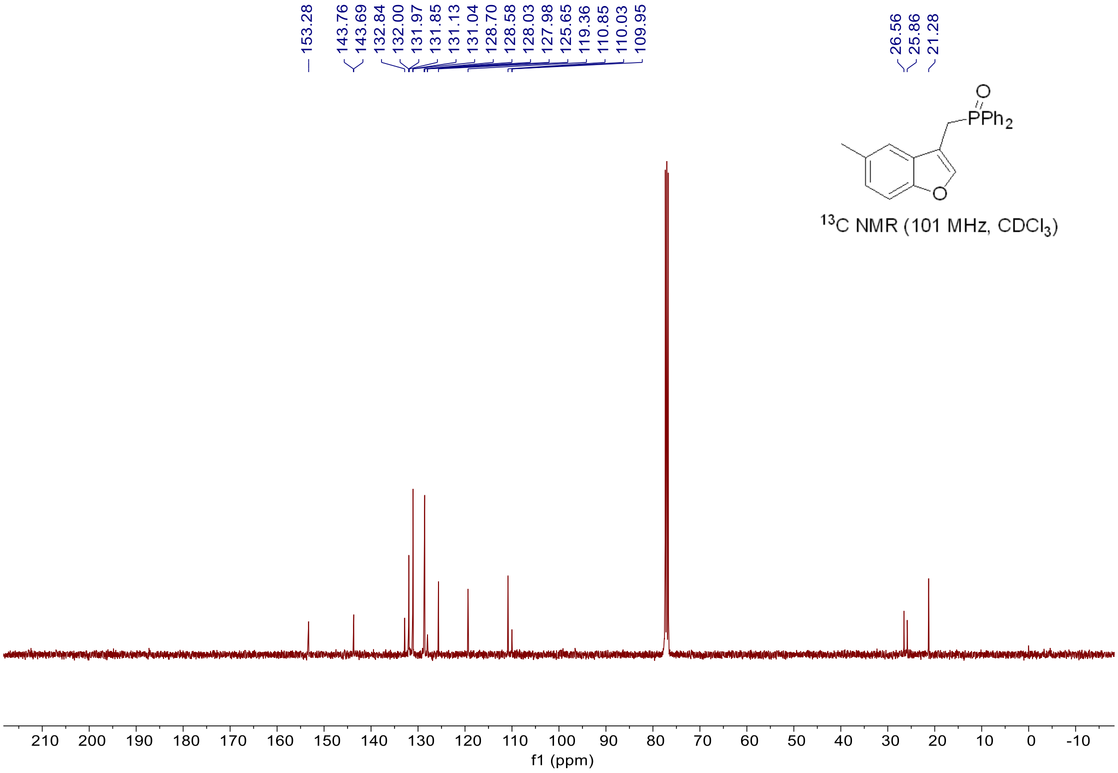

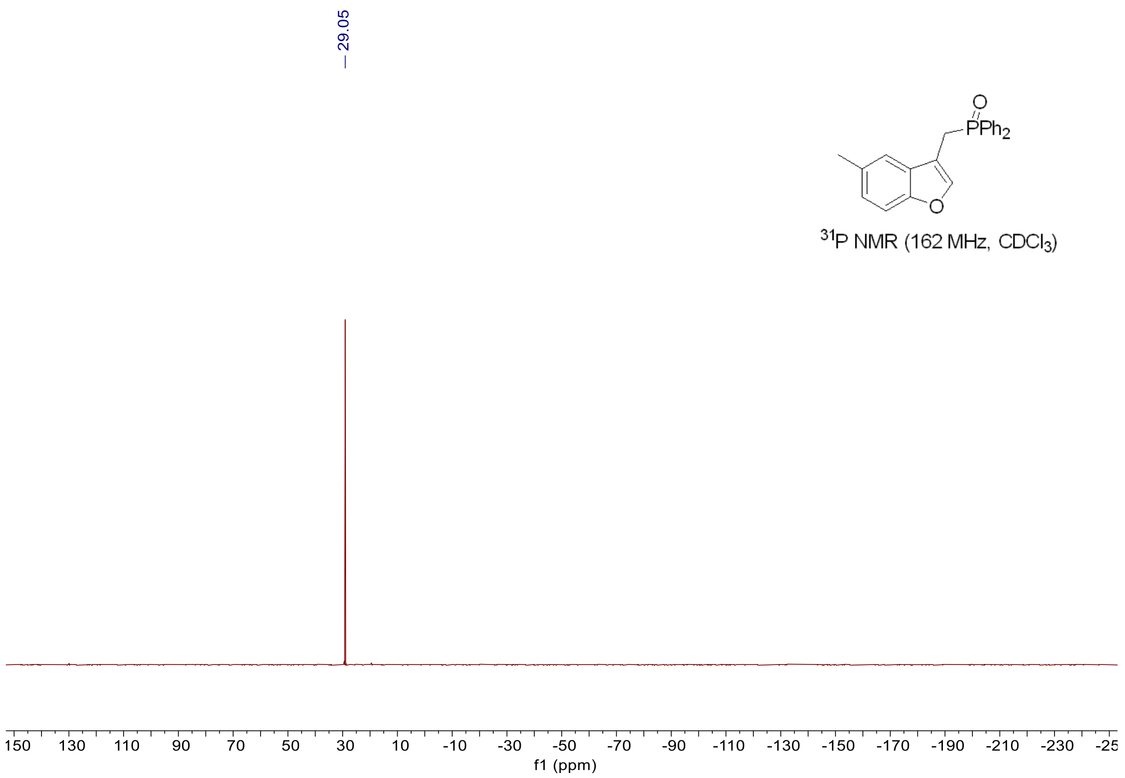

29.05
PPh2
O
31P NMR (162 MHz, CDCl3)
150 130 110 90 70 50 30 10 -10 -30 -50 -70 -90 -110 -130 -150 -170 -190 -210 -230 -25
f1 (ppm)

Supplementary Figure 41. ^{1}H, ^{13}C and ^{31}P NMR spectra of **3g**.

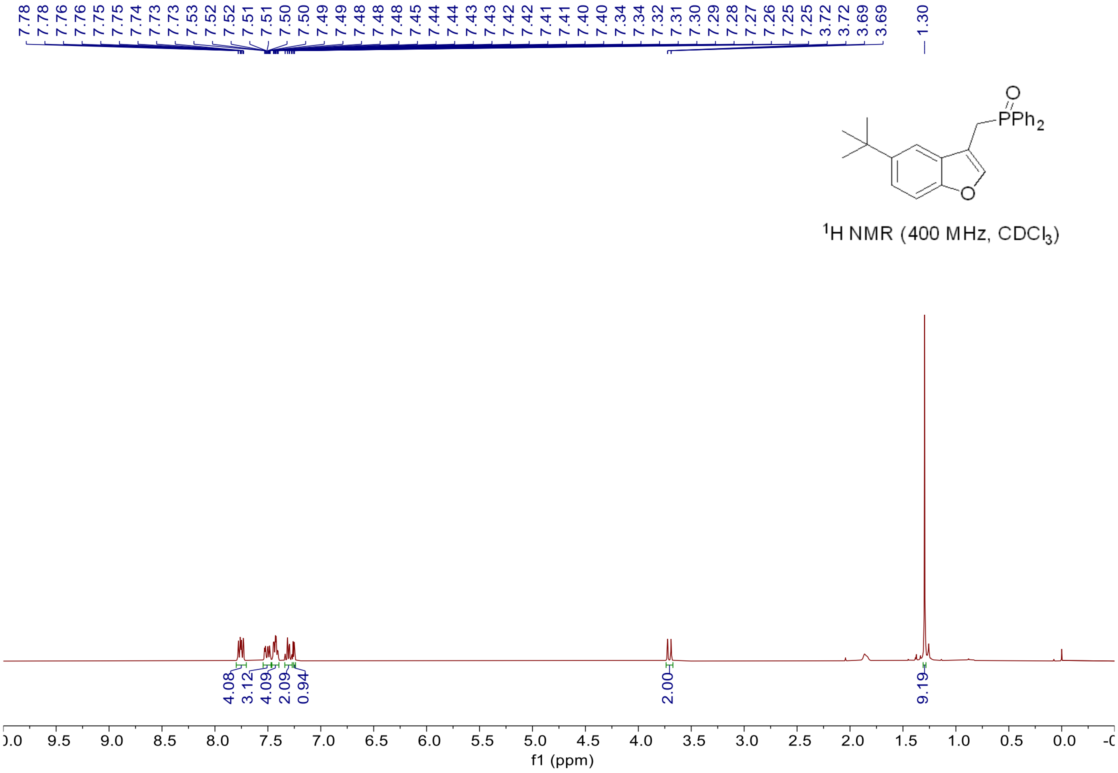

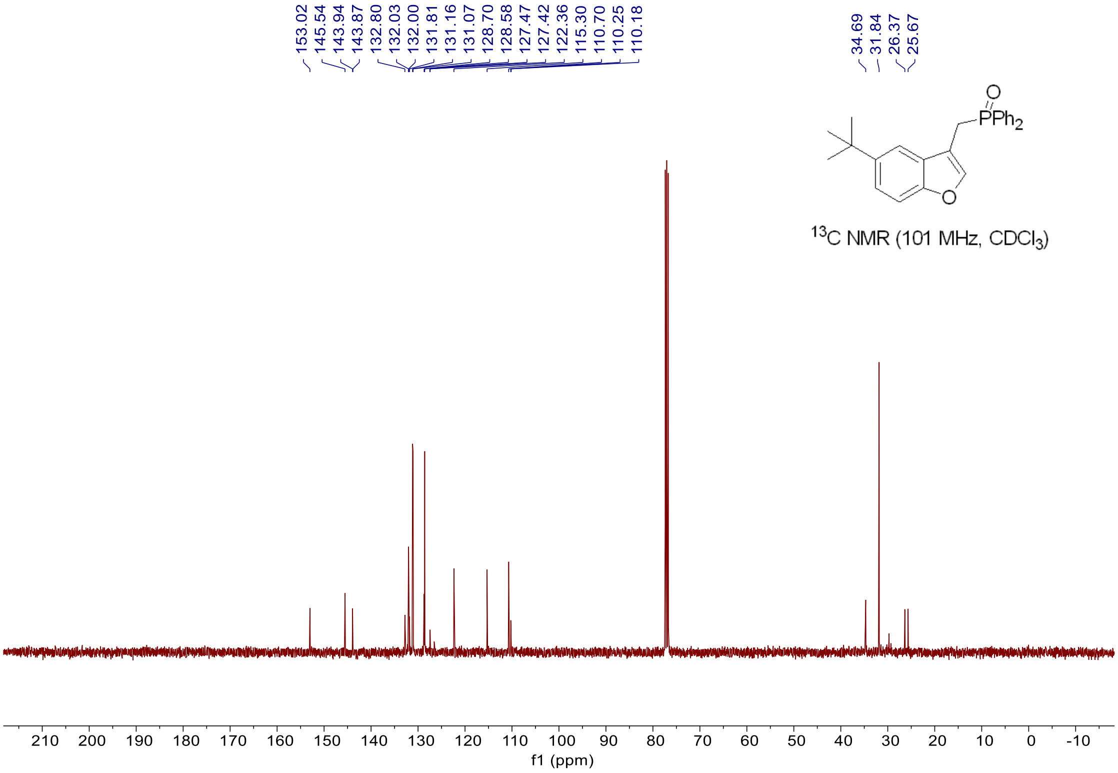

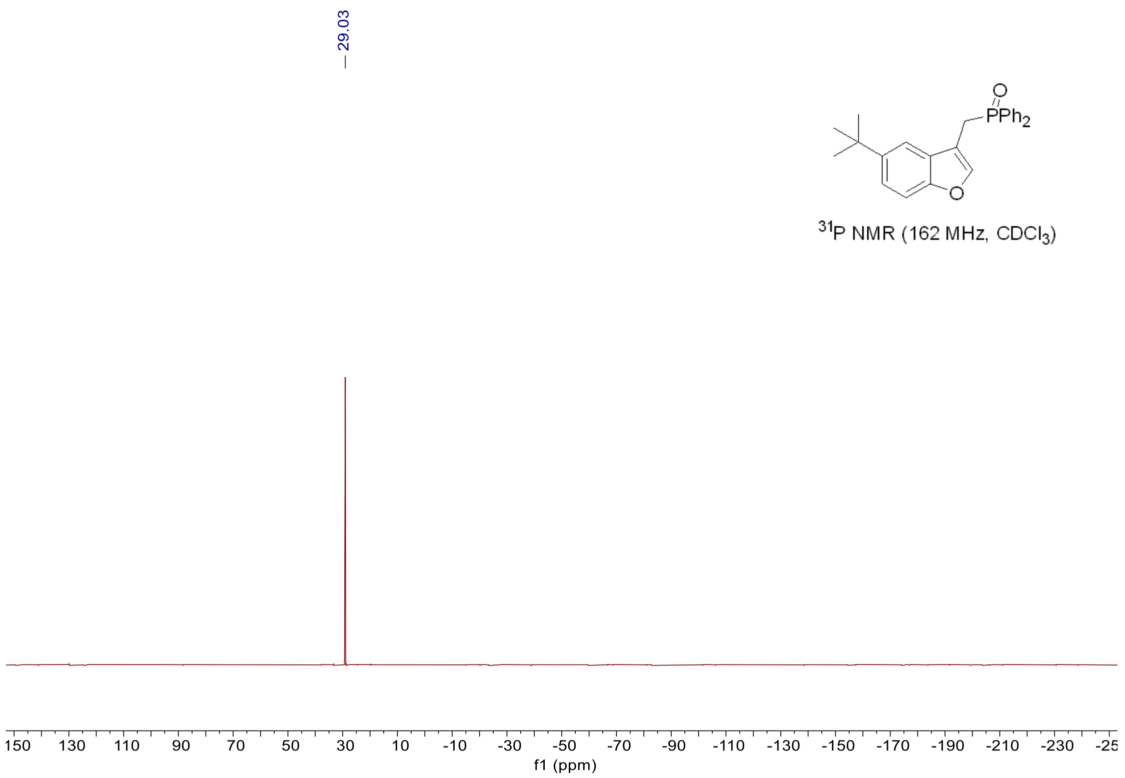
29.03
PPh2
O
31P NMR (162 MHz, CDCl3)
150
130
110
90
70
50
30
10
-10
-30
-50
-70
-90
-110
-130
-150
-170
-190
-210
-230
f1 (ppm)

Supplementary Figure 42. ^{1}H, ^{13}C and ^{31}P NMR spectra of **3h.**

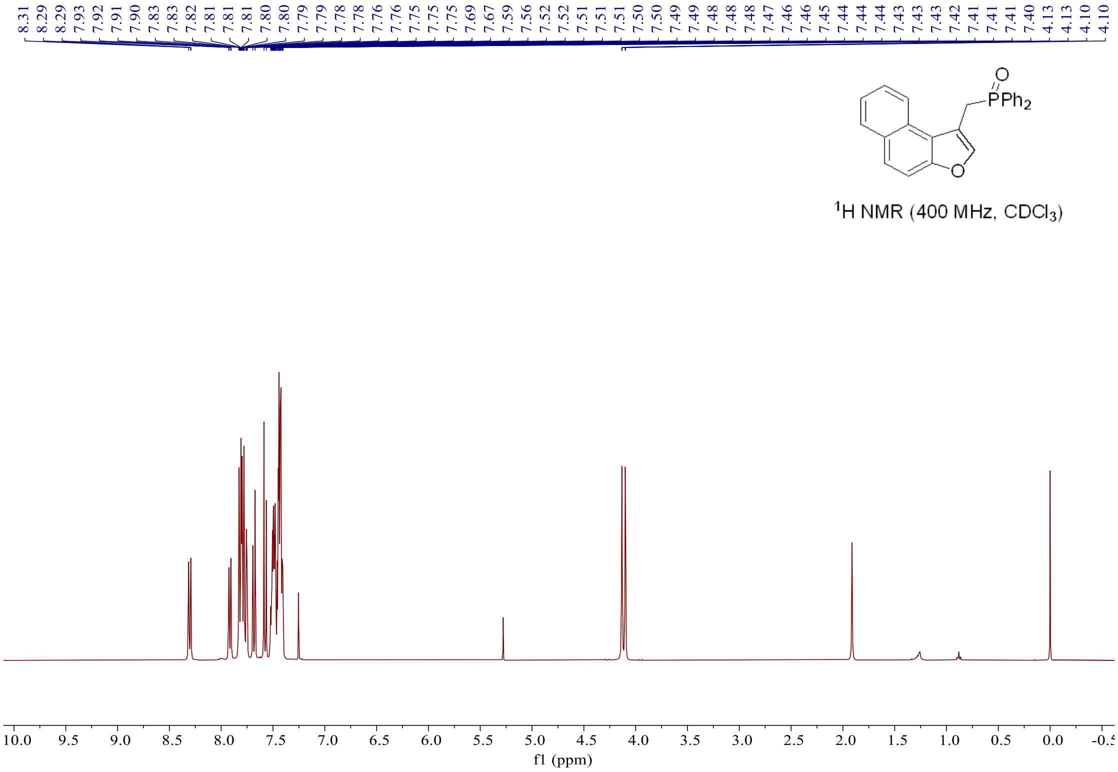

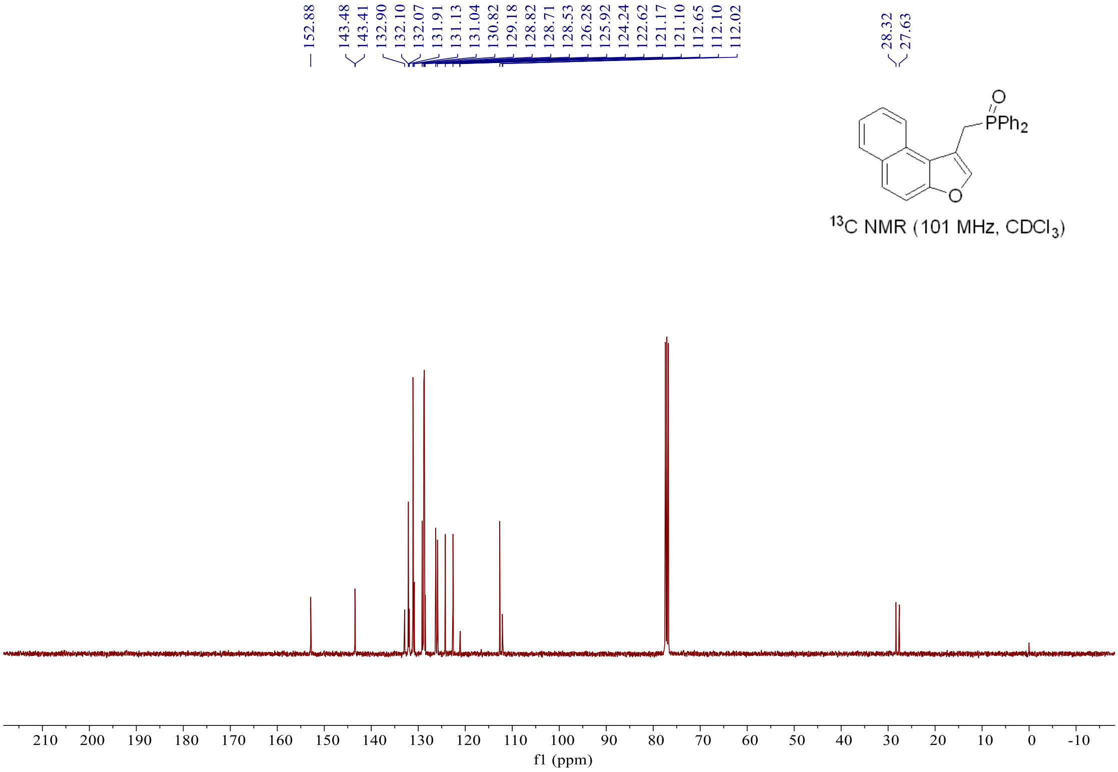

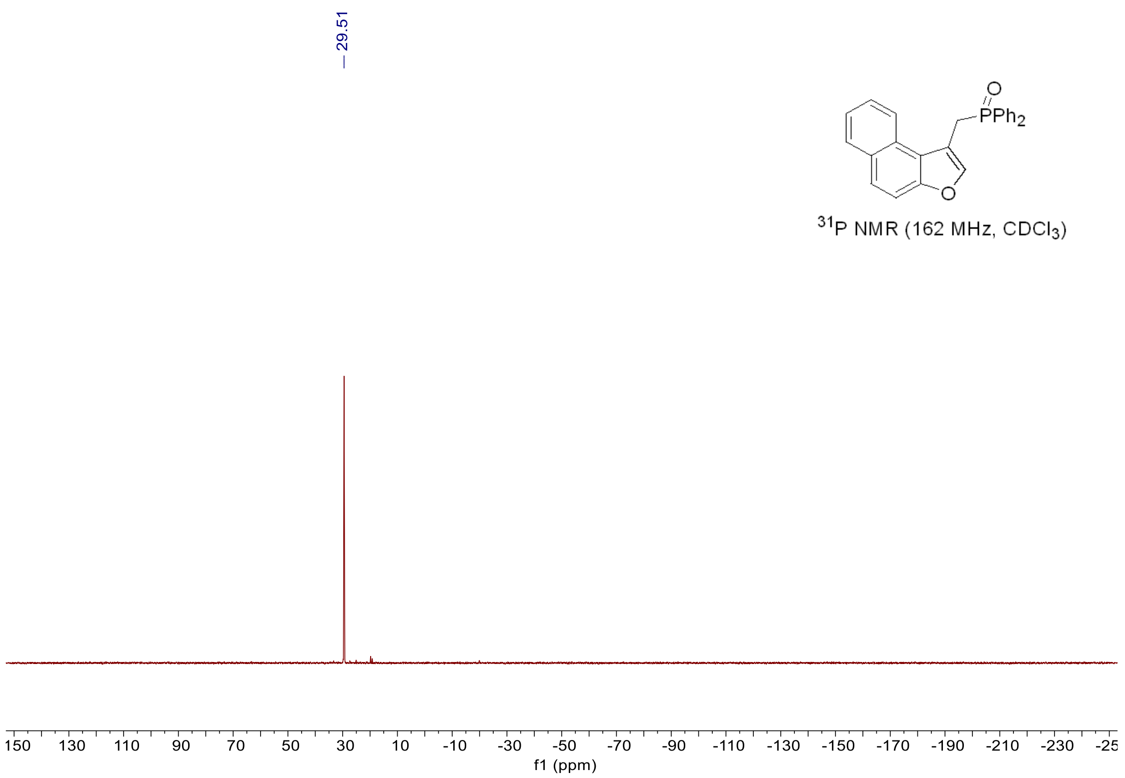
29.51
PPh2
O
31P NMR (162 MHz, CDCl3)
150 130 110 90 70 50 30 10 -10 -30 -50 -70 -90 -110 -130 -150 -170 -190 -210 -230 -25
f1 (ppm)

Supplementary Figure 43. ^{1}H, ^{13}C and ^{31}P NMR spectra of **3i**.

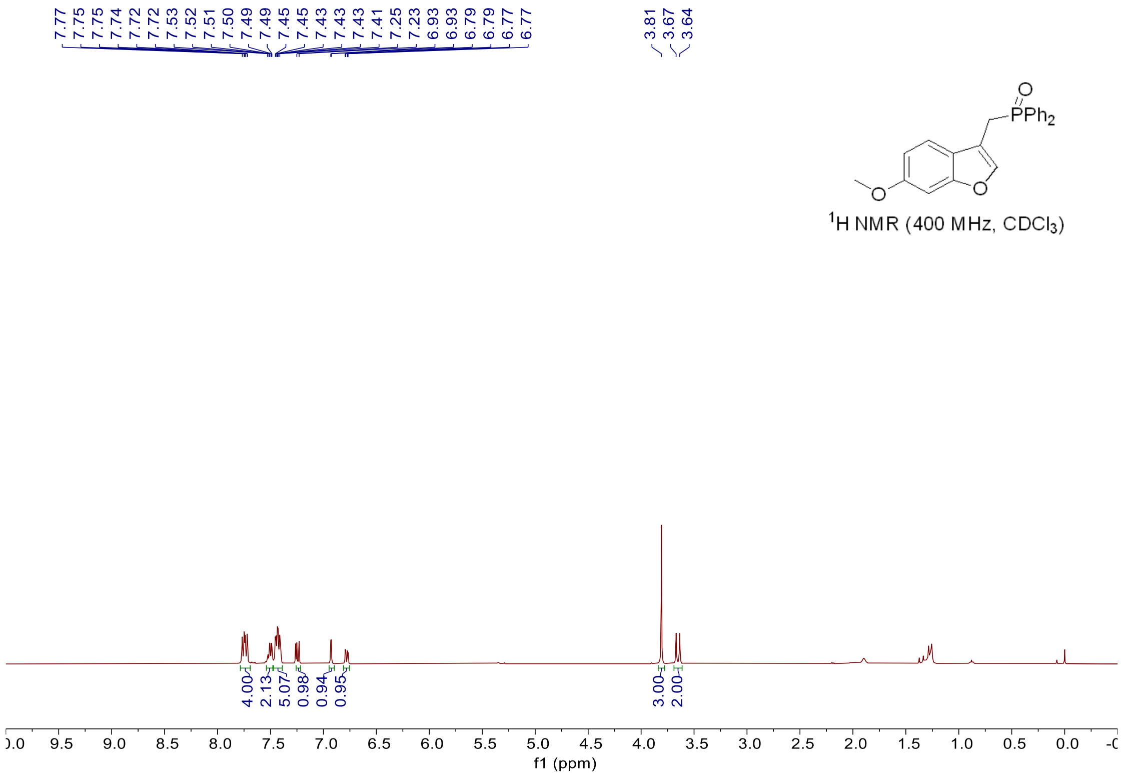

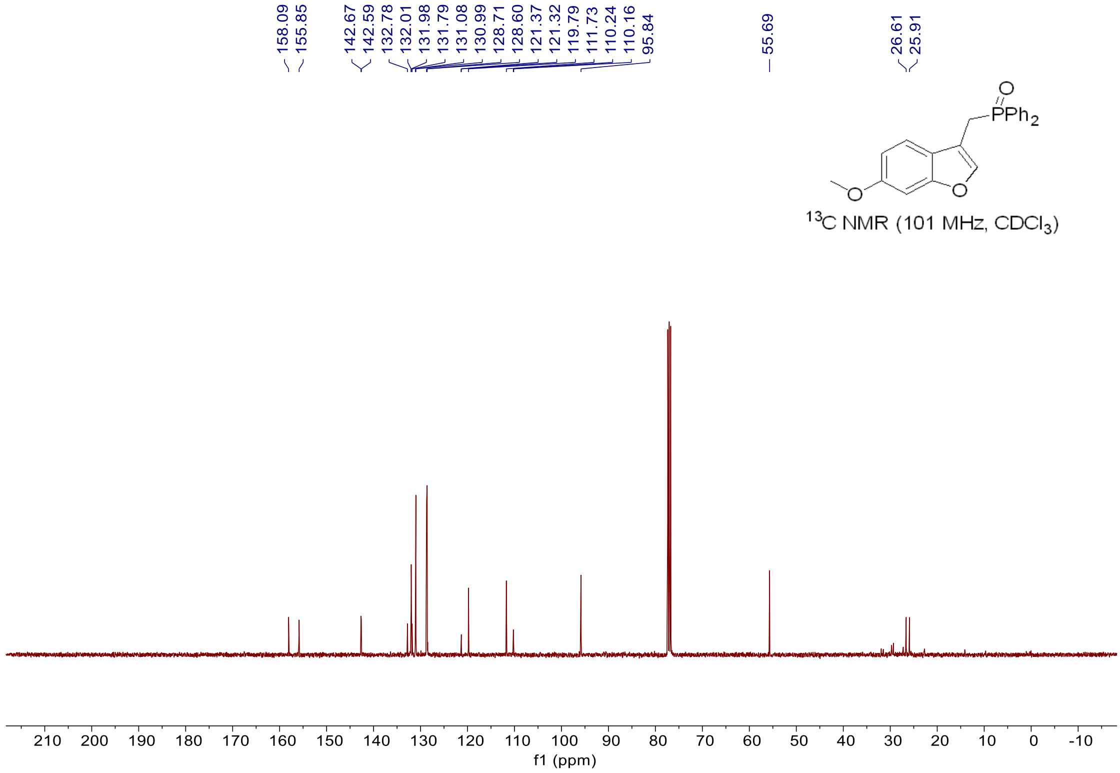

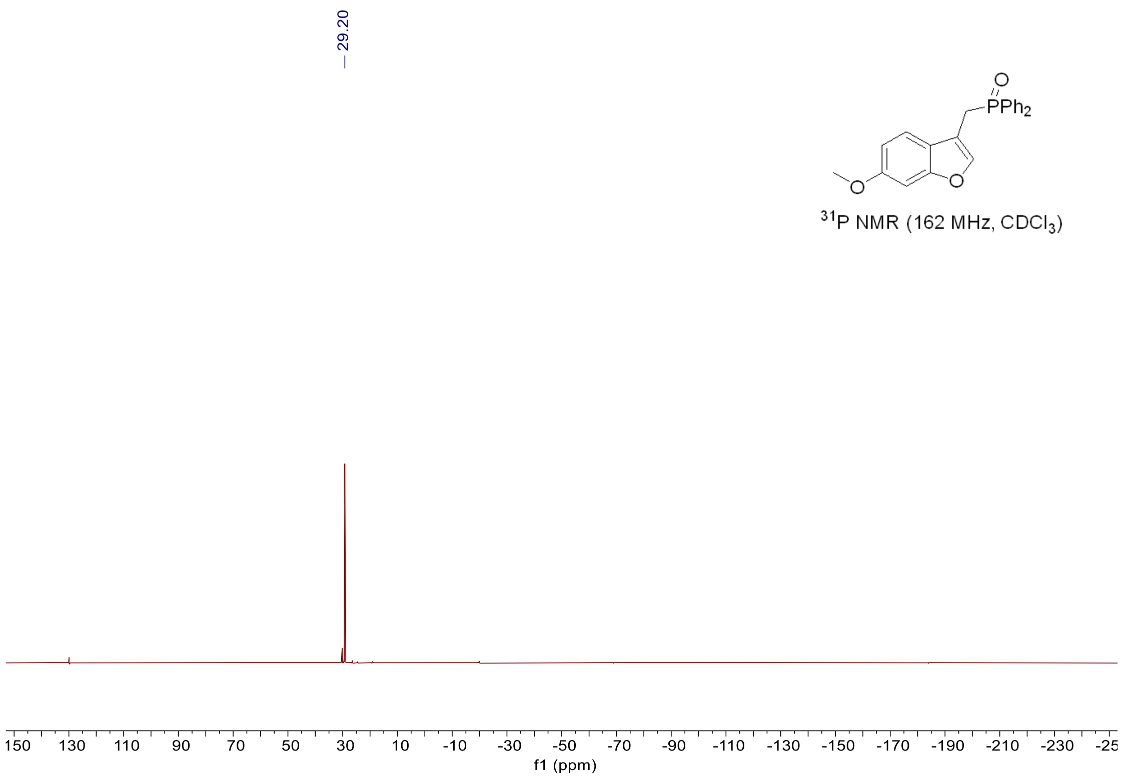
29.20
O
PPh2
O
O
31P NMR (162 MHz, CDCl3)
150 130 110 90 70 50 30 10 -10 -30 -50 -70 -90 -110 -130 -150 -170 -190 -210 -230 -25
f1 (ppm)

Supplementary Figure 44. ^{1}H, ^{13}C and ^{31}P NMR spectra of **3j**.

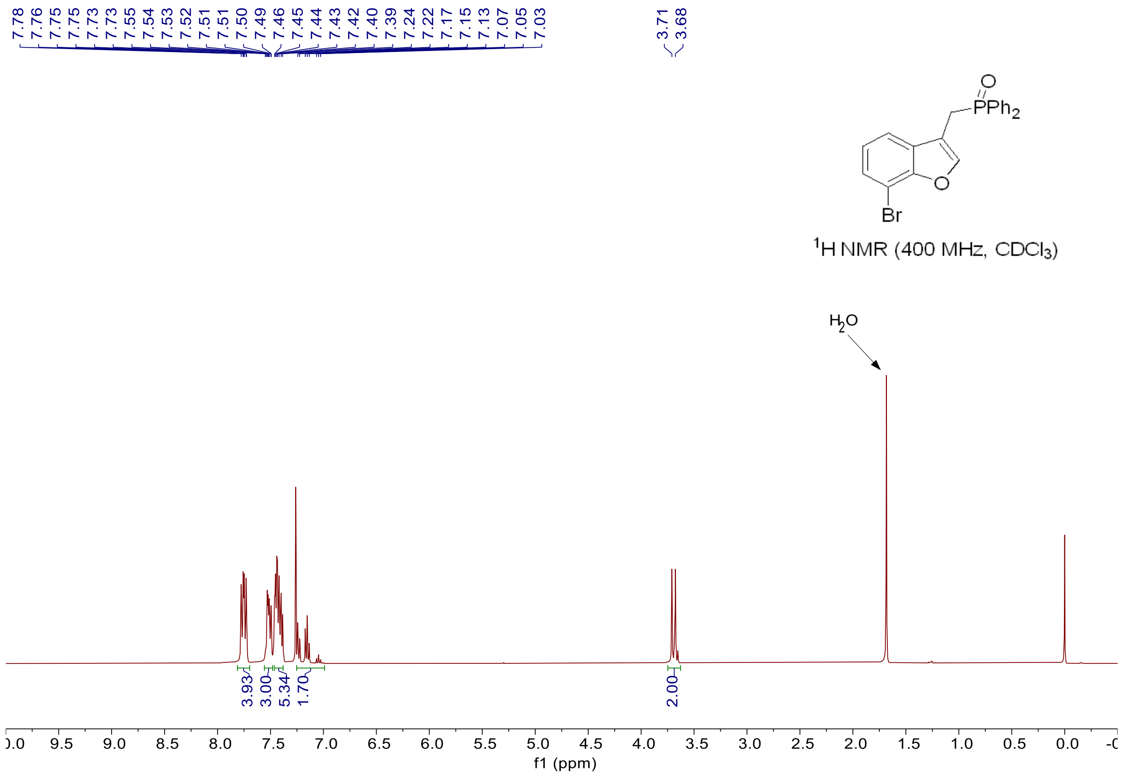

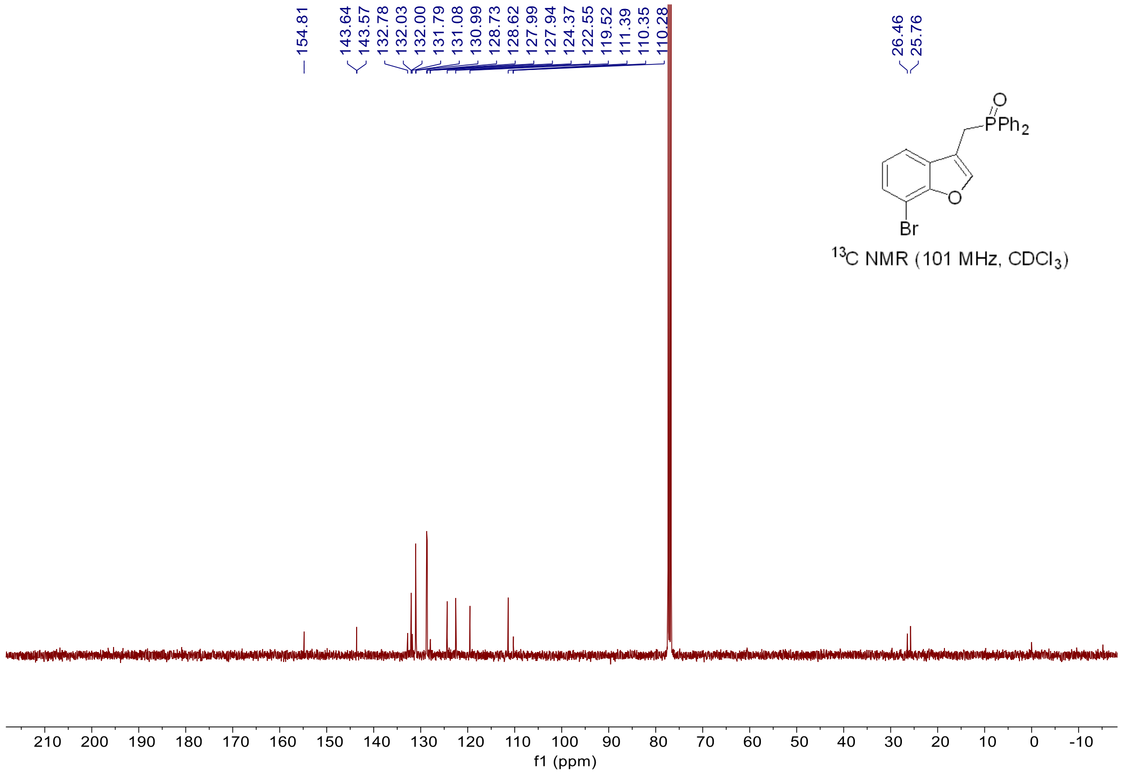

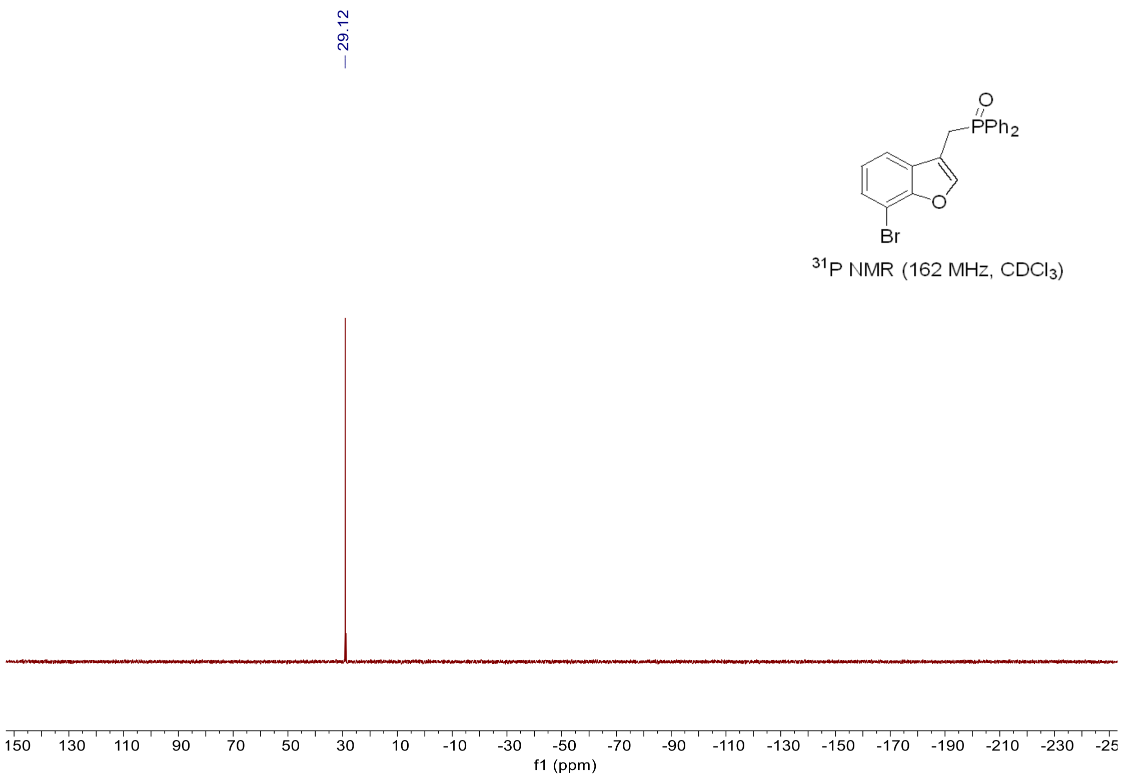
29.12
PPh2
O
Br
31P NMR (162 MHz, CDCl3)
150 130 110 90 70 50 30 10 -10 -30 -50 -70 -90 -110 -130 -150 -170 -190 -210 -230 -25
f1 (ppm)

Supplementary Figure 45. ^{1}H, ^{13}C and ^{31}P NMR spectra of **3k.**

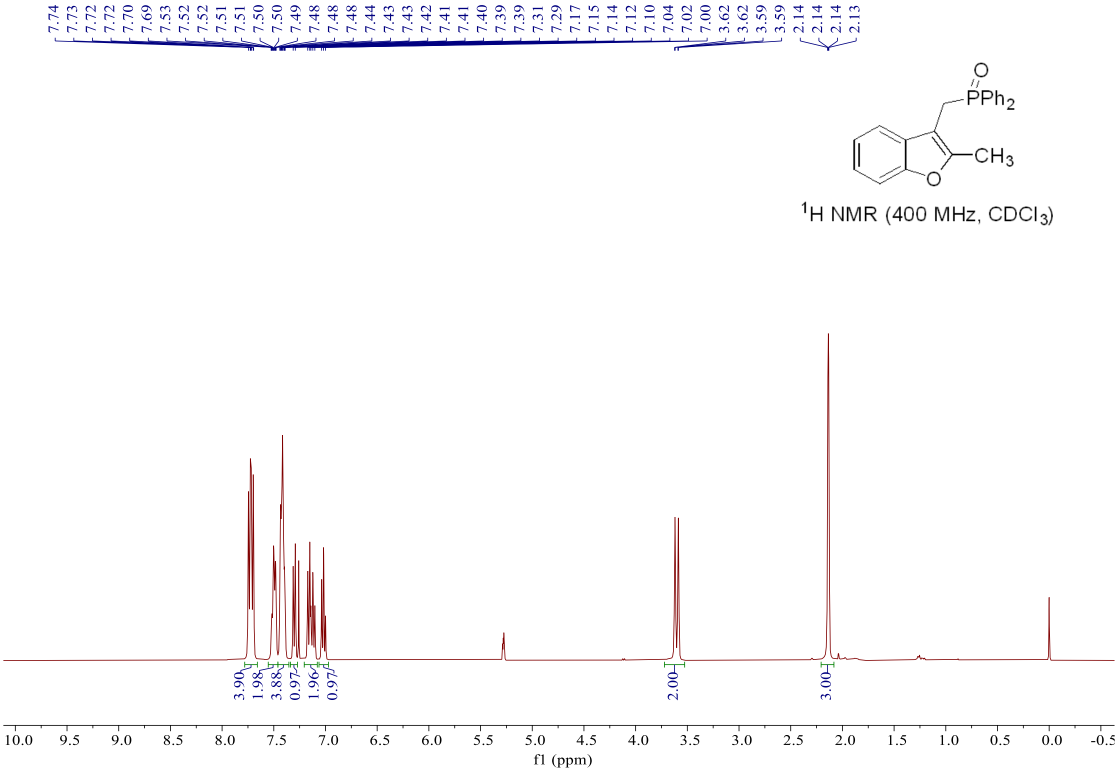

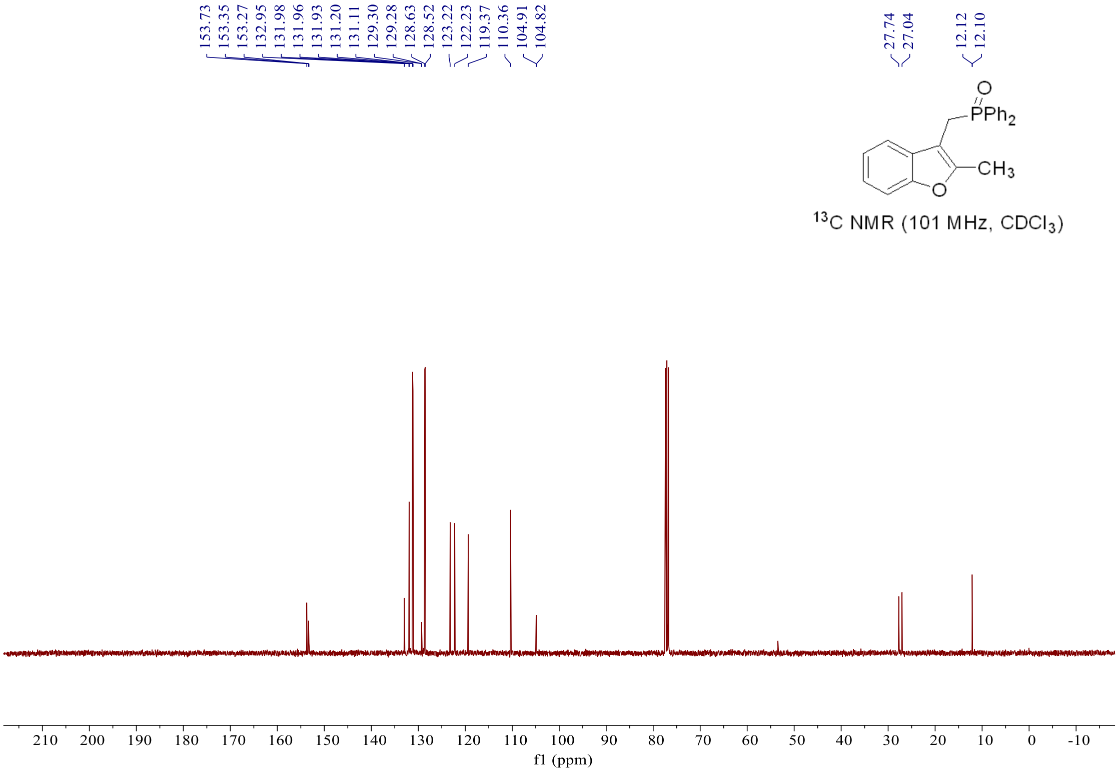

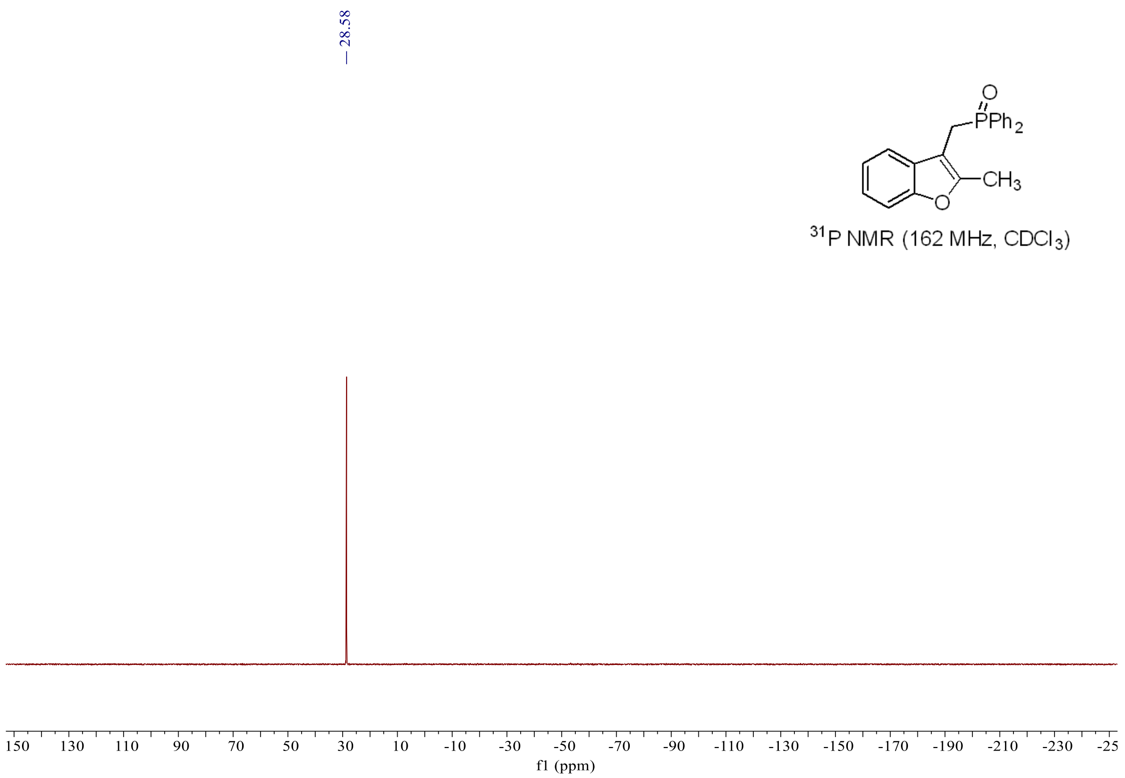
28.58
O
PPh2
CH3
O
31P NMR (162 MHz, CDCl3)
150 130 110 90 70 50 30 10 -10 -30 -50 -70 -90 -110 -130 -150 -170 -190 -210 -230 -25
f1 (ppm)

Supplementary Figure 46. ^{1}H, ^{13}C and ^{31}P NMR spectra of **3l**.

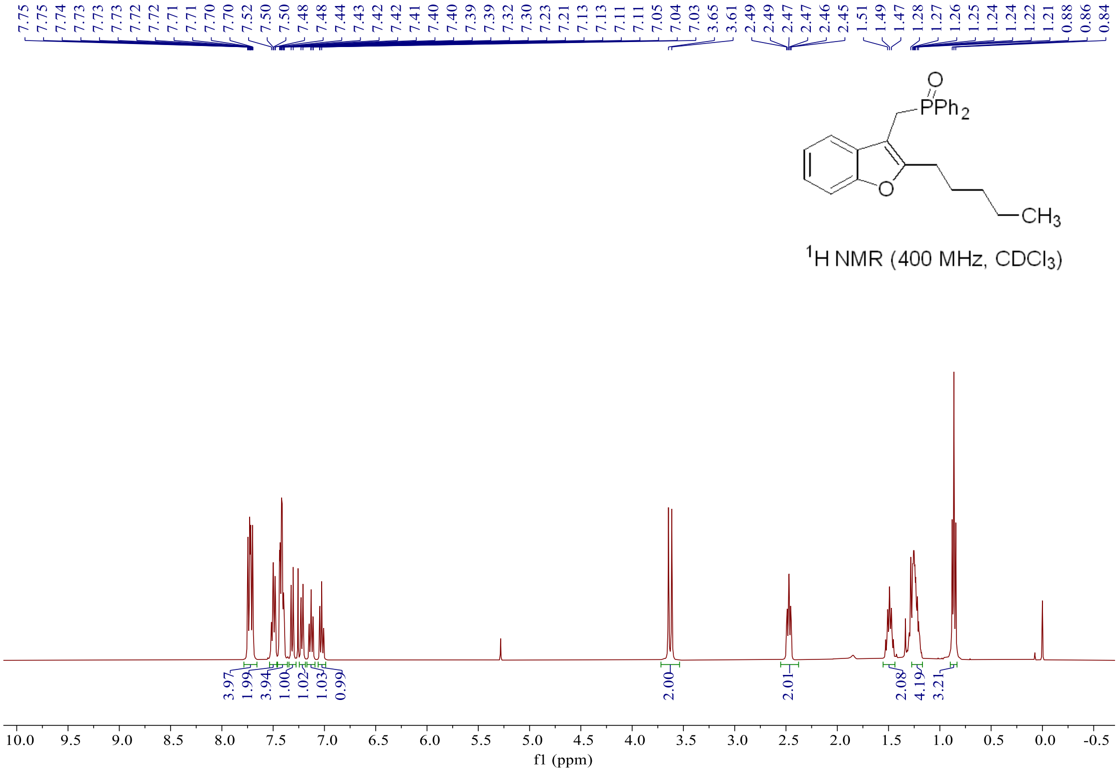

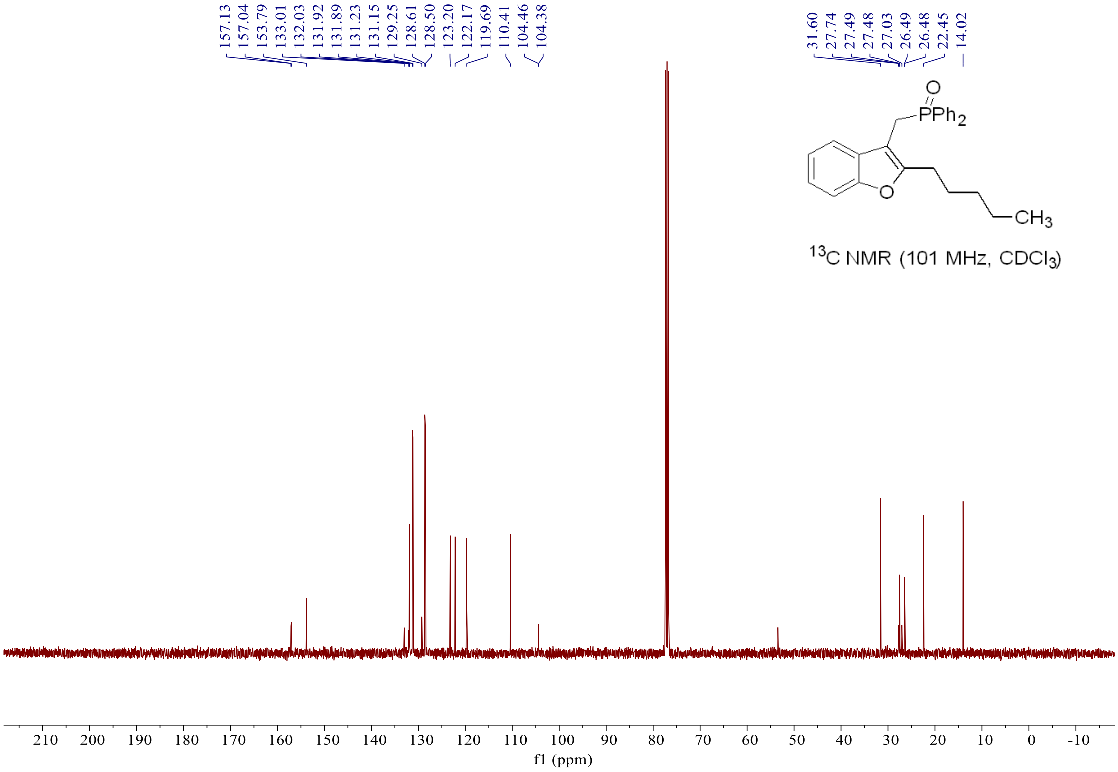

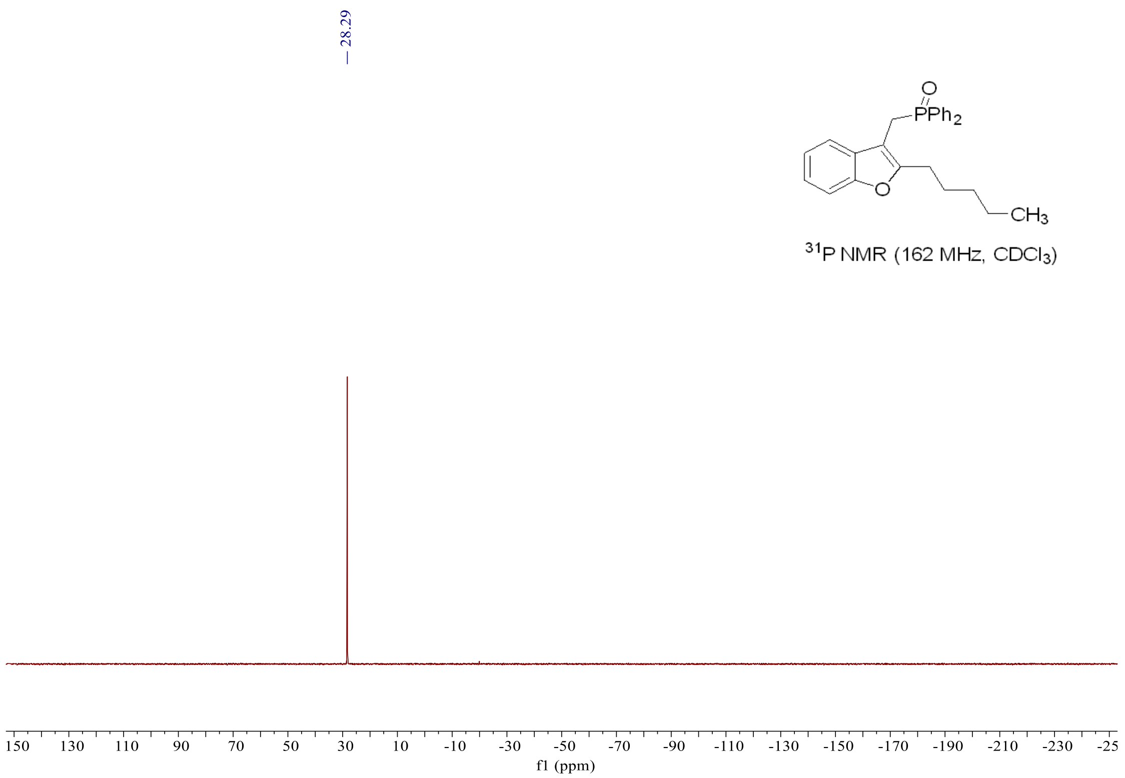
28.29
O
PPh2
O
CH3
31P NMR (162 MHz, CDCl3)
150 130 110 90 70 50 30 10 -10 -30 -50 -70 -90 -110 -130 -150 -170 -190 -210 -230 -25
f1 (ppm)

Supplementary Figure 47. ^{1}H, ^{13}C and ^{31}P NMR spectra of **3m**.

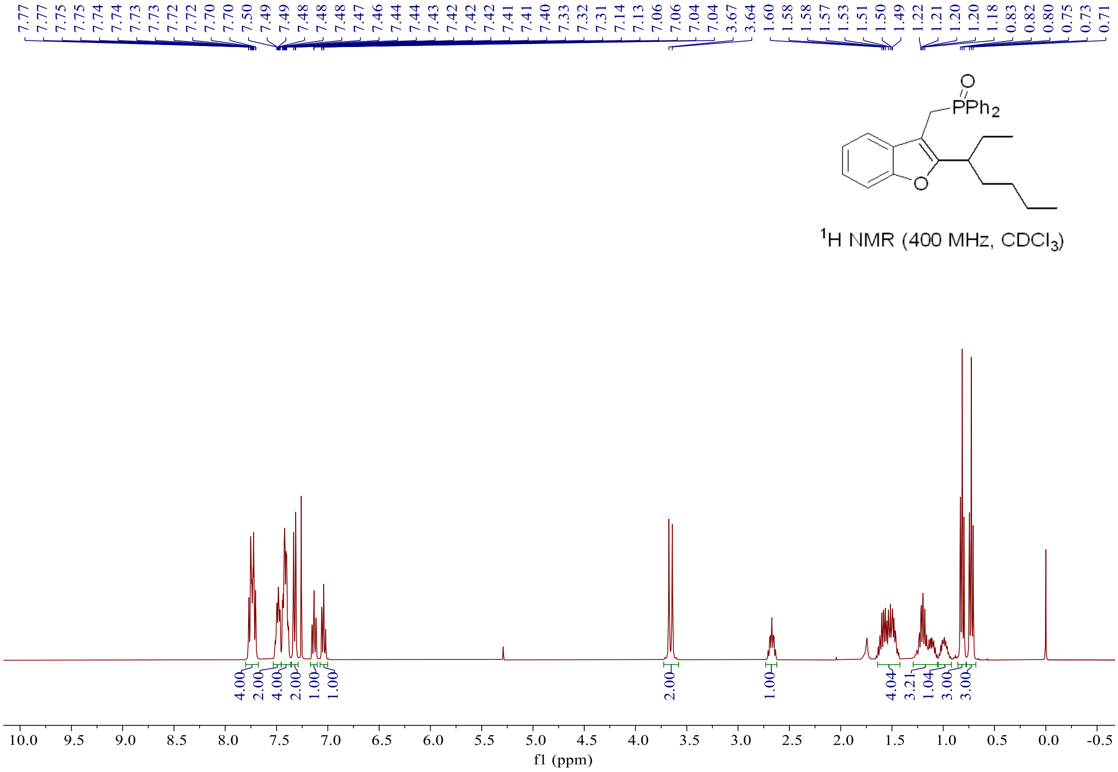

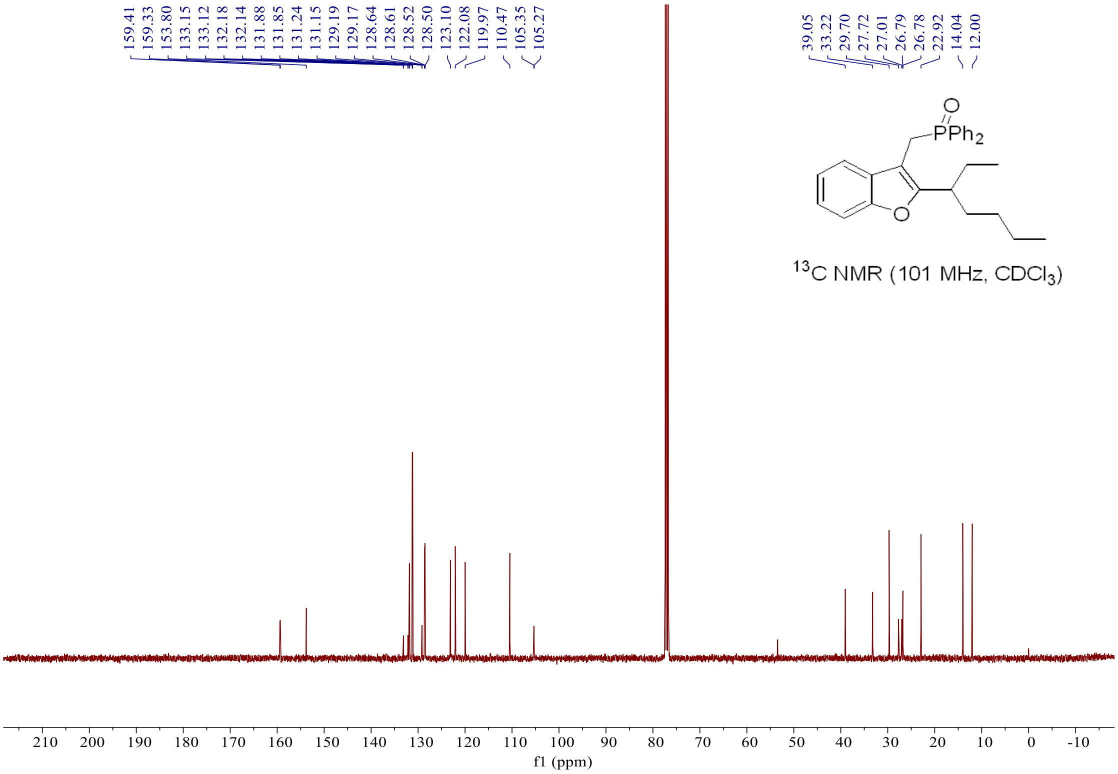

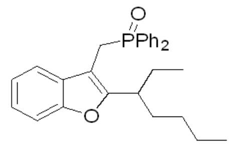

^{31}P NMR (162 MHz, $CDCl_3$)

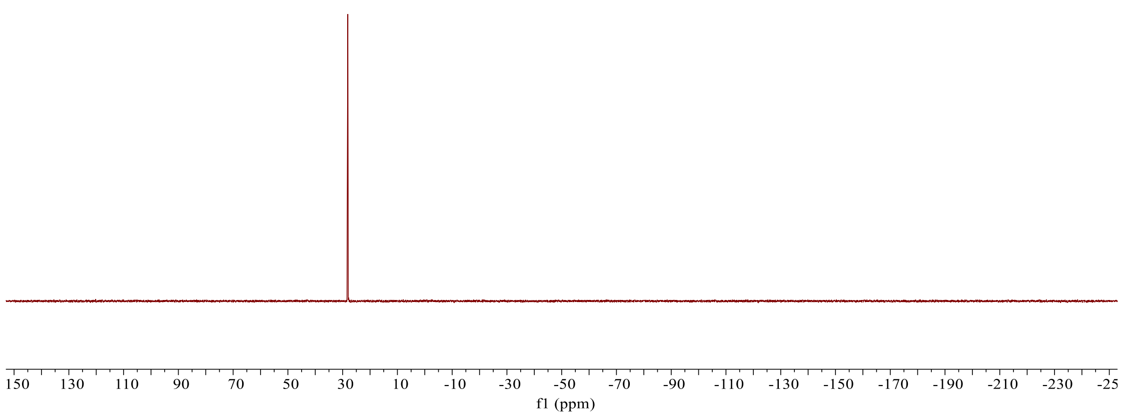

Supplementary Figure 48. ^{1}H and ^{13}C NMR spectra of **5a**.

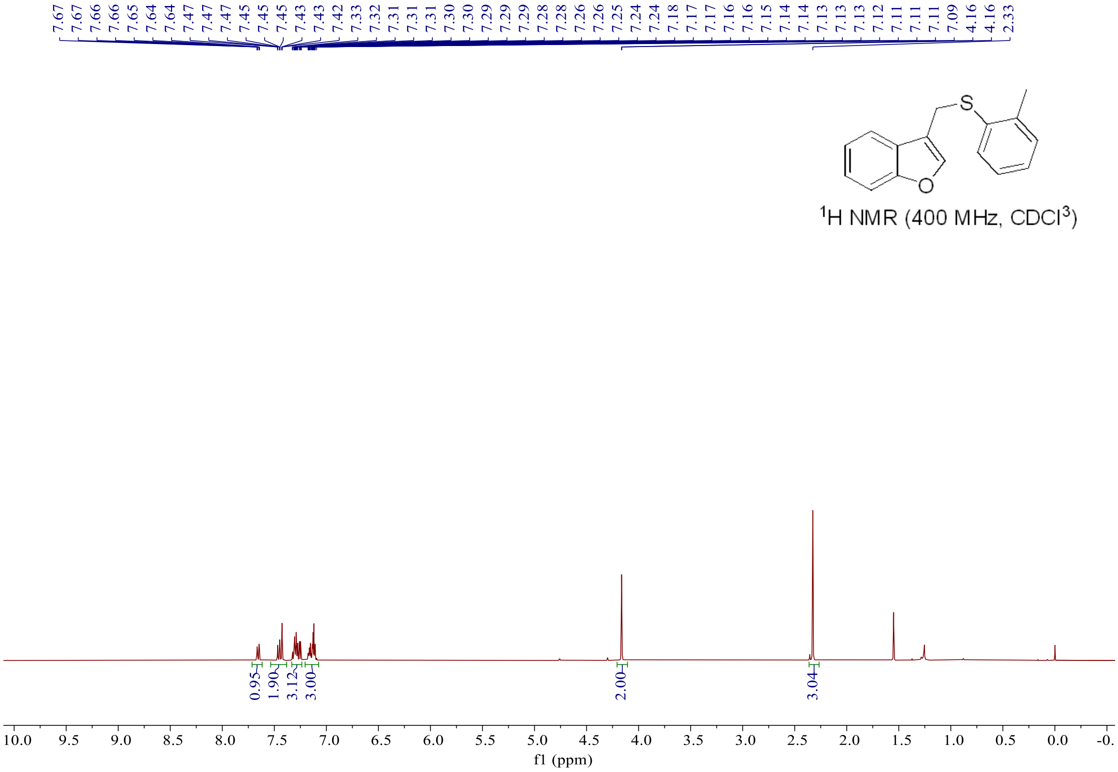

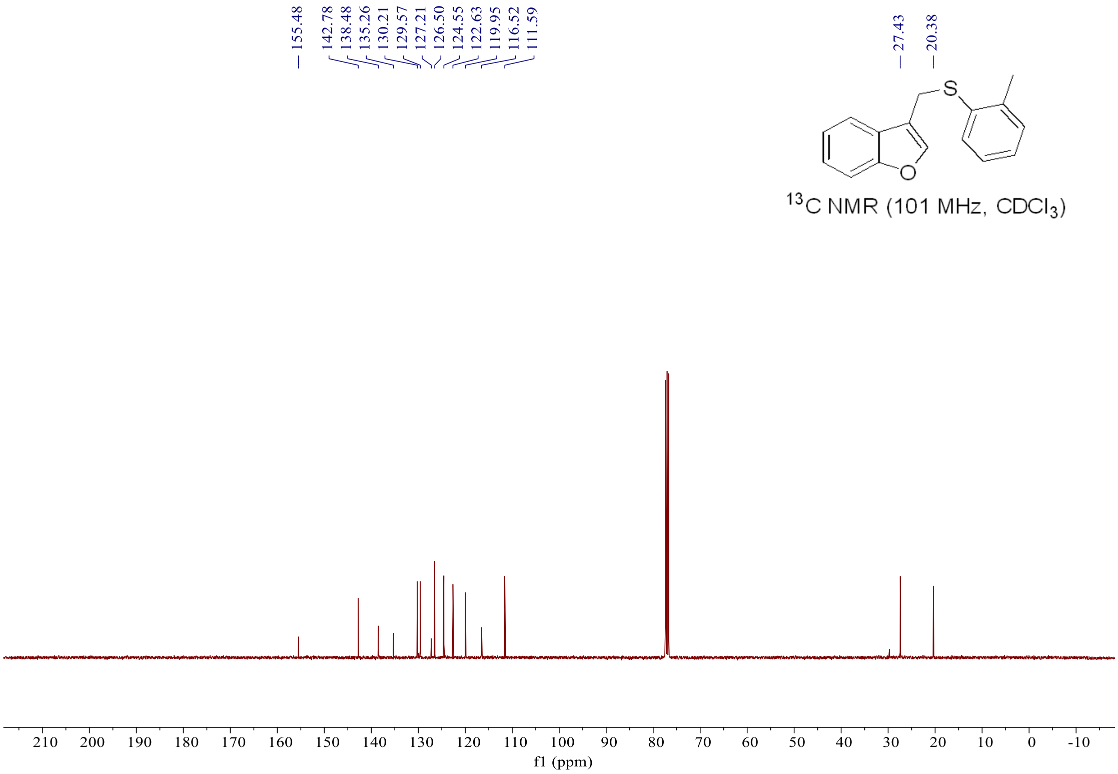

Supplementary Figure 49. ^{1}H and ^{13}C NMR spectra of **5b**.

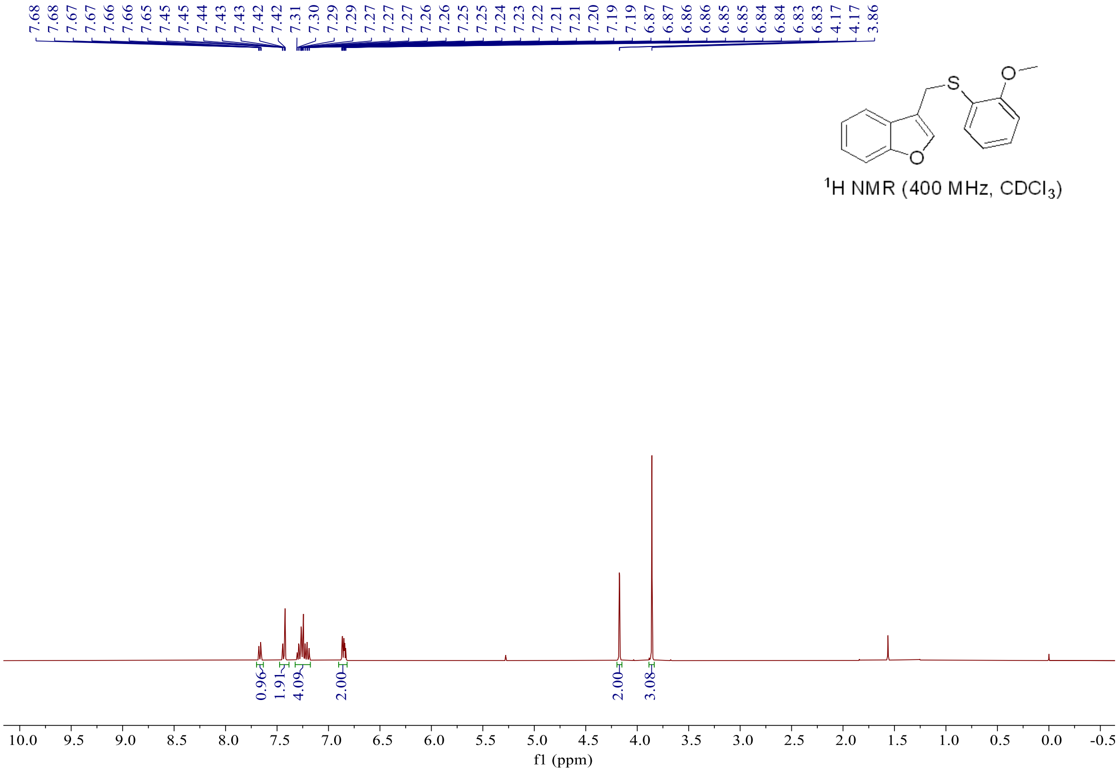

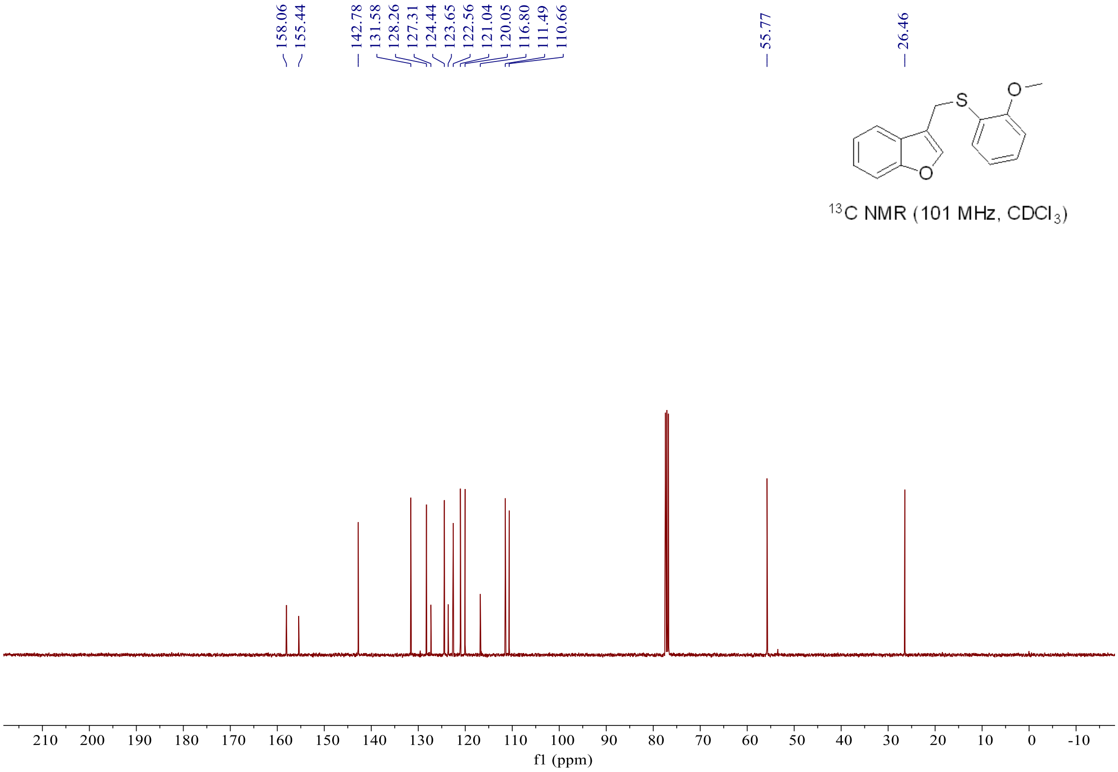

Supplementary Figure 50. ^{1}H, ^{13}C and ^{19}F NMR spectra of **5c**.

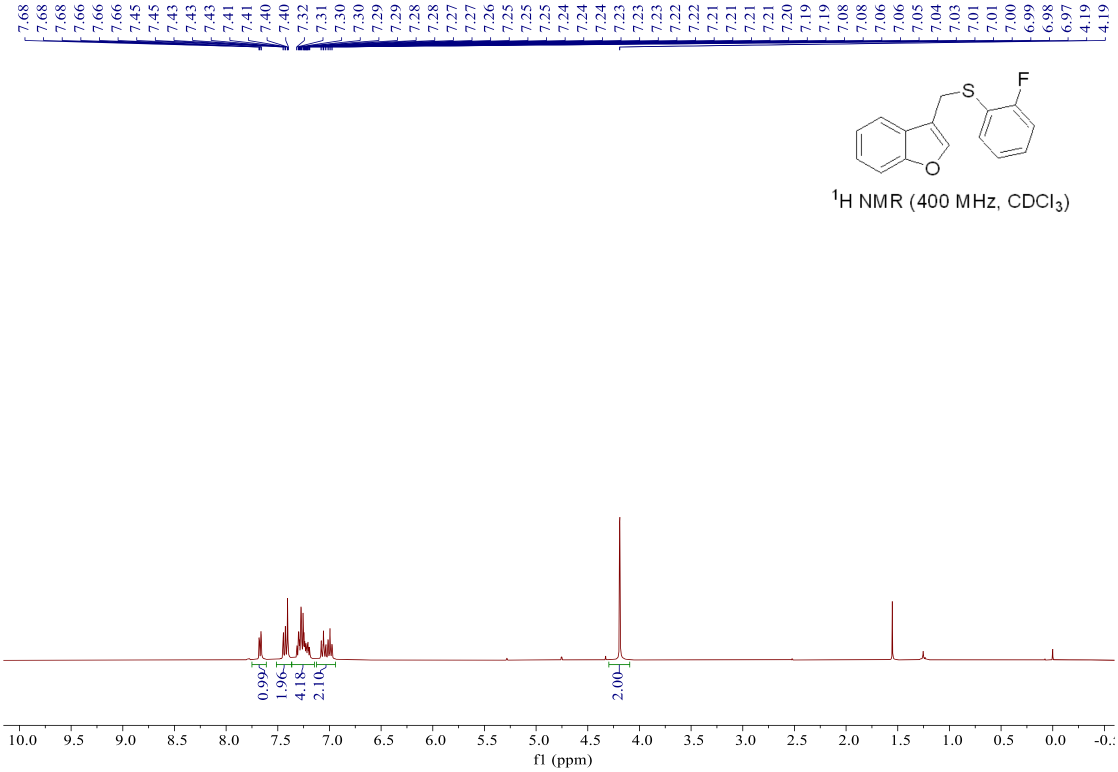

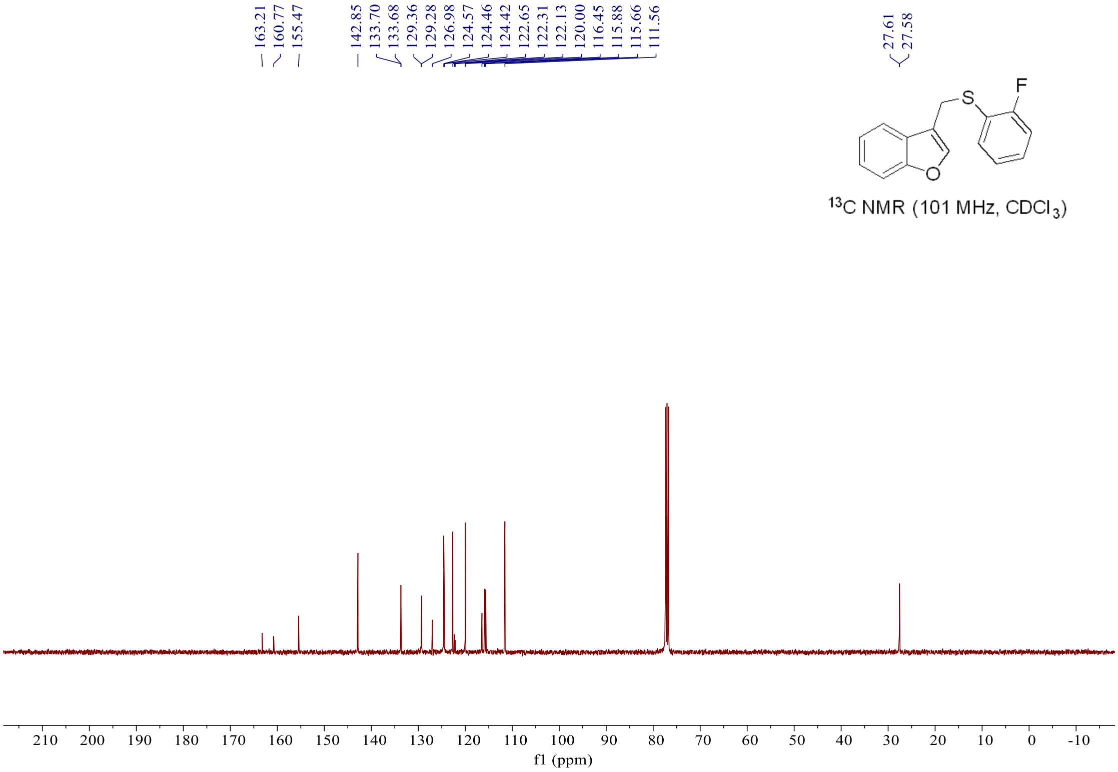

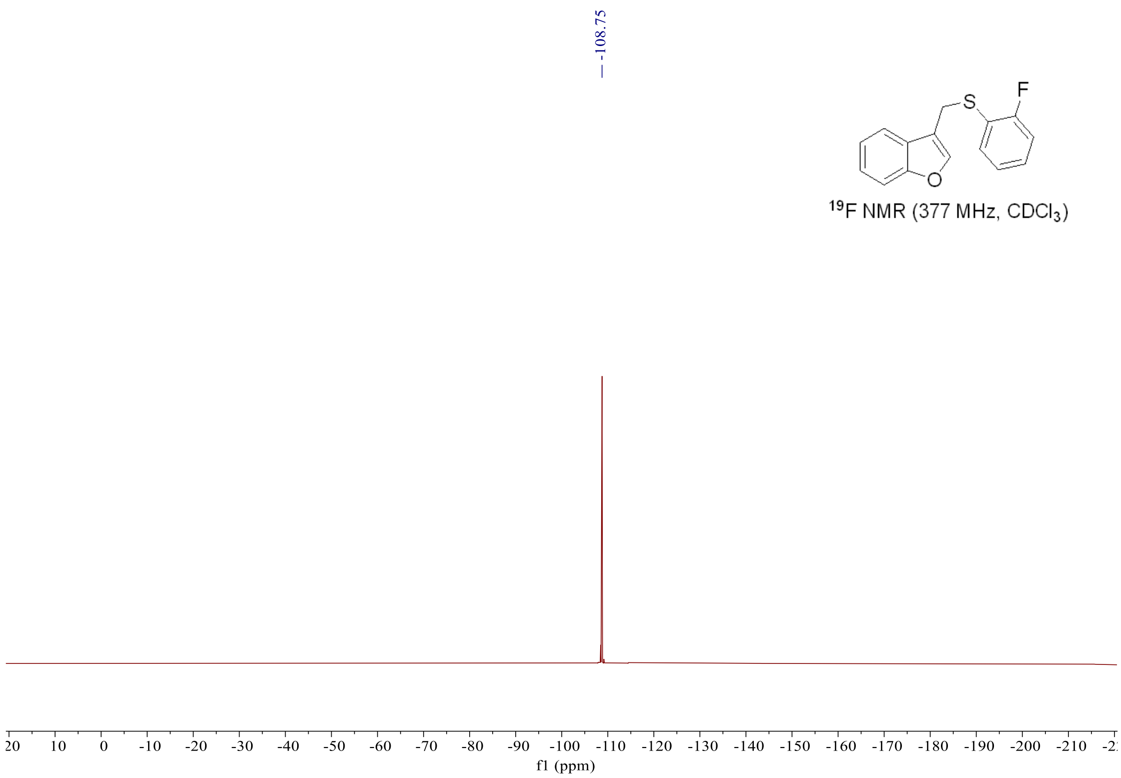
-108.75
F
S
O
^{19}F NMR (377 MHz, CDCl$_3$)
20 10 0 -10 -20 -30 -40 -50 -60 -70 -80 -90 -100 -110 -120 -130 -140 -150 -160 -170 -180 -190 -200 -210
f1 (ppm)

Supplementary Figure 51. ^{1}H and ^{13}C NMR spectra of **5d**.

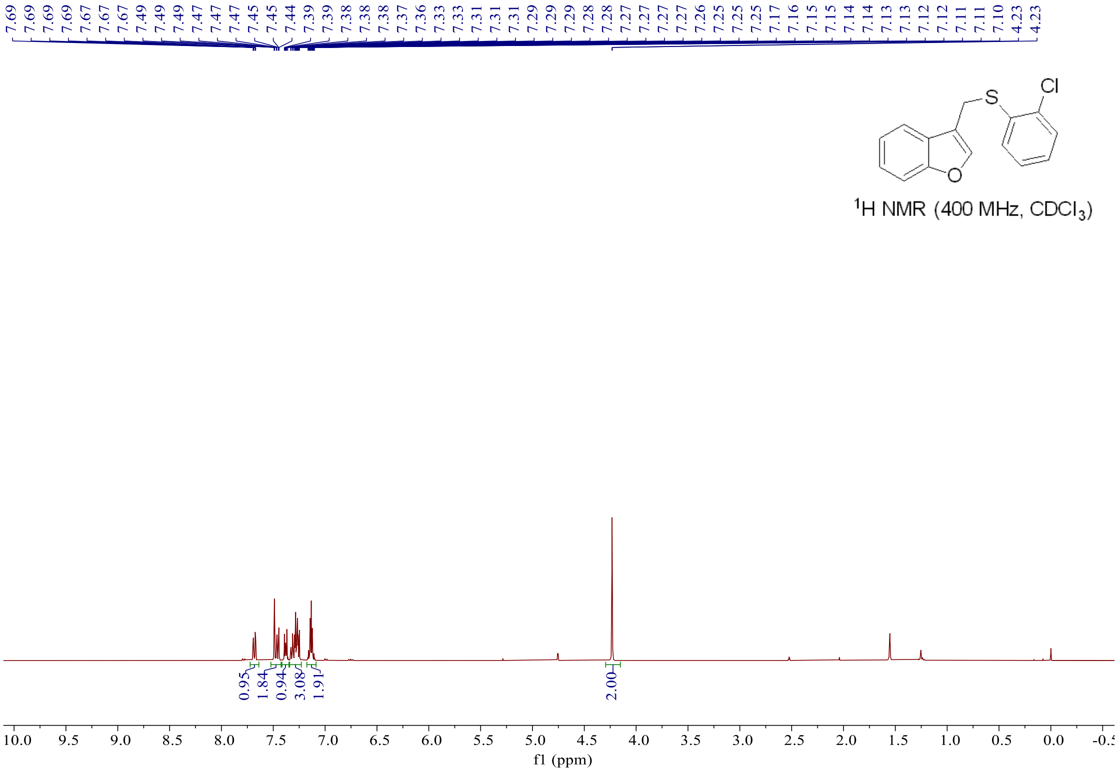

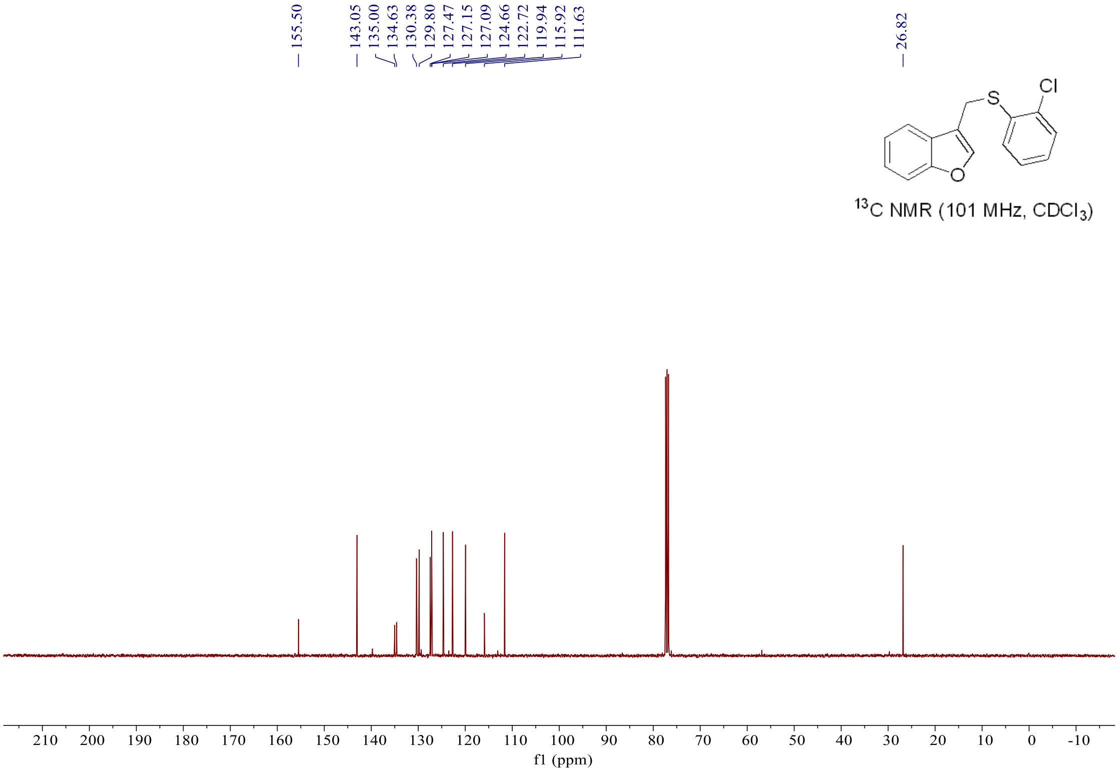

Supplementary Figure 52. ^{1}H and ^{13}C NMR spectra of **5e**.

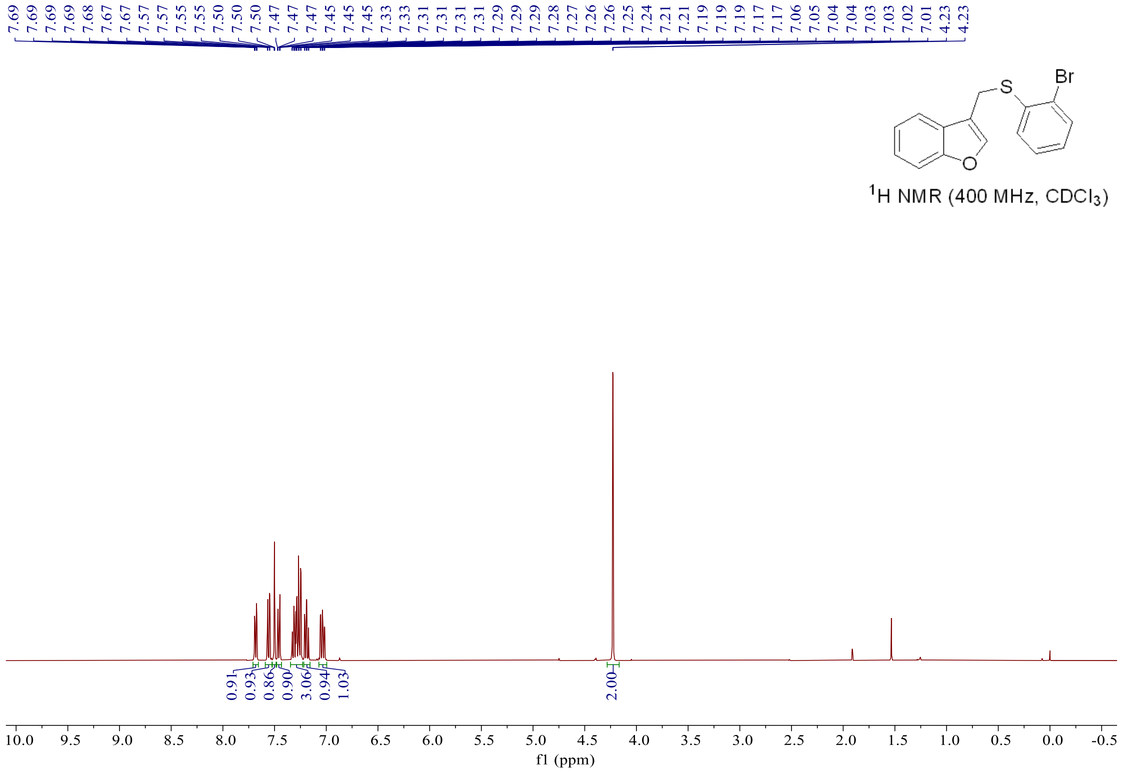

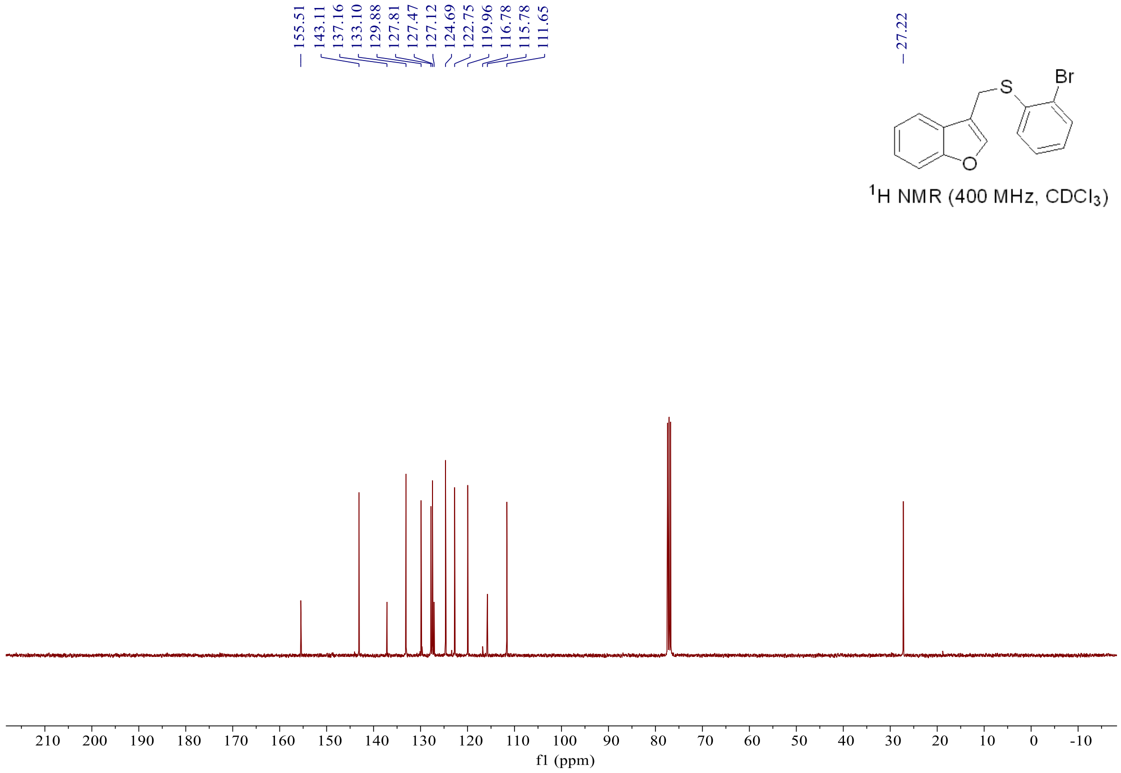

Supplementary Figure 53. ^{1}H and ^{13}C NMR spectra of **5f.**

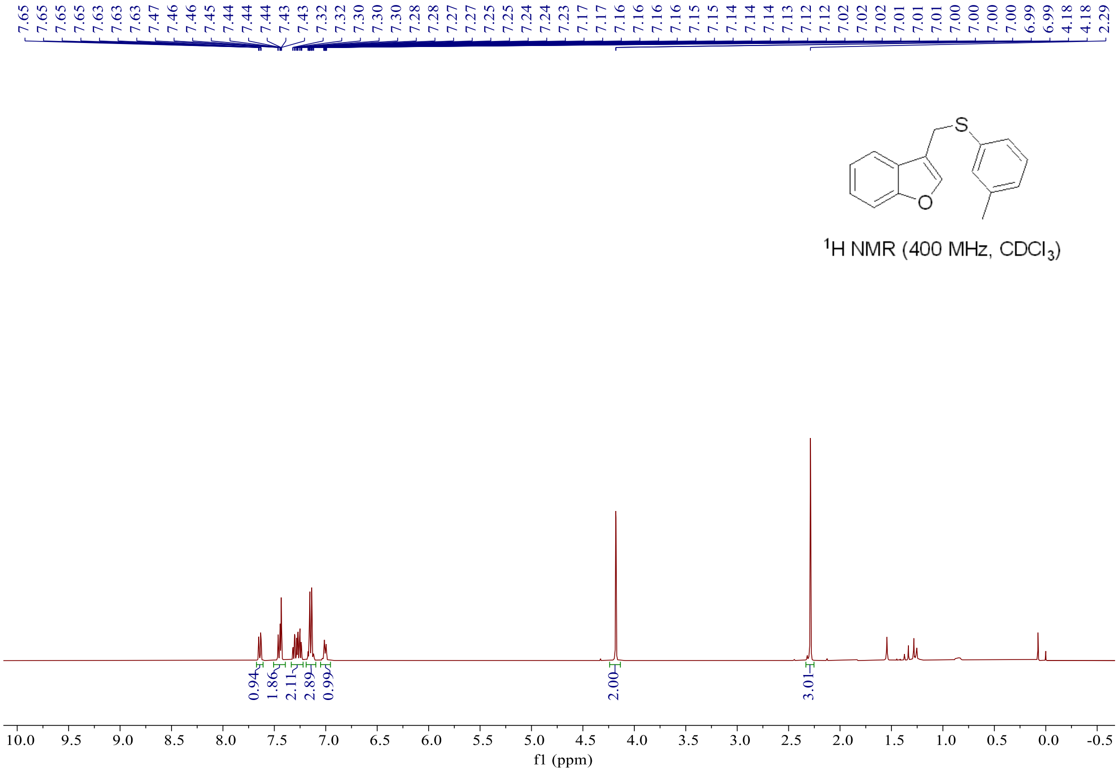

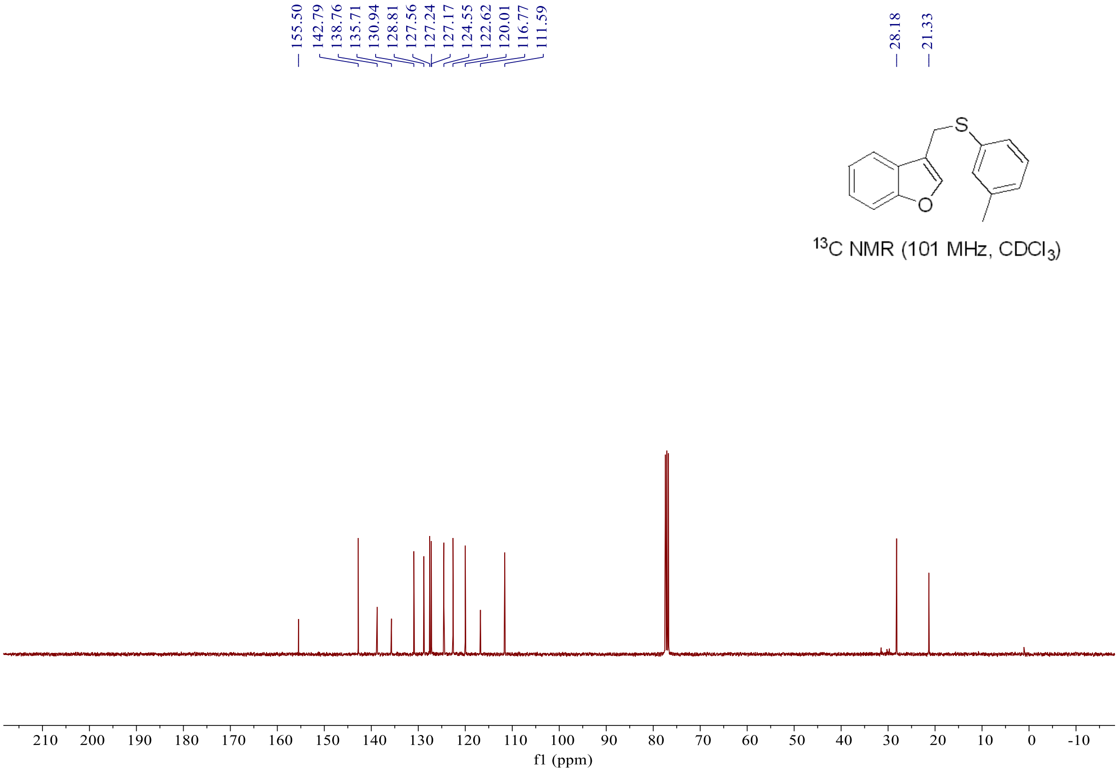

Supplementary Figure 54. ^{1}H and ^{13}C NMR spectra of **5g**.

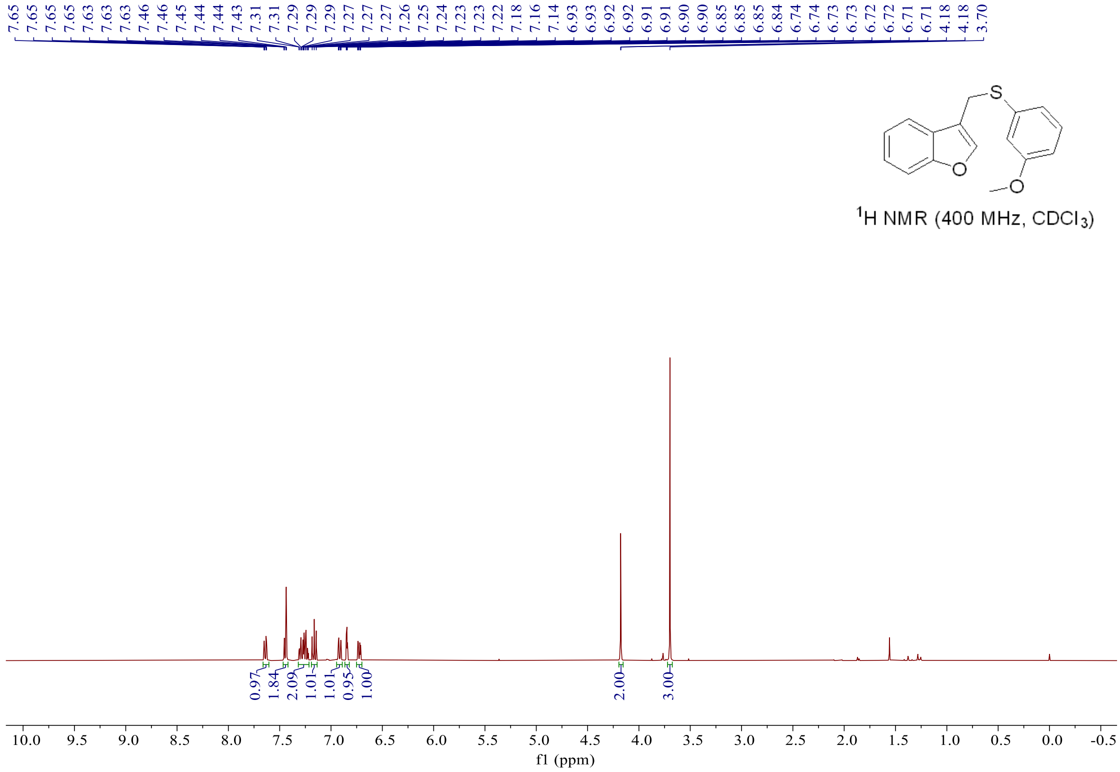

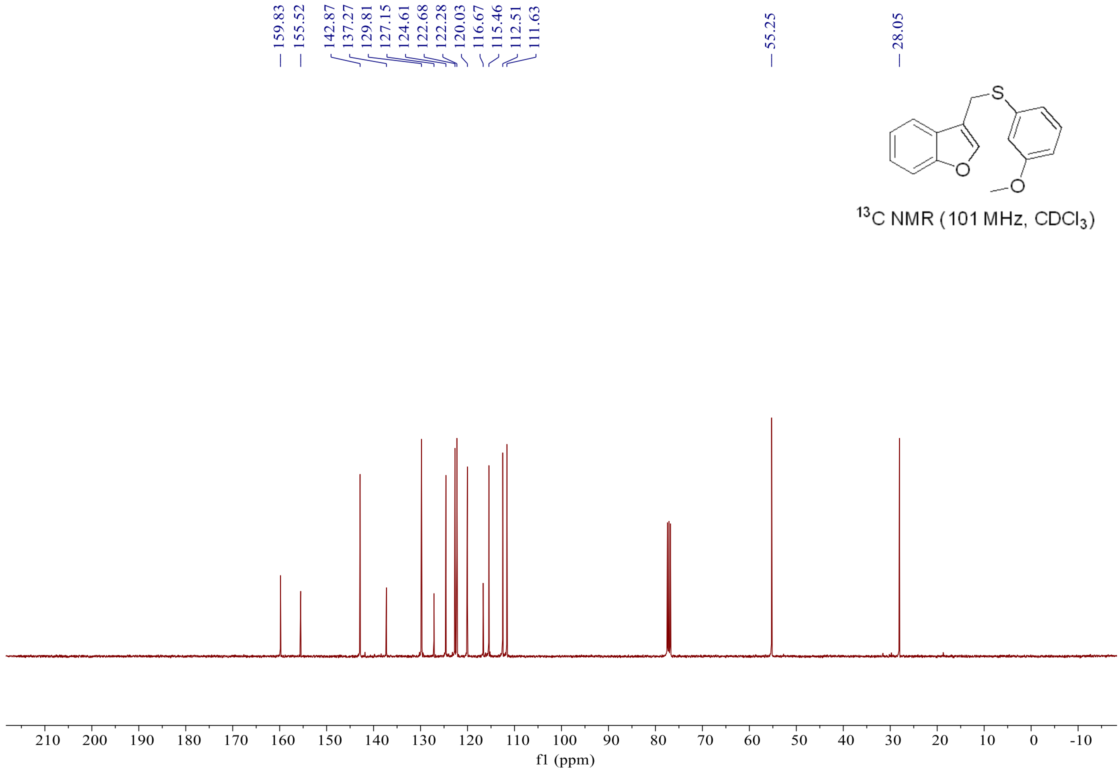

Supplementary Figure 55. ^{1}H and ^{13}C NMR spectra of **5h**.

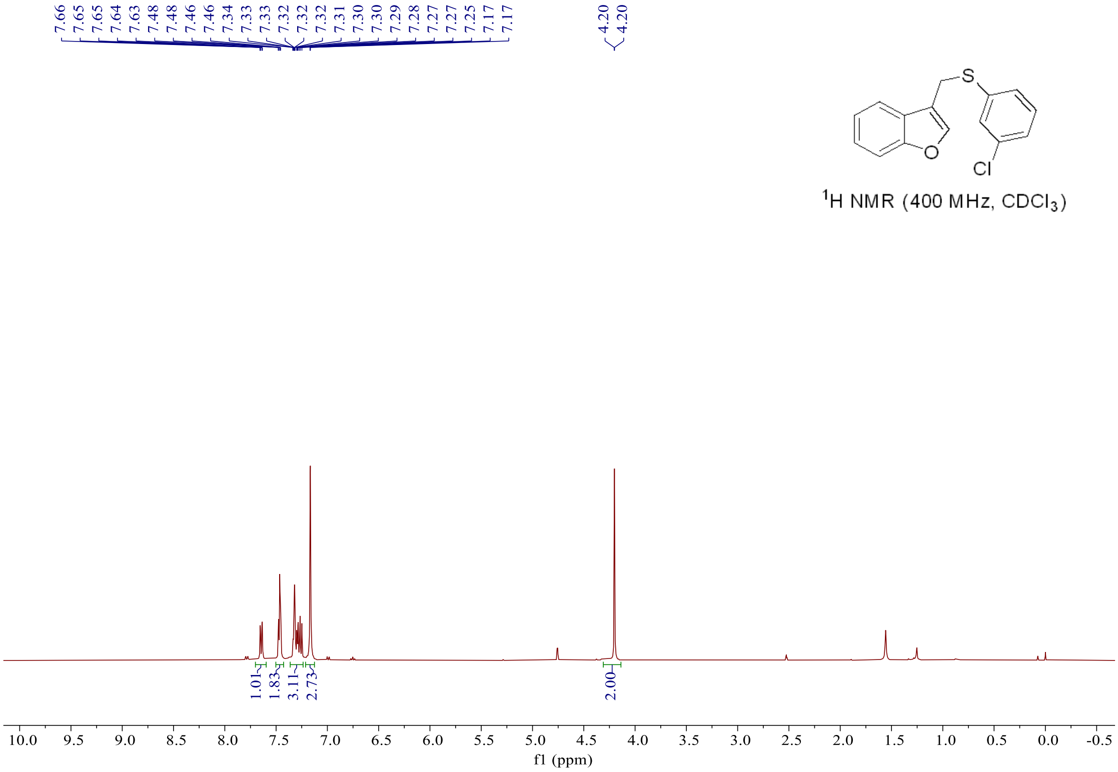

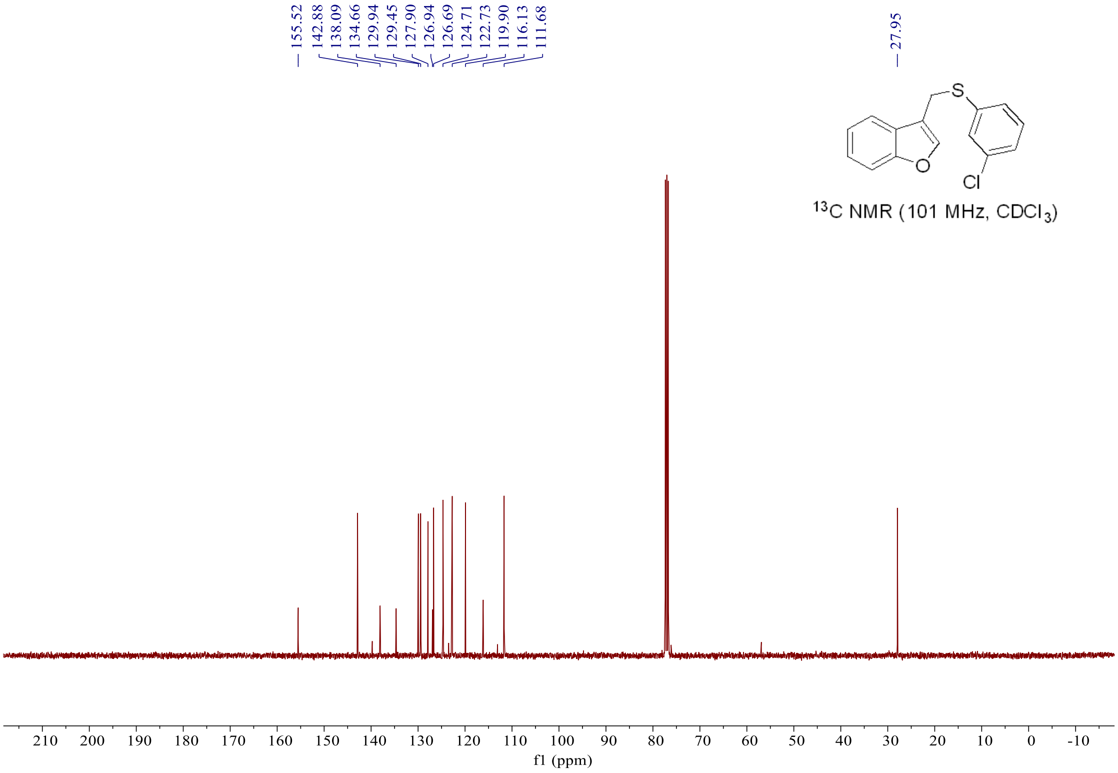

Supplementary Figure 56. ^{1}H and ^{13}C NMR spectra of **5i.**

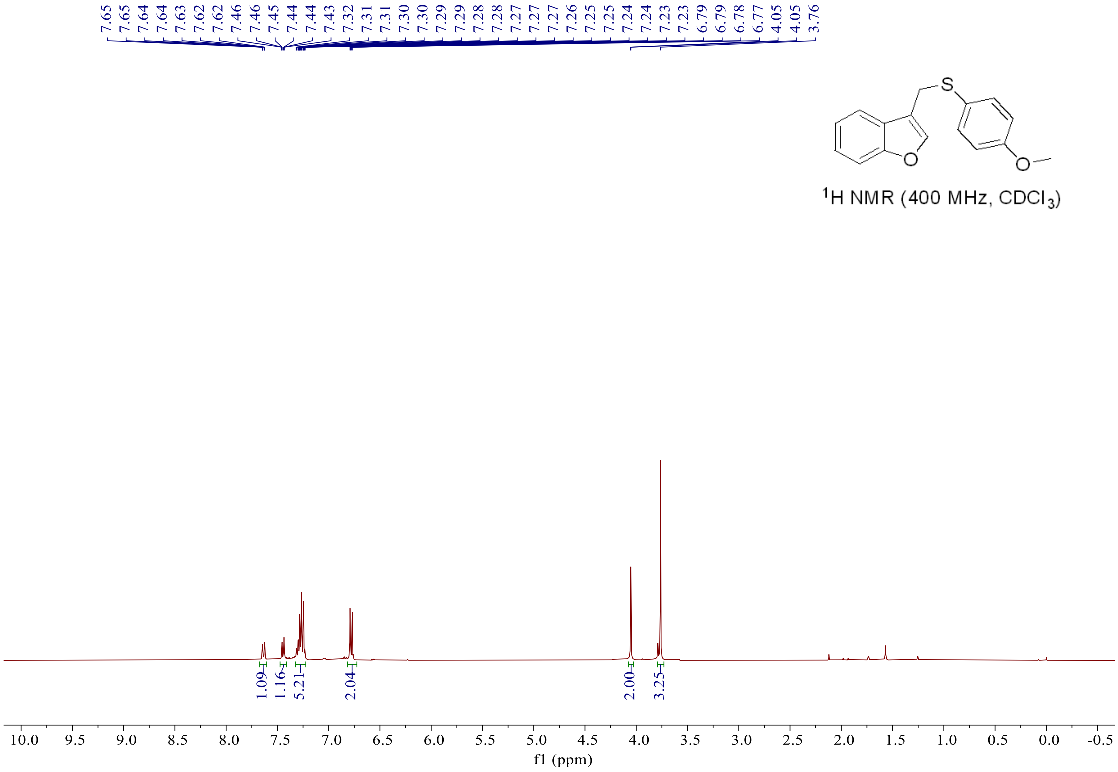

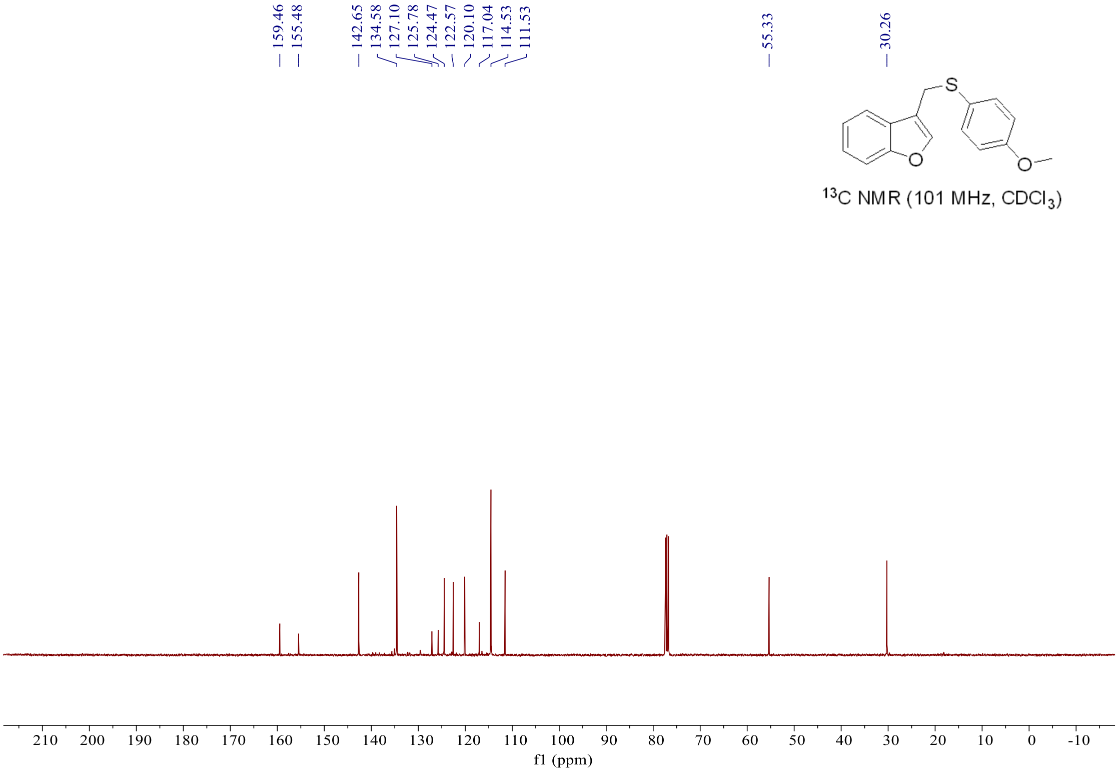

Supplementary Figure 57. ^{1}H and ^{13}C NMR spectra of **5j**.

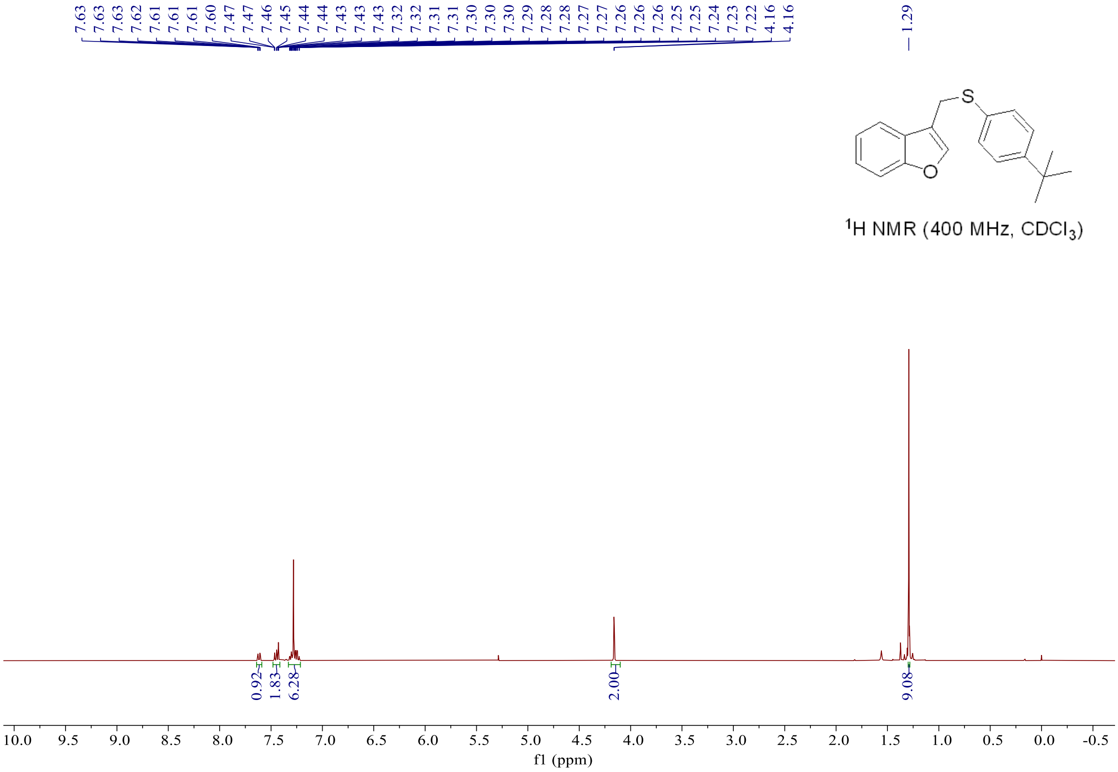

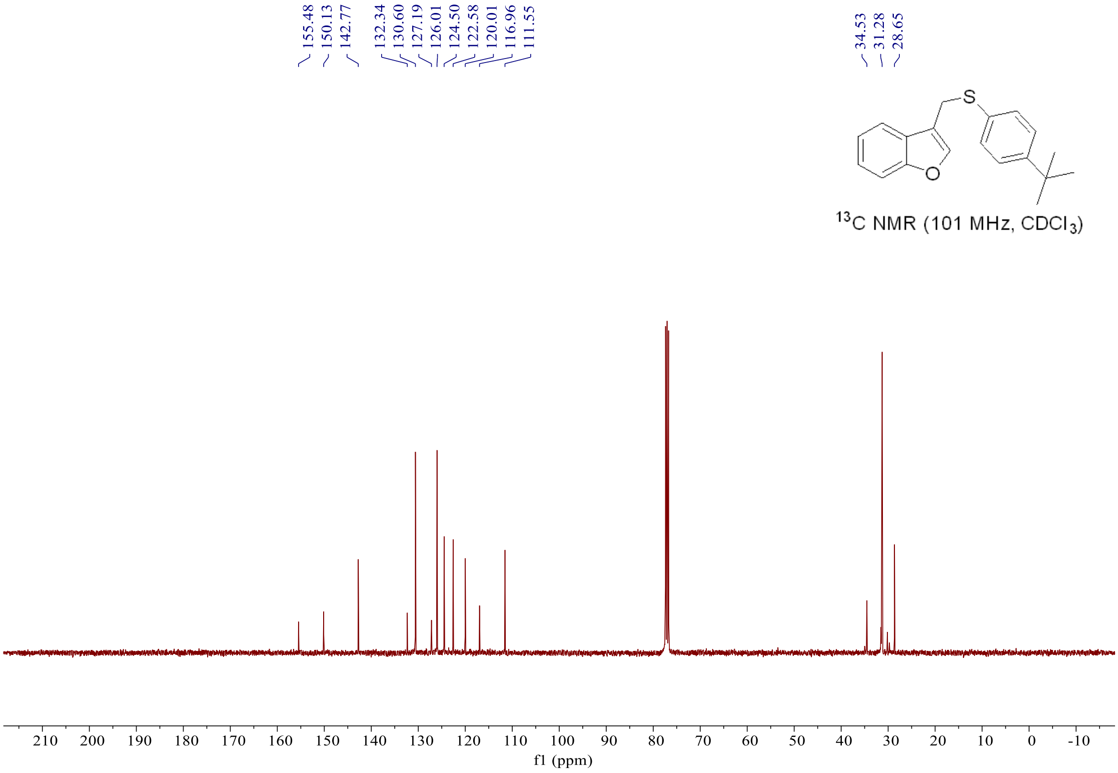

Supplementary Figure 58. ^{1}H and ^{13}C NMR spectra of **5k**.

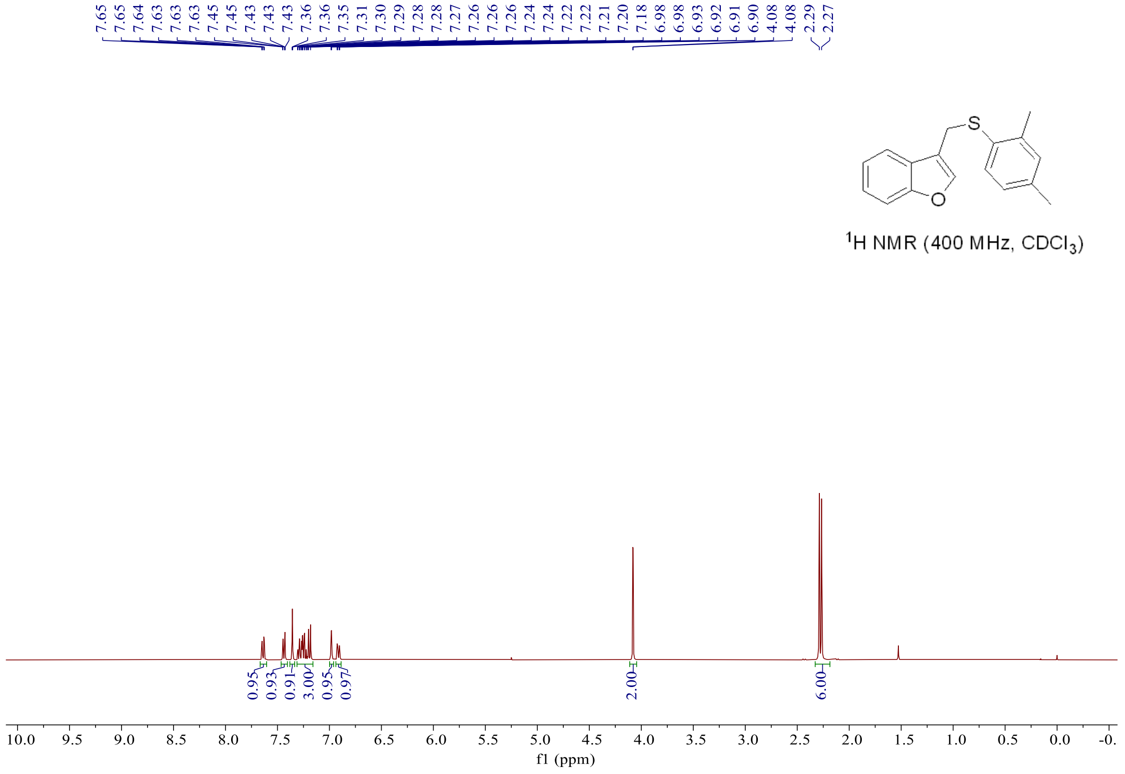

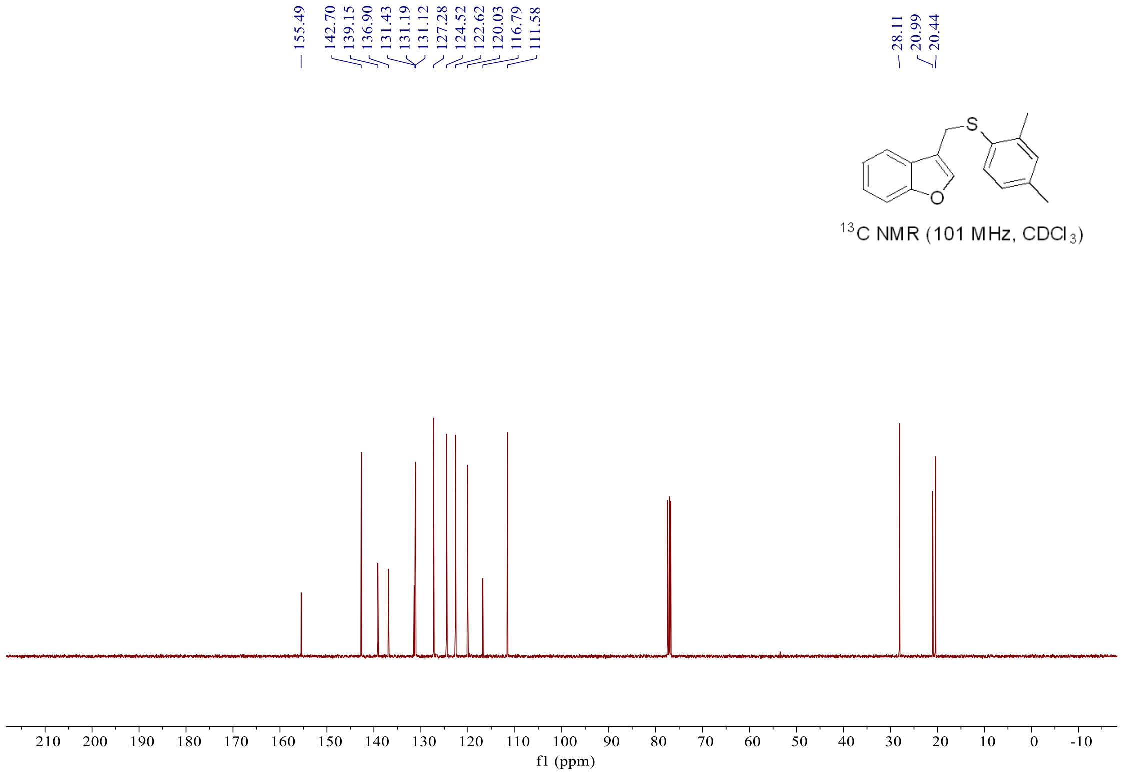

Supplementary Figure 59. ^{1}H and ^{13}C NMR spectra of **5l**.

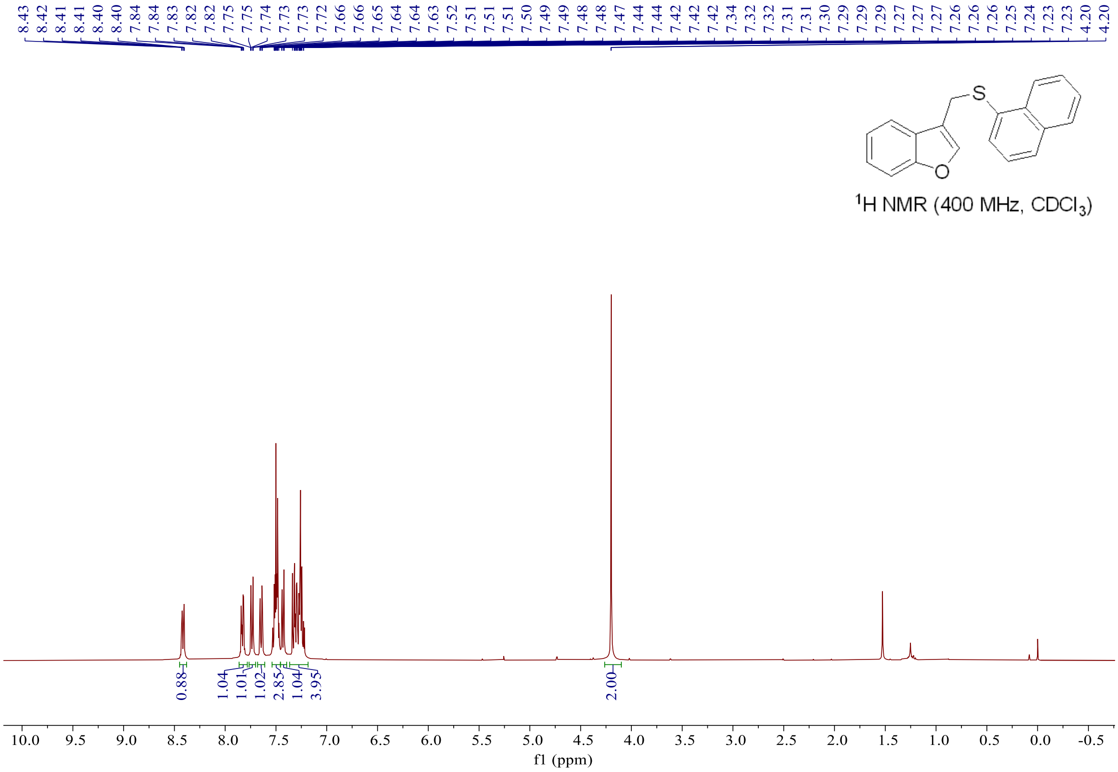

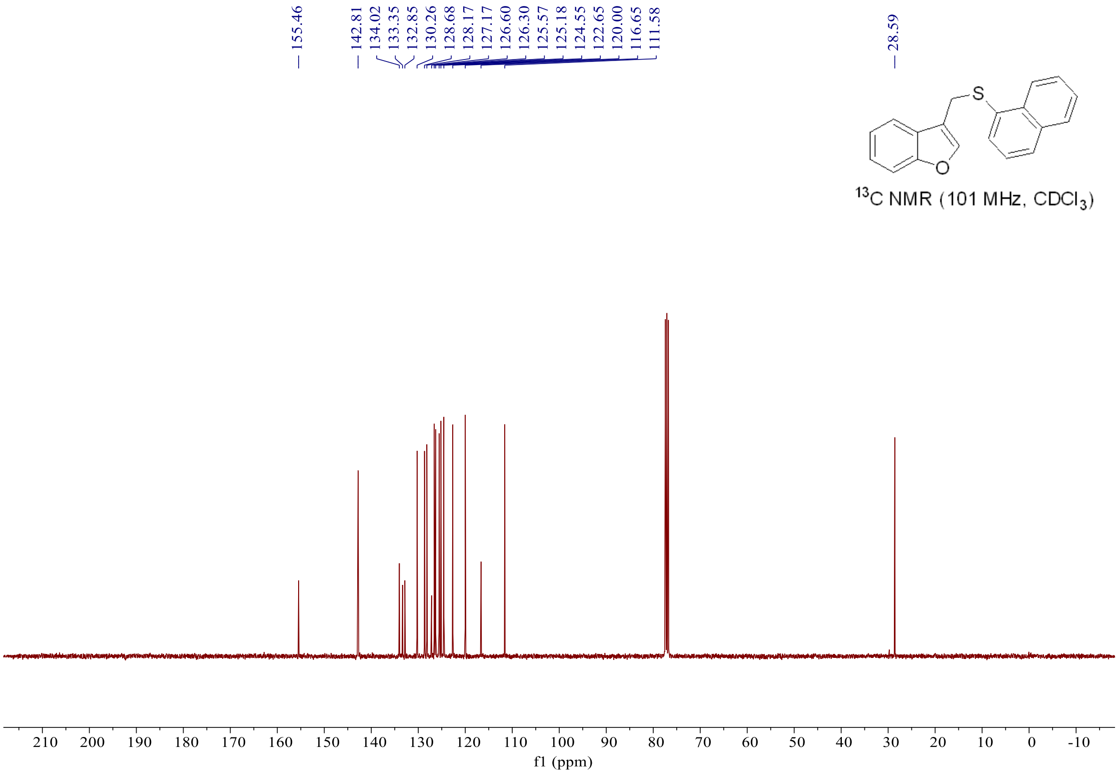

Supplementary Figure 60. ^{1}H and ^{13}C NMR spectra of **5m**.

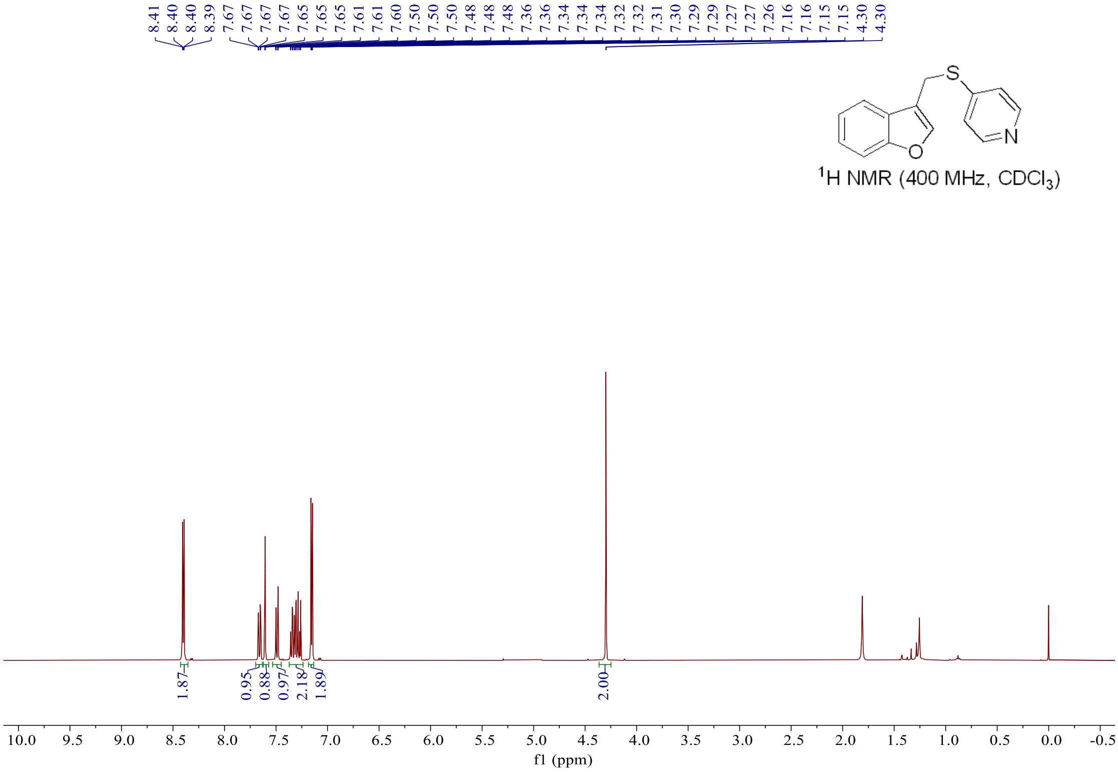

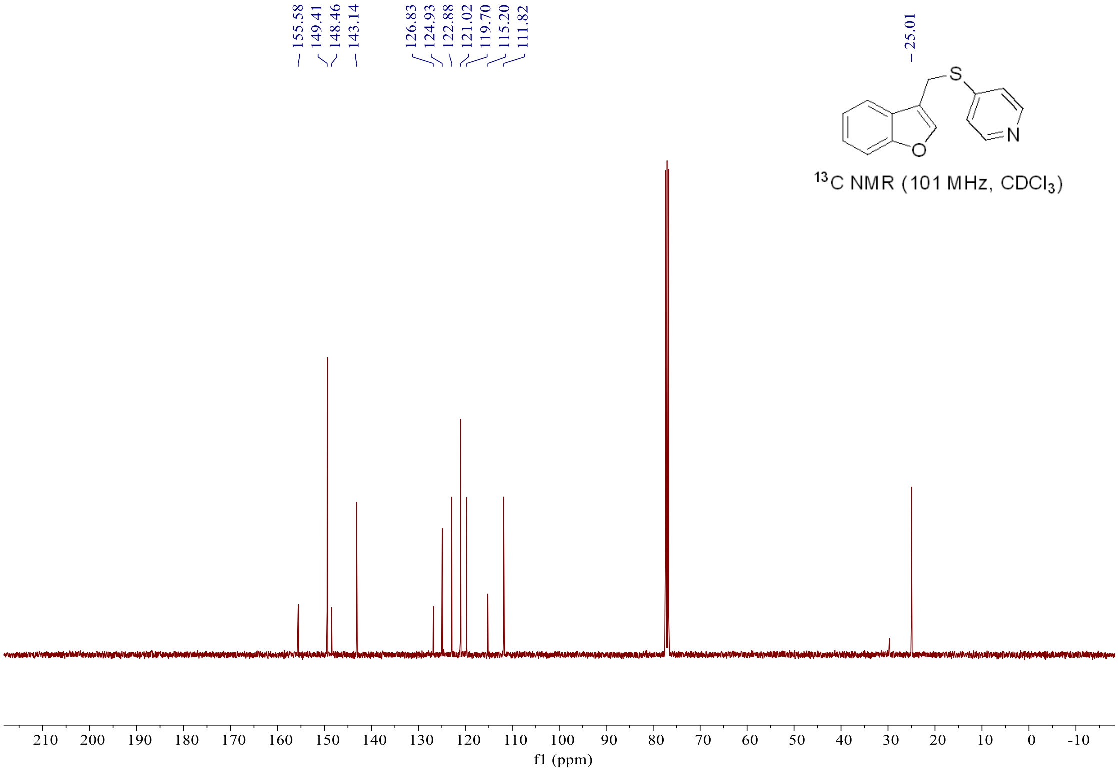

Supplementary Figure 61. ^{1}H and ^{13}C NMR spectra of **5n**.

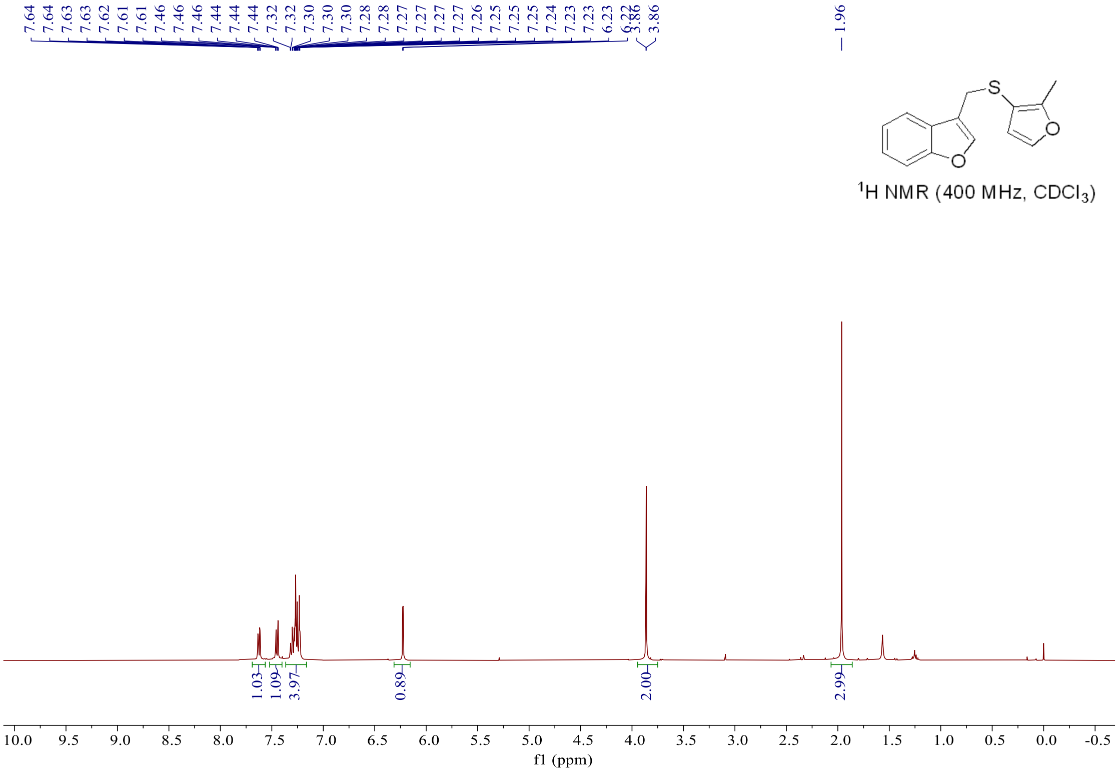

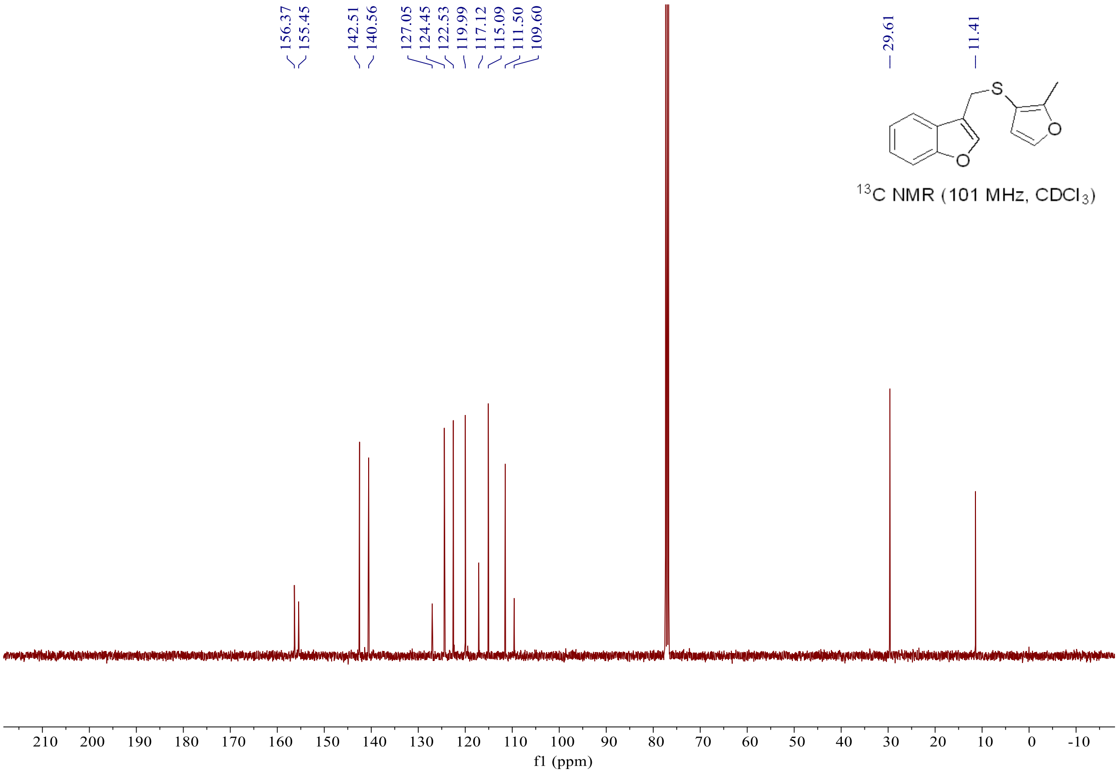

Supplementary Figure 62. ^{1}H and ^{13}C NMR spectra of **5o**.

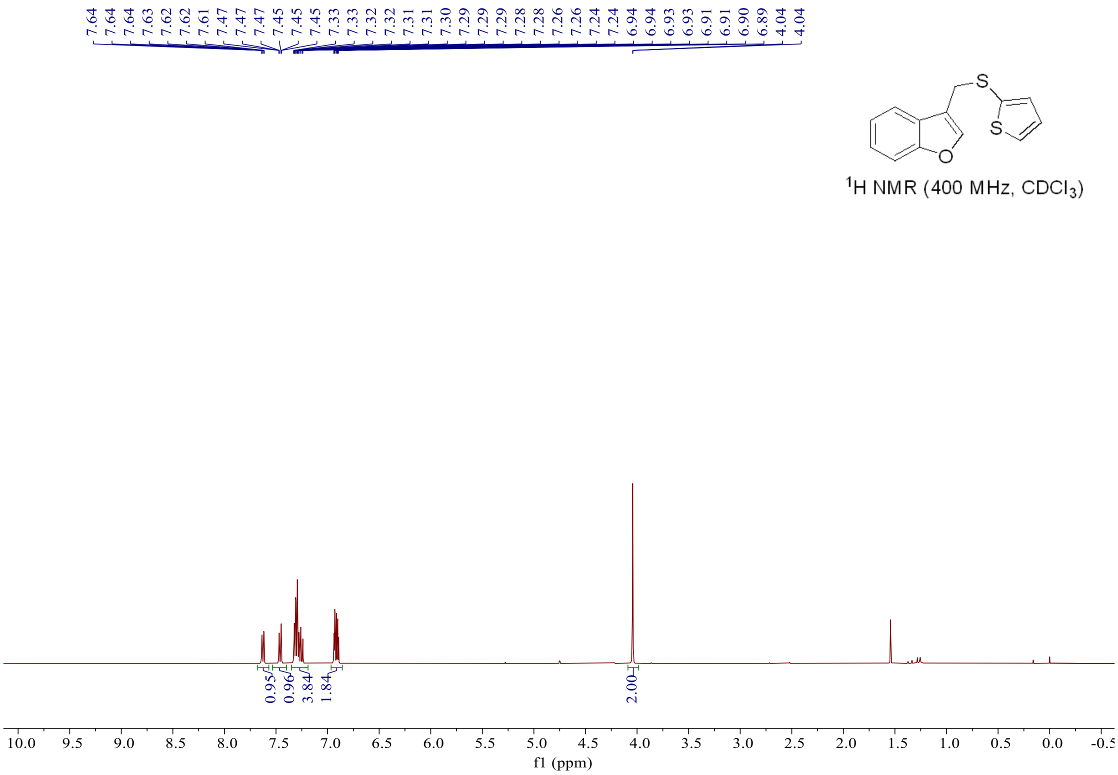

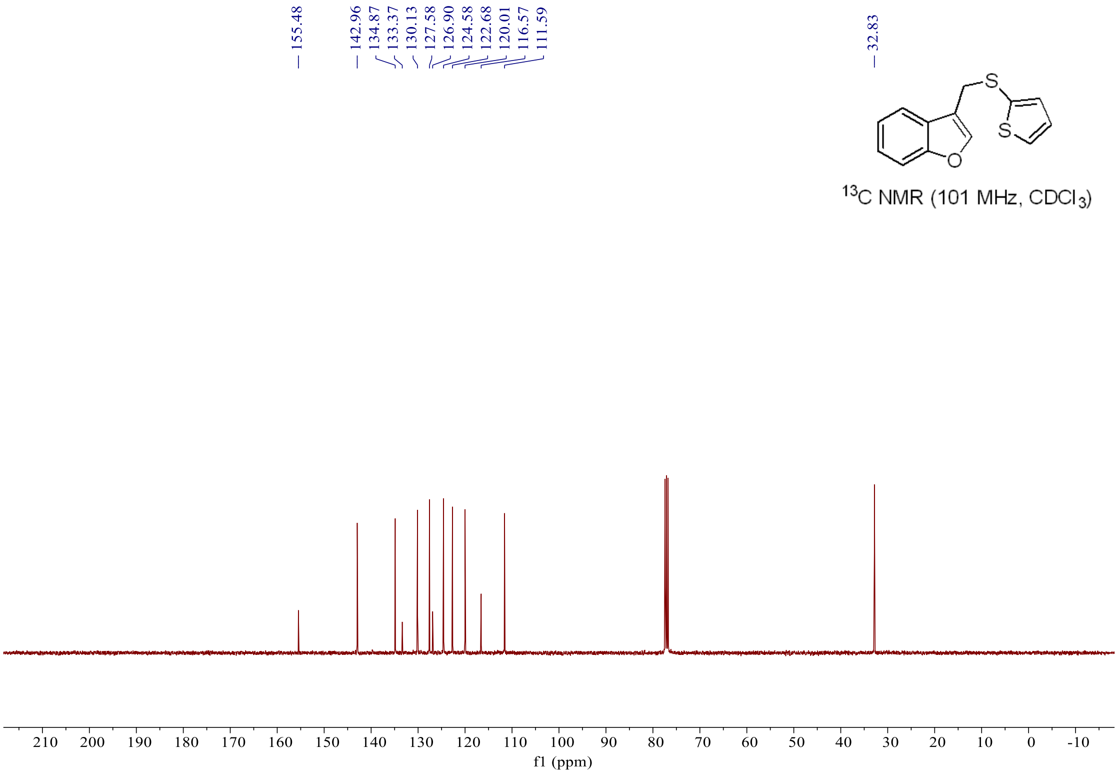

Supplementary Figure 63. ^{1}H and ^{13}C NMR spectra of **5p**.

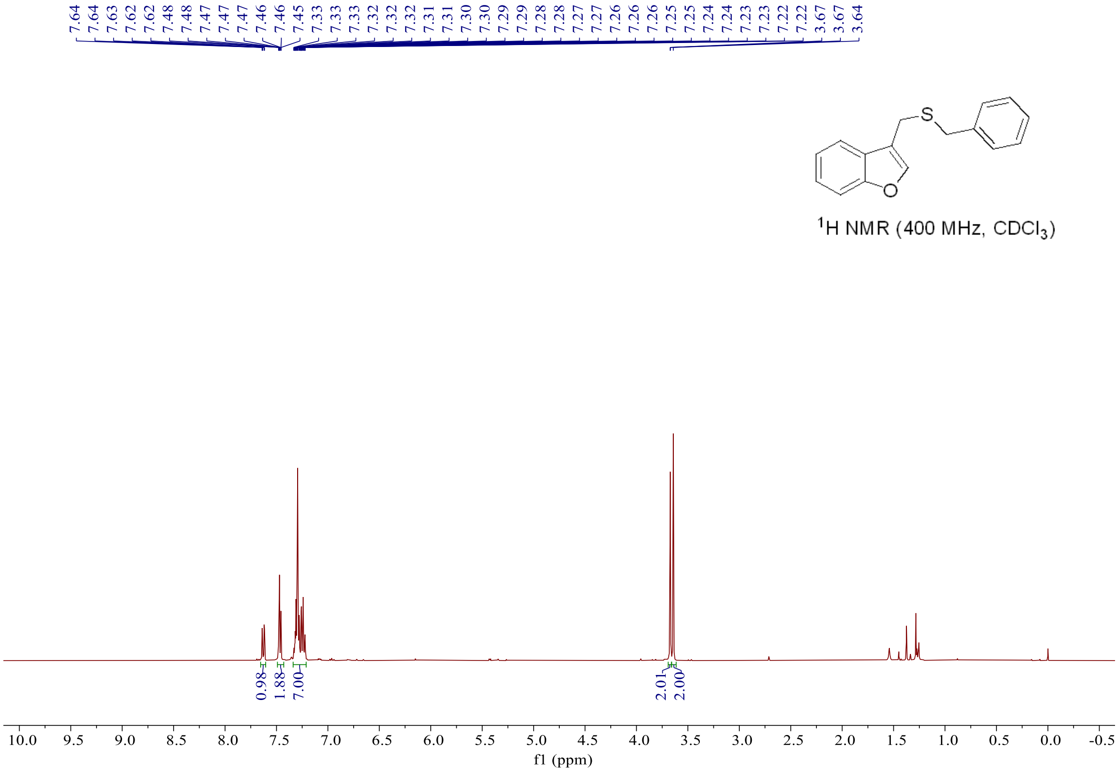

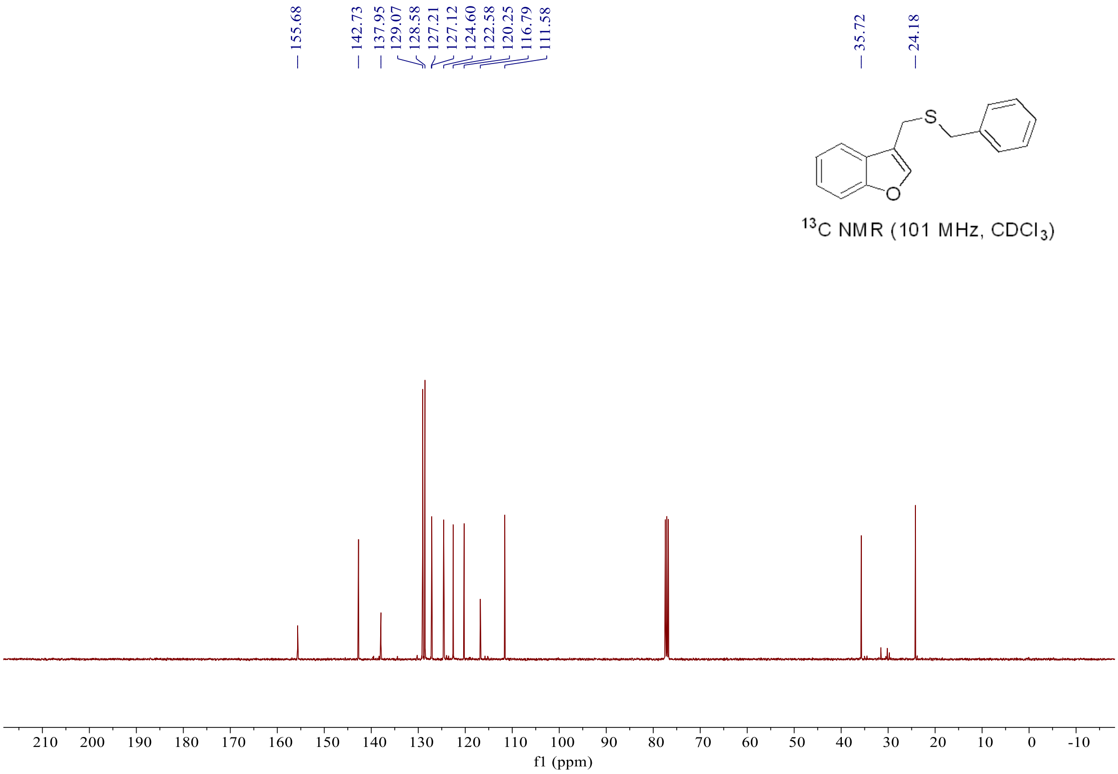

Supplementary Figure 64. ^{1}H and ^{13}C NMR spectra of **5q**.

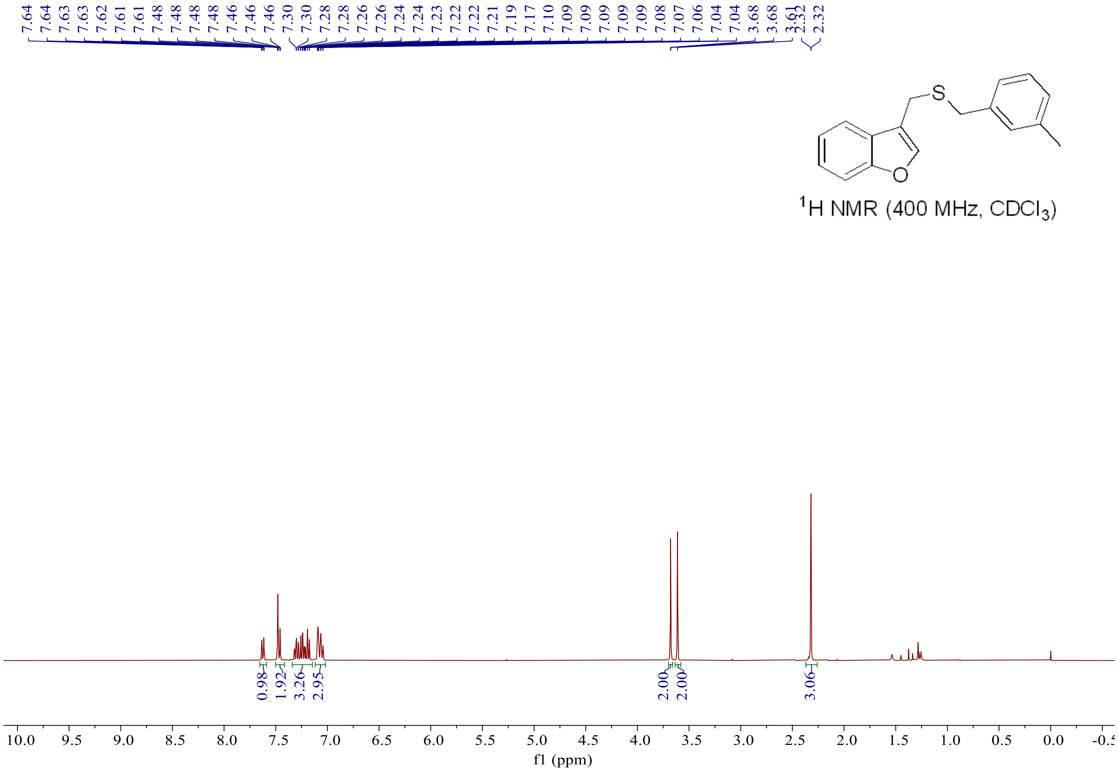

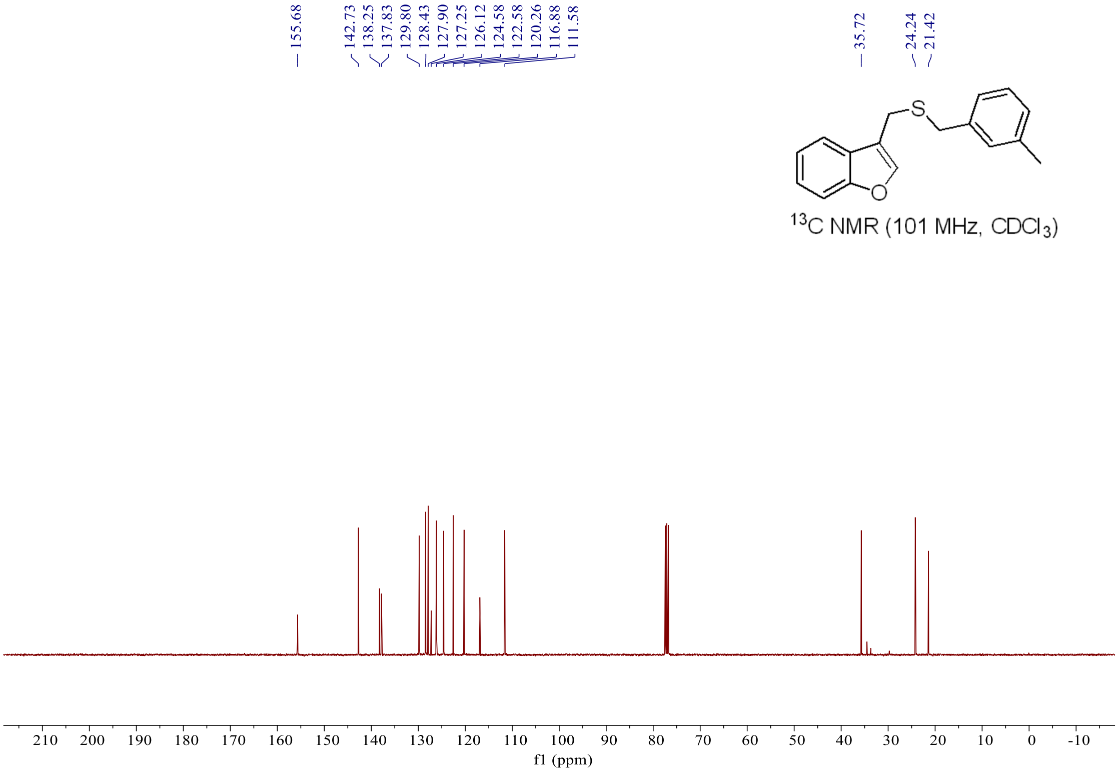

Supplementary Figure 65. ^{1}H and ^{13}C NMR spectra of **5r**.

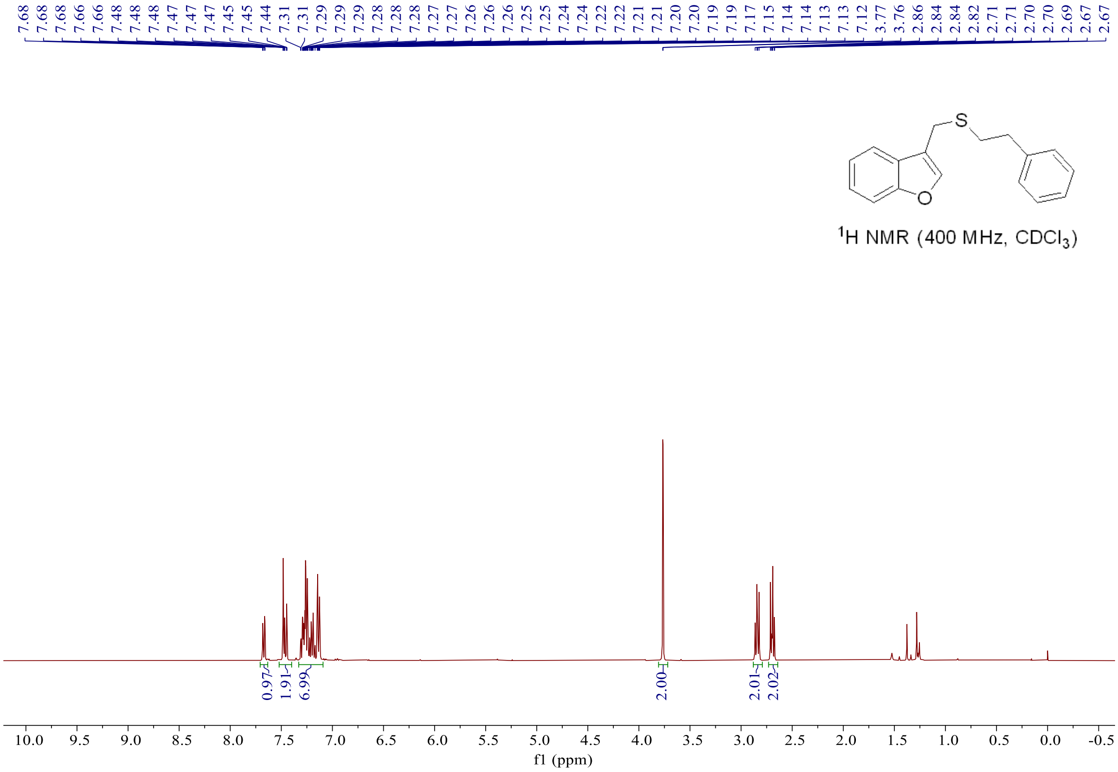

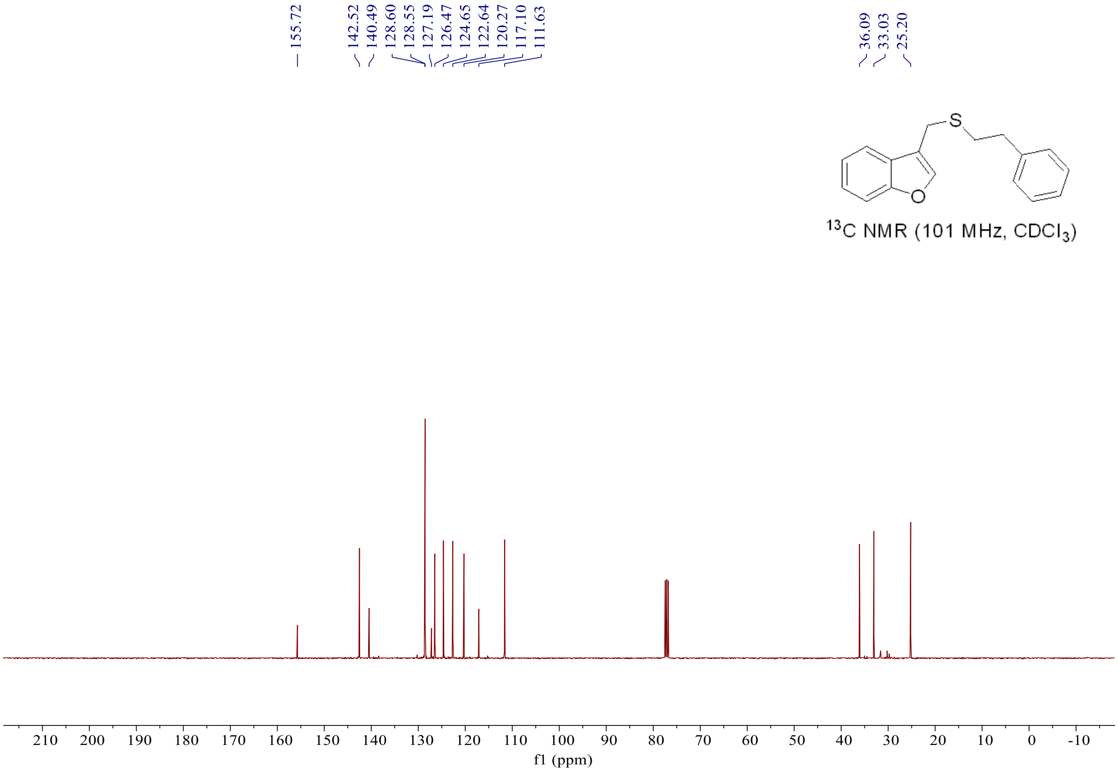

Supplementary Figure 66. ^{1}H and ^{13}C NMR spectra of **5s**.

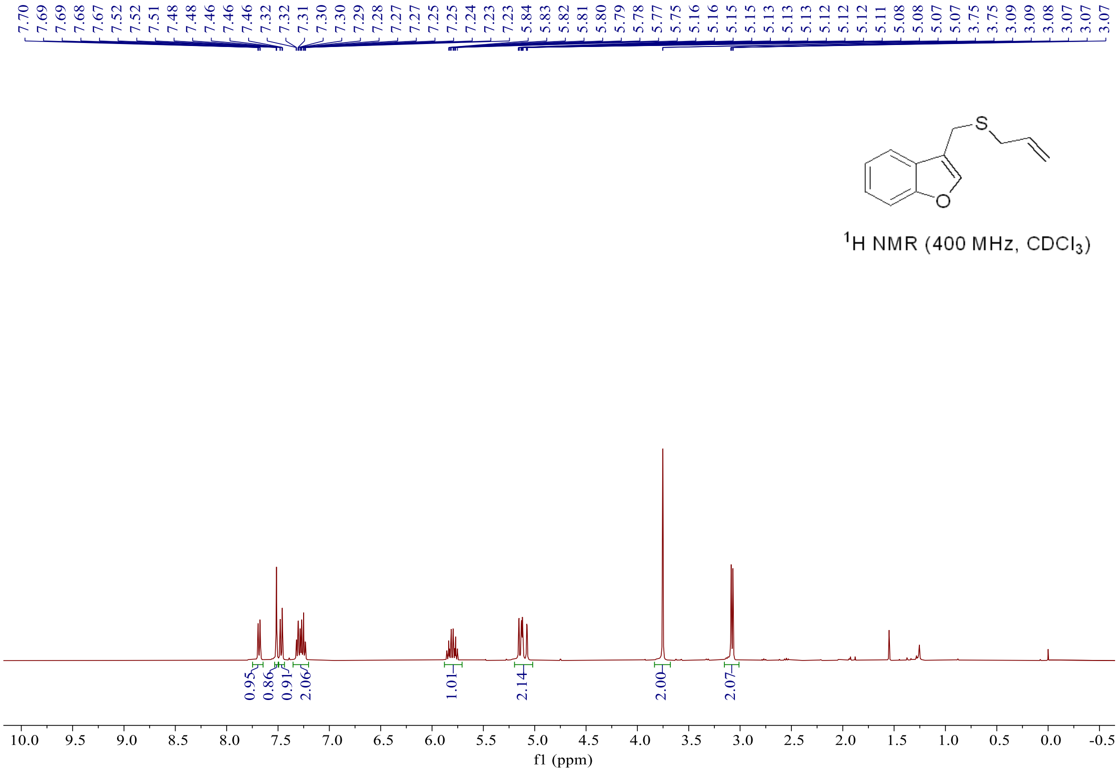

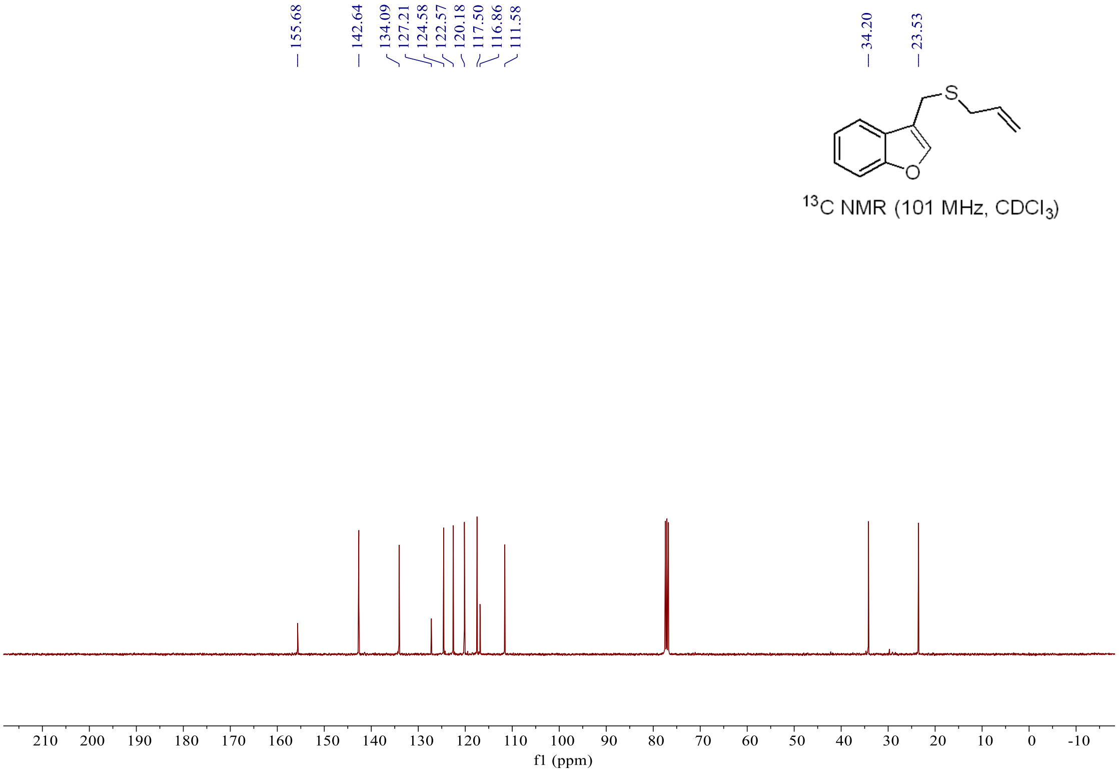

Supplementary Figure 67. ^{1}H and ^{13}C NMR spectra of **5t.**

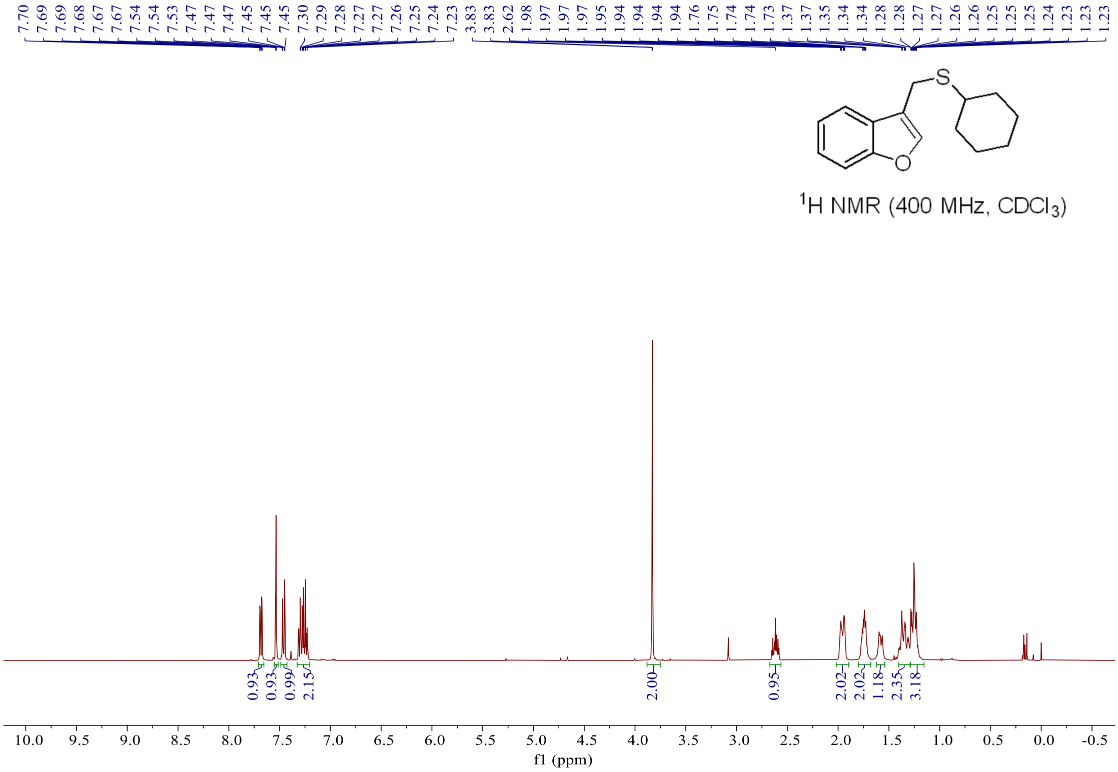

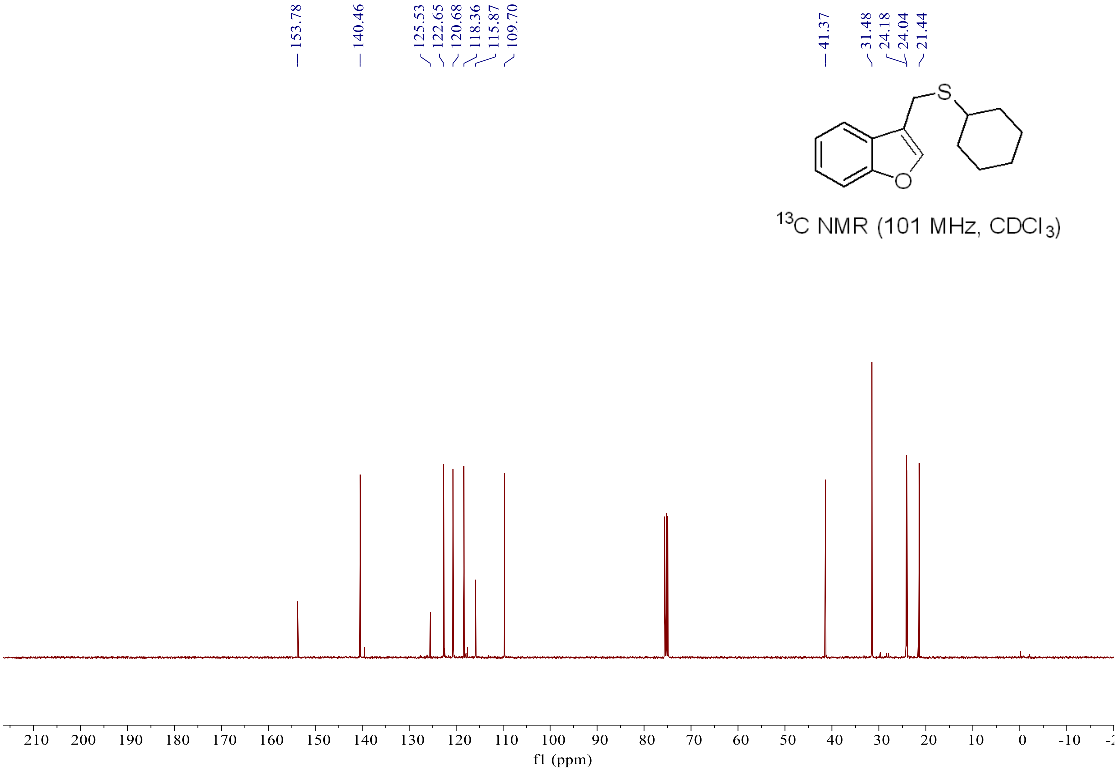

Supplementary Figure 68. ^{1}H and ^{13}C NMR spectra of **5u**.

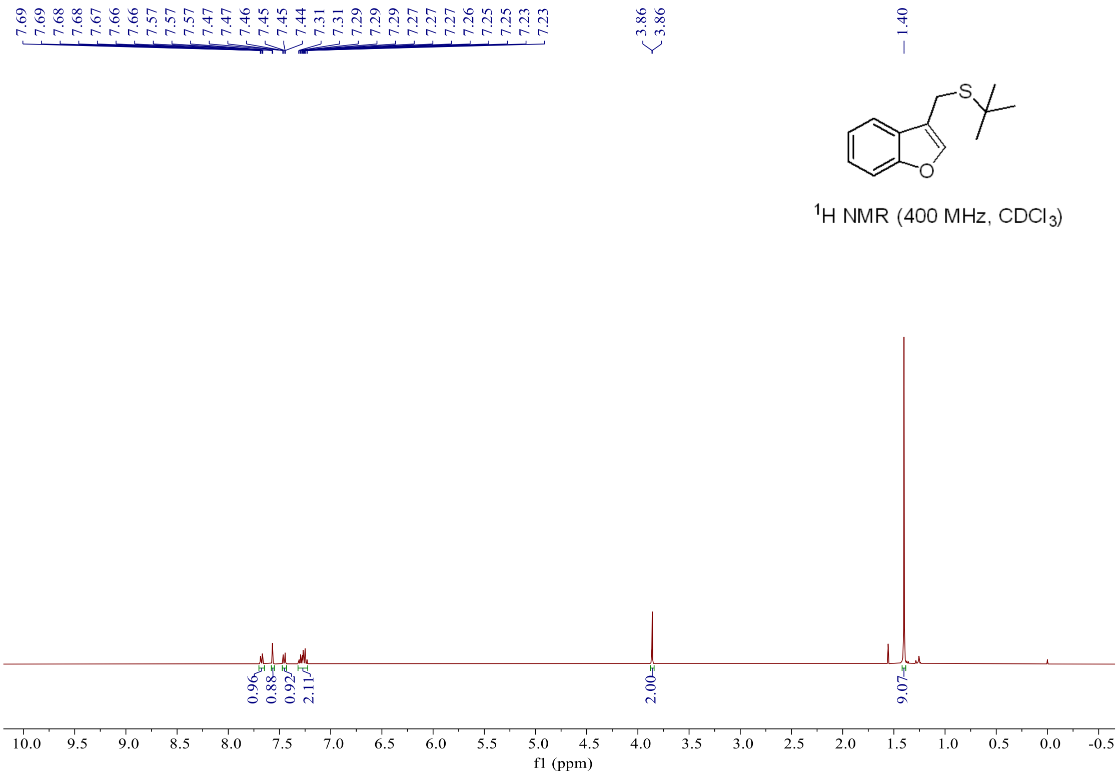

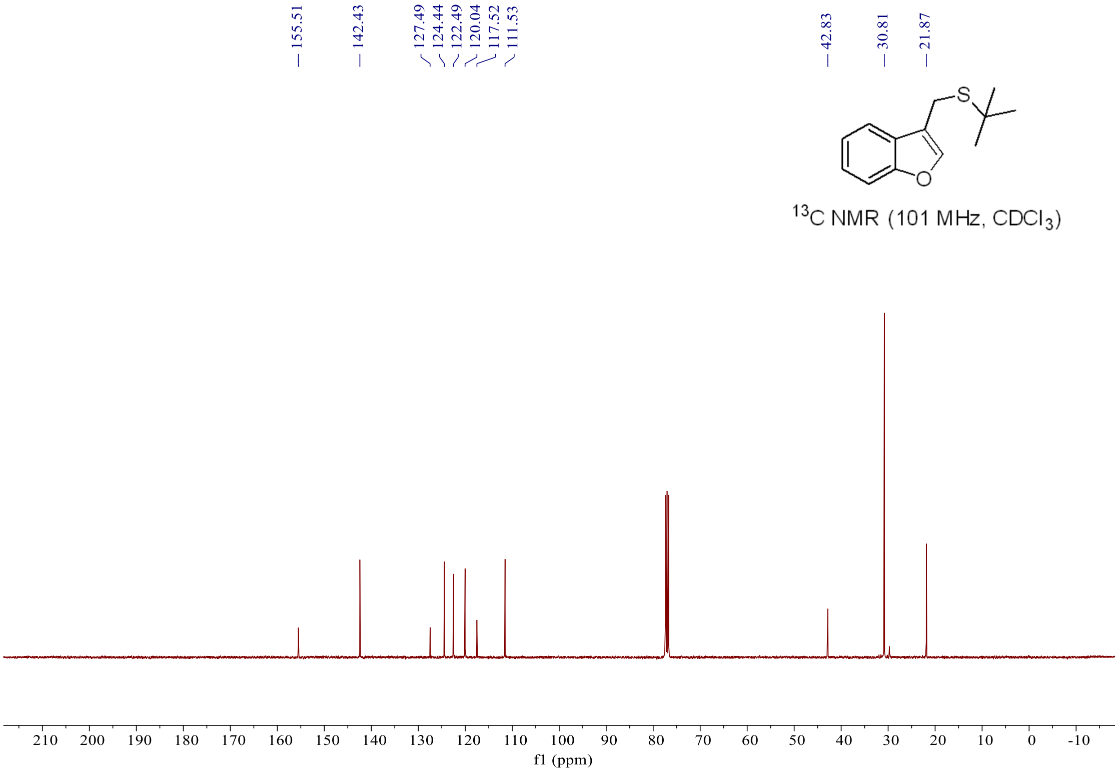

Supplementary Figure 69. ^{1}H and ^{13}C NMR spectra of **5v**.

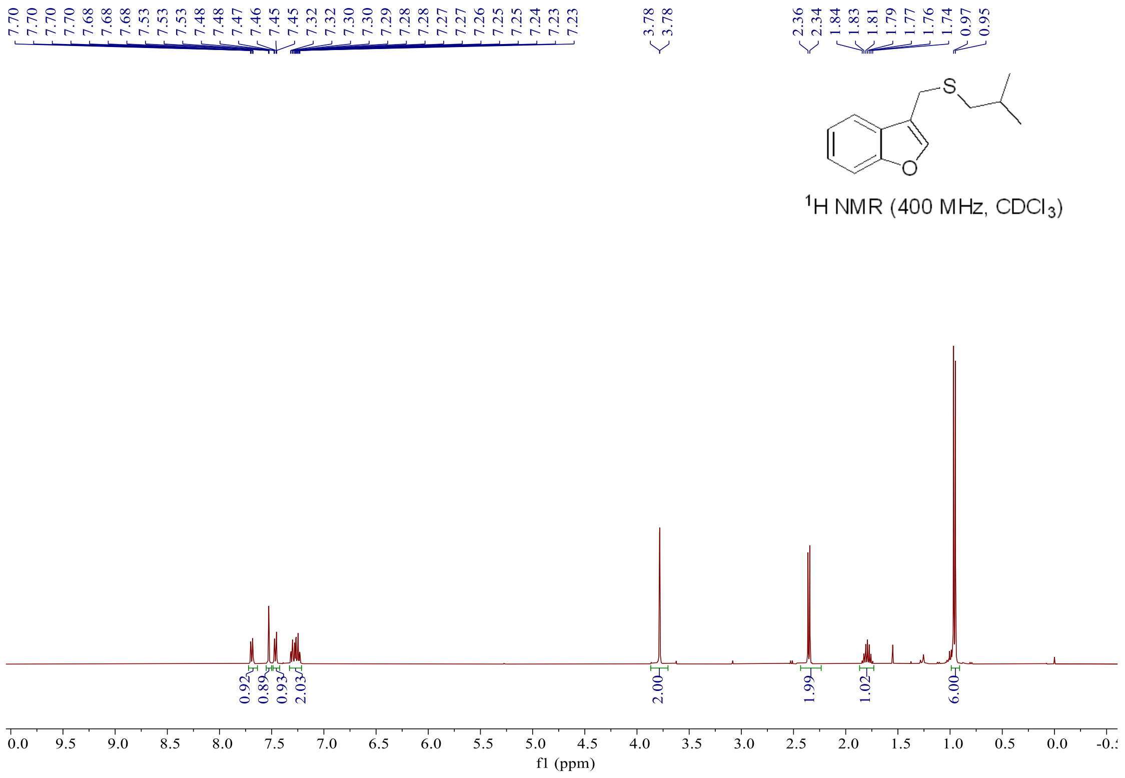

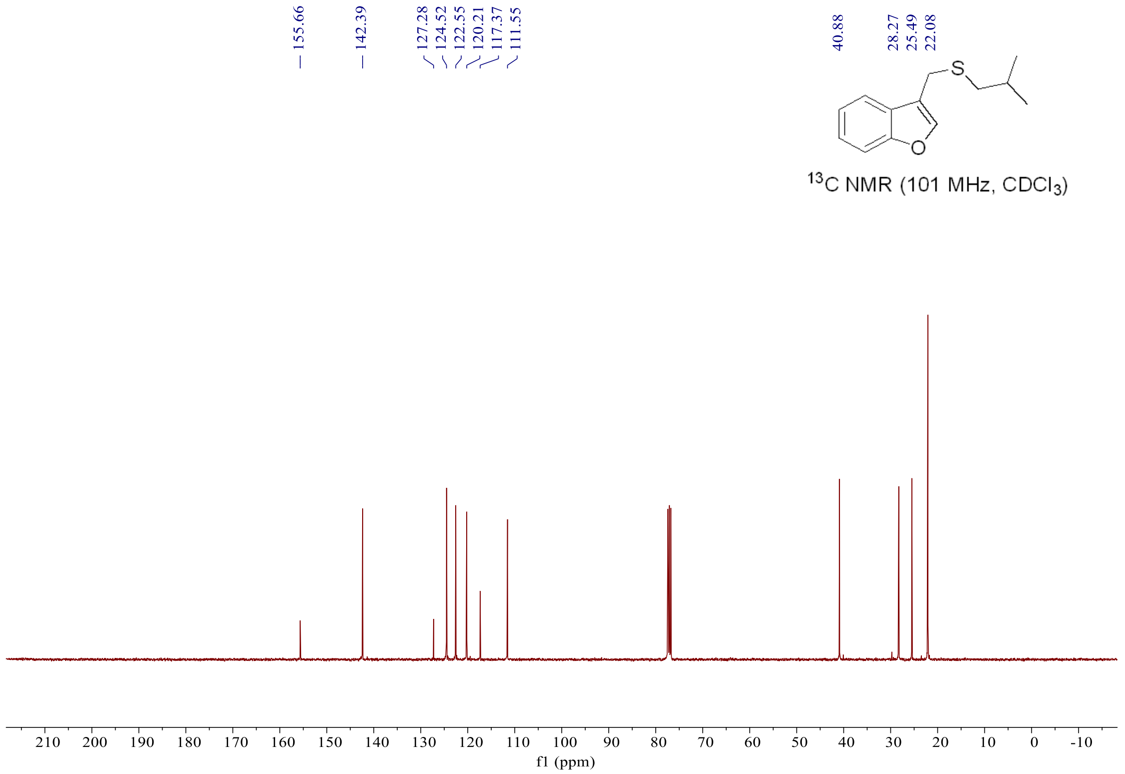

Supplementary Figure 70. ^{1}H and ^{13}C NMR spectra of **5w.**

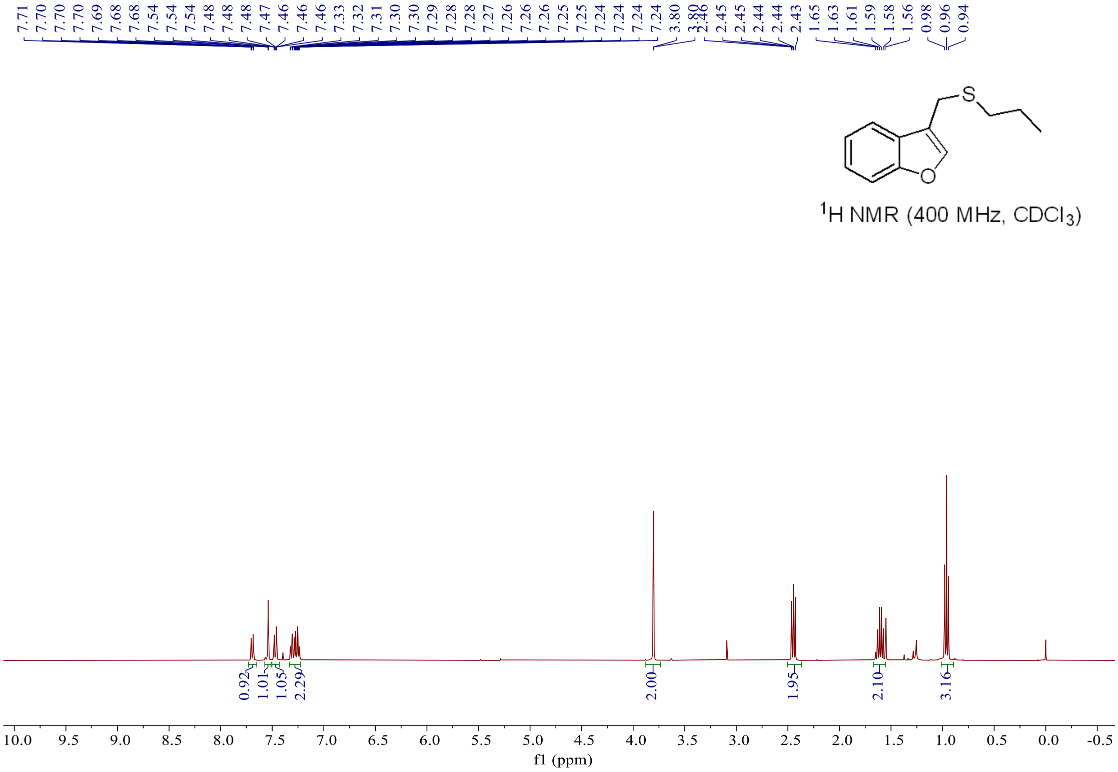

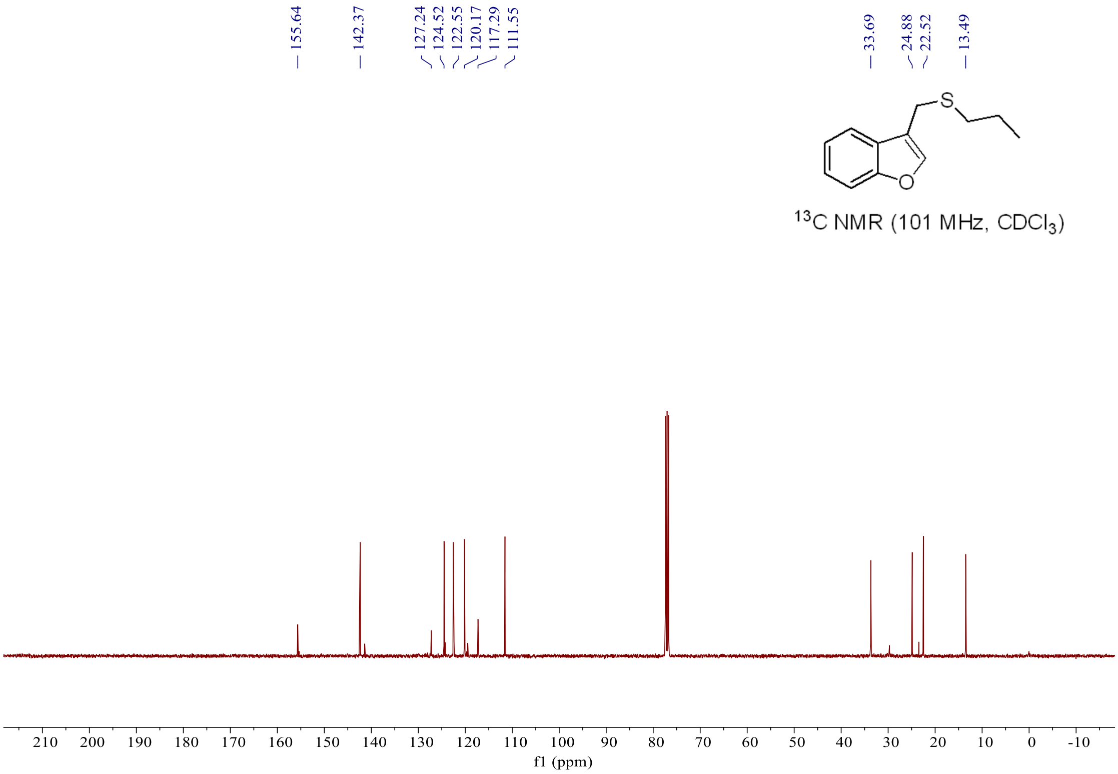

Supplementary Figure 71. ^{1}H and ^{13}C NMR spectra of **5x.**

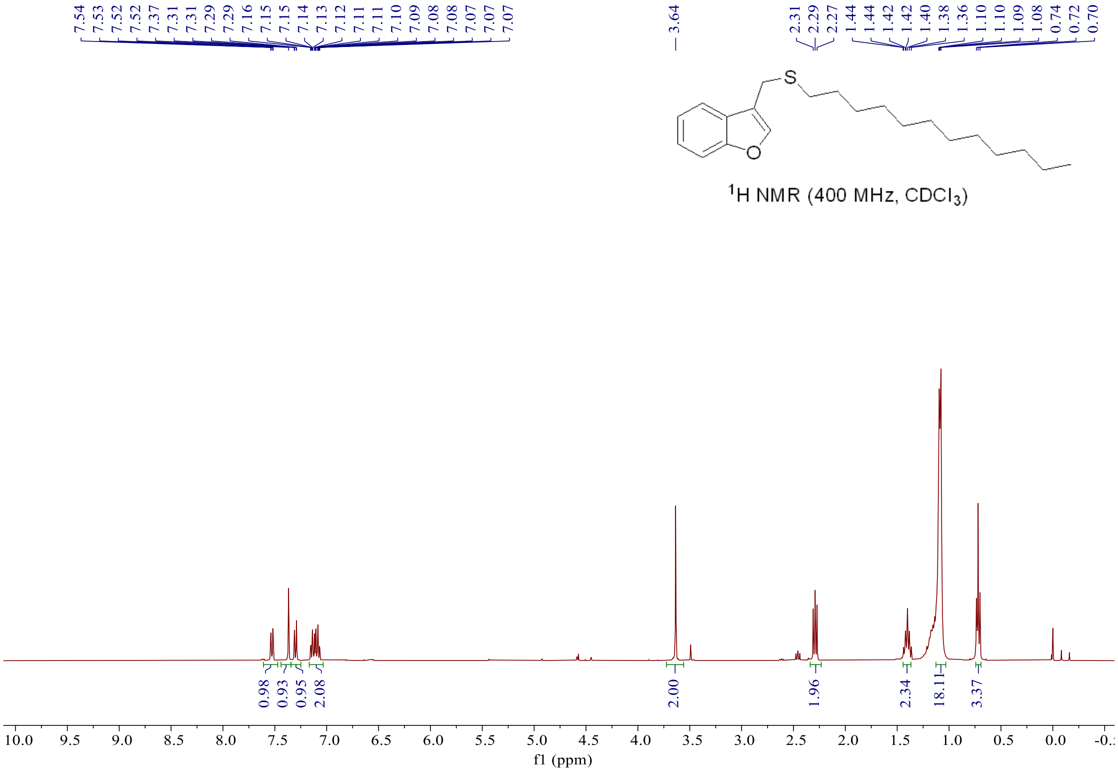

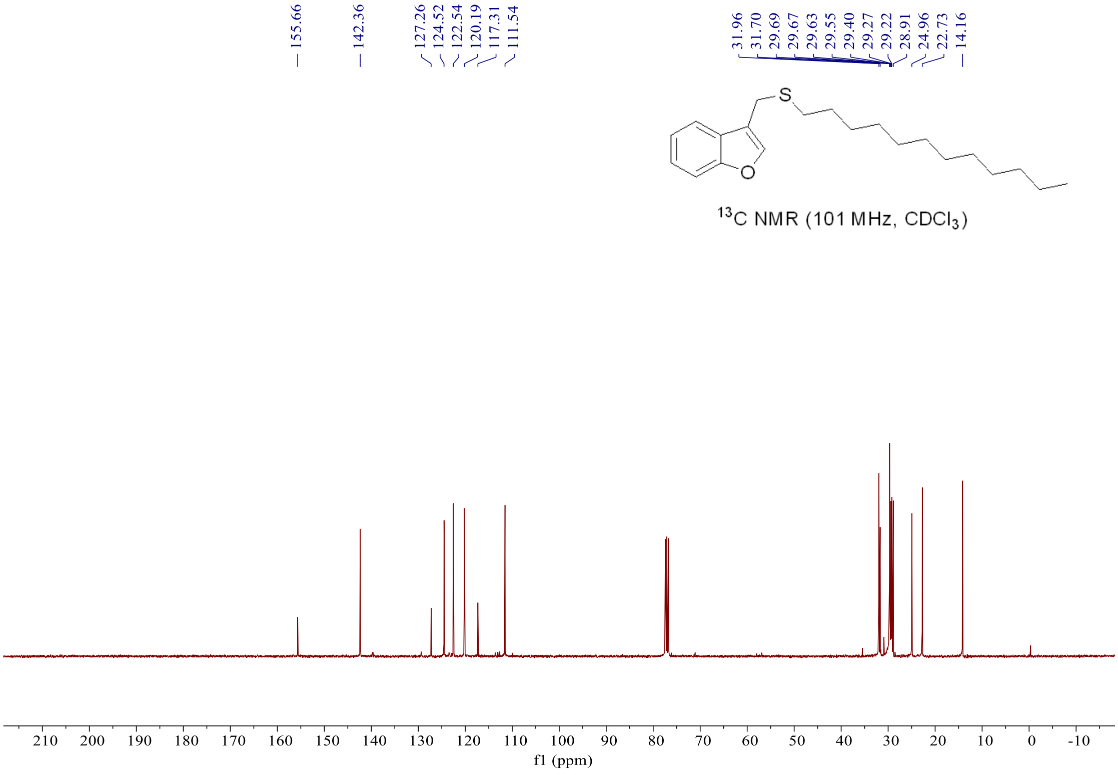

Supplementary Figure 72. ^{1}H and ^{13}C NMR spectra of **5y**.

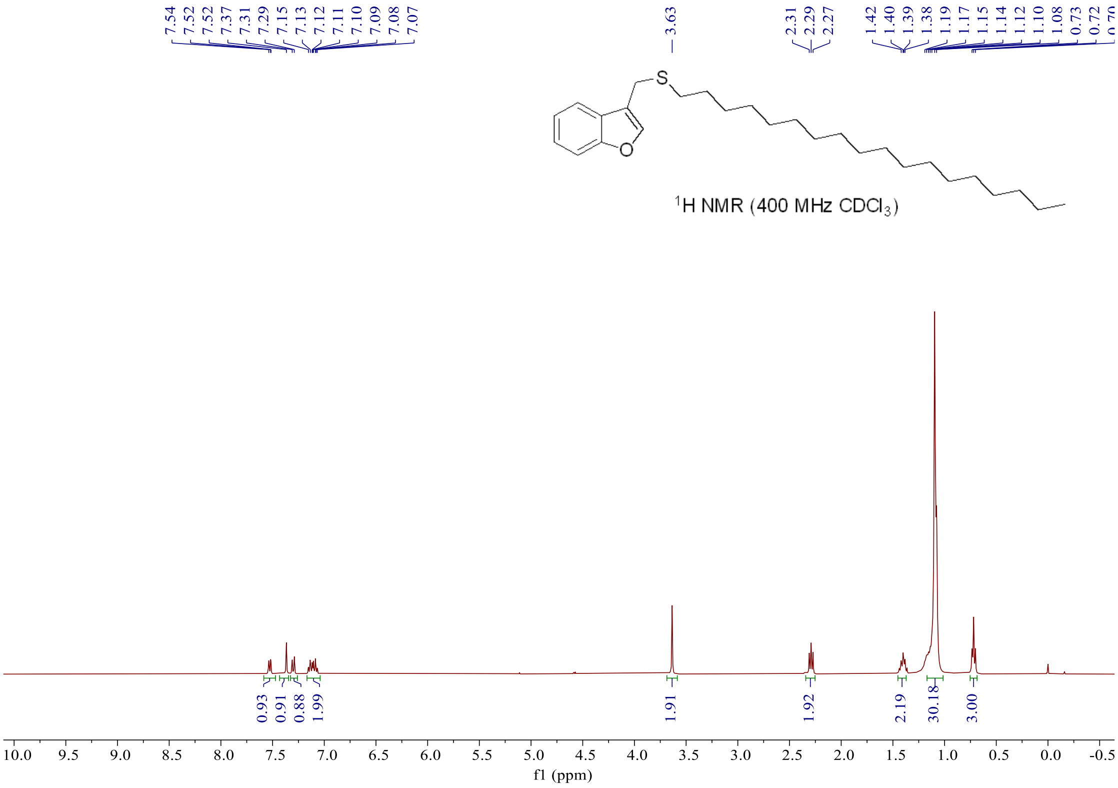

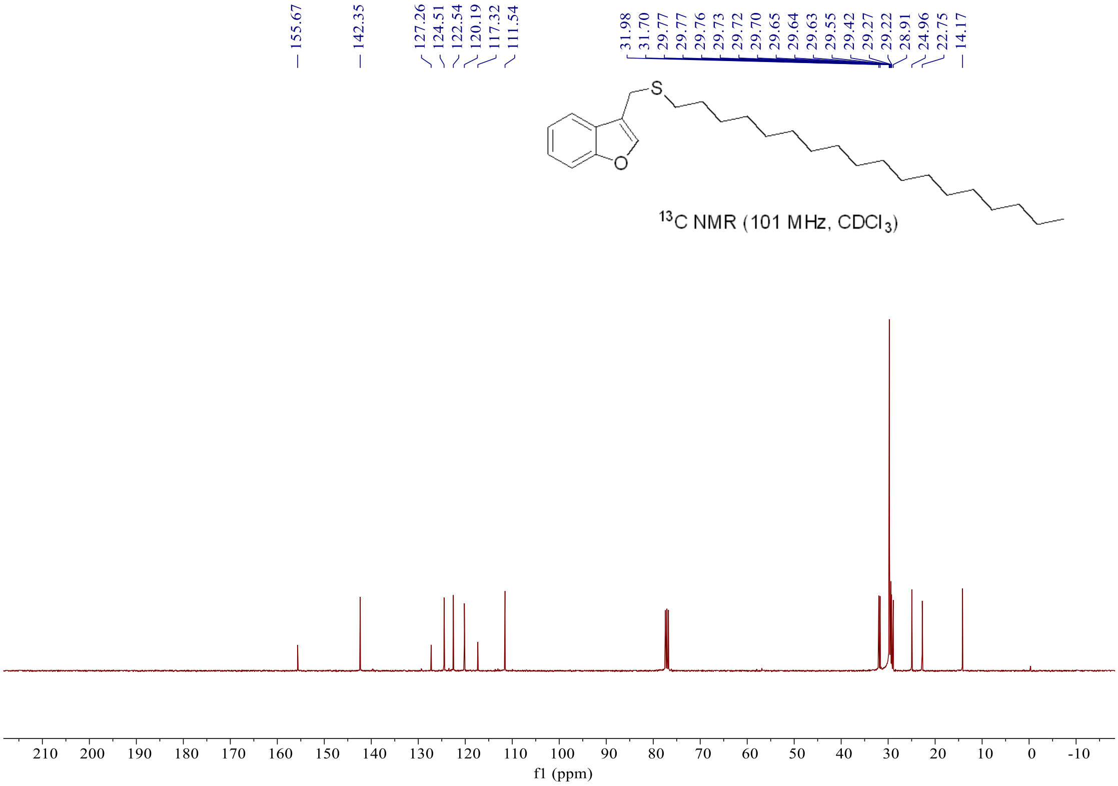

Supplementary Figure 73. ^{1}H and ^{13}C NMR spectra of **7a**.

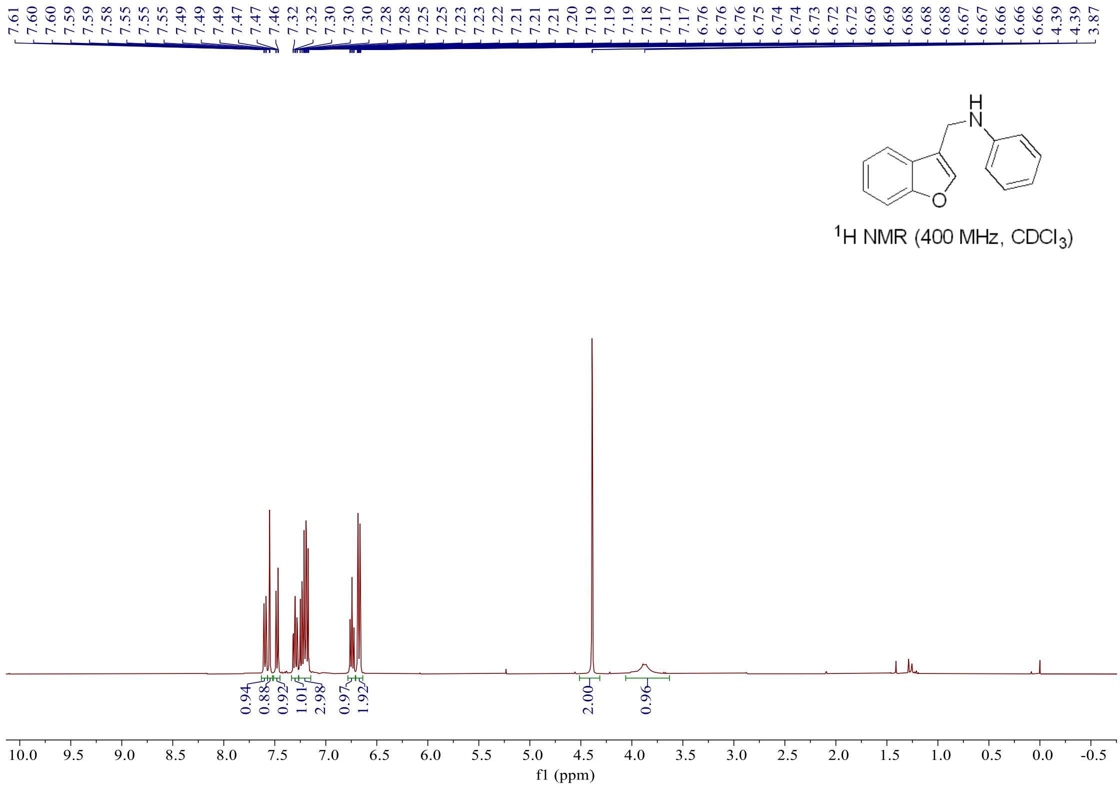

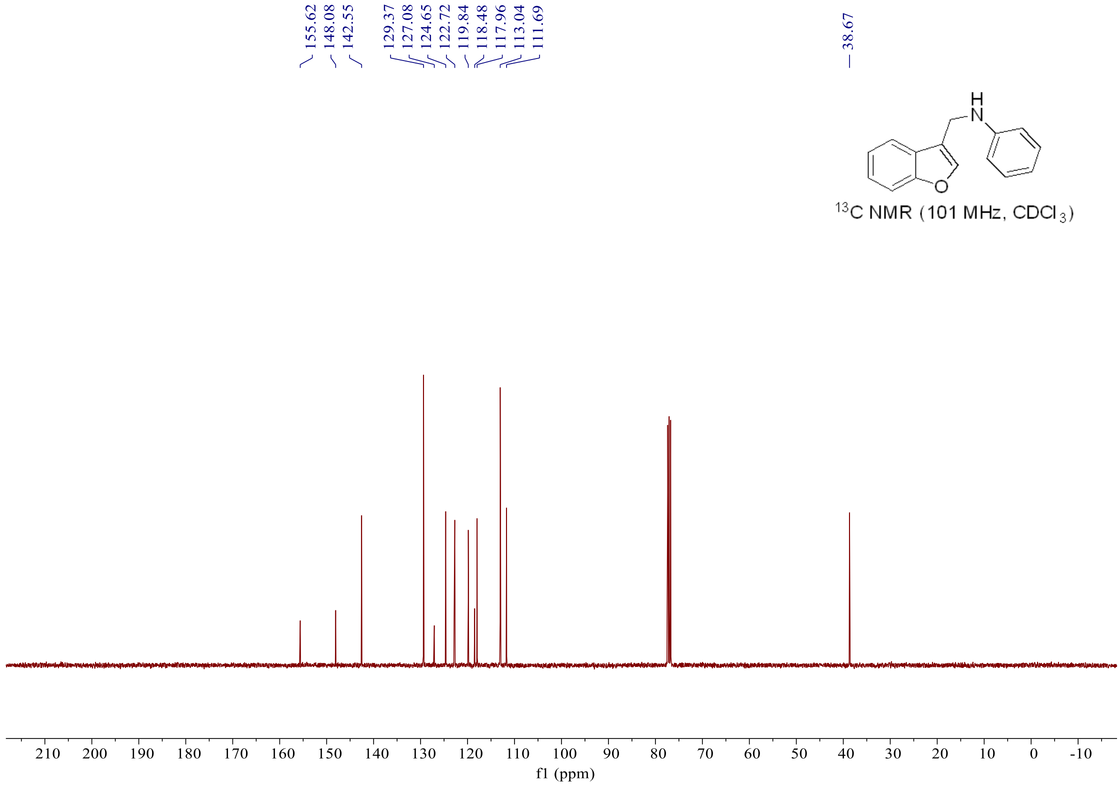

Supplementary Figure 74. ^{1}H and ^{13}C NMR spectra of **7b**.

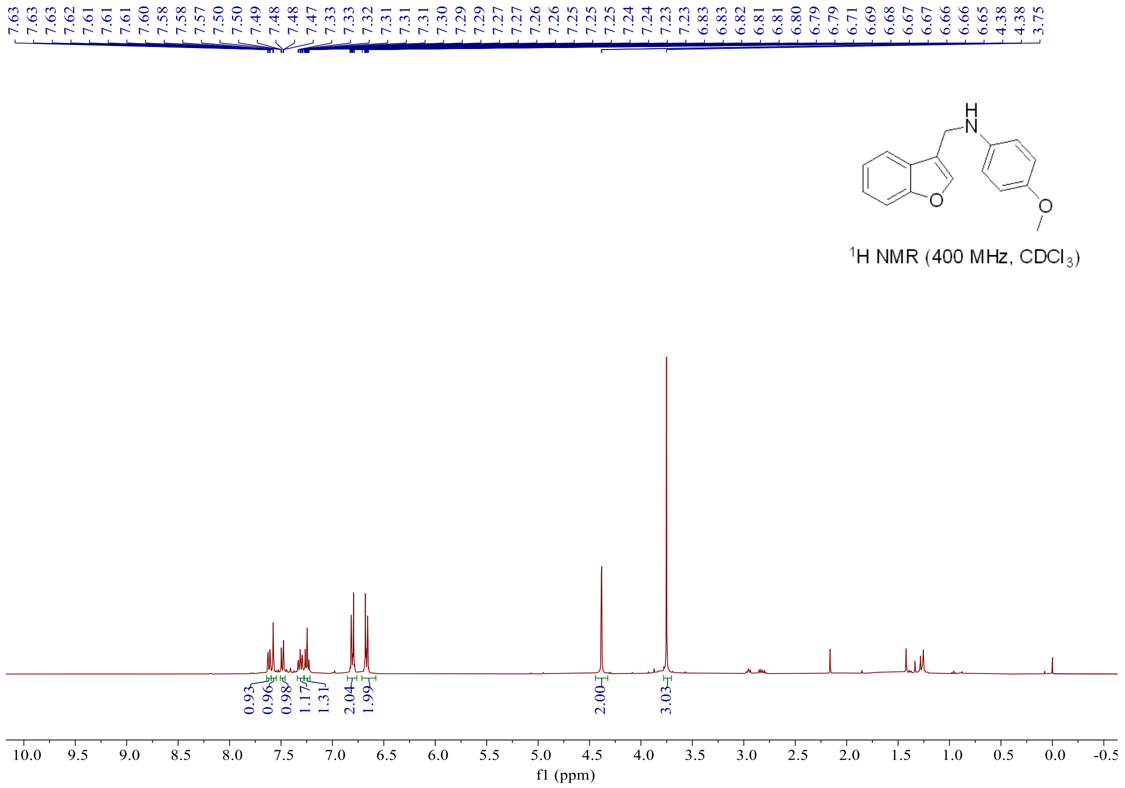

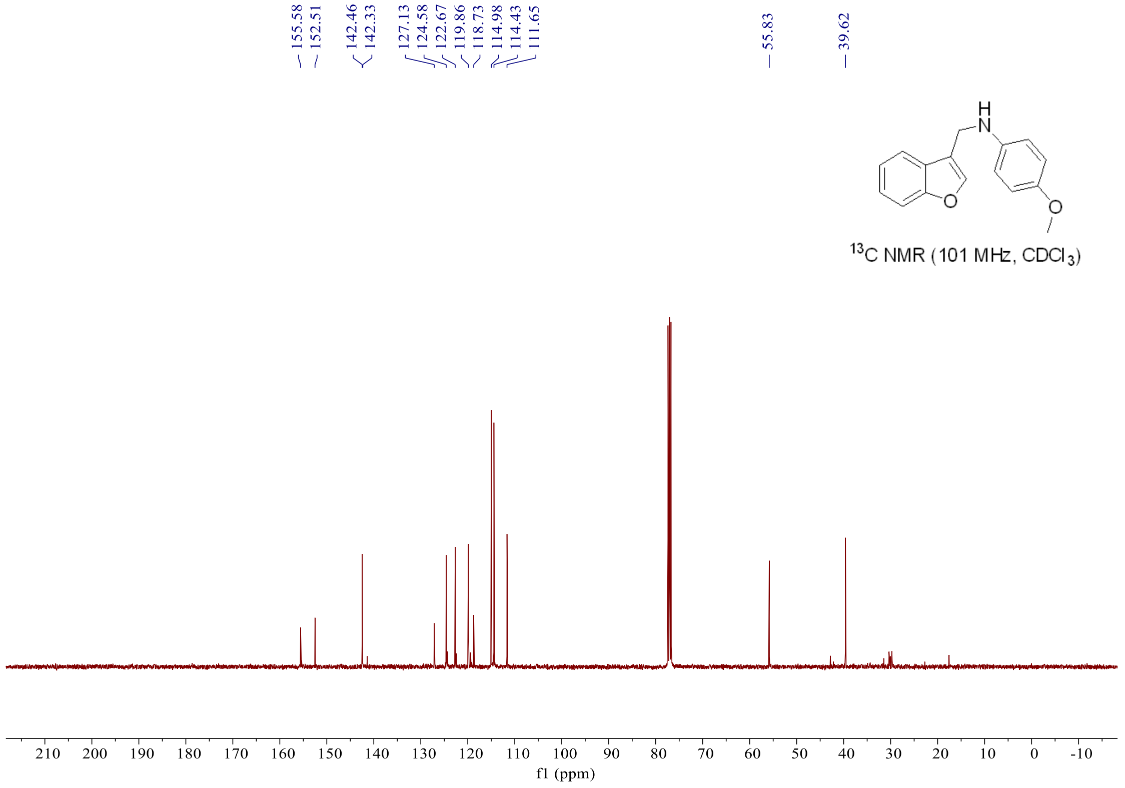

Supplementary Figure 75. ^1H and ^{13}C NMR spectra of **7c.**

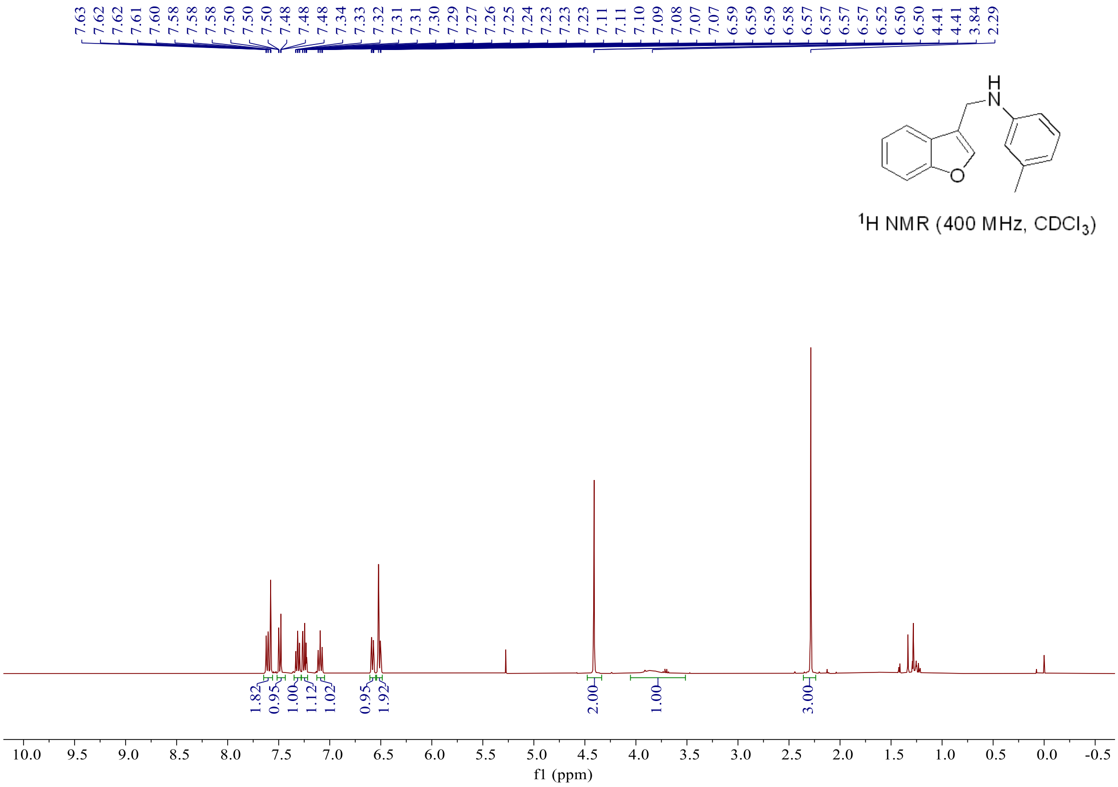

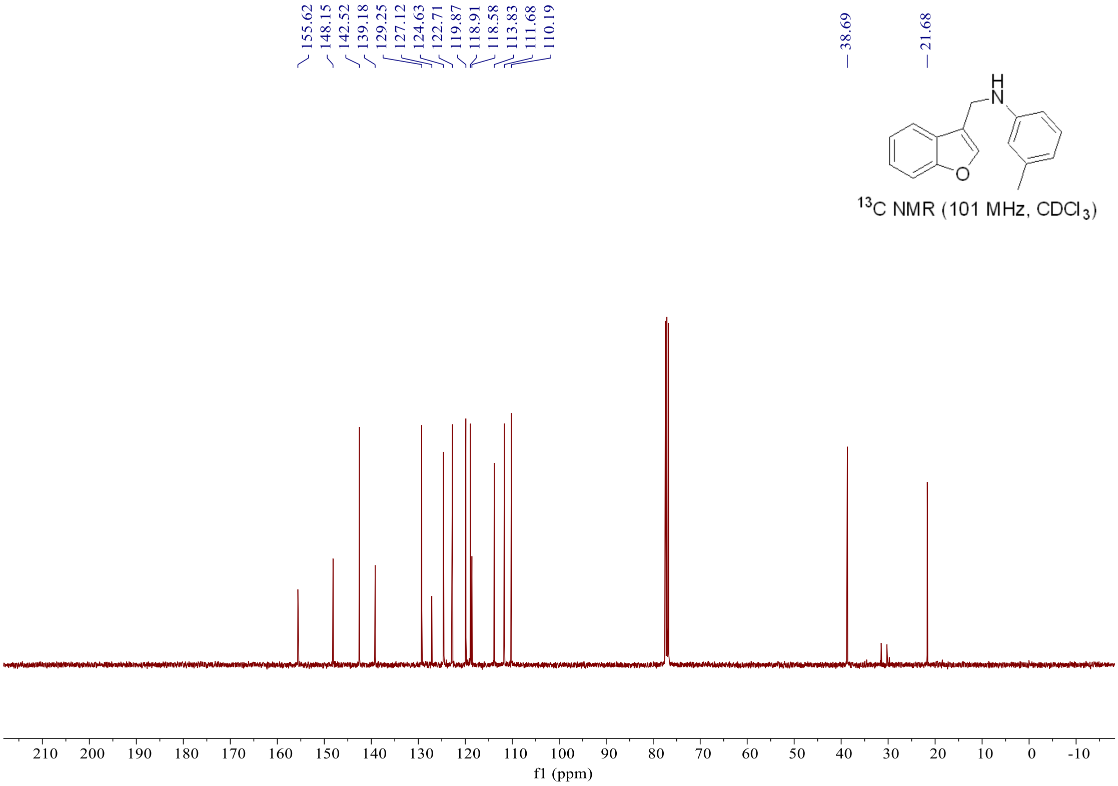

Supplementary Figure 76. ^{1}H, ^{13}C and ^{19}F NMR spectra of **7d**.

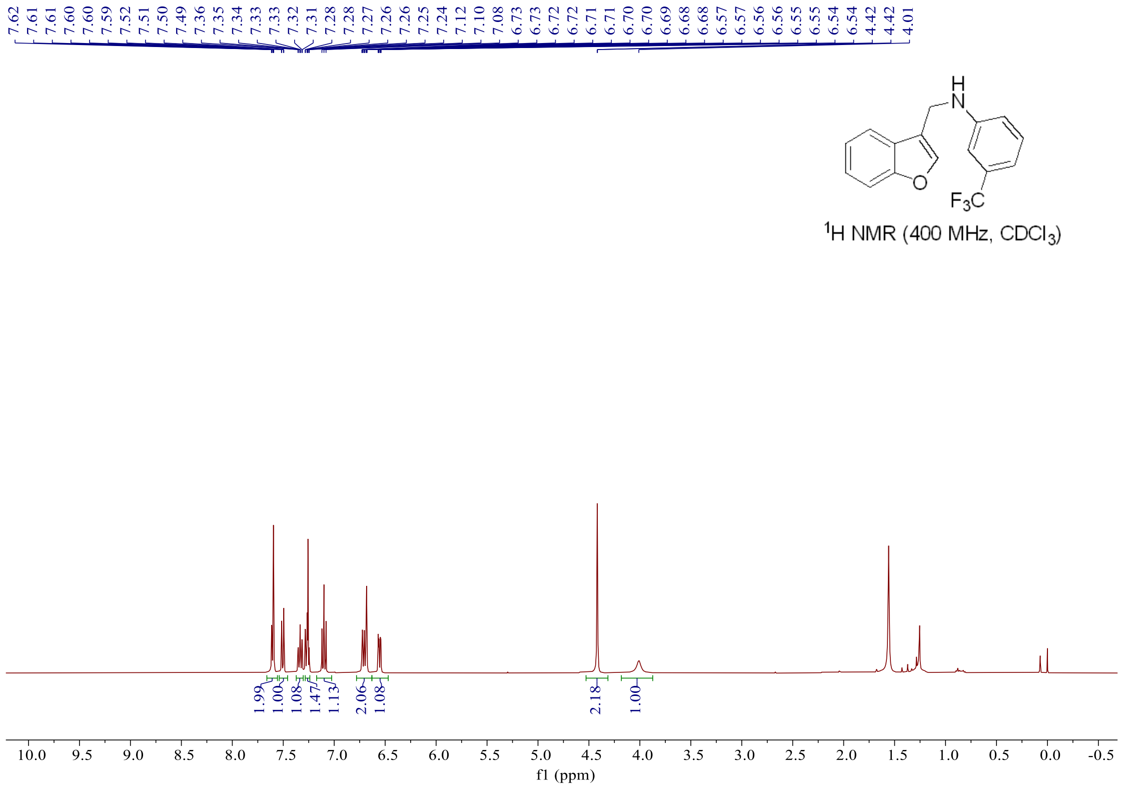

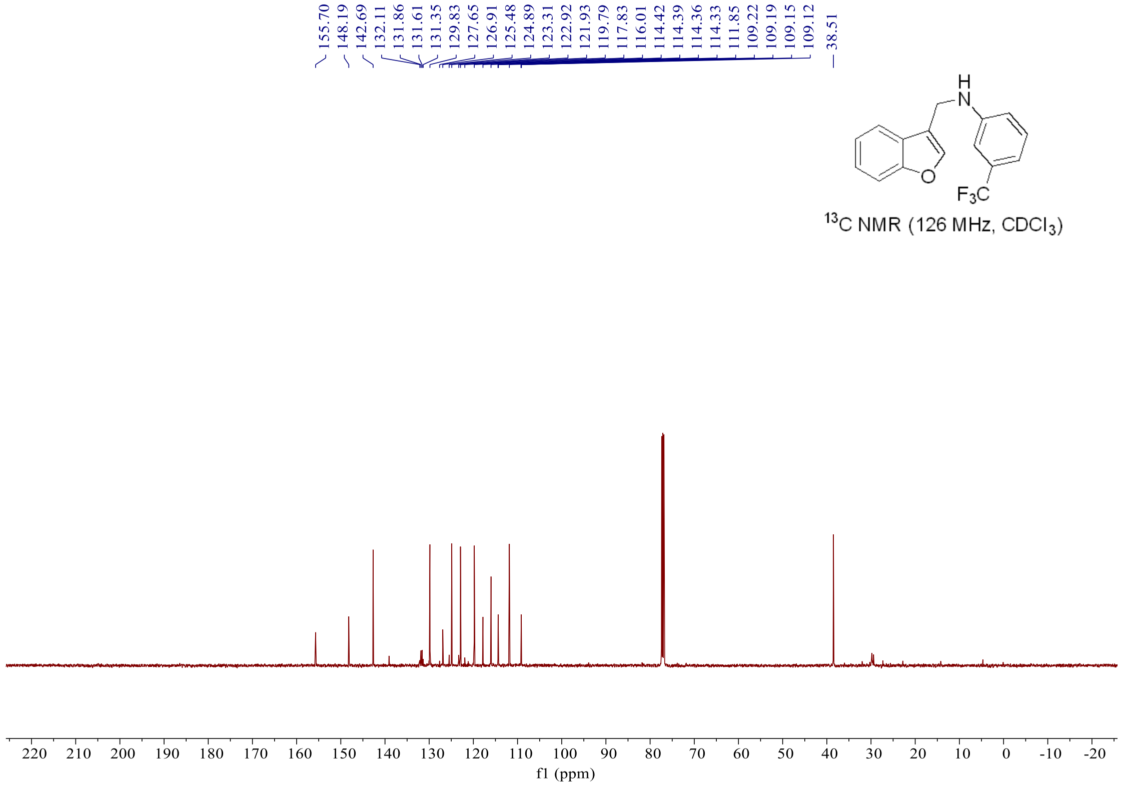

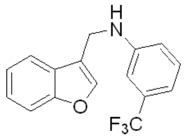

^{19}F NMR (377 MHz, $CDCl_3$)

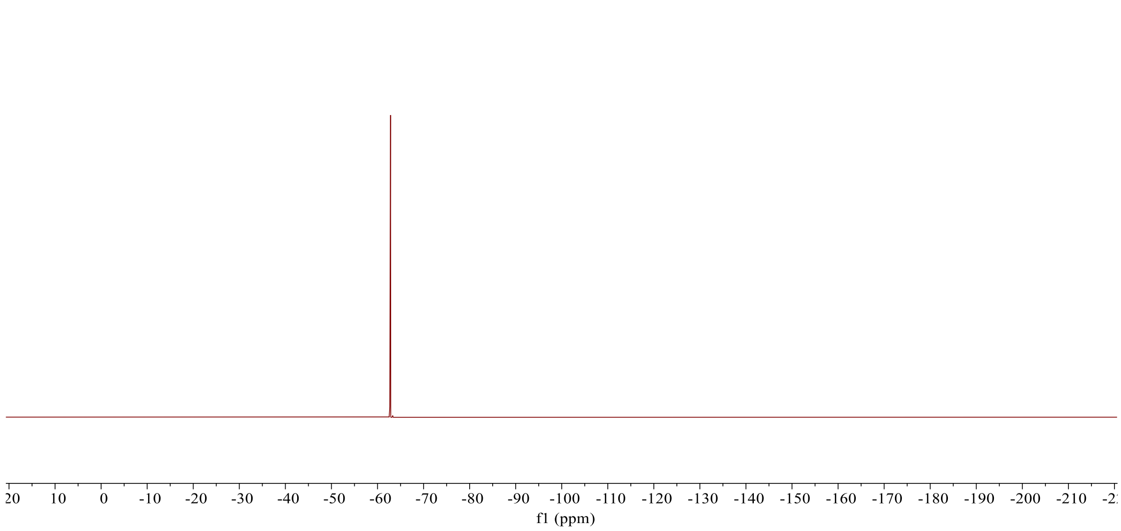

Supplementary Figure 77. ^{1}H and ^{13}C NMR spectra of **7e**.

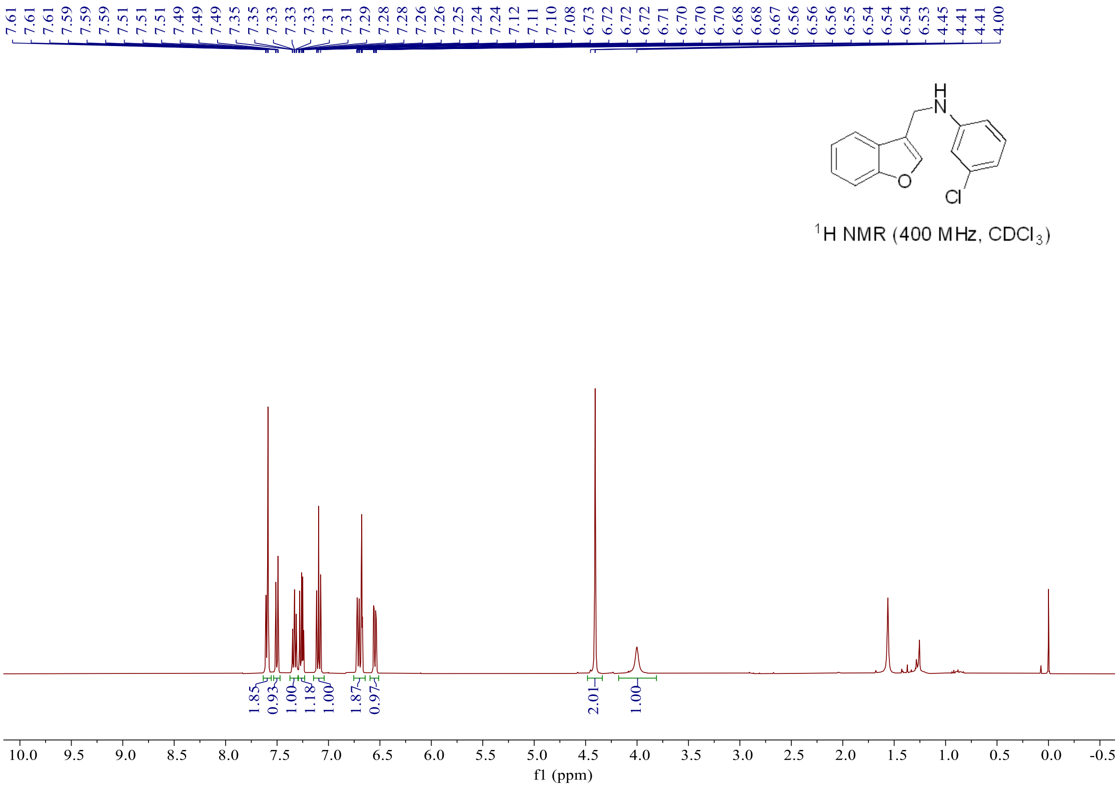

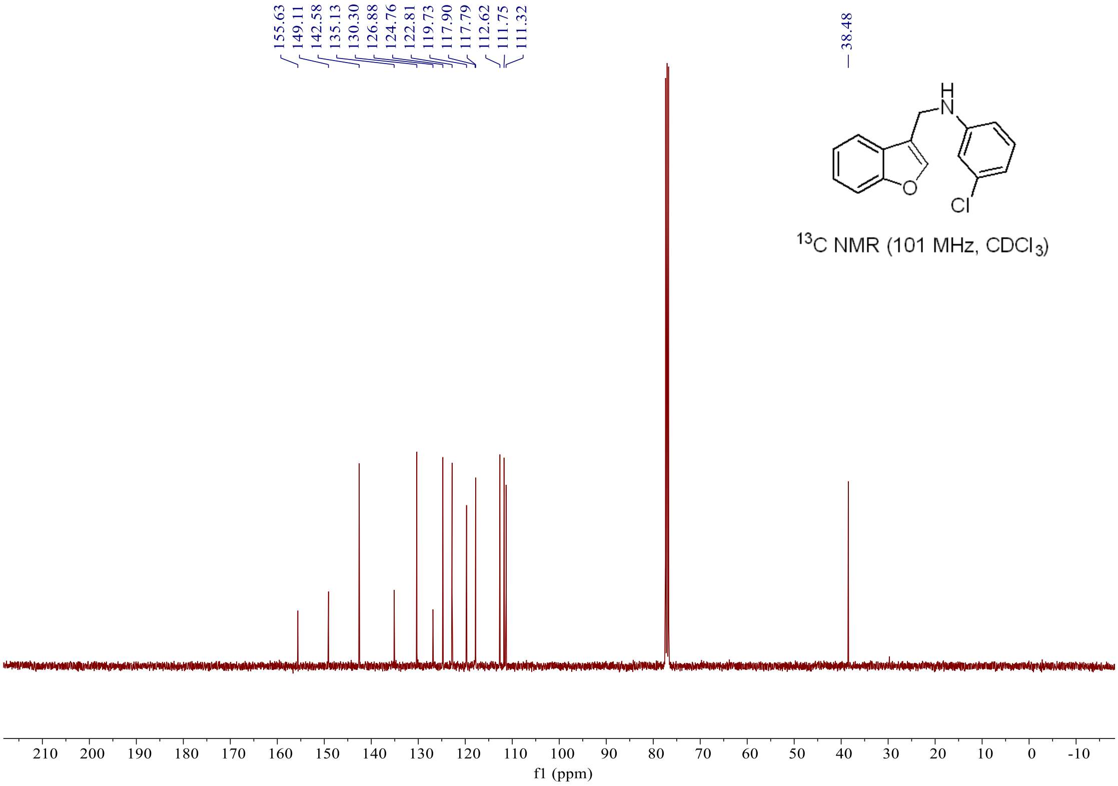

Supplementary Figure 78. ^{1}H and ^{13}C NMR spectra of **7f.**

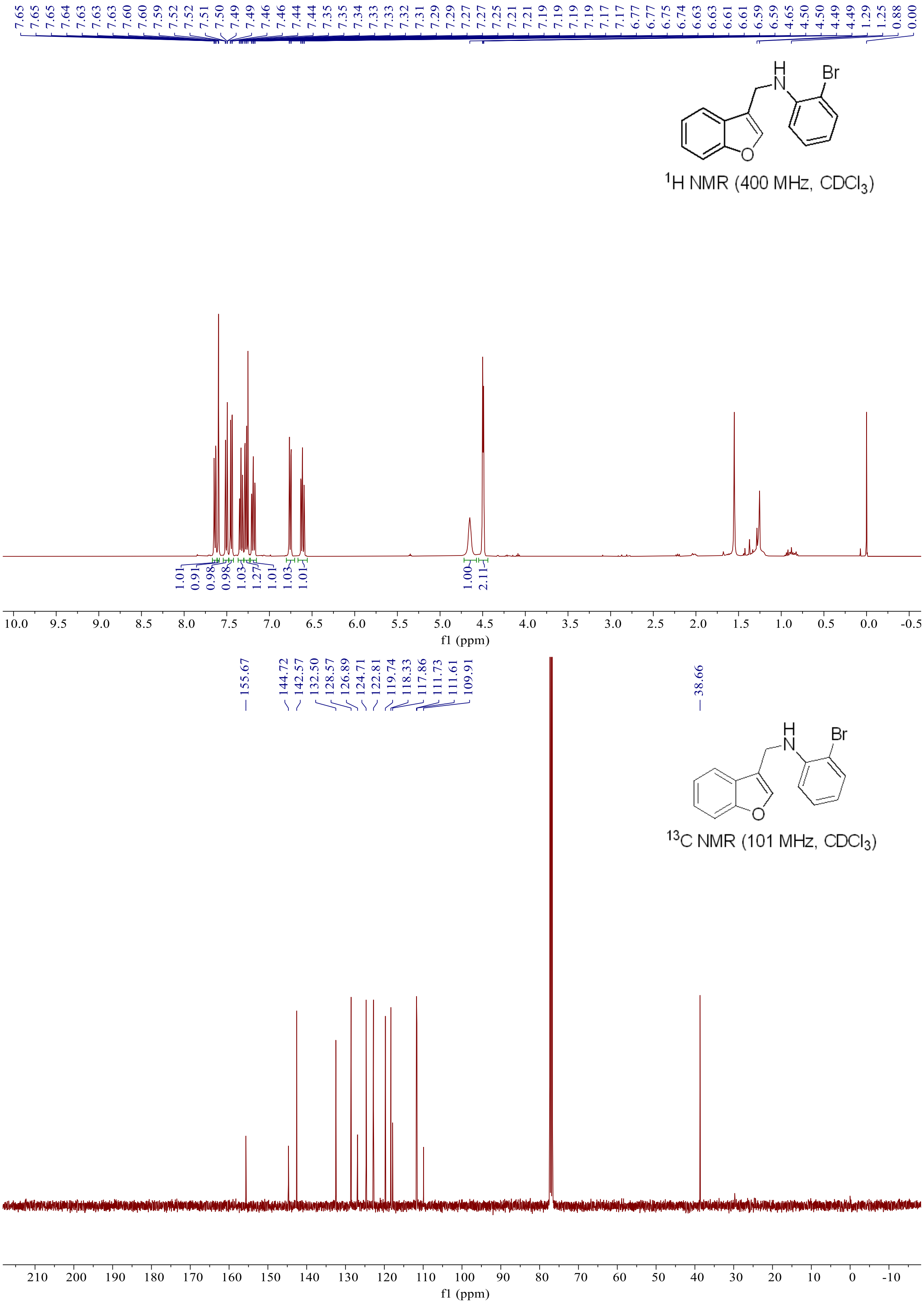

Supplementary Figure 79. ^{1}H and ^{13}C NMR spectra of **7g**.

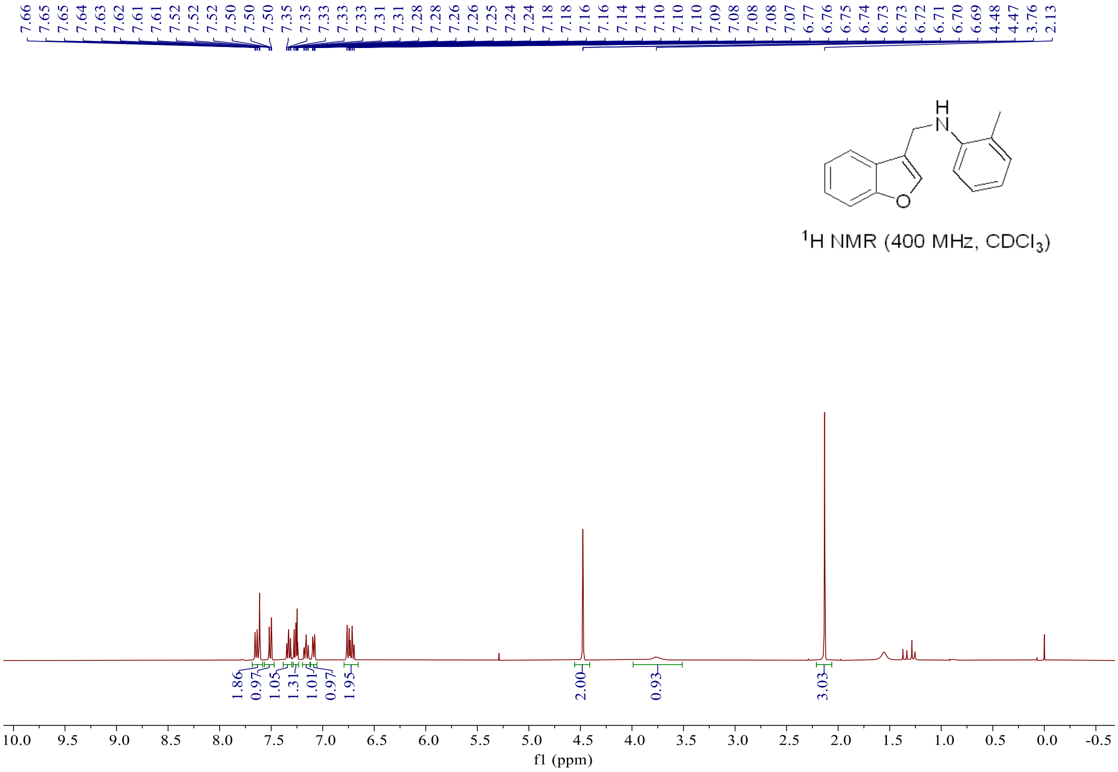

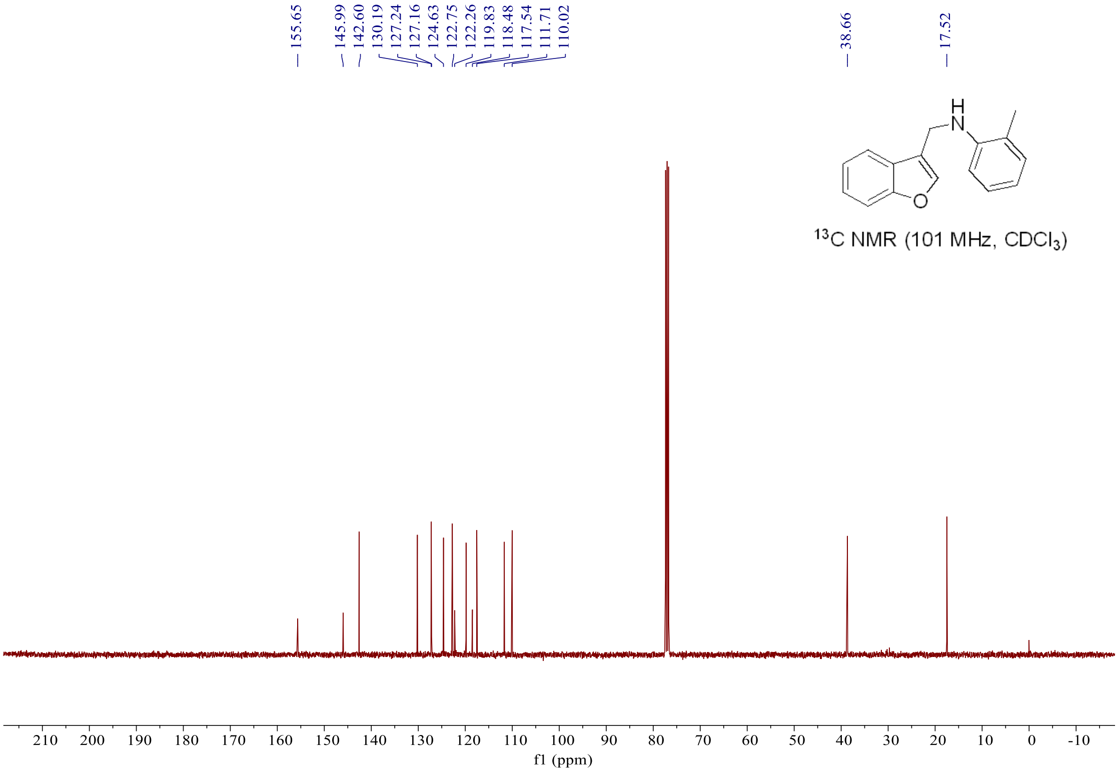

Supplementary Figure 80. ^{1}H and ^{13}C NMR spectra of **7h**.

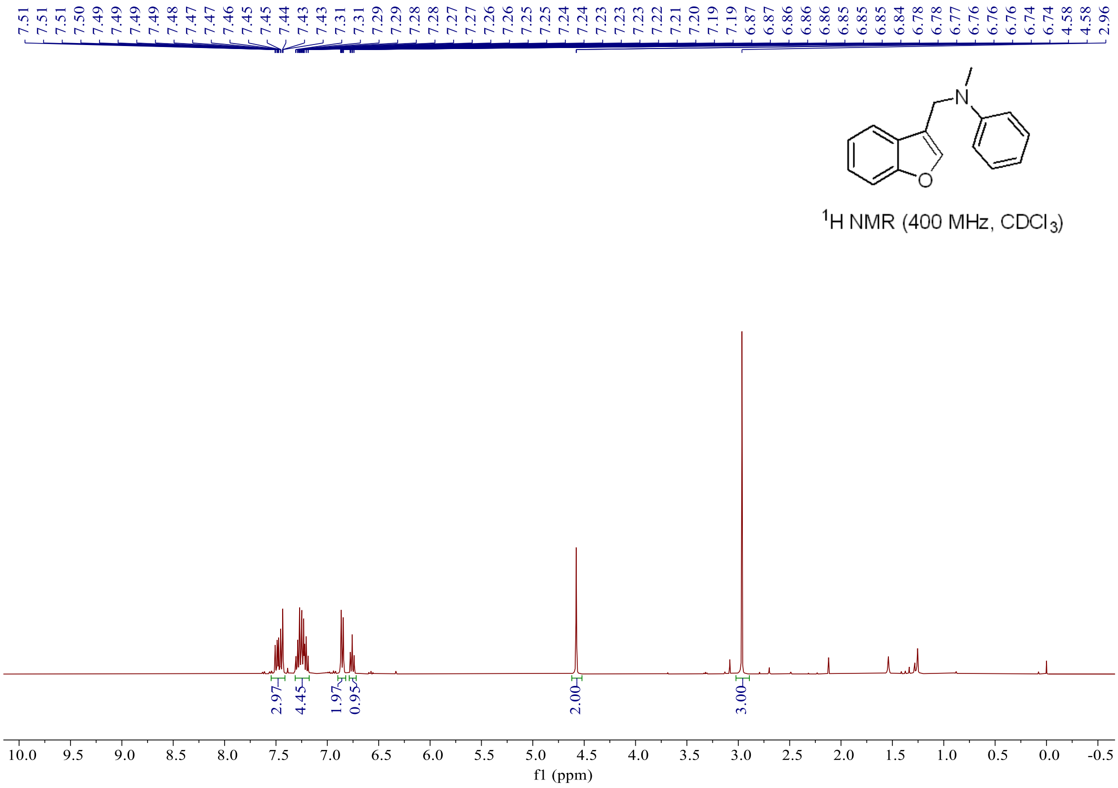

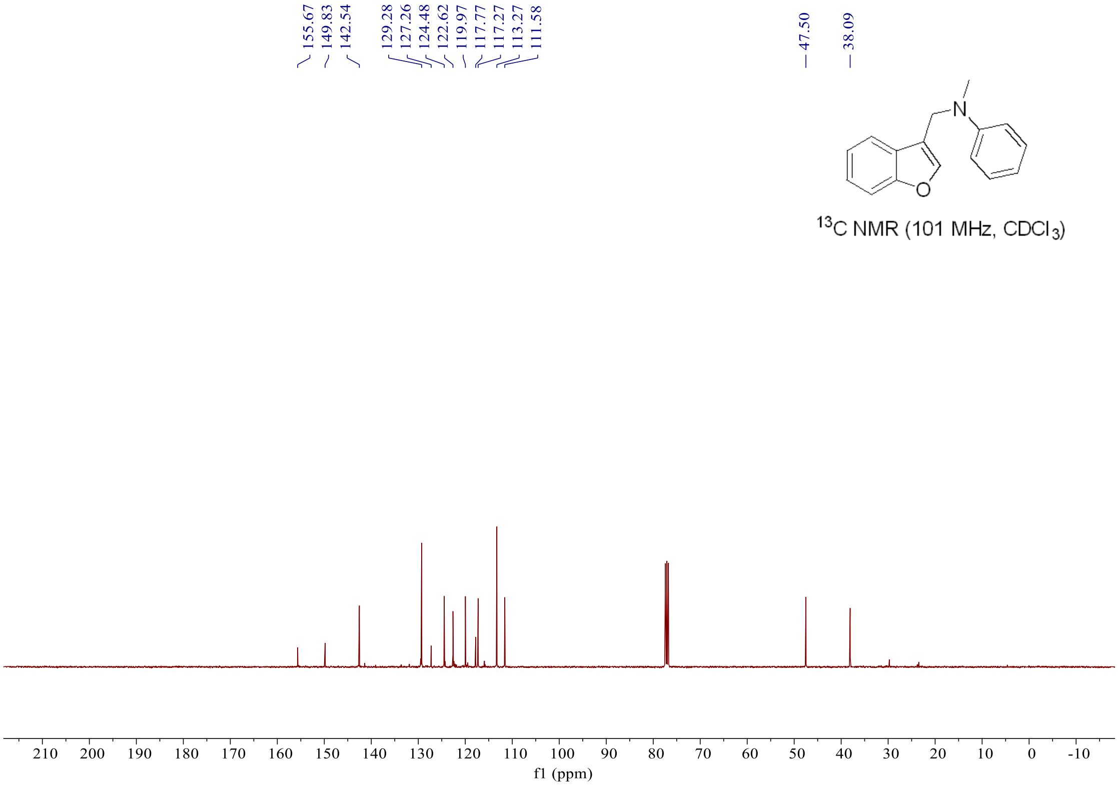

Supplementary Figure 81. ^{1}H, ^{13}C and ^{19}F NMR spectra of **7i.**

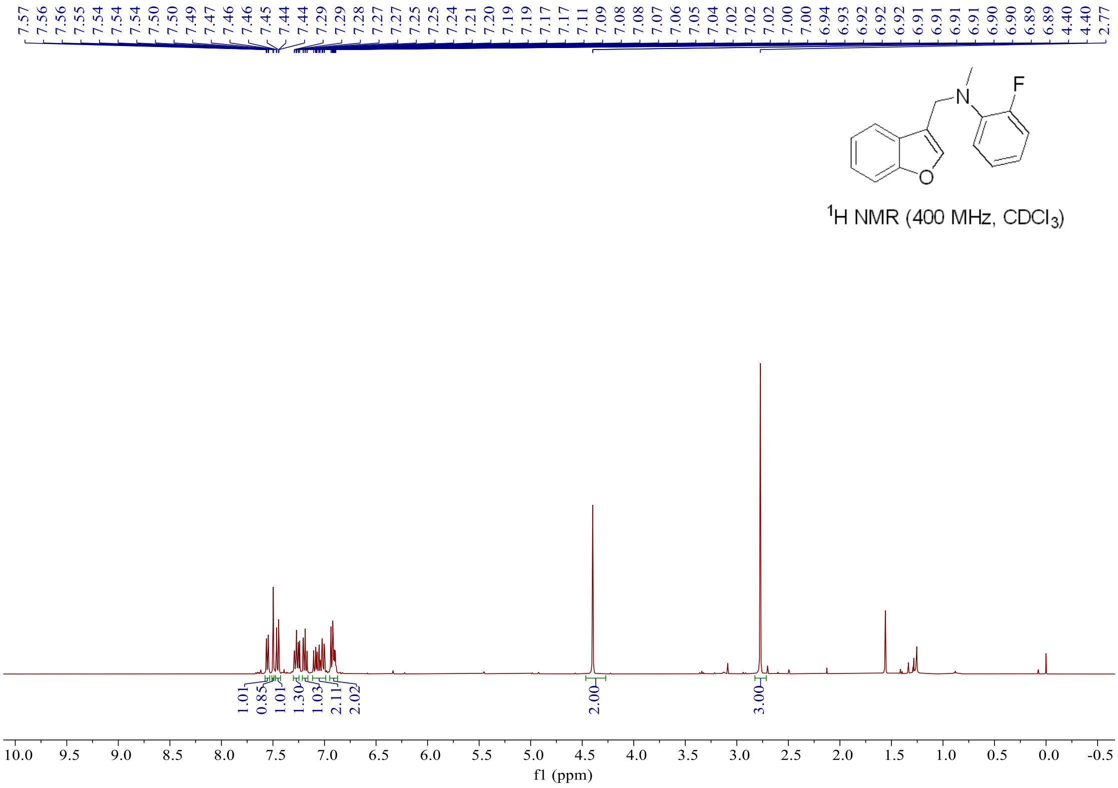

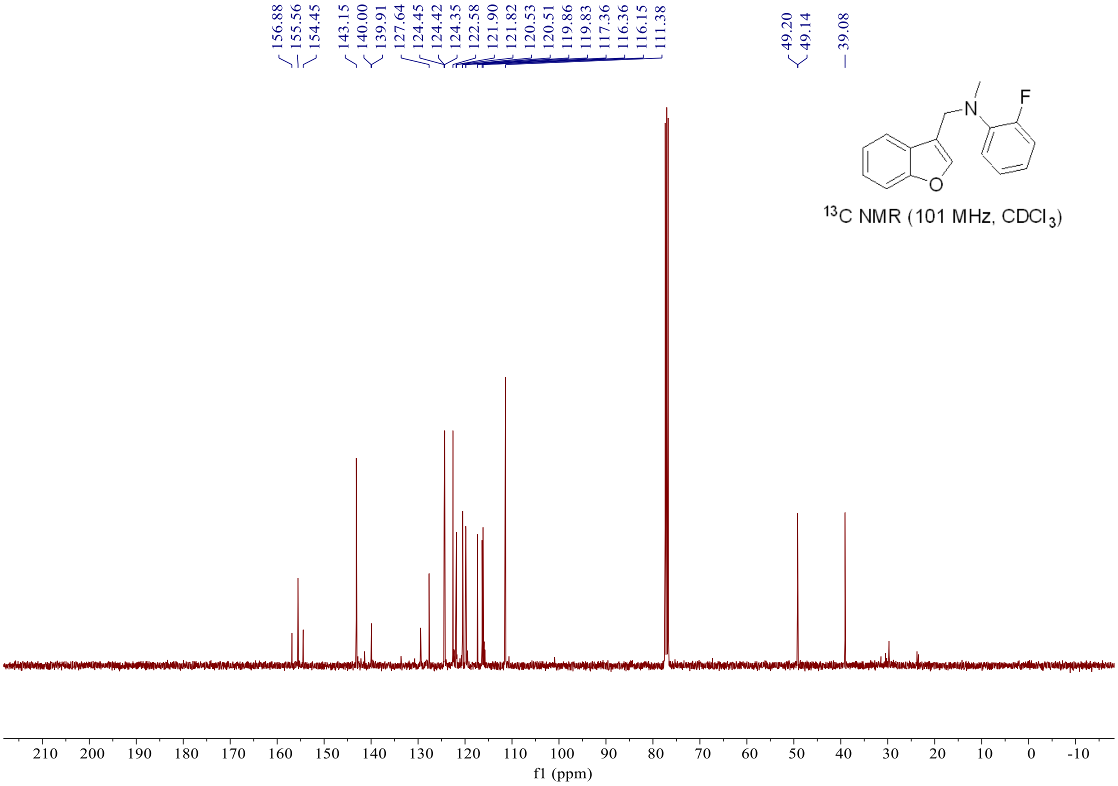

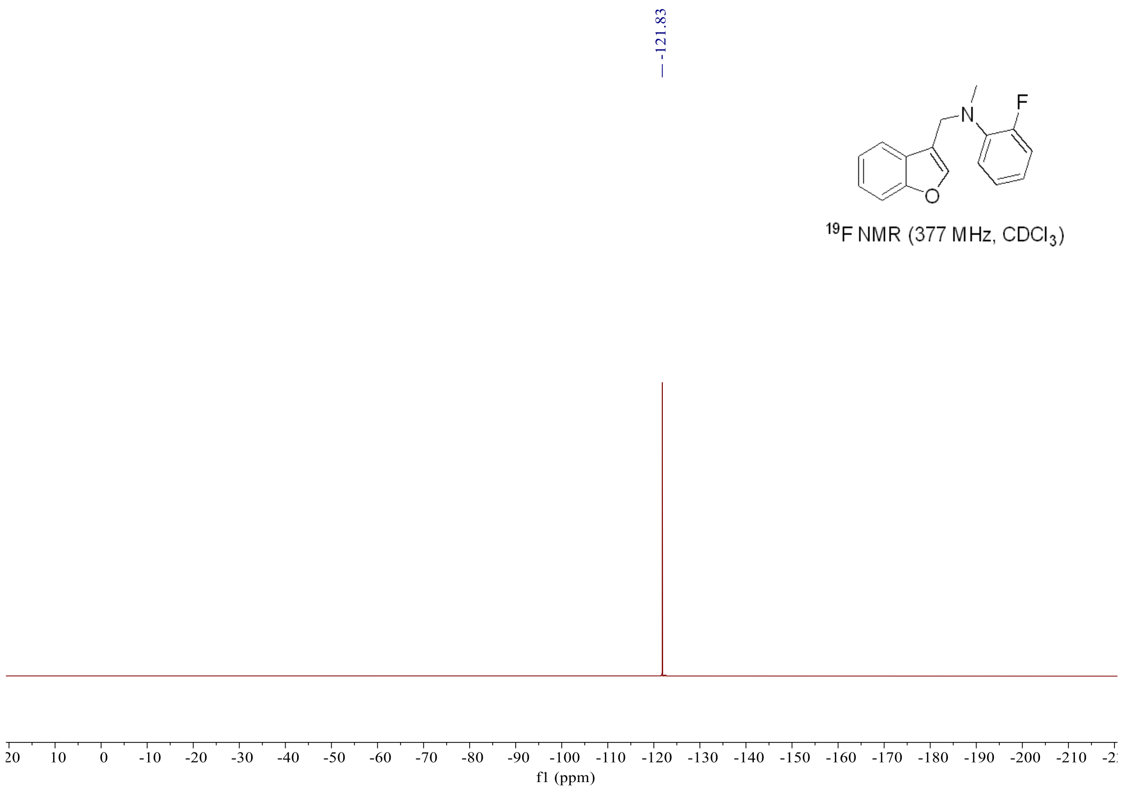
-121.83
F
N
O
19F NMR (377 MHz, CDCl3)
20 10 0 -10 -20 -30 -40 -50 -60 -70 -80 -90 -100 -110 -120 -130 -140 -150 -160 -170 -180 -190 -200 -210
f1 (ppm)

Supplementary Figure 82. ¹H and ¹³C NMR spectra of **7j.**

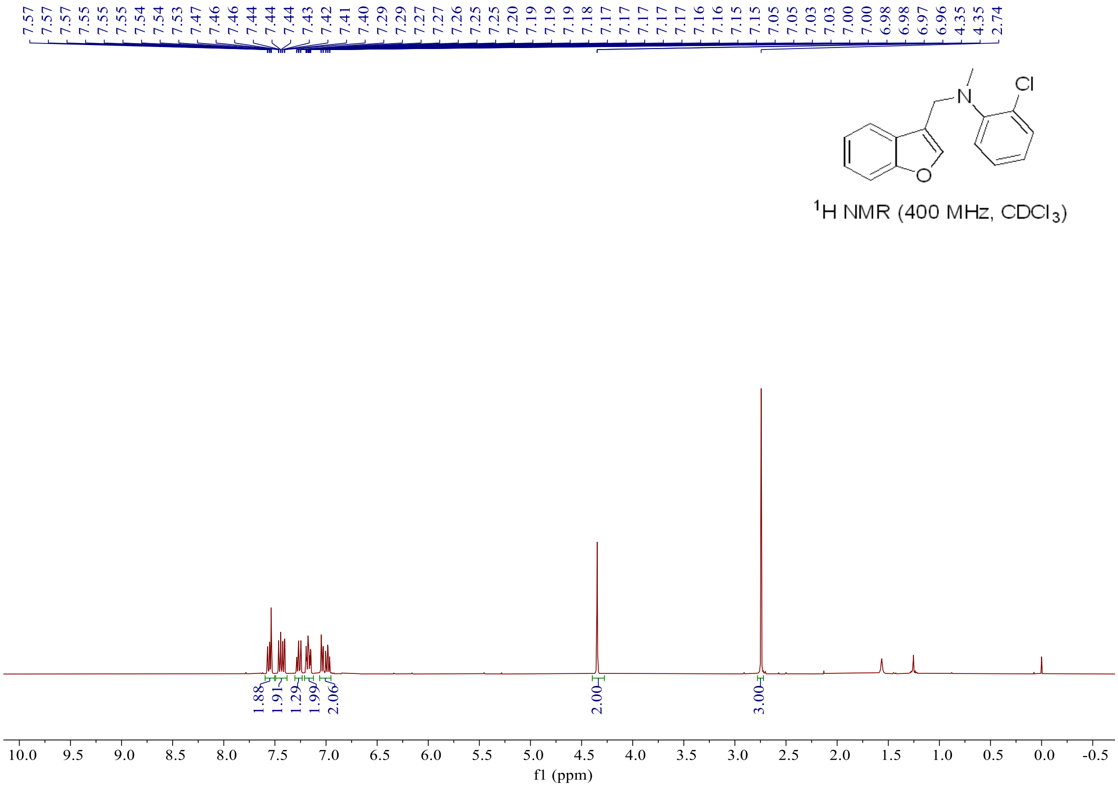

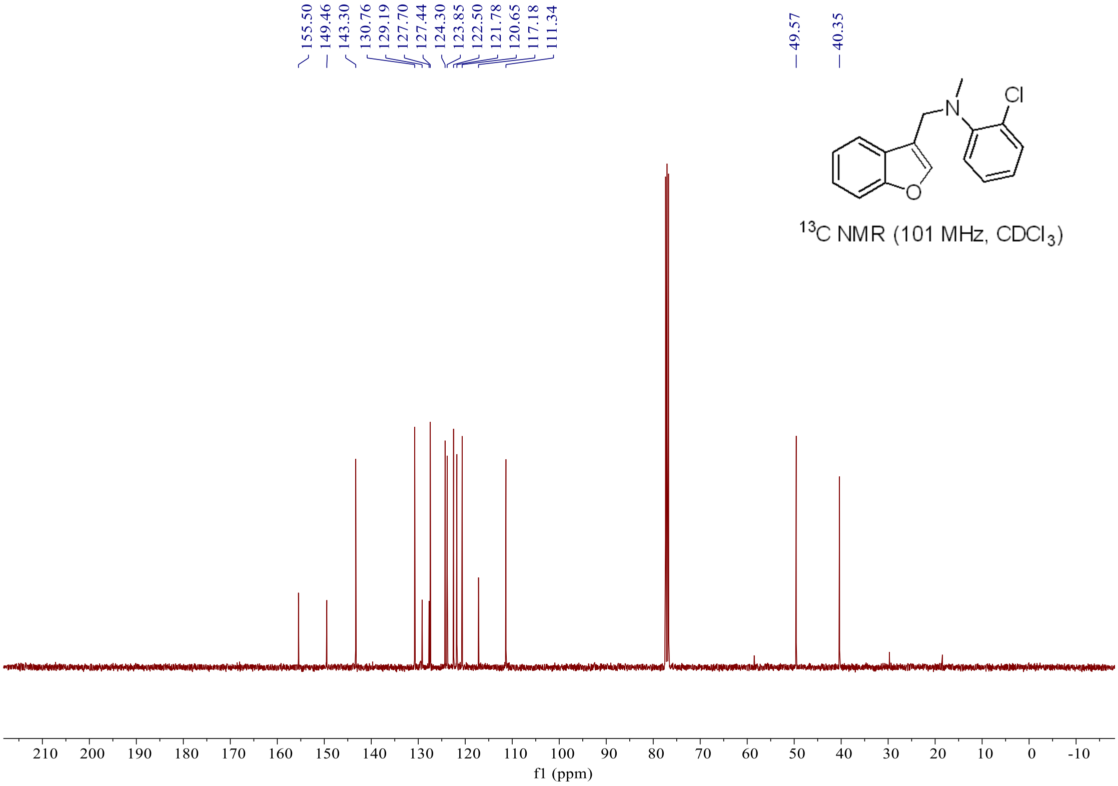

Supplementary Figure 83. ^{1}H and ^{13}C NMR spectra of **7k**.

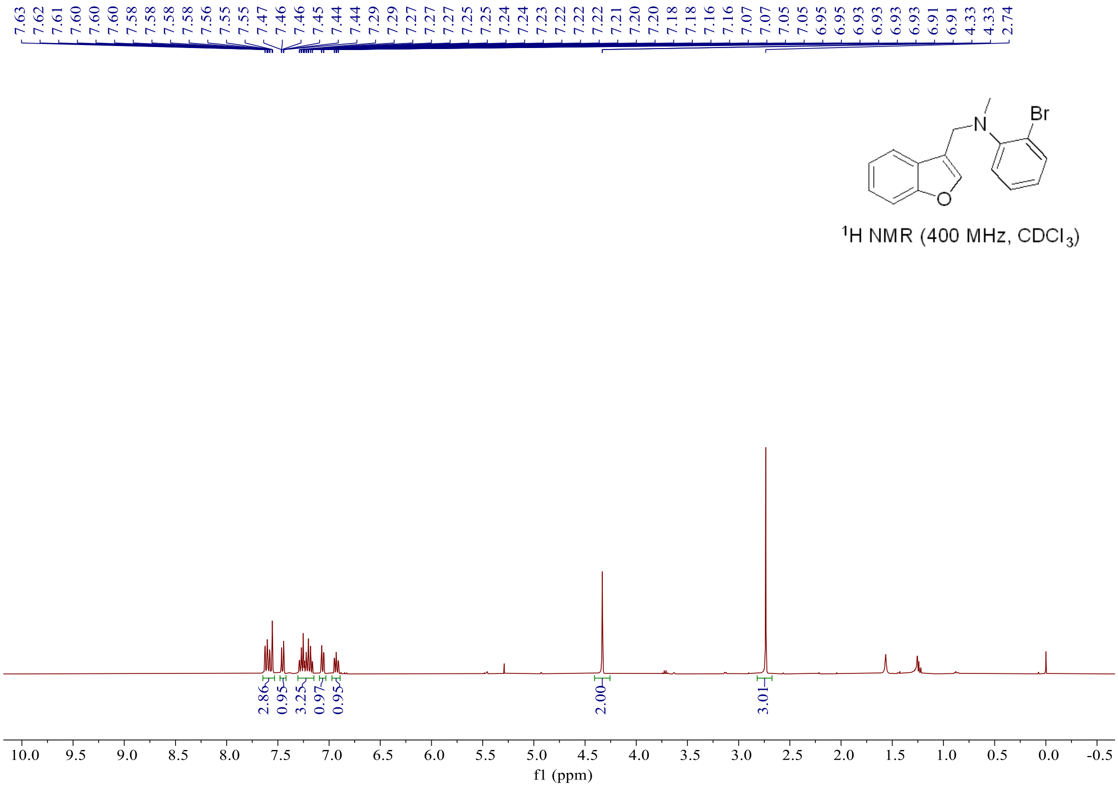

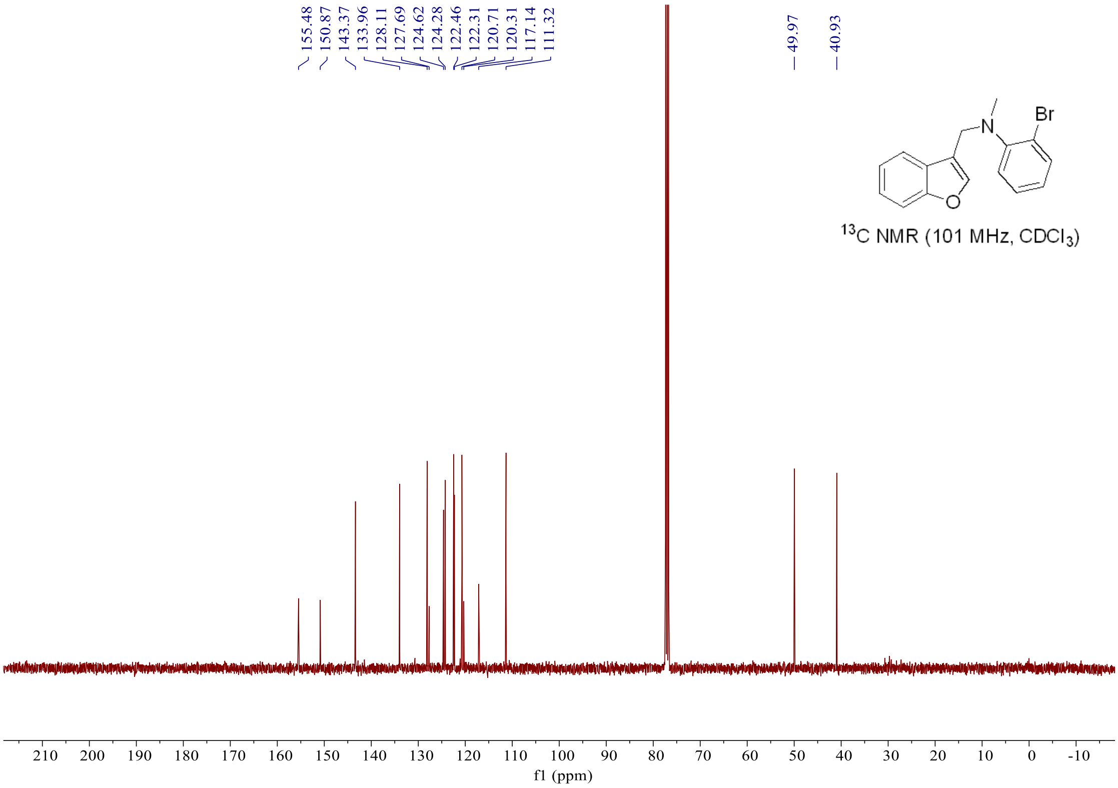

Supplementary Figure 84. ^{1}H and ^{13}C NMR spectra of **7l**.

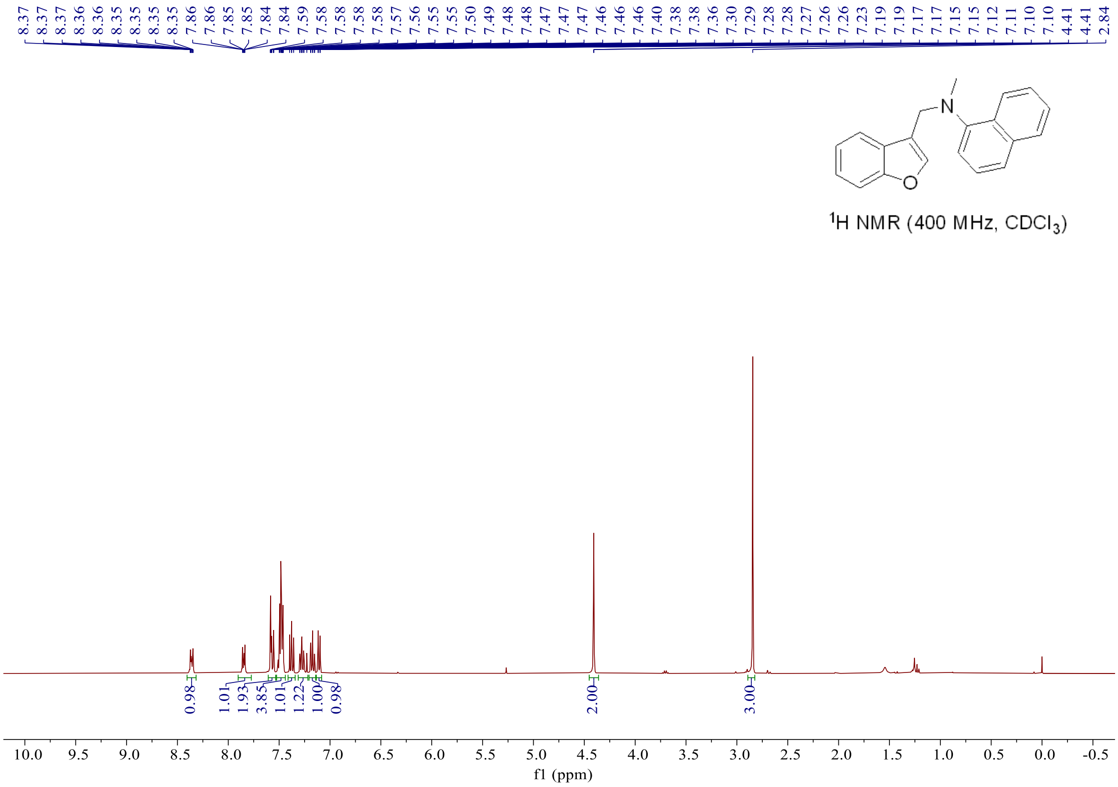

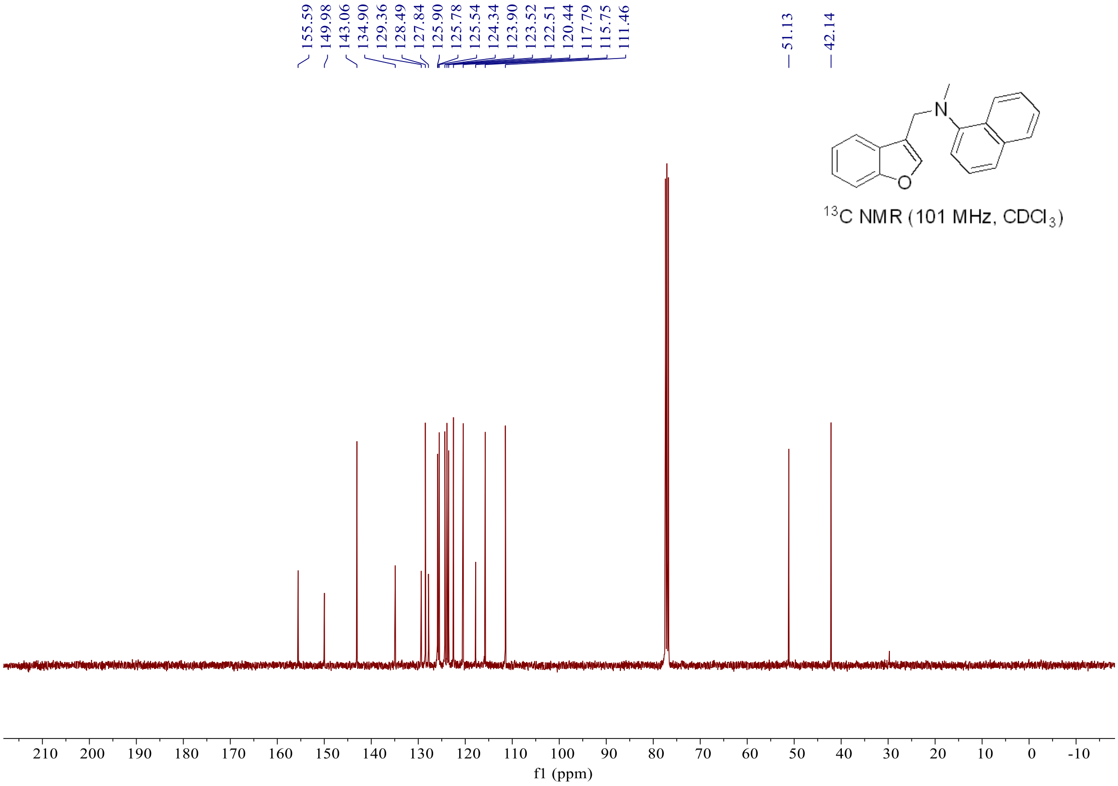

Supplementary Figure 85. ^{1}H and ^{13}C NMR spectra of **7m.**

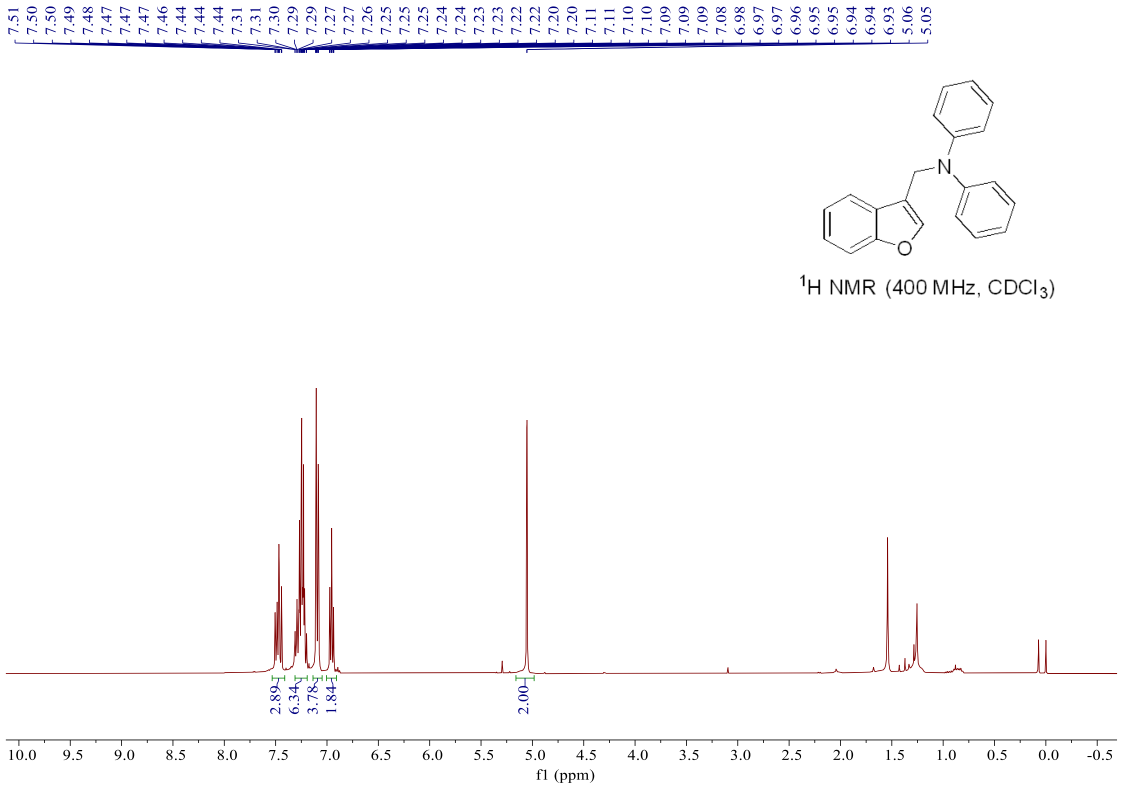

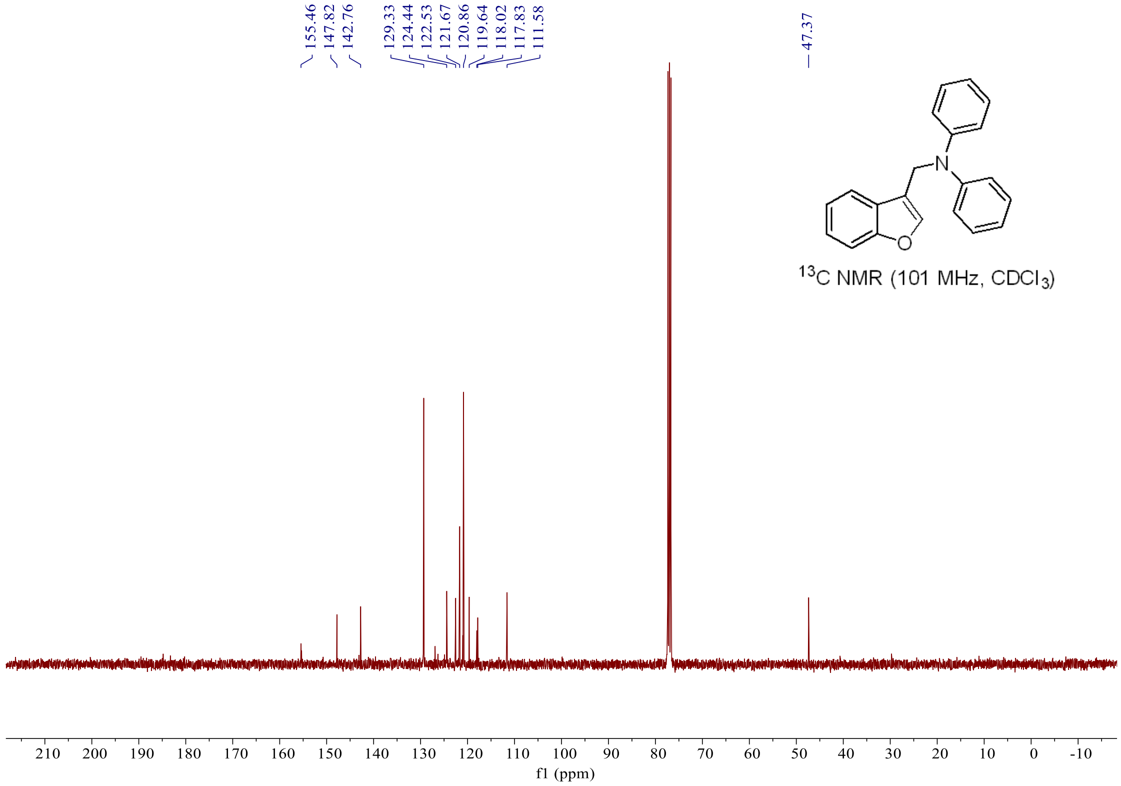

Supplementary Figure 86. ^{1}H, ^{13}C and ^{31}P NMR spectra of **7a'**.

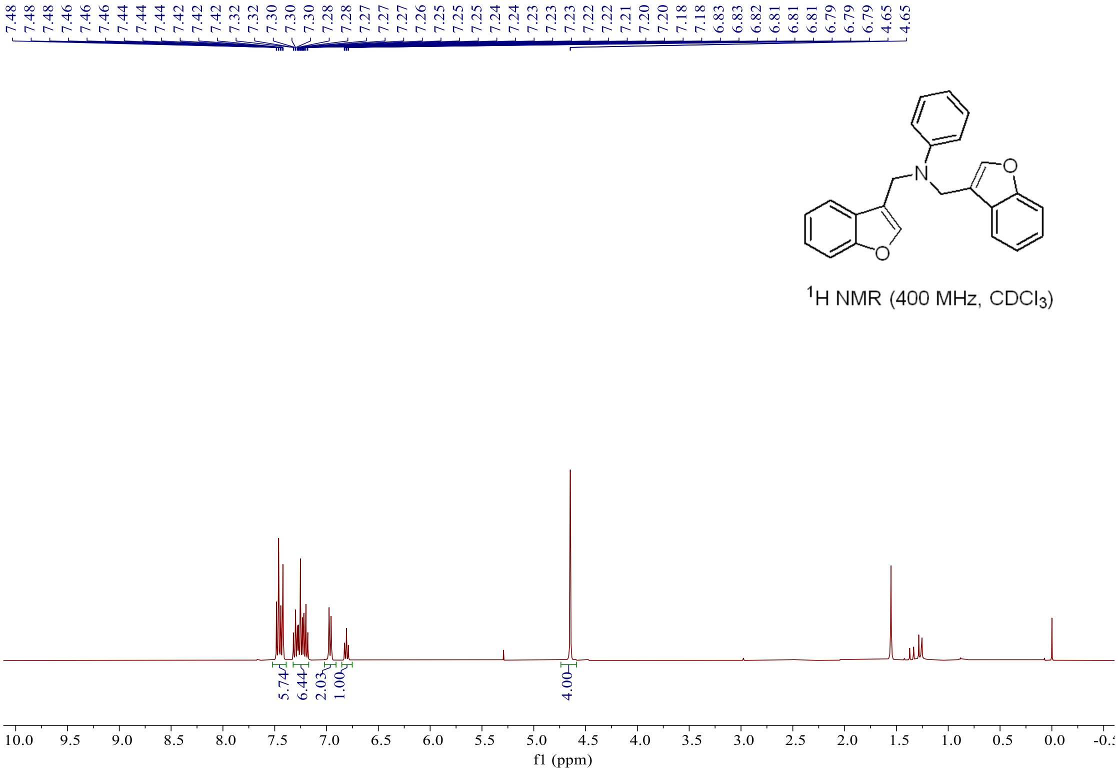

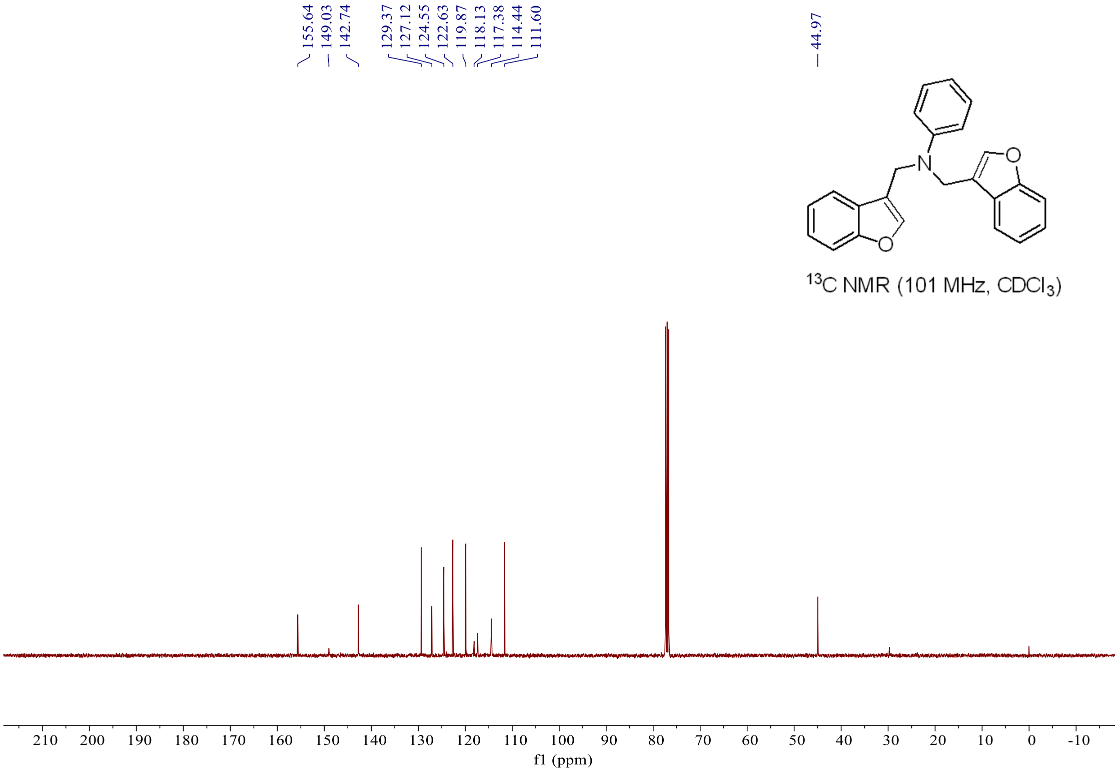

Supplementary Figure 87. ^{1}H, ^{13}C and ^{31}P NMR spectra of **8.**

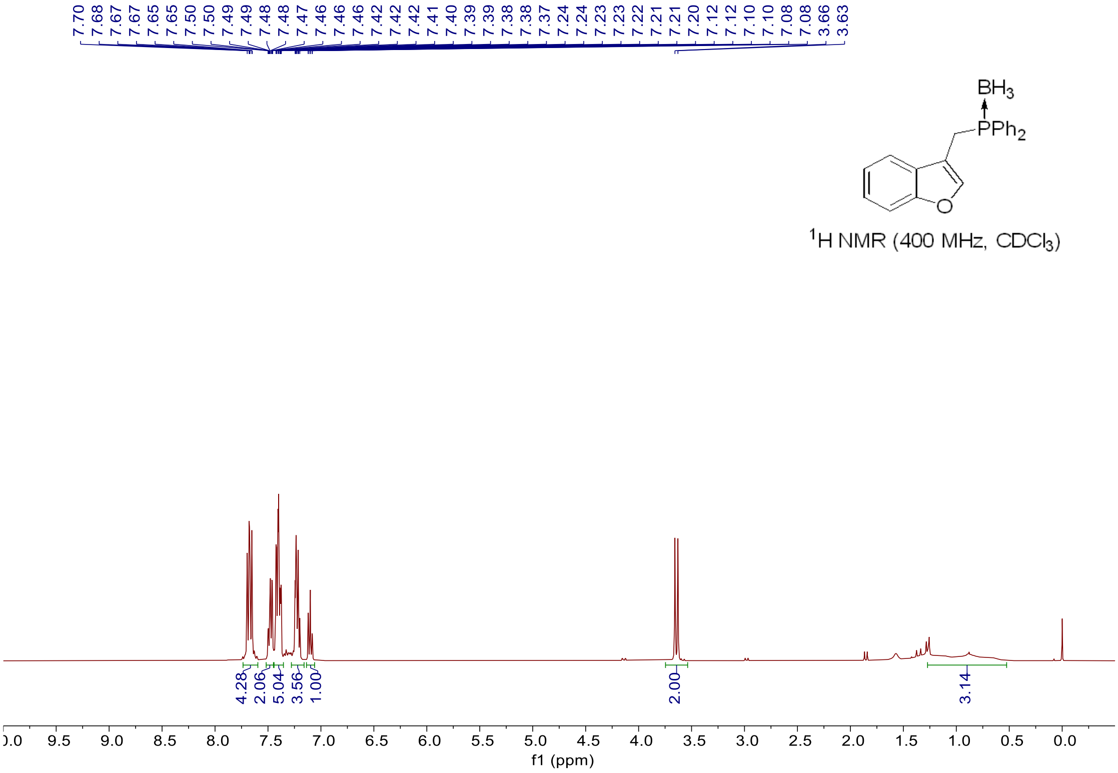

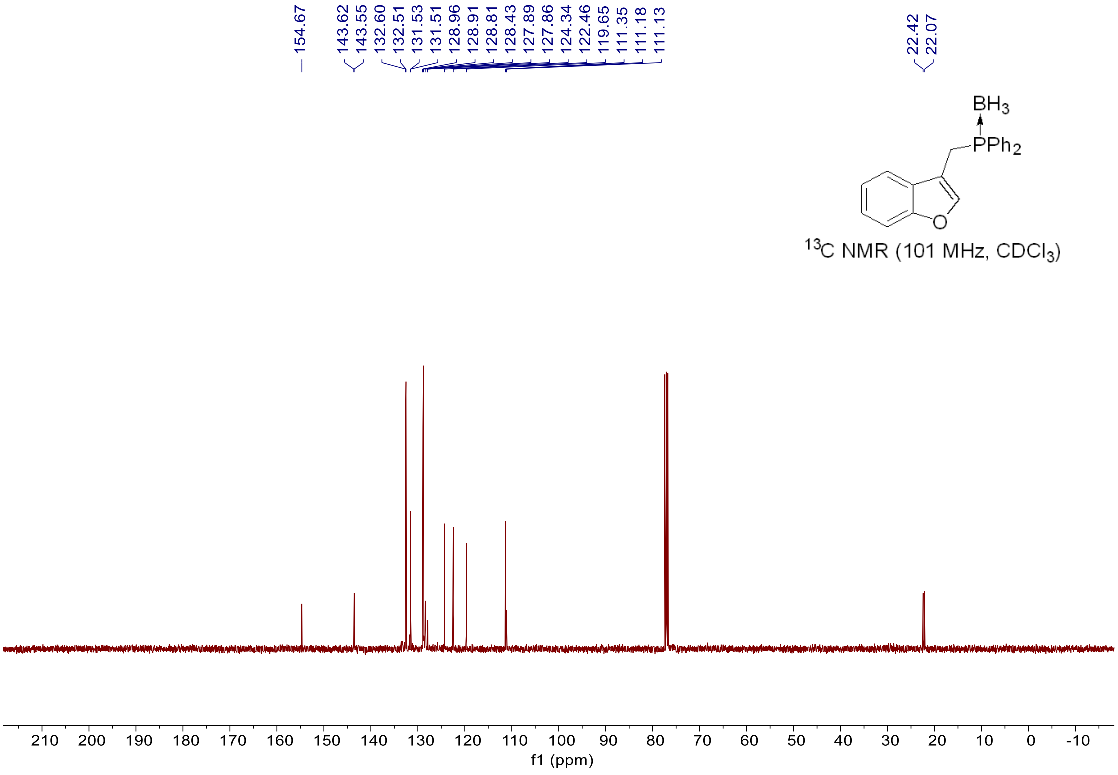

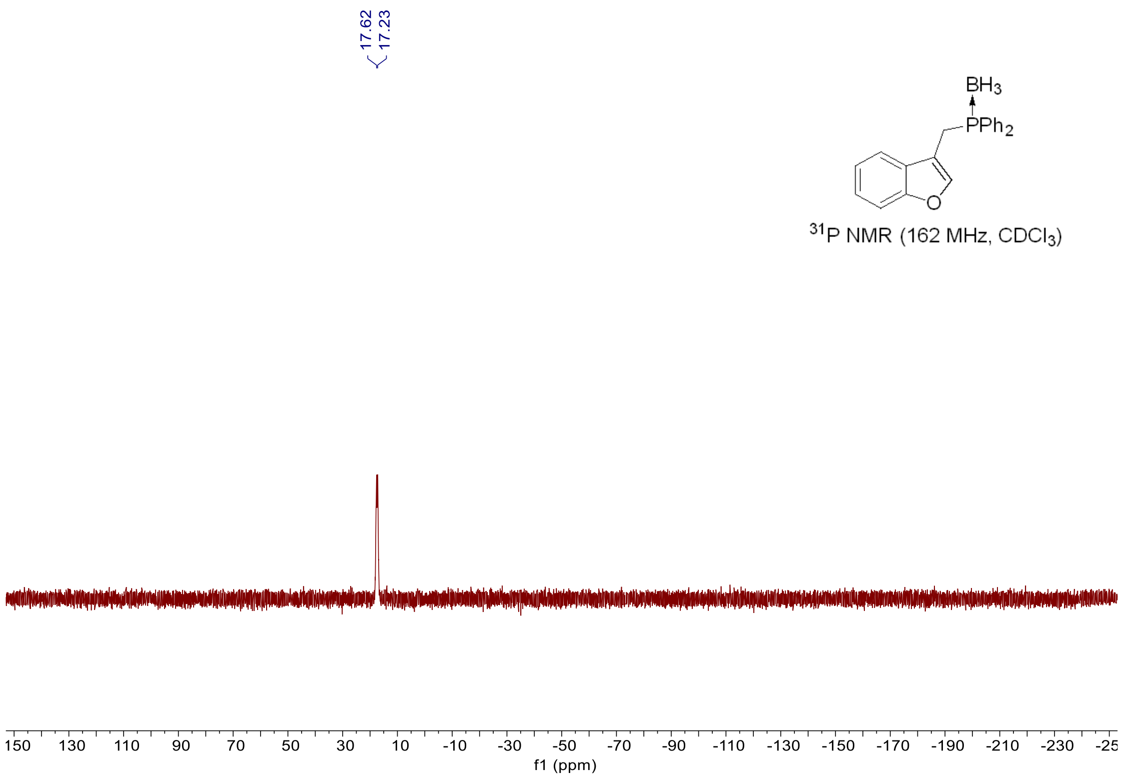
17.62
17.23
BH3
PPh2
O
31P NMR (162 MHz, CDCl3)
150 130 110 90 70 50 30 10 -10 -30 -50 -70 -90 -110 -130 -150 -170 -190 -210 -230 -25
f1 (ppm)

Supplementary Figure 88. ^{1}H, and ^{13}C NMR spectra of **S12**.

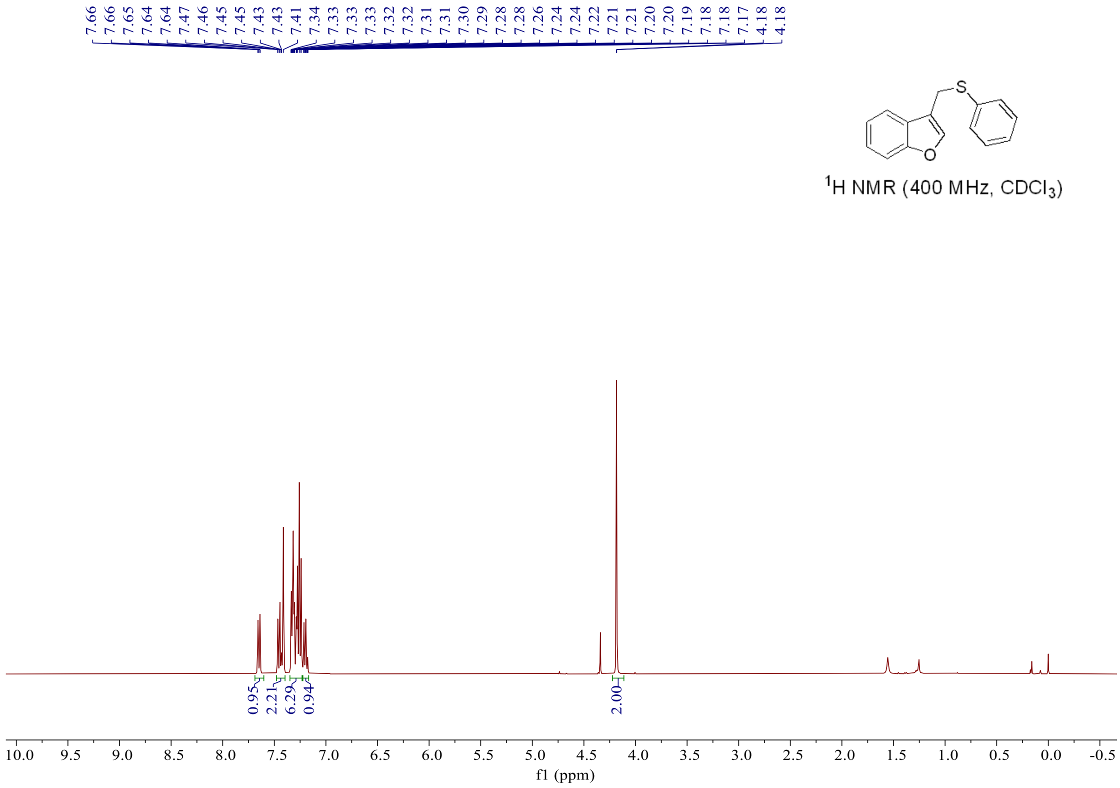

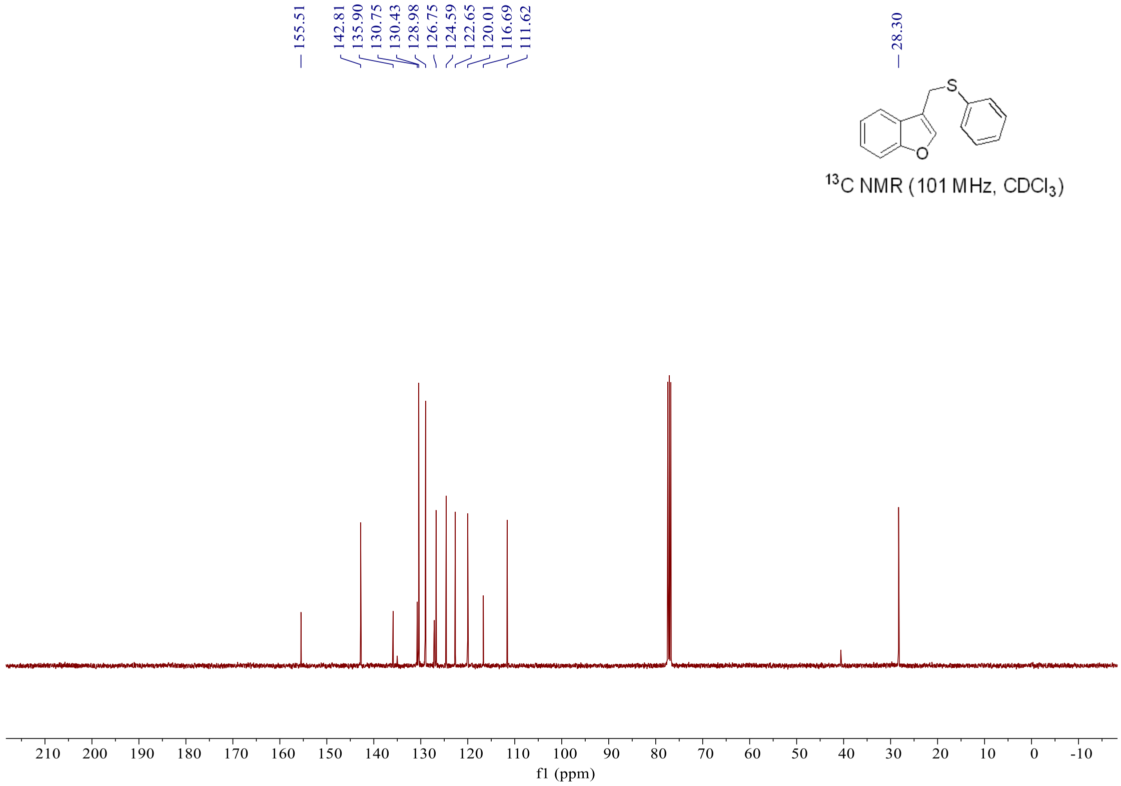

Supplementary Figure 89. ^{1}H, ^{13}C, ^{19}F and ^{31}P NMR spectra of **13**.

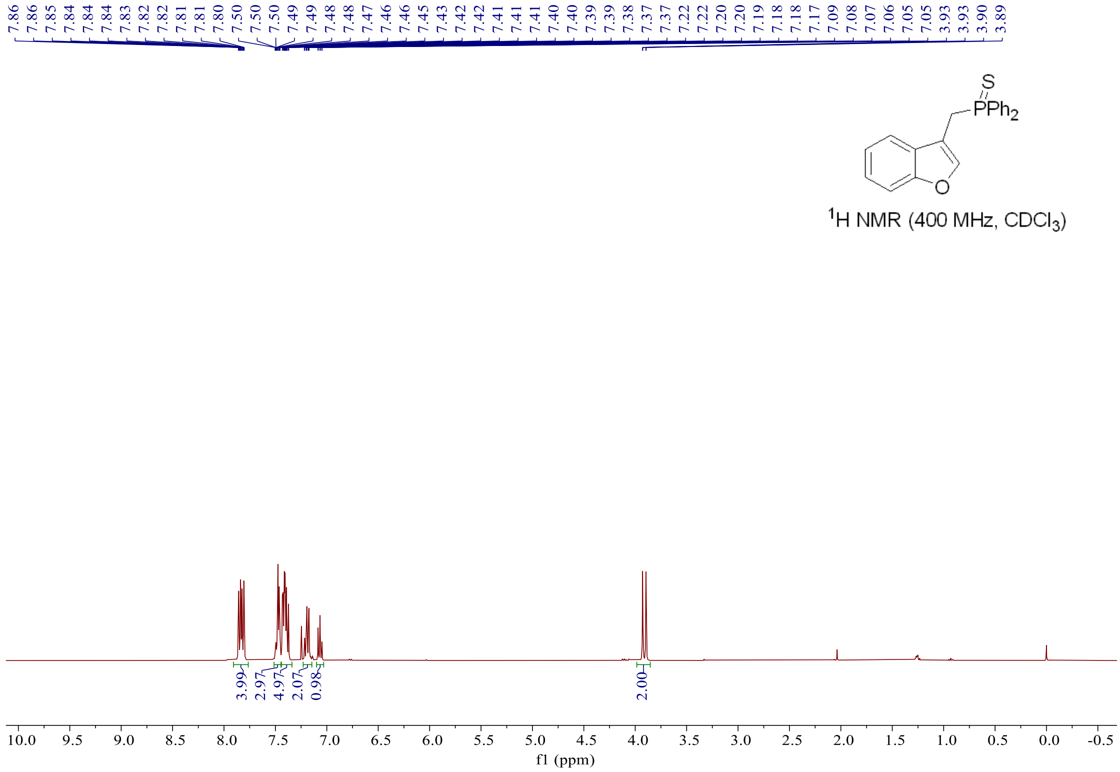

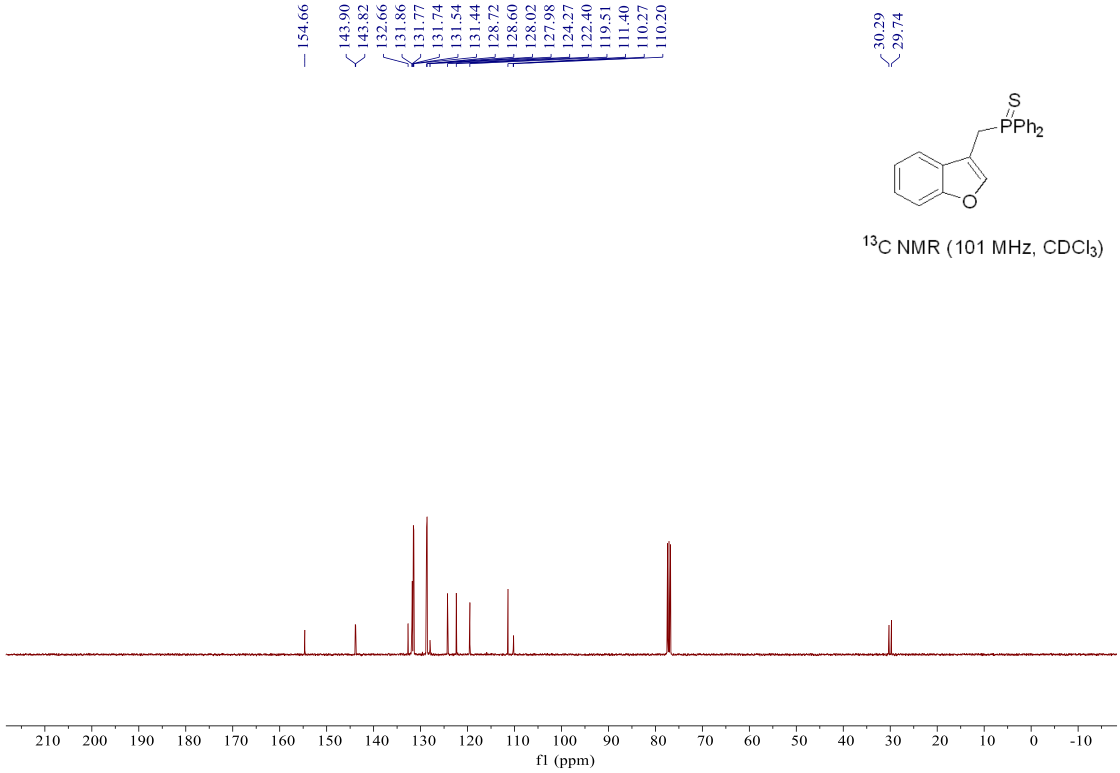

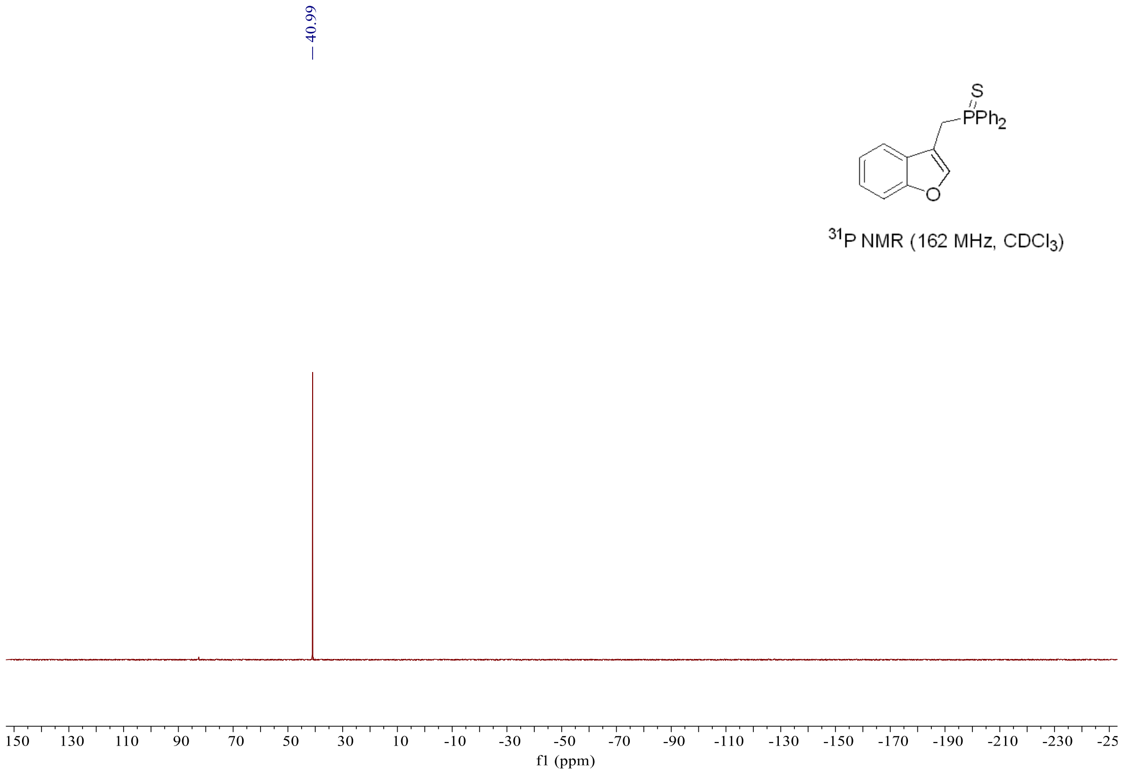

^{31}P NMR (162 MHz, $CDCl_3$)

Supplementary Figure 90. ^{1}H, ^{13}C, ^{19}F and ^{31}P NMR spectra of **14**.

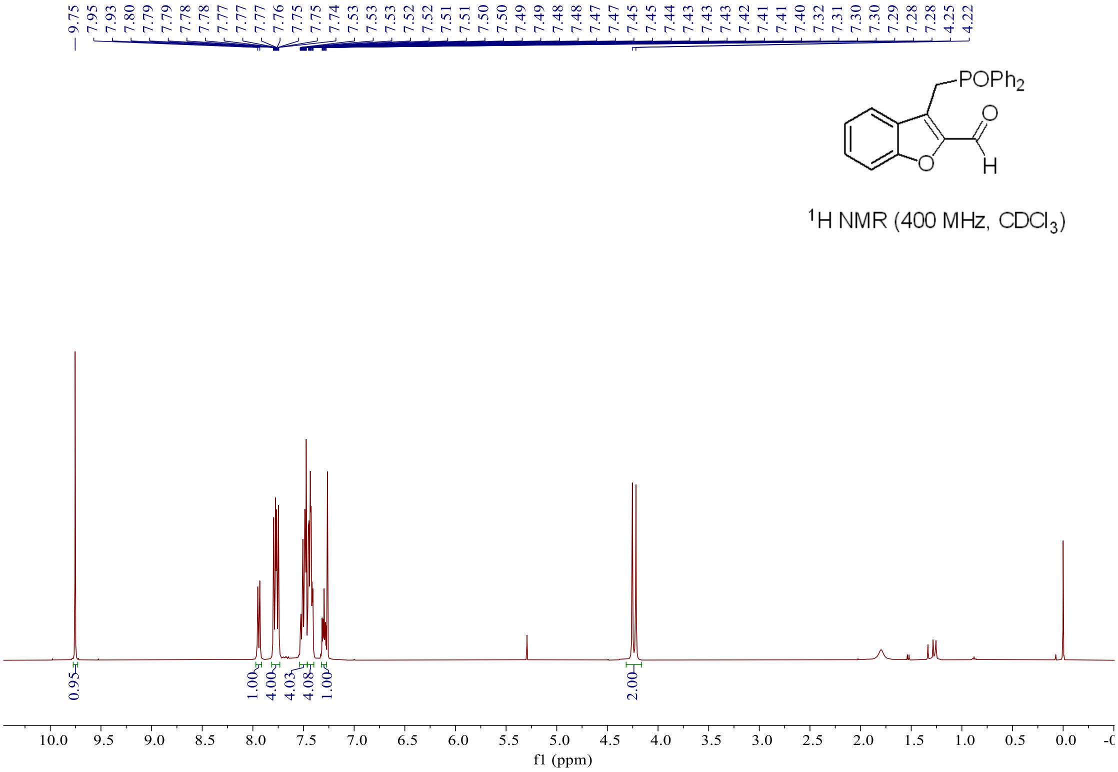

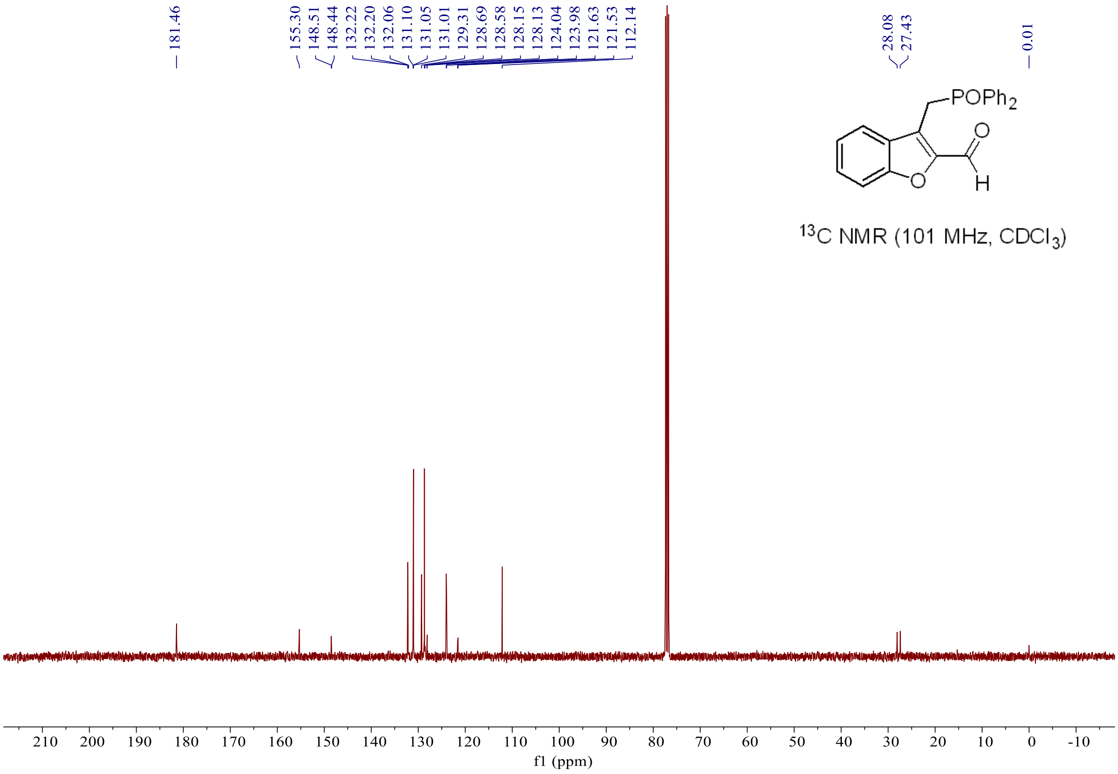

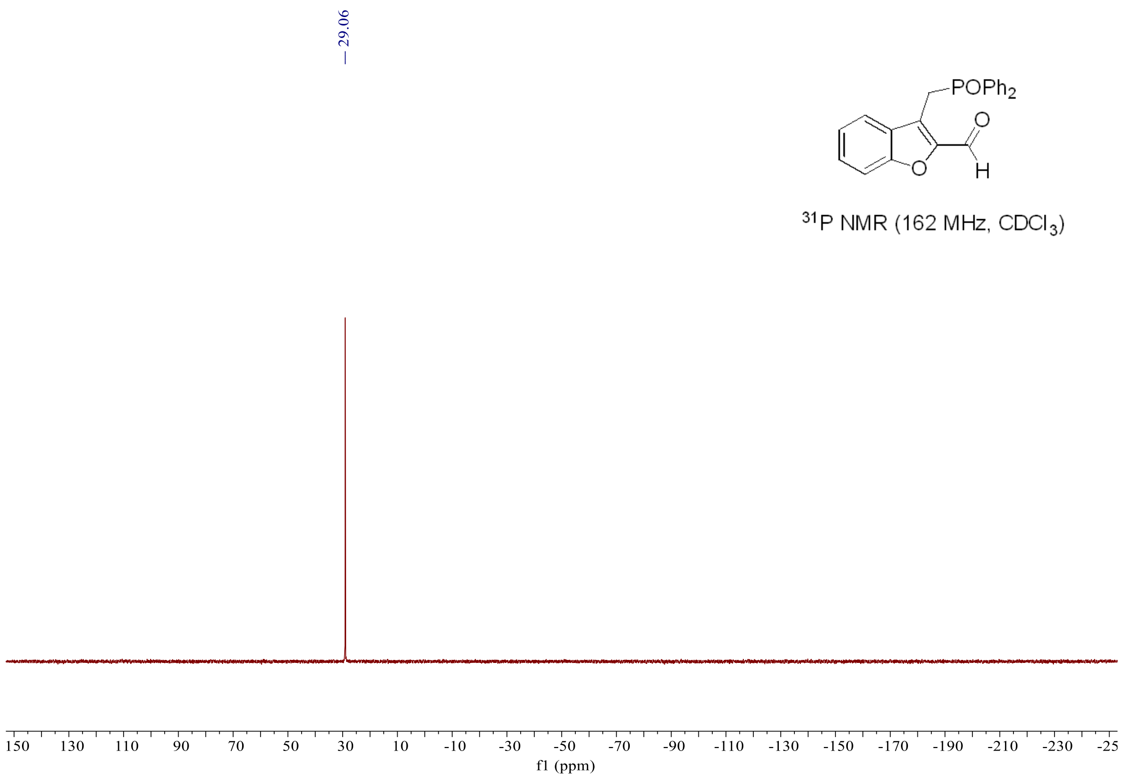
29.06
POPh2
O
H
O
150
130
110
90
70
50
30
10
-10
-30
-50
-70
-90
-110
-130
-150
-170
-190
-210
-230
-25
f1 (ppm)

^{31}P NMR (162 MHz, $CDCl_3$)

Supplementary Figure 91. ^{1}H, ^{13}C, ^{19}F and ^{31}P NMR spectra of **15.**

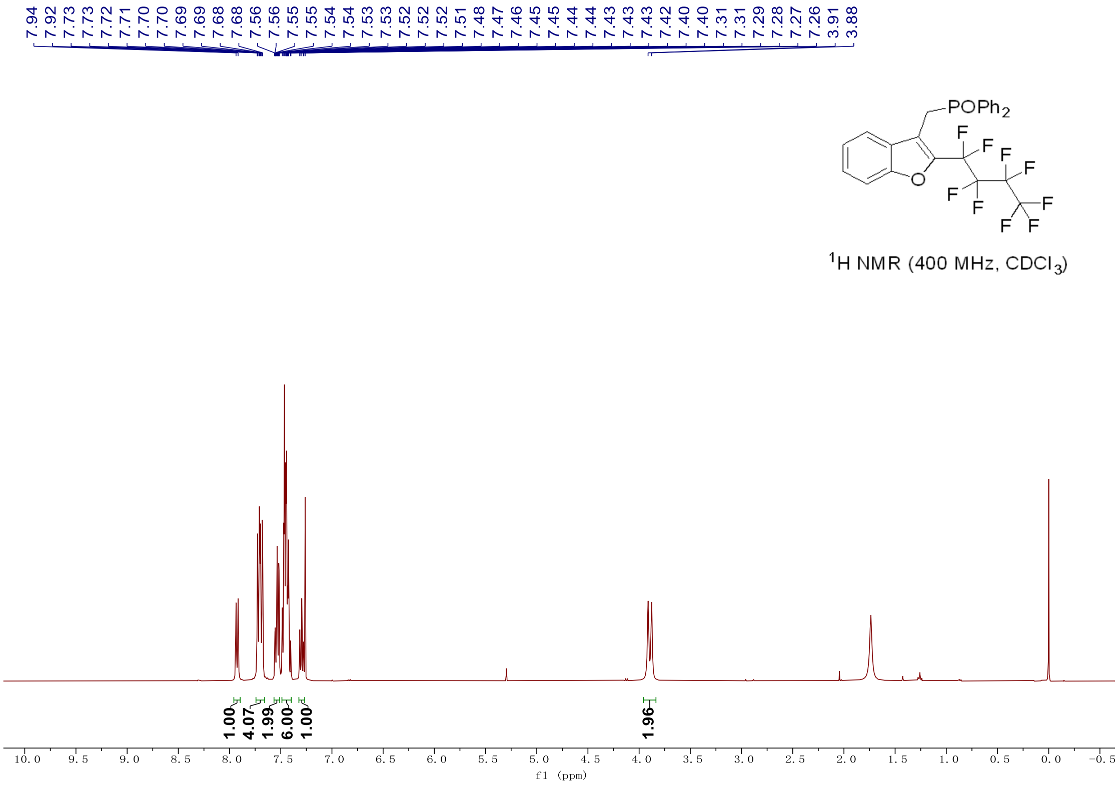

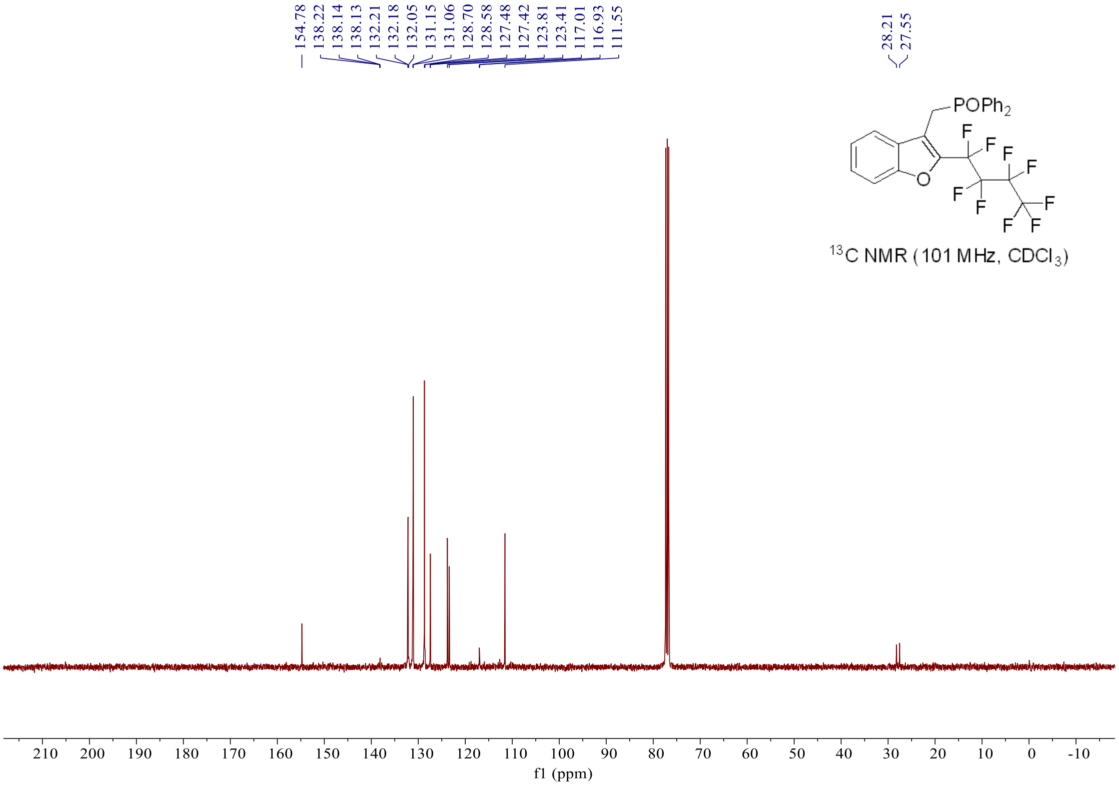

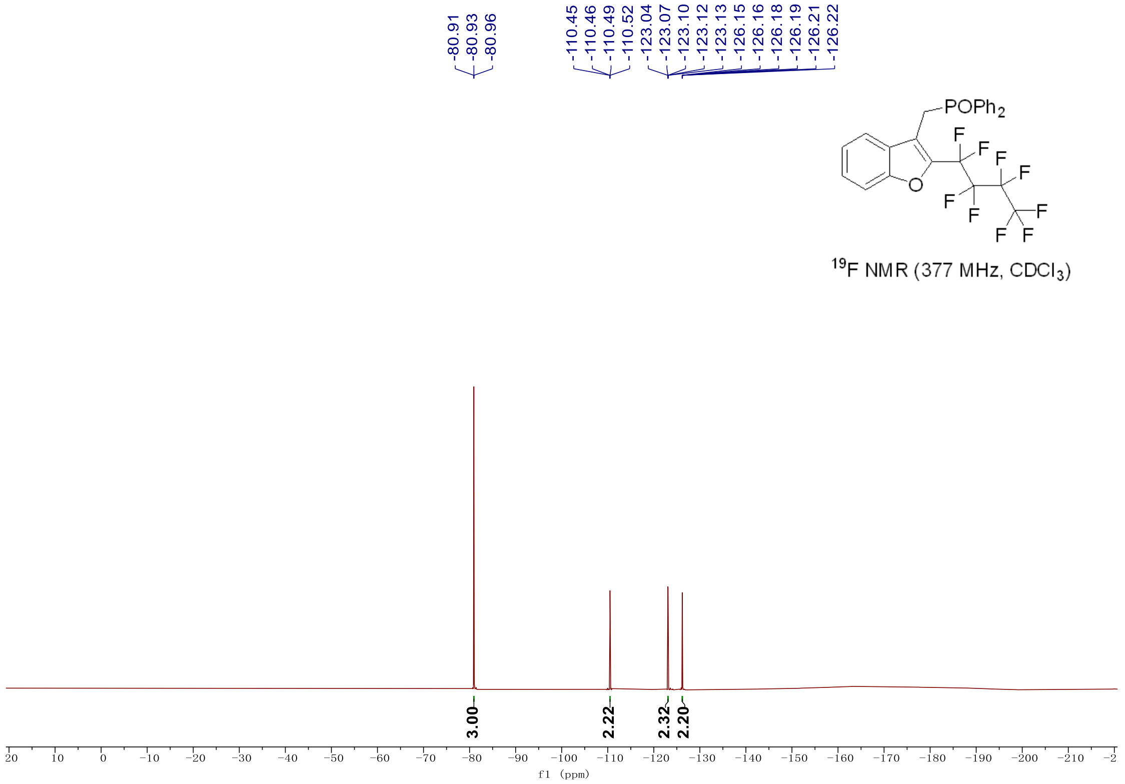
-80.91
-80.93
-80.96
-110.45
-110.46
-110.49
-110.52
-123.04
-123.07
-123.10
-123.12
-123.13
-126.15
-126.16
-126.18
-126.19
-126.21
-126.22
POPh2
F
O
^{19}F NMR (377 MHz, $CDCl_3$)
3.00
2.22
2.32
2.20
f1 (ppm)

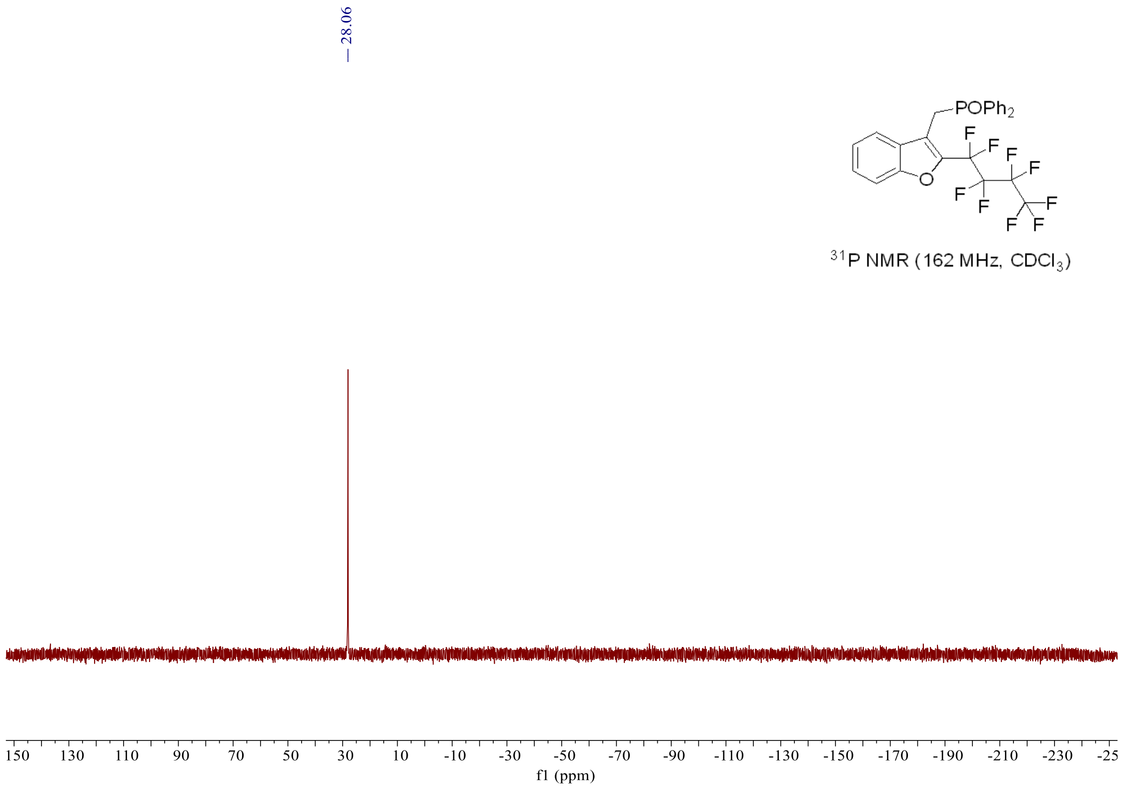
28.06
POPh2
F
O
^{31}P NMR (162 MHz, $CDCl_3$)
f1 (ppm)

Supplementary Figure 92. ^{1}H, ^{13}C, ^{19}F and ^{31}P NMR spectra of **16.**

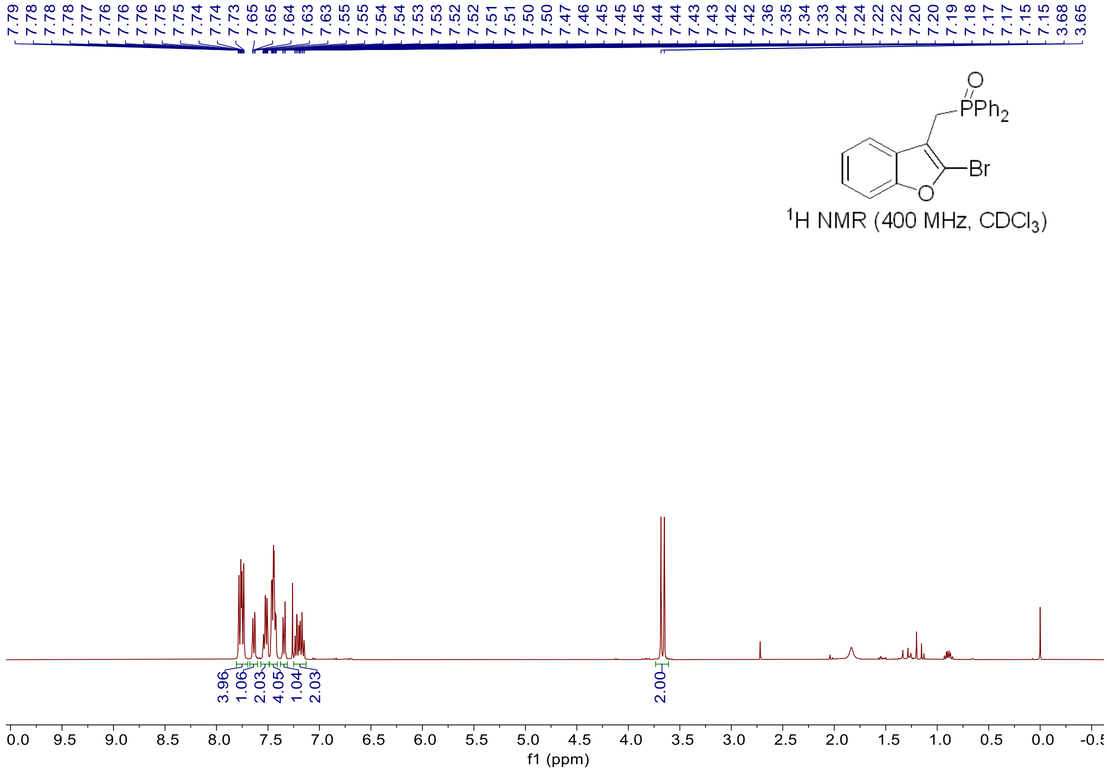

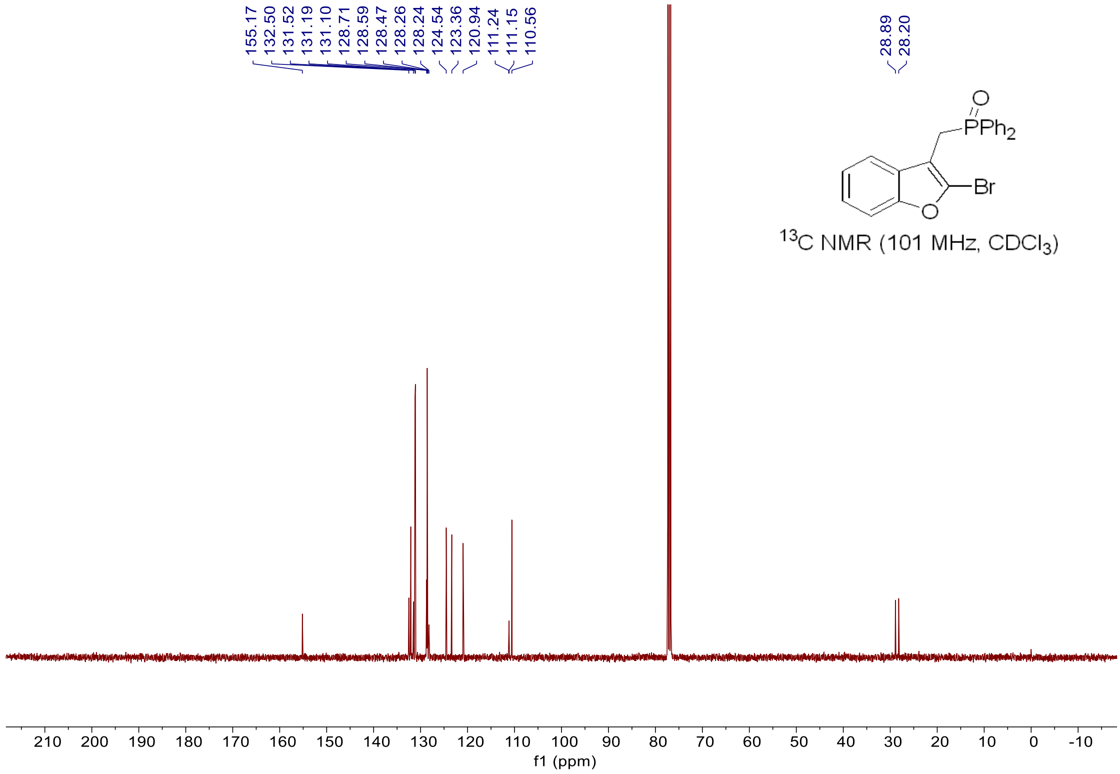

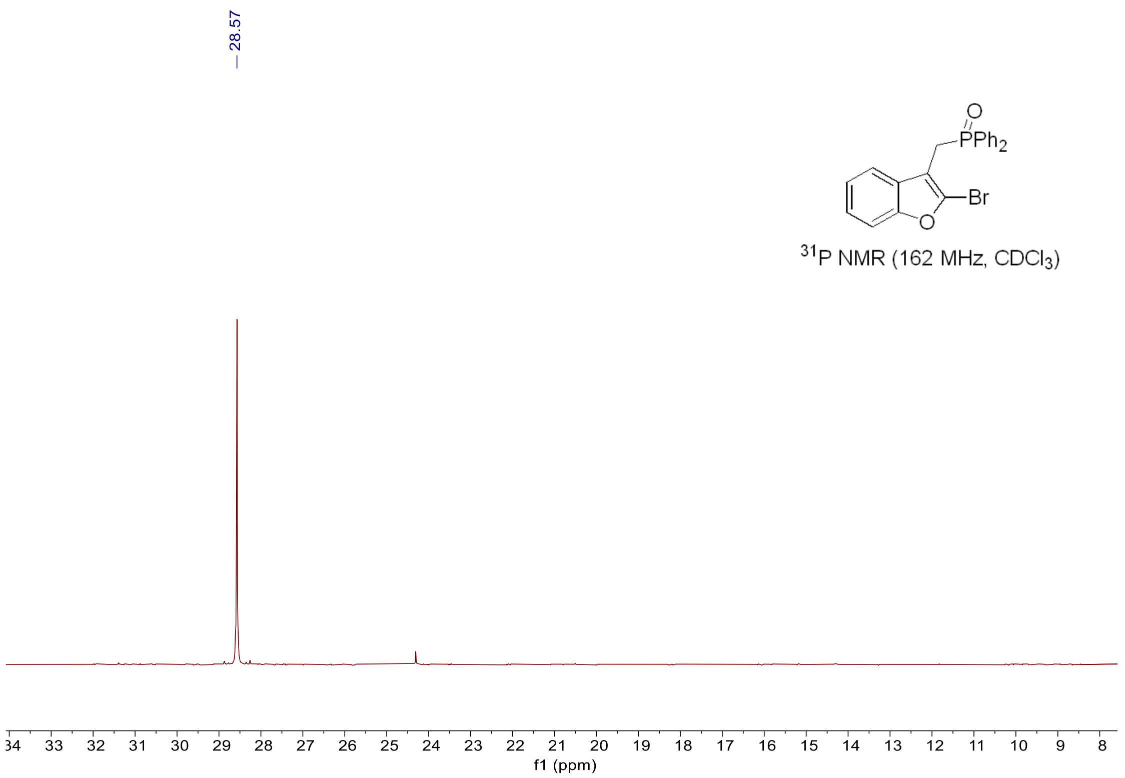

28.57
PPh2
Br
O
31P NMR (162 MHz, CDCl3)
34 33 32 31 30 29 28 27 26 25 24 23 22 21 20 19 18 17 16 15 14 13 12 11 10 9 8
f1 (ppm)

Supplementary Figure 93. ^{1}H, ^{13}C, ^{19}F and ^{31}P NMR spectra of **17**.

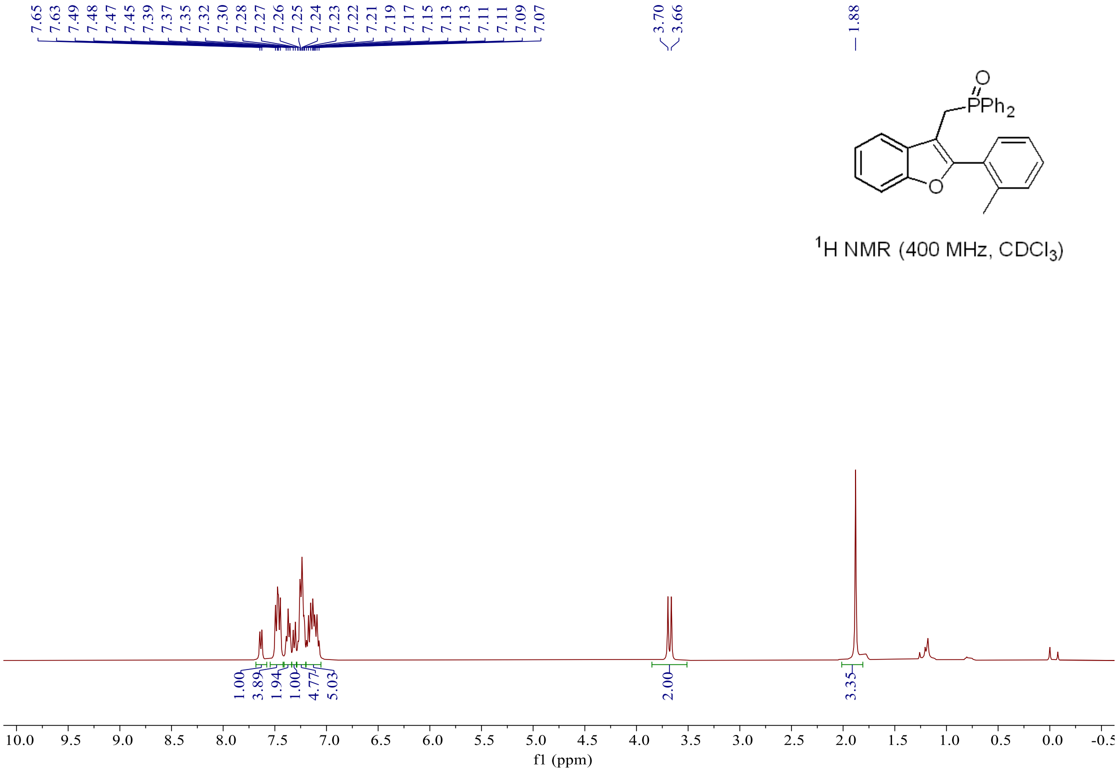

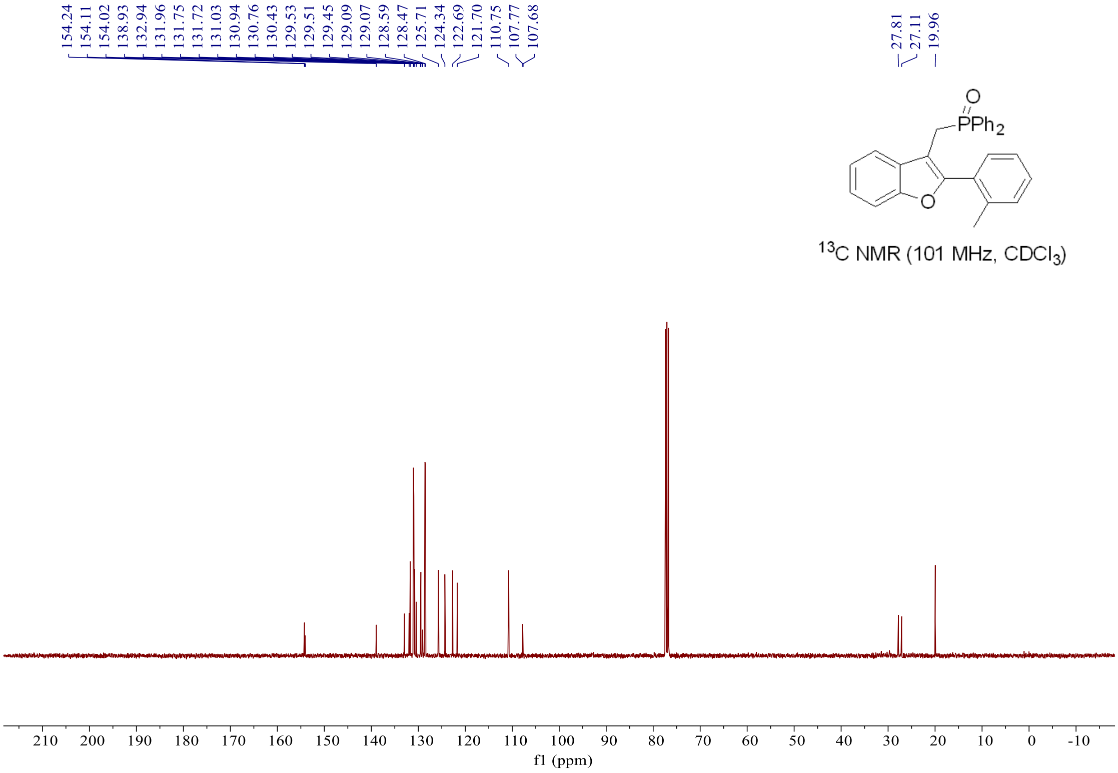

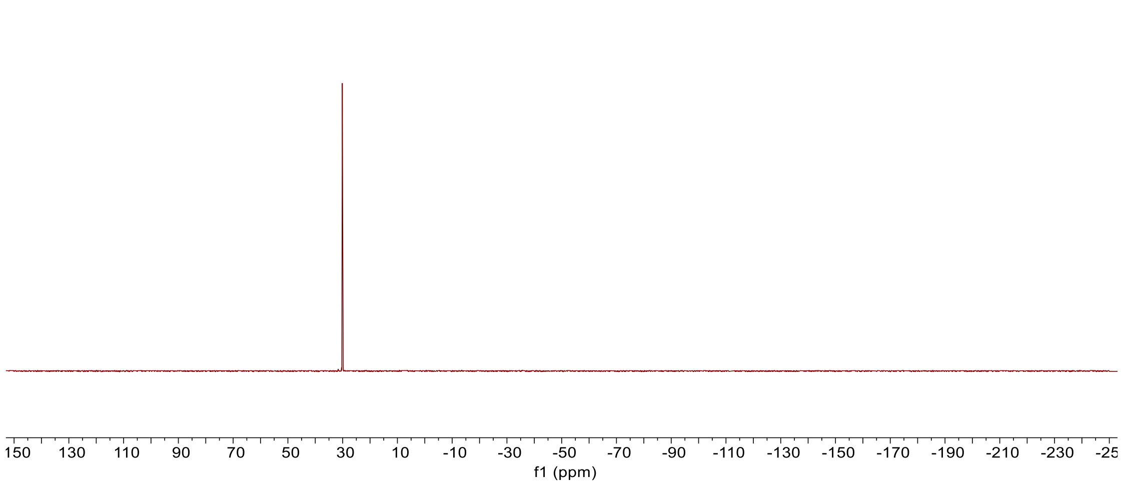

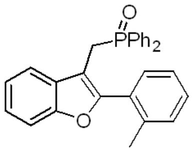

^{31}P NMR (162 MHz, $CDCl_3$)

Supplementary Figure 94. ^{1}H, ^{13}C, ^{19}F and ^{31}P NMR spectra of **18.**

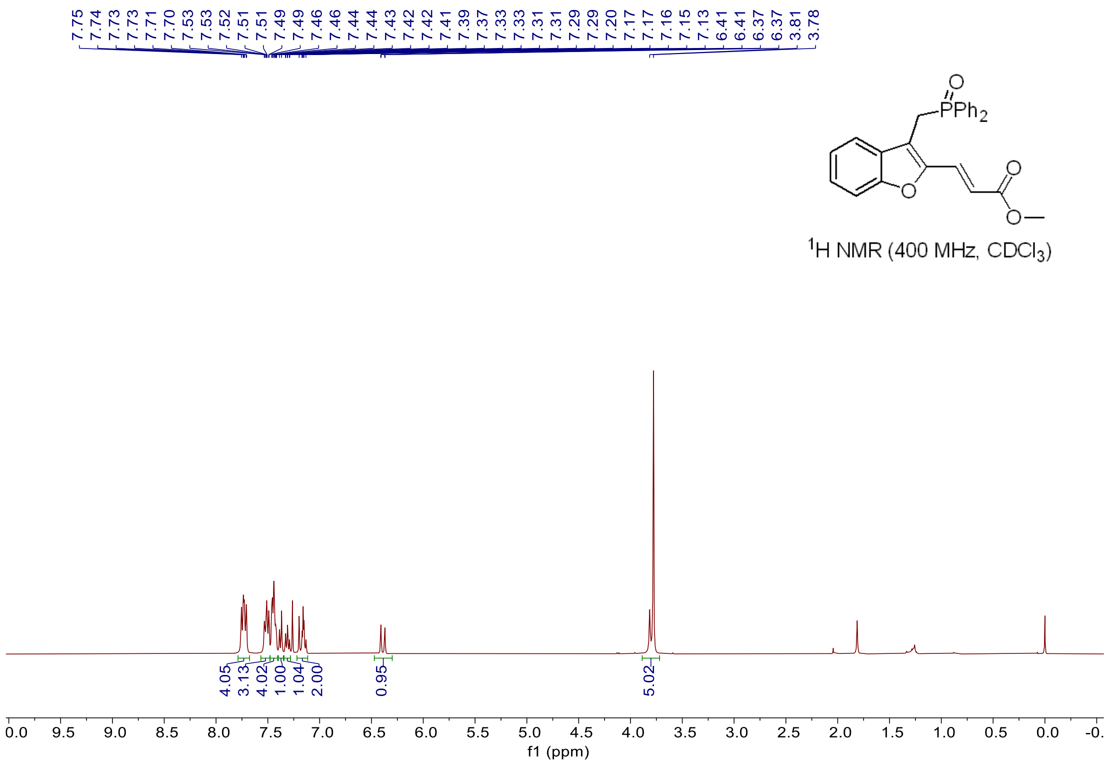

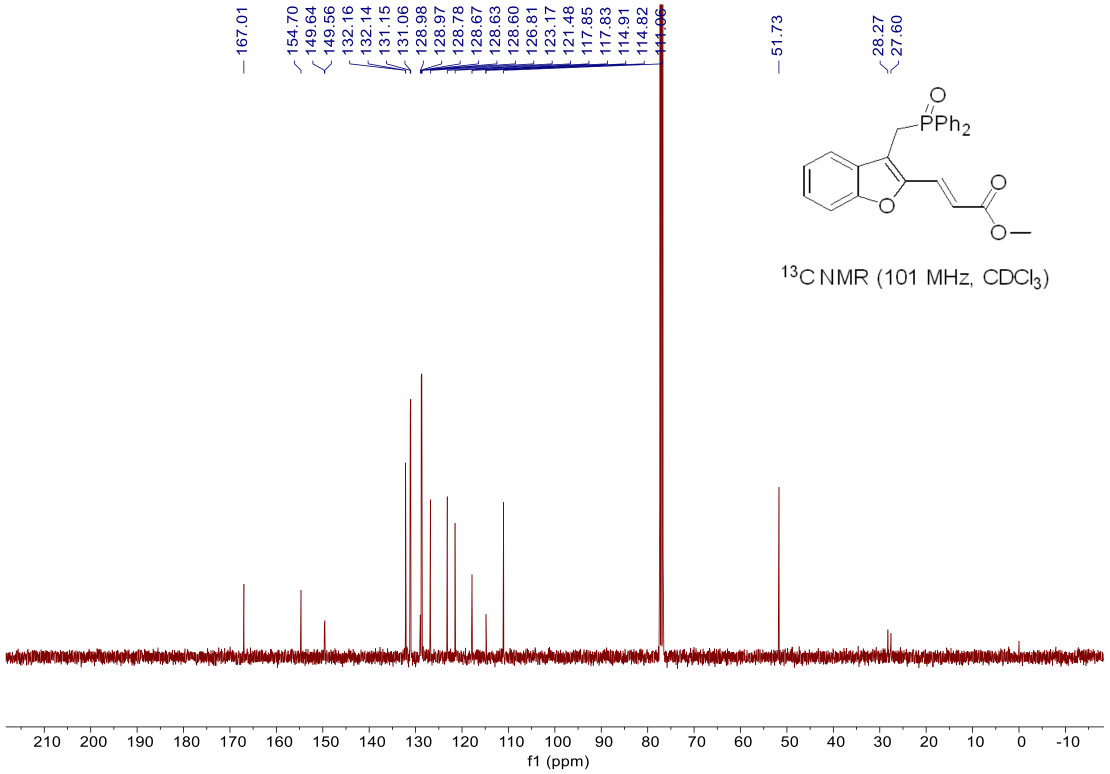

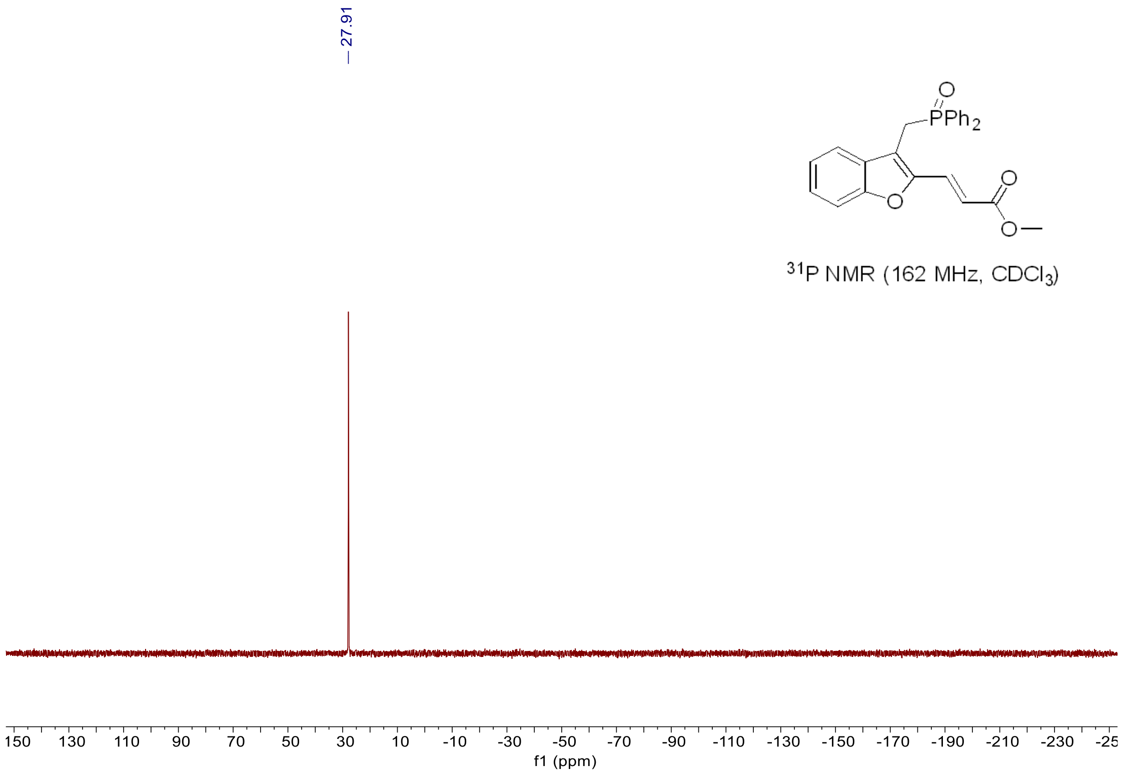
27.91
PPh2
O
O
O
O
31P NMR (162 MHz, CDCl3)
150 130 110 90 70 50 30 10 -10 -30 -50 -70 -90 -110 -130 -150 -170 -190 -210 -230 -25
f1 (ppm)

Ⅳ. Predicted Cytotoxicity of The Compounds Against Human Cells

5m

1. Physicochemical Property

Property	Value	Comment
Molecular Weight	241.06	Contain hydrogen atoms. Optimal:100~600
Volume	244.872	Van der Waals volume
Density	0.984	Density = MW / Volume
nHA	2	Number of hydrogen bond acceptors. Optimal:0~12
nHD	0	Number of hydrogen bond donors. Optimal:0~7
nRot	3	Number of rotatable bonds. Optimal:0~11
nRing	3	Number of rings. Optimal:0~6
MaxRing	9	Number of atoms in the biggest ring. Optimal:0~18
nHet	3	Number of heteroatoms. Optimal:1~15
fChar	0	Formal charge. Optimal:-4 ~4
nRig	16	Number of rigid bonds. Optimal:0~30
Flexibility	0.188	Flexibility = nRot /nRig
Stereo Centers	0	Optimal: ≤ 2
TPSA	26.03	Topological Polar Surface Area. Optimal:0~140
logS	-3.329	Log of the aqueous solubility. Optimal: -4~0.5 log mol/L
logP	3.536	Log of the octanol/water partition coefficient. Optimal: 0~3
logD	3.5	logP at physiological pH 7.4. Optimal: 1~3

2. Medicinal Chemistry

Property	Value	Decision	Comment
QED	0.645	●	■ A measure of drug-likeness based on the concept of desirability;<br>■ Attractive: > 0.67; unattractive: 0.49~0.67; too complex: < 0.34
SAscore	2.193	●	■ Synthetic accessibility score is designed to estimate ease of synthesis of drug-like molecules.<br>■ SAscore ≥ 6, difficult to synthesize; SAscore <6, easy to synthesize
Fsp3	0.071	●	■ The number of sp3 hybridized carbons / total carbon count, correlating with melting point and solubility.<br>■ Fsp^3 ≥0.42 is considered a suitable value.
MCE-18	13.0	●	■ MCE-18 stands for medicinal chemistry evolution.<br>■ MCE-18≥45 is considered a suitable value.

ADMETlab 2.0

NPscore	-0.893	-	■ Natural product-likeness score.<br>■ This score is typically in the range from –5 to 5. The higher the score is, the higher the probability is that the molecule is a NP.
Lipinski Rule	Accepted	●	■ MW ≤ 500; logP ≤ 5; Hacc ≤ 10; Hdon ≤ 5<br>■ If two properties are out of range, a poor absorption or permeability is possible, one is acceptable.
Pfizer Rule	Rejected	●	logP > 3; TPSA < 75<br>Compounds with a high log P (>3) and low TPSA (<75) are likely to be toxic.
GSK Rule	Accepted	●	■ MW ≤ 400; logP ≤ 4<br>■ Compounds satisfying the GSK rule may have a more favorable ADMET profile
Golden Triangle	Accepted	●	■ 200 ≤ MW ≤ 50; -2 ≤ logD ≤ 5<br>■ Compounds satisfying the Golden Triangle rule may have a more favorable ADMET profile.
PAINS	0 alerts	-	Pan Assay Interference Compounds, frequent hitters, Alpha-screen artifacts and reactive compound.
ALARM NMR	1 alerts	-	Thiol reactive compounds.
BMS	0 alerts	-	Undesirable, reactive compounds.
Chelator Rule	0 alerts	-	Chelating compounds.

3. Absorption

Property	Value	Decision	Comment
Caco-2 Permeability	-4.324	●	Optimal: higher than -5.15 Log unit
MDCK Permeability	2.9e-05	●	■ low permeability: < 2 × 10^{-6} cm/s<br>■ medium permeability: 2–20 × 10^{-6} cm/s<br>■ high passive permeability: > 20 × 10^{-6} cm/s
Pgp-inhibitor	0.009	●	■ Category 1: Inhibitor; Category 0: Non-inhibitor;<br>■ The output value is the probability of being Pgp-inhibitor
Pgp-substrate	0.035	●	■ Category 1: substrate; Category 0: Non-substrate;<br>■ The output value is the probability of being Pgp-substrate
HIA	0.014	●	■ Human Intestinal Absorption<br>■ Category 1: HIA+(HIA < 30%); Category 0: HIA-(HIA < 30%); The output value is the probability of being HIA+
$F_{20\%}$	0.94	●	■ 20% Bioavailability<br>■ Category 1: $F_{20\%}$+ (bioavailability < 20%); Category 0: $F_{20\%}$- (bioavailability ≥ 20%); The output value is the probability of being $F_{20\%}$+

ADMETlab 2.0

$F_{30\%}$	0.094	🟢	■ 30% Bioavailability<br>■ Category 1: $F_{30\%}$+ (bioavailability < 30%); Category 0: $F_{30\%}$- (bioavailability ≥ 30%); The output value is the probability of being $F_{30\%}$+

4. Distribution

Property	Value	Decision	Comment
PPB	95.91%	🔴	■ Plasma Protein Binding<br>■ Optimal: < 90%. Drugs with high protein-bound may have a low therapeutic index.
VD	2.734	🟢	■ Volume Distribution<br>■ Optimal: 0.04-20L/kg
BBB Penetration	0.855	🔴	■ Blood-Brain Barrier Penetration<br>■ Category 1: BBB+; Category 0: BBB-; The output value is the probability of being BBB+
Fu	3.765%	🔴	■ The fraction unbound in plasms<br>■ Low: <5%; Middle: 5~20%; High: > 20%

5. Metabolism

Property	Value	Comment
CYP1A2 inhibitor	0.996	■ Category 1: Inhibitor; Category 0: Non-inhibitor;<br>■ The output value is the probability of being inhibitor.
CYP1A2 substrate	0.353	■ Category 1: Substrate; Category 0: Non-substrate;<br>■ The output value is the probability of being substrate.
CYP2C19 inhibitor	0.978	■ Category 1: Inhibitor; Category 0: Non-inhibitor;<br>■ The output value is the probability of being inhibitor.
CYP2C19 substrate	0.1	■ Category 1: Substrate; Category 0: Non-substrate;<br>■ The output value is the probability of being substrate.
CYP2C9 inhibitor	0.936	■ Category 1: Inhibitor; Category 0: Non-inhibitor;<br>■ The output value is the probability of being inhibitor.
CYP2C9 substrate	0.642	■ Category 1: Substrate; Category 0: Non-substrate;<br>■ The output value is the probability of being substrate.
CYP2D6 inhibitor	0.967	■ Category 1: Inhibitor; Category 0: Non-inhibitor;<br>■ The output value is the probability of being inhibitor.
CYP2D6 substrate	0.849	■ Category 1: Substrate; Category 0: Non-substrate;<br>■ The output value is the probability of being substrate.
CYP3A4 inhibitor	0.953	■ Category 1: Inhibitor; Category 0: Non-inhibitor;<br>■ The output value is the probability of being inhibitor.
CYP3A4 substrate	0.48	■ Category 1: Substrate; Category 0: Non-substrate;<br>■ The output value is the probability of being substrate.

ADMETlab 2.0

6. Excretion

Property	Value	Decision	Comment
CL	11.433	●	■ Clearance<br>■ High: >15 mL/min/kg; moderate: 5-15 mL/min/kg; low: <5 mL/min/kg
$T_{1/2}$	0.509	-	■ Category 1: long half-life ; Category 0: short half-life;<br>■ long half-life: >3h; short half-life: <3h<br>■ The output value is the probability of having long half-life.

7. Toxicity

Property	Value	Decision	Comment
hERG Blockers	0.208	●	■ Category 1: active; Category 0: inactive;<br>■ The output value is the probability of being active.
H-HT	0.149	●	■ Human Hepatotoxicity<br>■ Category 1: H-HT positive(+); Category 0: H-HT negative(-);<br>■ The output value is the probability of being toxic.
DILI	0.959	●	■ Drug Induced Liver Injury.<br>■ Category 1: drugs with a high risk of DILI; Category 0: drugs with no risk of DILI. The output value is the probability of being toxic.
AMES Toxicity	0.123	●	■ Category 1: Ames positive(+); Category 0: Ames negative(-);<br>■ The output value is the probability of being toxic.
Rat Oral Acute Toxicity	0.689	●	■ Category 0: low-toxicity; Category 1: high-toxicity;<br>■ The output value is the probability of being highly toxic.
FDAMDD	0.083	●	■ Maximum Recommended Daily Dose<br>■ Category 1: FDAMDD (+); Category 0: FDAMDD (-)<br>■ The output value is the probability of being positive.
Skin Sensiti zation	0.693	●	■ Category 1: Sensitizer; Category 0: Non-sensitizer;<br>■ The output value is the probability of being sensitizer.
Carcinogen city	0.206	●	■ Category 1: carcinogens; Category 0: non-carcinogens;<br>■ The output value is the probability of being toxic.
Eye Corrosion	0.016	●	■ Category 1: corrosives ; Category 0: noncorrosives<br>■ The output value is the probability of being corrosives.
Eye Irritation	0.954	●	■ Category 1: irritants ; Category 0: nonirritants<br>■ The output value is the probability of being irritants.

ADMETlab 2.0

Respiratory Toxicity	0.91	●	■ Category 1: respiratory toxicants; Category 0: respiratory nontoxicants<br>■ The output value is the probability of being toxic.

8. Environmental toxicity

Property	Value	Comment
Bioconcentration Factors	2.18	■ Bioconcentration factors are used for considering secondary poisoning potential and assessing risks to human health via the food chain.<br>■ The unit is –log10[(mg/L)/(1000*MW)]
IGC_{50}	4.546	■ Tetrahymena pyriformis 50 percent growth inhibition concentration<br>■ The unit is –log10[(mg/L)/(1000*MW)]
$LC_{50}FM$	5.845	■ 96-hour fathead minnow 50 percent lethal concentration<br>■ The unit is –log10[(mg/L)/(1000*MW)]
$LC_{50}DM$	5.835	■ 48-hour daphnia magna 50 percent lethal concentration<br>■ The unit is –log10[(mg/L)/(1000*MW)]

9. Tox21 pathway

Property	Value	Decision	Comment
NR-AR	0.028	●	■ Androgen receptor<br>■ Category 1: actives ; Category 0: inactives;<br>■ The output value is the probability of being active.
NR-AR-LBD	0.004	●	■ Androgen receptor ligand-binding domain<br>■ Category 1: actives ; Category 0: inactives;<br>■ The output value is the probability of being active.
NR-AhR	0.908	●	■ Aryl hydrocarbon receptor<br>■ Category 1: actives ; Category 0: inactives;<br>■ The output value is the probability of being active.
NR-Aromatase	0.937	●	■ Category 1: actives ; Category 0: inactives;<br>■ The output value is the probability of being active.
NR-ER	0.624	●	■ Estrogen receptor<br>■ Category 1: actives ; Category 0: inactives;<br>■ The output value is the probability of being active.
NR-ER-LBD	0.124	●	■ Estrogen receptor ligand-binding domain<br>■ Category 1: actives ; Category 0: inactives;<br>■ The output value is the probability of being active.
NR-PPAR-gamma	0.049	●	■ Peroxisome proliferator-activated receptor gamma<br>■ Category 1: actives ; Category 0: inactives;<br>■ The output value is the probability of being active.
SR-ARE	0.664	●	■ Antioxidant response element<br>■ Category 1: actives ; Category 0: inactives;<br>■ The output value is the probability of being active.
SR-ATAD5	0.883	●	■ ATPase family AAA domain-containing protein 5<br>■ Category 1: actives ; Category 0: inactives;<br>■ The output value is the probability of being active.

ADMETlab 2.0

SR-HSE	0.899		■ Heat shock factor response element<br>■ Category 1: actives ; Category 0: inactives;<br>■ The output value is the probability of being active.
SR-MMP	0.532		■ Mitochondrial membrane potential<br>■ Category 1: actives ; Category 0: inactives;<br>■ The output value is the probability of being active.
SR-p53	0.576		■ Category 1: actives ; Category 0: inactives;<br>■ The output value is the probability of being active.

10. Toxicophore Rules

**Property**	**Value**	**Comment**
Acute Toxicity Rule	0 alerts	■ 20 substructures<br>■ acute toxicity during oral administration
Genotoxic Carcinogenicity Rule	0 alerts	■ 117 substructures<br>■ carcinogenicity or mutagenicity
NonGenotoxic Carcinogenicity Rule	0 alerts	■ 23 substructures<br>■ carcinogenicity through nongenotoxic mechanisms
Skin Sensitization Rule	1 alerts	■ 155 substructures<br>■ skin irritation
Aquatic Toxicity Rule	0 alerts	■ 99 substructures<br>■ toxicity to liquid(water)
NonBiodegradable Rule	1 alerts	■ 19 substructures<br>■ non-biodegradable
SureChEMBL Rule	0 alerts	■ 164 substructures<br>■ MedChem unfriendly status

ADMETlab 2.0

1. Physicochemical Property

Property	Value	Comment
Molecular Weight	204.06	Contain hydrogen atoms. Optimal:100~600
Volume	213.112	Van der Waals volume
Density	0.958	Density = MW / Volume
nHA	1	Number of hydrogen bond acceptors. Optimal:0~12
nHD	0	Number of hydrogen bond donors. Optimal:0~7
nRot	4	Number of rotatable bonds. Optimal:0~11
nRing	2	Number of rings. Optimal:0~6
MaxRing	9	Number of atoms in the biggest ring. Optimal:0~18
nHet	2	Number of heteroatoms. Optimal:1~15
fChar	0	Formal charge. Optimal:-4 ~4
nRig	11	Number of rigid bonds. Optimal:0~30
Flexibility	0.364	Flexibility = nRot /nRig
Stereo Centers	0	Optimal: ≤ 2
TPSA	13.14	Topological Polar Surface Area. Optimal:0~140
logS	-3.782	Log of the aqueous solubility. Optimal: -4~0.5 log mol/L
logP	3.69	Log of the octanol/water partition coefficient. Optimal: 0~3
logD	3.542	logP at physiological pH 7.4. Optimal: 1~3

2. Medicinal Chemistry

Property	Value	Decision	Comment
QED	0.554	●	■ A measure of drug-likeness based on the concept of desirability; ■ Attractive: > 0.67; unattractive: 0.49~0.67; too complex: < 0.34
SAscore	2.506	●	■ Synthetic accessibility score is designed to estimate ease of synthesis of drug-like molecules. ■ SAscore ≥ 6, difficult to synthesize; SAscore <6, easy to synthesize
Fsp3	0.167	●	■ The number of sp3 hybridized carbons / total carbon count, correlating with melting point and solubility. ■ Fsp^3 ≥0.42 is considered a suitable value.
MCE-18	9.0	●	■ MCE-18 stands for medicinal chemistry evolution. ■ MCE-18≥45 is considered a suitable value.

ADMETlab 2.0

NPscore	-0.431	-	■ Natural product-likeness score.<br>■ This score is typically in the range from –5 to 5. The higher the score is, the higher the probability is that the molecule is a NP.
Lipinski Rule	Accepted	●	■ MW ≤ 500; logP ≤ 5; Hacc ≤ 10; Hdon ≤ 5<br>■ If two properties are out of range, a poor absorption or permeability is possible, one is acceptable.
Pfizer Rule	Rejected	●	logP > 3; TPSA < 75<br>Compounds with a high log P (>3) and low TPSA (<75) are likely to be toxic.
GSK Rule	Accepted	●	■ MW ≤ 400; logP ≤ 4<br>■ Compounds satisfying the GSK rule may have a more favorable ADMET profile
Golden Triangle	Accepted	●	■ 200 ≤ MW ≤ 50; -2 ≤ logD ≤ 5<br>■ Compounds satisfying the Golden Triangle rule may have a more favorable ADMET profile.
PAINS	0 alerts	-	Pan Assay Interference Compounds, frequent hitters, Alpha-screen artifacts and reactive compound.
ALARM NMR	1 alerts	-	Thiol reactive compounds.
BMS	0 alerts	-	Undesirable, reactive compounds.
Chelator Rule	0 alerts	-	Chelating compounds.

3. Absorption

Property	Value	Decision	Comment
Caco-2 Permeability	-4.62	●	Optimal: higher than -5.15 Log unit
MDCK Permeability	2.6e-05	●	■ low permeability: < 2 × 10^{-6} cm/s<br>■ medium permeability: 2–20 × 10^{-6} cm/s<br>■ high passive permeability: > 20 × 10^{-6} cm/s
Pgp-inhibitor	0.217	●	■ Category 1: Inhibitor; Category 0: Non-inhibitor;<br>■ The output value is the probability of being Pgp-inhibitor
Pgp-substrate	0.001	●	■ Category 1: substrate; Category 0: Non-substrate;<br>■ The output value is the probability of being Pgp-substrate
HIA	0.004	●	■ Human Intestinal Absorption<br>■ Category 1: HIA+(HIA < 30%); Category 0: HIA-(HIA < 30%); The output value is the probability of being HIA+
$F_{20\%}$	0.032	●	■ 20% Bioavailability<br>■ Category 1: $F_{20\%}$+ (bioavailability < 20%); Category 0: $F_{20\%}$- (bioavailability ≥ 20%); The output value is the probability of being $F_{20\%}$+

ADMETlab 2.0

$F_{30\%}$	0.28	●	■ 30% Bioavailability<br>■ Category 1: $F_{30\%}$+ (bioavailability < 30%); Category 0: $F_{30\%}$- (bioavailability ≥ 30%); The output value is the probability of being $F_{30\%}$+

4. Distribution

Property	Value	Decision	Comment
PPB	97.19%	●	■ Plasma Protein Binding<br>■ Optimal: < 90%. Drugs with high protein-bound may have a low therapeutic index.
VD	1.215	●	■ Volume Distribution<br>■ Optimal: 0.04-20L/kg
BBB Penetration	0.184	●	■ Blood-Brain Barrier Penetration<br>■ Category 1: BBB+; Category 0: BBB-; The output value is the probability of being BBB+
Fu	2.031%	●	■ The fraction unbound in plasms<br>■ Low: <5%; Middle: 5~20%; High: > 20%

5. Metabolism

Property	Value	Comment
CYP1A2 inhibitor	0.989	■ Category 1: Inhibitor; Category 0: Non-inhibitor;<br>■ The output value is the probability of being inhibitor.
CYP1A2 substrate	0.719	■ Category 1: Substrate; Category 0: Non-substrate;<br>■ The output value is the probability of being substrate.
CYP2C19 inhibitor	0.95	■ Category 1: Inhibitor; Category 0: Non-inhibitor;<br>■ The output value is the probability of being inhibitor.
CYP2C19 substrate	0.164	■ Category 1: Substrate; Category 0: Non-substrate;<br>■ The output value is the probability of being substrate.
CYP2C9 inhibitor	0.374	■ Category 1: Inhibitor; Category 0: Non-inhibitor;<br>■ The output value is the probability of being inhibitor.
CYP2C9 substrate	0.833	■ Category 1: Substrate; Category 0: Non-substrate;<br>■ The output value is the probability of being substrate.
CYP2D6 inhibitor	0.905	■ Category 1: Inhibitor; Category 0: Non-inhibitor;<br>■ The output value is the probability of being inhibitor.
CYP2D6 substrate	0.927	■ Category 1: Substrate; Category 0: Non-substrate;<br>■ The output value is the probability of being substrate.
CYP3A4 inhibitor	0.885	■ Category 1: Inhibitor; Category 0: Non-inhibitor;<br>■ The output value is the probability of being inhibitor.
CYP3A4 substrate	0.266	■ Category 1: Substrate; Category 0: Non-substrate;<br>■ The output value is the probability of being substrate.

ADMETlab 2.0

6. Excretion

Property	Value	Decision	Comment
CL	12.219	●	■ Clearance<br>■ High: >15 mL/min/kg; moderate: 5-15 mL/min/kg; low: <5 mL/min/kg
$T_{1/2}$	0.768	-	■ Category 1: long half-life ; Category 0: short half-life;<br>■ long half-life: >3h; short half-life: <3h<br>■ The output value is the probability of having long half-life.

7. Toxicity

Property	Value	Decision	Comment
hERG Blockers	0.014	●	■ Category 1: active; Category 0: inactive;<br>■ The output value is the probability of being active.
H-HT	0.078	●	■ Human Hepatotoxicity<br>■ Category 1: H-HT positive(+); Category 0: H-HT negative(-);<br>■ The output value is the probability of being toxic.
DILI	0.571	●	■ Drug Induced Liver Injury.<br>■ Category 1: drugs with a high risk of DILI; Category 0: drugs with no risk of DILI. The output value is the probability of being toxic.
AMES Toxicity	0.106	●	■ Category 1: Ames positive(+); Category 0: Ames negative(-);<br>■ The output value is the probability of being toxic.
Rat Oral Acute Toxicity	0.112	●	■ Category 0: low-toxicity; Category 1: high-toxicity;<br>■ The output value is the probability of being highly toxic.
FDAMDD	0.046	●	■ Maximum Recommended Daily Dose<br>■ Category 1: FDAMDD (+); Category 0: FDAMDD (-)<br>■ The output value is the probability of being positive.
Skin Sensiti zation	0.868	●	■ Category 1: Sensitizer; Category 0: Non-sensitizer;<br>■ The output value is the probability of being sensitizer.
Carcinogen city	0.838	●	■ Category 1: carcinogens; Category 0: non-carcinogens;<br>■ The output value is the probability of being toxic.
Eye Corrosion	0.138	●	■ Category 1: corrosives ; Category 0: noncorrosives<br>■ The output value is the probability of being corrosives.
Eye Irritation	0.983	●	■ Category 1: irritants ; Category 0: nonirritants<br>■ The output value is the probability of being irritants.

ADMETlab 2.0

Respiratory Toxicity	0.166	🟢	■ Category 1: respiratory toxicants; Category 0: respiratory nontoxicants<br>■ The output value is the probability of being toxic.

8. Environmental toxicity

Property	Value	Comment
Bioconcentration Factors	2.33	■ Bioconcentration factors are used for considering secondary poisoning potential and assessing risks to human health via the food chain.<br>■ The unit is –log10[(mg/L)/(1000*MW)]
IGC_{50}	3.607	■ Tetrahymena pyriformis 50 percent growth inhibition concentration<br>■ The unit is –log10[(mg/L)/(1000*MW)]
$LC_{50}FM$	4.713	■ 96-hour fathead minnow 50 percent lethal concentration<br>■ The unit is –log10[(mg/L)/(1000*MW)]
$LC_{50}DM$	4.936	■ 48-hour daphnia magna 50 percent lethal concentration<br>■ The unit is –log10[(mg/L)/(1000*MW)]

9. Tox21 pathway

Property	Value	Decision	Comment
NR-AR	0.016	🟢	■ Androgen receptor<br>■ Category 1: actives ; Category 0: inactives;<br>■ The output value is the probability of being active.
NR-AR-LBD	0.011	🟢	■ Androgen receptor ligand-binding domain<br>■ Category 1: actives ; Category 0: inactives;<br>■ The output value is the probability of being active.
NR-AhR	0.721	🔴	■ Aryl hydrocarbon receptor<br>■ Category 1: actives ; Category 0: inactives;<br>■ The output value is the probability of being active.
NR-Aromatase	0.021	🟢	■ Category 1: actives ; Category 0: inactives;<br>■ The output value is the probability of being active.
NR-ER	0.852	🔴	■ Estrogen receptor<br>■ Category 1: actives ; Category 0: inactives;<br>■ The output value is the probability of being active.
NR-ER-LBD	0.016	🟢	■ Estrogen receptor ligand-binding domain<br>■ Category 1: actives ; Category 0: inactives;<br>■ The output value is the probability of being active.
NR-PPAR-gamma	0.12	🟢	■ Peroxisome proliferator-activated receptor gamma<br>■ Category 1: actives ; Category 0: inactives;<br>■ The output value is the probability of being active.
SR-ARE	0.28	🟢	■ Antioxidant response element<br>■ Category 1: actives ; Category 0: inactives;<br>■ The output value is the probability of being active.
SR-ATAD5	0.4	🟠	■ ATPase family AAA domain-containing protein 5<br>■ Category 1: actives ; Category 0: inactives;<br>■ The output value is the probability of being active.

ADMETlab 2.0

SR-HSE	0.038	●	■ Heat shock factor response element<br>■ Category 1: actives ; Category 0: inactives;<br>■ The output value is the probability of being active.
SR-MMP	0.096	●	■ Mitochondrial membrane potential<br>■ Category 1: actives ; Category 0: inactives;<br>■ The output value is the probability of being active.
SR-p53	0.283	●	■ Category 1: actives ; Category 0: inactives;<br>■ The output value is the probability of being active.

10. Toxicophore Rules

Property	Value	Comment
Acute Toxicity Rule	0 alerts	■ 20 substructures<br>■ acute toxicity during oral administration
Genotoxic Carcinogenicity Rule	0 alerts	■ 117 substructures<br>■ carcinogenicity or mutagenicity
NonGenotoxic Carcinogenicity Rule	0 alerts	■ 23 substructures<br>■ carcinogenicity through nongenotoxic mechanisms
Skin Sensitization Rule	0 alerts	■ 155 substructures<br>■ skin irritation
Aquatic Toxicity Rule	0 alerts	■ 99 substructures<br>■ toxicity to liquid(water)
NonBiodegradable Rule	0 alerts	■ 19 substructures<br>■ non-biodegradable
SureChEMBL Rule	0 alerts	■ 164 substructures<br>■ MedChem unfriendly status

ADMETlab 2.0

1. Physicochemical Property

Property	Value	Comment
Molecular Weight	291.09	Contain hydrogen atoms. Optimal:100~600
Volume	279.157	Van der Waals volume
Density	1.043	Density = MW / Volume
nHA	2	Number of hydrogen bond acceptors. Optimal:0~12
nHD	1	Number of hydrogen bond donors. Optimal:0~7
nRot	4	Number of rotatable bonds. Optimal:0~11
nRing	3	Number of rings. Optimal:0~6
MaxRing	9	Number of atoms in the biggest ring. Optimal:0~18
nHet	5	Number of heteroatoms. Optimal:1~15
fChar	0	Formal charge. Optimal:-4 ~4
nRig	16	Number of rigid bonds. Optimal:0~30
Flexibility	0.25	Flexibility = nRot /nRig
Stereo Centers	0	Optimal: ≤ 2
TPSA	25.17	Topological Polar Surface Area. Optimal:0~140
logS	-5.38	Log of the aqueous solubility. Optimal: -4~0.5 log mol/L
logP	4.414	Log of the octanol/water partition coefficient. Optimal: 0~3
logD	4.013	logP at physiological pH 7.4. Optimal: 1~3

2. Medicinal Chemistry

Property	Value	Decision	Comment
QED	0.729	●	■ A measure of drug-likeness based on the concept of desirability;<br>■ Attractive: > 0.67; unattractive: 0.49~0.67; too complex: < 0.34
SAscore	2.045	●	■ Synthetic accessibility score is designed to estimate ease of synthesis of drug-like molecules.<br>■ SAscore ≥ 6, difficult to synthesize; SAscore <6, easy to synthesize
Fsp3	0.125	●	■ The number of sp3 hybridized carbons / total carbon count, correlating with melting point and solubility.<br>■ Fsp^3 ≥0.42 is considered a suitable value.
MCE-18	17.0	●	■ MCE-18 stands for medicinal chemistry evolution.<br>■ MCE-18≥45 is considered a suitable value.

ADMETlab 2.0

NPscore	-1.174	-	■ Natural product-likeness score.<br>■ This score is typically in the range from –5 to 5. The higher the score is, the higher the probability is that the molecule is a NP.
Lipinski Rule	Accepted	●	■ MW ≤ 500; logP ≤ 5; Hacc ≤ 10; Hdon ≤ 5<br>■ If two properties are out of range, a poor absorption or permeability is possible, one is acceptable.
Pfizer Rule	Rejected	●	logP > 3; TPSA < 75<br>Compounds with a high log P (>3) and low TPSA (<75) are likely to be toxic.
GSK Rule	Rejected	●	■ MW ≤ 400; logP ≤ 4<br>■ Compounds satisfying the GSK rule may have a more favorable ADMET profile
Golden Triangle	Accepted	●	■ 200 ≤ MW ≤ 50; -2 ≤ logD ≤ 5<br>■ Compounds satisfying the Golden Triangle rule may have a more favorable ADMET profile.
PAINS	0 alerts	-	Pan Assay Interference Compounds, frequent hitters, Alpha-screen artifacts and reactive compound.
ALARM NMR	1 alerts	-	Thiol reactive compounds.
BMS	0 alerts	-	Undesirable, reactive compounds.
Chelator Rule	0 alerts	-	Chelating compounds.

3. Absorption

Property	Value	Decision	Comment
Caco-2 Permeability	-4.685	●	Optimal: higher than -5.15 Log unit
MDCK Permeability	1.4e-05	●	■ low permeability: < 2 × 10^{-6} cm/s<br>■ medium permeability: 2–20 × 10^{-6} cm/s<br>■ high passive permeability: > 20 × 10^{-6} cm/s
Pgp-inhibitor	0.072	●	■ Category 1: Inhibitor; Category 0: Non-inhibitor;<br>■ The output value is the probability of being Pgp-inhibitor
Pgp-substrate	0.313	●	■ Category 1: substrate; Category 0: Non-substrate;<br>■ The output value is the probability of being Pgp-substrate
HIA	0.004	●	■ Human Intestinal Absorption<br>■ Category 1: HIA+(HIA < 30%); Category 0: HIA-(HIA < 30%); The output value is the probability of being HIA+
$F_{20\%}$	0.39	●	■ 20% Bioavailability<br>■ Category 1: $F_{20\%}$+ (bioavailability < 20%); Category 0: $F_{20\%}$- (bioavailability ≥ 20%); The output value is the probability of being $F_{20\%}$+

ADMETlab 2.0

$F_{30\%}$	0.459		■ 30% Bioavailability<br>■ Category 1: $F_{30\%}$+ (bioavailability < 30%); Category 0: $F_{30\%}$- (bioavailability ≥ 30%); The output value is the probability of being $F_{30\%}$+

4. Distribution

Property	Value	Decision	Comment
PPB	98.71%		■ Plasma Protein Binding<br>■ Optimal: < 90%. Drugs with high protein-bound may have a low therapeutic index.
VD	2.876		■ Volume Distribution<br>■ Optimal: 0.04-20L/kg
BBB Penetration	0.385		■ Blood-Brain Barrier Penetration<br>■ Category 1: BBB+; Category 0: BBB-; The output value is the probability of being BBB+
Fu	0.990%		■ The fraction unbound in plasms<br>■ Low: <5%; Middle: 5~20%; High: > 20%

5. Metabolism

Property	Value	Comment
CYP1A2 inhibitor	0.984	■ Category 1: Inhibitor; Category 0: Non-inhibitor;<br>■ The output value is the probability of being inhibitor.
CYP1A2 substrate	0.833	■ Category 1: Substrate; Category 0: Non-substrate;<br>■ The output value is the probability of being substrate.
CYP2C19 inhibitor	0.969	■ Category 1: Inhibitor; Category 0: Non-inhibitor;<br>■ The output value is the probability of being inhibitor.
CYP2C19 substrate	0.068	■ Category 1: Substrate; Category 0: Non-substrate;<br>■ The output value is the probability of being substrate.
CYP2C9 inhibitor	0.394	■ Category 1: Inhibitor; Category 0: Non-inhibitor;<br>■ The output value is the probability of being inhibitor.
CYP2C9 substrate	0.36	■ Category 1: Substrate; Category 0: Non-substrate;<br>■ The output value is the probability of being substrate.
CYP2D6 inhibitor	0.848	■ Category 1: Inhibitor; Category 0: Non-inhibitor;<br>■ The output value is the probability of being inhibitor.
CYP2D6 substrate	0.903	■ Category 1: Substrate; Category 0: Non-substrate;<br>■ The output value is the probability of being substrate.
CYP3A4 inhibitor	0.822	■ Category 1: Inhibitor; Category 0: Non-inhibitor;<br>■ The output value is the probability of being inhibitor.
CYP3A4 substrate	0.146	■ Category 1: Substrate; Category 0: Non-substrate;<br>■ The output value is the probability of being substrate.

ADMETlab 2.0

6. Excretion

Property	Value	Decision	Comment
CL	8.747	●	■ Clearance<br>■ High: >15 mL/min/kg; moderate: 5-15 mL/min/kg; low: <5 mL/min/kg
$T_{1/2}$	0.105	-	■ Category 1: long half-life ; Category 0: short half-life;<br>■ long half-life: >3h; short half-life: <3h<br>■ The output value is the probability of having long half-life.

7. Toxicity

Property	Value	Decision	Comment
hERG Blockers	0.081	●	■ Category 1: active; Category 0: inactive;<br>■ The output value is the probability of being active.
H-HT	0.446	●	■ Human Hepatotoxicity<br>■ Category 1: H-HT positive(+); Category 0: H-HT negative(-);<br>■ The output value is the probability of being toxic.
DILI	0.103	●	■ Drug Induced Liver Injury.<br>■ Category 1: drugs with a high risk of DILI; Category 0: drugs with no risk of DILI. The output value is the probability of being toxic.
AMES Toxicity	0.021	●	■ Category 1: Ames positive(+); Category 0: Ames negative(-);<br>■ The output value is the probability of being toxic.
Rat Oral Acute Toxicity	0.482	●	■ Category 0: low-toxicity; Category 1: high-toxicity;<br>■ The output value is the probability of being highly toxic.
FDAMDD	0.261	●	■ Maximum Recommended Daily Dose<br>■ Category 1: FDAMDD (+); Category 0: FDAMDD (-)<br>■ The output value is the probability of being positive.
Skin Sensiti zation	0.064	●	■ Category 1: Sensitizer; Category 0: Non-sensitizer;<br>■ The output value is the probability of being sensitizer.
Carcinogen city	0.21	●	■ Category 1: carcinogens; Category 0: non-carcinogens;<br>■ The output value is the probability of being toxic.
Eye Corrosion	0.007	●	■ Category 1: corrosives ; Category 0: noncorrosives<br>■ The output value is the probability of being corrosives.
Eye Irritation	0.445	●	■ Category 1: irritants ; Category 0: nonirritants<br>■ The output value is the probability of being irritants.

ADMETlab 2.0

Respiratory Toxicity	0.719	🔴	■ Category 1: respiratory toxicants; Category 0: respiratory nontoxicants<br>■ The output value is the probability of being toxic.

8. Environmental toxicity

Property	Value	Comment
Bioconcentration Factors	2.168	■ Bioconcentration factors are used for considering secondary poisoning potential and assessing risks to human health via the food chain.<br>■ The unit is –log10[(mg/L)/(1000*MW)]
IGC_{50}	4.268	■ Tetrahymena pyriformis 50 percent growth inhibition concentration<br>■ The unit is –log10[(mg/L)/(1000*MW)]
$LC_{50}FM$	5.088	■ 96-hour fathead minnow 50 percent lethal concentration<br>■ The unit is –log10[(mg/L)/(1000*MW)]
$LC_{50}DM$	6.592	■ 48-hour daphnia magna 50 percent lethal concentration<br>■ The unit is –log10[(mg/L)/(1000*MW)]

9. Tox21 pathway

Property	Value	Decision	Comment
NR-AR	0.553	🟠	■ Androgen receptor<br>■ Category 1: actives ; Category 0: inactives;<br>■ The output value is the probability of being active.
NR-AR-LBD	0.012	🟢	■ Androgen receptor ligand-binding domain<br>■ Category 1: actives ; Category 0: inactives;<br>■ The output value is the probability of being active.
NR-AhR	0.823	🔴	■ Aryl hydrocarbon receptor<br>■ Category 1: actives ; Category 0: inactives;<br>■ The output value is the probability of being active.
NR-Aromatase	0.538	🟠	■ Category 1: actives ; Category 0: inactives;<br>■ The output value is the probability of being active.
NR-ER	0.792	🔴	■ Estrogen receptor<br>■ Category 1: actives ; Category 0: inactives;<br>■ The output value is the probability of being active.
NR-ER-LBD	0.022	🟢	■ Estrogen receptor ligand-binding domain<br>■ Category 1: actives ; Category 0: inactives;<br>■ The output value is the probability of being active.
NR-PPAR-gamma	0.017	🟢	■ Peroxisome proliferator-activated receptor gamma<br>■ Category 1: actives ; Category 0: inactives;<br>■ The output value is the probability of being active.
SR-ARE	0.637	🟠	■ Antioxidant response element<br>■ Category 1: actives ; Category 0: inactives;<br>■ The output value is the probability of being active.
SR-ATAD5	0.335	🟠	■ ATPase family AAA domain-containing protein 5<br>■ Category 1: actives ; Category 0: inactives;<br>■ The output value is the probability of being active.

ADMETlab 2.0

SR-HSE	0.087	●	■ Heat shock factor response element<br>■ Category 1: actives ; Category 0: inactives;<br>■ The output value is the probability of being active.
SR-MMP	0.68	●	■ Mitochondrial membrane potential<br>■ Category 1: actives ; Category 0: inactives;<br>■ The output value is the probability of being active.
SR-p53	0.474	●	■ Category 1: actives ; Category 0: inactives;<br>■ The output value is the probability of being active.

10. Toxicophore Rules

Property	Value	Comment
Acute Toxicity Rule	0 alerts	■ 20 substructures<br>■ acute toxicity during oral administration
Genotoxic Carcinogenicity Rule	1 alerts	■ 117 substructures<br>■ carcinogenicity or mutagenicity
NonGenotoxic Carcinogenicity Rule	0 alerts	■ 23 substructures<br>■ carcinogenicity through nongenotoxic mechanisms
Skin Sensitization Rule	2 alerts	■ 155 substructures<br>■ skin irritation
Aquatic Toxicity Rule	2 alerts	■ 99 substructures<br>■ toxicity to liquid(water)
NonBiodegradable Rule	1 alerts	■ 19 substructures<br>■ non-biodegradable
SureChEMBL Rule	0 alerts	■ 164 substructures<br>■ MedChem unfriendly status

ADMETlab 2.0

1. Physicochemical Property

Property	Value	Comment
Molecular Weight	118.04	Contain hydrogen atoms. Optimal:100~600
Volume	128.056	Van der Waals volume
Density	0.922	Density = MW / Volume
nHA	1	Number of hydrogen bond acceptors. Optimal:0~12
nHD	0	Number of hydrogen bond donors. Optimal:0~7
nRot	0	Number of rotatable bonds. Optimal:0~11
nRing	2	Number of rings. Optimal:0~6
MaxRing	9	Number of atoms in the biggest ring. Optimal:0~18
nHet	1	Number of heteroatoms. Optimal:1~15
fChar	0	Formal charge. Optimal:-4 ~4
nRig	10	Number of rigid bonds. Optimal:0~30
Flexibility	0.0	Flexibility = nRot /nRig
Stereo Centers	0	Optimal: ≤ 2
TPSA	13.14	Topological Polar Surface Area. Optimal:0~140
logS	-2.684	Log of the aqueous solubility. Optimal: -4~0.5 log mol/L
logP	2.578	Log of the octanol/water partition coefficient. Optimal: 0~3
logD	2.802	logP at physiological pH 7.4. Optimal: 1~3

2. Medicinal Chemistry

Property	Value	Decision	Comment
QED	0.517	●	■ A measure of drug-likeness based on the concept of desirability;<br>■ Attractive: > 0.67; unattractive: 0.49~0.67; too complex: < 0.34
SAscore	1.804	●	■ Synthetic accessibility score is designed to estimate ease of synthesis of drug-like molecules.<br>■ SAscore ≥ 6, difficult to synthesize; SAscore <6, easy to synthesize
Fsp3	0.0	●	■ The number of sp3 hybridized carbons / total carbon count, correlating with melting point and solubility.<br>■ Fsp^3 ≥0.42 is considered a suitable value.
MCE-18	8.0	●	■ MCE-18 stands for medicinal chemistry evolution.<br>■ MCE-18≥45 is considered a suitable value.

ADMETlab 2.0

NPscore	0.392	-	■ Natural product-likeness score.<br>■ This score is typically in the range from –5 to 5. The higher the score is, the higher the probability is that the molecule is a NP.
Lipinski Rule	Accepted	🟢	■ MW ≤ 500; logP ≤ 5; Hacc ≤ 10; Hdon ≤ 5<br>■ If two properties are out of range, a poor absorption or permeability is possible, one is acceptable.
Pfizer Rule	Accepted	🟢	logP > 3; TPSA < 75<br>Compounds with a high log P (>3) and low TPSA (<75) are likely to be toxic.
GSK Rule	Accepted	🟢	■ MW ≤ 400; logP ≤ 4<br>■ Compounds satisfying the GSK rule may have a more favorable ADMET profile
Golden Triangle	Rejected	🔴	■ 200 ≤ MW ≤ 50; -2 ≤ logD ≤ 5<br>■ Compounds satisfying the Golden Triangle rule may have a more favorable ADMET profile.
PAINS	0 alerts	-	Pan Assay Interference Compounds, frequent hitters, Alpha-screen artifacts and reactive compound.
ALARM NMR	0 alerts	-	Thiol reactive compounds.
BMS	0 alerts	-	Undesirable, reactive compounds.
Chelator Rule	0 alerts	-	Chelating compounds.

3. Absorption

**Property**	**Value**	**Decision**	**Comment**
Caco-2 Permeability	-4.214	🟢	Optimal: higher than -5.15 Log unit
MDCK Permeability	2.7e-05	🟢	■ low permeability: < 2 × 10^{-6} cm/s<br>■ medium permeability: 2–20 × 10^{-6} cm/s<br>■ high passive permeability: > 20 × 10^{-6} cm/s
Pgp-inhibitor	0.002	🟢	■ Category 1: Inhibitor; Category 0: Non-inhibitor;<br>■ The output value is the probability of being Pgp-inhibitor
Pgp-substrate	0.162	🟢	■ Category 1: substrate; Category 0: Non-substrate;<br>■ The output value is the probability of being Pgp-substrate
HIA	0.005	🟢	■ Human Intestinal Absorption<br>■ Category 1: HIA+(HIA < 30%); Category 0: HIA-(HIA < 30%); The output value is the probability of being HIA+
$F_{20\%}$	0.967	🔴	■ 20% Bioavailability<br>■ Category 1: $F_{20\%}$+ (bioavailability < 20%); Category 0: $F_{20\%}$- (bioavailability ≥ 20%); The output value is the probability of being $F_{20\%}$+

ADMETlab 2.0

$F_{30\%}$	0.335	●	■ 30% Bioavailability<br>■ Category 1: $F_{30\%}$+ (bioavailability < 30%); Category 0: $F_{30\%}$- (bioavailability ≥ 30%); The output value is the probability of being $F_{30\%}$+

4. Distribution

Property	Value	Decision	Comment
PPB	93.63%	●	■ Plasma Protein Binding<br>■ Optimal: < 90%. Drugs with high protein-bound may have a low therapeutic index.
VD	1.57	●	■ Volume Distribution<br>■ Optimal: 0.04-20L/kg
BBB Penetration	0.48	●	■ Blood-Brain Barrier Penetration<br>■ Category 1: BBB+; Category 0: BBB-; The output value is the probability of being BBB+
Fu	8.825%	●	■ The fraction unbound in plasms<br>■ Low: <5%; Middle: 5~20%; High: > 20%

5. Metabolism

Property	Value	Comment
CYP1A2 inhibitor	0.989	■ Category 1: Inhibitor; Category 0: Non-inhibitor;<br>■ The output value is the probability of being inhibitor.
CYP1A2 substrate	0.84	■ Category 1: Substrate; Category 0: Non-substrate;<br>■ The output value is the probability of being substrate.
CYP2C19 inhibitor	0.841	■ Category 1: Inhibitor; Category 0: Non-inhibitor;<br>■ The output value is the probability of being inhibitor.
CYP2C19 substrate	0.195	■ Category 1: Substrate; Category 0: Non-substrate;<br>■ The output value is the probability of being substrate.
CYP2C9 inhibitor	0.156	■ Category 1: Inhibitor; Category 0: Non-inhibitor;<br>■ The output value is the probability of being inhibitor.
CYP2C9 substrate	0.383	■ Category 1: Substrate; Category 0: Non-substrate;<br>■ The output value is the probability of being substrate.
CYP2D6 inhibitor	0.139	■ Category 1: Inhibitor; Category 0: Non-inhibitor;<br>■ The output value is the probability of being inhibitor.
CYP2D6 substrate	0.624	■ Category 1: Substrate; Category 0: Non-substrate;<br>■ The output value is the probability of being substrate.
CYP3A4 inhibitor	0.031	■ Category 1: Inhibitor; Category 0: Non-inhibitor;<br>■ The output value is the probability of being inhibitor.
CYP3A4 substrate	0.257	■ Category 1: Substrate; Category 0: Non-substrate;<br>■ The output value is the probability of being substrate.

ADMETlab 2.0

6. Excretion

Property	Value	Decision	Comment
CL	12.144	●	■ Clearance<br>■ High: >15 mL/min/kg; moderate: 5-15 mL/min/kg; low: <5 mL/min/kg
$T_{1/2}$	0.682	-	■ Category 1: long half-life ; Category 0: short half-life;<br>■ long half-life: >3h; short half-life: <3h<br>■ The output value is the probability of having long half-life.

7. Toxicity

Property	Value	Decision	Comment
hERG Blockers	0.083	●	■ Category 1: active; Category 0: inactive;<br>■ The output value is the probability of being active.
H-HT	0.058	●	■ Human Hepatotoxicity<br>■ Category 1: H-HT positive(+); Category 0: H-HT negative(-);<br>■ The output value is the probability of being toxic.
DILI	0.573	●	■ Drug Induced Liver Injury.<br>■ Category 1: drugs with a high risk of DILI; Category 0: drugs with no risk of DILI. The output value is the probability of being toxic.
AMES Toxicity	0.311	●	■ Category 1: Ames positive(+); Category 0: Ames negative(-);<br>■ The output value is the probability of being toxic.
Rat Oral Acute Toxicity	0.891	●	■ Category 0: low-toxicity; Category 1: high-toxicity;<br>■ The output value is the probability of being highly toxic.
FDAMDD	0.031	●	■ Maximum Recommended Daily Dose<br>■ Category 1: FDAMDD (+); Category 0: FDAMDD (-)<br>■ The output value is the probability of being positive.
Skin Sensiti zation	0.198	●	■ Category 1: Sensitizer; Category 0: Non-sensitizer;<br>■ The output value is the probability of being sensitizer.
Carcinogen city	0.821	●	■ Category 1: carcinogens; Category 0: non-carcinogens;<br>■ The output value is the probability of being toxic.
Eye Corrosion	0.912	●	■ Category 1: corrosives ; Category 0: noncorrosives<br>■ The output value is the probability of being corrosives.
Eye Irritation	0.995	●	■ Category 1: irritants ; Category 0: nonirritants<br>■ The output value is the probability of being irritants.

ADMETlab 2.0

Respiratory Toxicity	0.832	●	■ Category 1: respiratory toxicants; Category 0: respiratory nontoxicants<br>■ The output value is the probability of being toxic.

8. Environmental toxicity

Property	Value	Comment
Bioconcentration Factors	1.563	■ Bioconcentration factors are used for considering secondary poisoning potential and assessing risks to human health via the food chain.<br>■ The unit is –log10[(mg/L)/(1000*MW)]
IGC_{50}	3.232	■ Tetrahymena pyriformis 50 percent growth inhibition concentration<br>■ The unit is –log10[(mg/L)/(1000*MW)]
$LC_{50}FM$	3.94	■ 96-hour fathead minnow 50 percent lethal concentration<br>■ The unit is –log10[(mg/L)/(1000*MW)]
$LC_{50}DM$	4.735	■ 48-hour daphnia magna 50 percent lethal concentration<br>■ The unit is –log10[(mg/L)/(1000*MW)]

9. Tox21 pathway

Property	Value	Decision	Comment
NR-AR	0.016	●	■ Androgen receptor<br>■ Category 1: actives ; Category 0: inactives;<br>■ The output value is the probability of being active.
NR-AR-LBD	0.008	●	■ Androgen receptor ligand-binding domain<br>■ Category 1: actives ; Category 0: inactives;<br>■ The output value is the probability of being active.
NR-AhR	0.027	●	■ Aryl hydrocarbon receptor<br>■ Category 1: actives ; Category 0: inactives;<br>■ The output value is the probability of being active.
NR-Aromatase	0.008	●	■ Category 1: actives ; Category 0: inactives;<br>■ The output value is the probability of being active.
NR-ER	0.49	●	■ Estrogen receptor<br>■ Category 1: actives ; Category 0: inactives;<br>■ The output value is the probability of being active.
NR-ER-LBD	0.008	●	■ Estrogen receptor ligand-binding domain<br>■ Category 1: actives ; Category 0: inactives;<br>■ The output value is the probability of being active.
NR-PPAR-gamma	0.003	●	■ Peroxisome proliferator-activated receptor gamma<br>■ Category 1: actives ; Category 0: inactives;<br>■ The output value is the probability of being active.
SR-ARE	0.065	●	■ Antioxidant response element<br>■ Category 1: actives ; Category 0: inactives;<br>■ The output value is the probability of being active.
SR-ATAD5	0.048	●	■ ATPase family AAA domain-containing protein 5<br>■ Category 1: actives ; Category 0: inactives;<br>■ The output value is the probability of being active.

ADMETlab 2.0

SR-HSE	0.496	●	■ Heat shock factor response element<br>■ Category 1: actives ; Category 0: inactives;<br>■ The output value is the probability of being active.
SR-MMP	0.032	●	■ Mitochondrial membrane potential<br>■ Category 1: actives ; Category 0: inactives;<br>■ The output value is the probability of being active.
SR-p53	0.008	●	■ Category 1: actives ; Category 0: inactives;<br>■ The output value is the probability of being active.

10. Toxicophore Rules

Property	Value	Comment
Acute Toxicity Rule	0 alerts	■ 20 substructures<br>■ acute toxicity during oral administration
Genotoxic Carcinogenicity Rule	0 alerts	■ 117 substructures<br>■ carcinogenicity or mutagenicity
NonGenotoxic Carcinogenicity Rule	0 alerts	■ 23 substructures<br>■ carcinogenicity through nongenotoxic mechanisms
Skin Sensitization Rule	0 alerts	■ 155 substructures<br>■ skin irritation
Aquatic Toxicity Rule	0 alerts	■ 99 substructures<br>■ toxicity to liquid(water)
NonBiodegradable Rule	0 alerts	■ 19 substructures<br>■ non-biodegradable
SureChEMBL Rule	0 alerts	■ 164 substructures<br>■ MedChem unfriendly status

ADMETlab 2.0

Supplementary References

1. Grigg, R. et al. Palladium catalysed tandem cyclisation-anion capture processes. Part 3. Organoboron anion transfer agents. *Tetrahedron*, **53**, 11803 – 11826 (1997).
2. Liu, C. et al. Transition-Metal-Free Synthesis of Electron Rich 1, 3-Dienes via Base Promoted Isomerization of Propargylic Ethers. *Eur. J. Org. Chem.* **2020**, 483 – 487 (2020).
3. Jiang, T., Gao, J., Li, J., Yan, B., Shao, H., Chen, Y. Catalyzer for ethylene oligomerization. *China*, CN109331878 A **2019**-02-15.
4. Biallas, P. et al. The deazidoalkoxylation: Sequential nucleophilic substitutions with diazidated diethyl malonate. *J. Org. Chem.* **84**, 1654–1663 (2019).
5. Bismuto, A., Delcaillau, T., Müller, P. & Morandi, B. Nickel-catalyzed amination of aryl thioethers: A combined synthetic and mechanistic study. *ACS Catal.* **10**, 4630 – 4639 (2020).
6. Connelly, N. G., & Geiger, W. E. Chemical redox agents for organometallic chemistry. *Chem. Rev.* **96**, 877 – 910 (1996).
7. Deng, G. et al. Synthesis of Benzofuran Derivatives through Cascade Radical Cyclization / Intermolecular Coupling of 2-Azaallyls. *Angew. Chem. Int. Ed.* **58**, 2826 – 2830 (2019).
